# Proteomic Identification and Time-Course Monitoring of Secreted Proteins During Expansion of Human Mesenchymal Stem/Stromal in Stirred-Tank Bioreactor

**DOI:** 10.3389/fbioe.2019.00154

**Published:** 2019-06-26

**Authors:** Amanda Mizukami, Carolina Hassibe Thomé, Germano Aguiar Ferreira, Guilherme Pauperio Lanfredi, Dimas Tadeu Covas, Sharon J. Pitteri, Kamilla Swiech, Vitor Marcel Faça

**Affiliations:** ^1^Faculty of Medicine of Ribeirão Preto, Hemotherapy Center of Ribeirão Preto, University of São Paulo, Ribeirão Preto, Brazil; ^2^Department of Biochemistry and Immunology, Faculty of Medicine of Ribeirão Preto, University of São Paulo, Ribeirão Preto, Brazil; ^3^Department of Radiology, Canary Center at Stanford for Cancer Early Detection, Stanford University School of Medicine, Stanford, CA, United States; ^4^Department of Pharmaceutical Sciences, Faculty of Pharmaceutical Sciences of Ribeirao Preto, University of São Paulo, Ribeirão Preto, Brazil

**Keywords:** mesenchymal stem/stromal cells, umbilical cord matrix, secretome, bioreactors, proteomic analysis, mass spectrometry

## Abstract

The therapeutic potential of mesenchymal stem/stromal cells (MSC) is widely recognized for the treatment of several diseases, including acute graft-vs.-host disease (GVHD), hematological malignancies, cardiovascular, bone, and cartilage diseases. More recently, this therapeutic efficacy has been attributed to the bioactive molecules that these cells secrete (secretome), now being referred as medicinal signaling cells. This fact raises the opportunity of therapeutically using MSC-derived soluble factors rather than cells themselves, enabling their translation into the clinic. Indeed, many clinical trials are now studying the effects of MSC-secretome in the context of cell-free therapy. MSC secretome profile varies between donors, source, and culture conditions, making their therapeutic use very challenging. Therefore, identifying these soluble proteins and evaluating their production in a reproducible and scalable manner is even more relevant. In this work, we analyzed the global profile of proteins secreted by umbilical cord matrix (UCM) derived-MSC in static conditions by using mass spectrometry, enabling the identification of thousands of proteins. Afterwards, relevant proteins were chosen and monitored in the supernatant of a fully-controllable, closed and scalable system (bioreactor) by using multiple reaction monitoring (MRM) mass spectrometric technique in a time-dependent manner. The results showed that the majority of interesting proteins were enriched through time in culture, with the last day of culture being the ideal time for supernatant collection. The use of this regenerative “soup,” which is frequently discarded, could represent a step toward a safe, robust and reproducible cell-free product to be used in the medical therapeutic field. The future use of chemically defined culture-media will certainly facilitate secretome production according to Good Manufacturing Practice (GMP) standards.

## Introduction

Mesenchymal stem/stromal cells (MSC) are multipotent cells with recognized therapeutic potential for several diseases, confirmed by the exponential increase of clinical trial studies in recent years (Mao et al., 2017). Besides their differentiation versatility, immunomodulatory properties of MSC have been considered the most promising characteristic for cellular therapy applications (Le Blanc et al., 2008). MSC can be found in almost all tissues and share specific characteristics by consensus definition: spindle-shaped morphology, plastic-adherent, positive expression of CD73, CD90, and CD105, lack of expression of hematopoietic stem cell markers and multilineage differentiation (Dunavin et al., 2017).

The discovery that only a small portion of MSC (after local or systemic injection) is capable of engrafting onto the injured tissue, led to the supposition that the therapeutic effect from this therapy is directly related to its paracrine effect. Indeed, studies have shown that <1% of MSC survive for more than 1 week after infusion (Vizoso et al., 2017). This hypothesis opened up new therapeutic perspectives with the aim of developing cell-free strategies based on the use of the MSC secretome as a safe and potential alternative in the cell therapy field (Baglio et al., 2012). These paracrine or autocrine effects are related to several cellular processes (immunomodulation, chemoattraction, apoptosis, and angiogenesis), making the set of proteins secreted by MSC of great relevance. In the context of immunological disorders, MSC are capable of modifying the immune response and secreting paracrine mediators, reducing inflammation and accelerating tissue regeneration by activating resident stem cells and mobilizing systemic stem cells through chemotactic signaling (Nonnis, 2015). Thus, MSC can be considered a vehicle of multiple bioactive factors *in situ*, possessing the great advantage of being site-specific (Faça, 2012).

MSC-sourced secretome could be obtained from the expended medium after cell culture, namely conditioned medium (CM), and may represent advantages compared to living cells in terms of storage, handling, safety, and cost-effectiveness, since cell harvesting is not necessary (Vizoso et al., 2017). Moreover, the CM could be recovered from monolayer-culture, and the use of fully-controllable, closed and scalable bioreactor could limit heterogeneity, improving reproducibility and robustness, while enhancing predictability in the composition and function of secretome-derived products (Phelps et al., 2018).

The proteomic profile has been widely used to uncover the MSC secretome and allows for the large-scale investigation of secreted proteins, their function but mainly the understanding of pathological conditions and mechanisms *in vivo* (Stastna and Van Eyk, 2012). Since the MSC secretome are considered environment-dependent, and consequently dynamic, it is expected that there are multiple relevant proteins to be characterized. Based on these findings, a qualitative and quantitative analysis of the secreted proteins from these cells is a fundamental step to better understand the mechanisms underlying the therapeutic effects and to evaluate the actions of the identified components (Salgado and Gimble, 2013). So far, mass spectrometry (MS) technology allows for a more accurate identification and quantification of these extended lists of proteins, making possible in the near future a more rational application of MSC in human therapy.

The objective of this work was the identification of a globally secreted proteomic profile of MSC derived from umbilical cord matrix (UCM MSC) under static conditions using LC-MS/MS and to monitor the production of selected proteins in the bioreactor supernatant by multiple reaction monitoring (MRM) experiments during the time of bioreactor-culture.

## Materials and Methods

### Mesenchymal Stem/Stromal Cell Culture

Human umbilical cords were collected from full-term newborns (39–40 weeks, cesarean births), after informed consent from the person legally responsible for the donor. Institutional Ethical Review Board (Clinics Hospital, Ribeirão Preto Medical School, University of São Paulo, Protocol HCRP number 14906/2010). Mesenchymal stem/stromal cells derived from umbilical cord matrix (UCM MSC) were isolated according to the protocol described previously (Mizukami et al., 2013). Cells were cultured in alpha-MEM (GIBCO, Grand Island, NY) with 10% fetal bovine serum (FBS) (HyClone, Canada) at 37°C and 5% CO_2_ in a humidified atmosphere. UCM MSC at passages 4–5 were used in this study.

### Generation of Enriched Supernatant From UCM MSC

**Figure 1:**
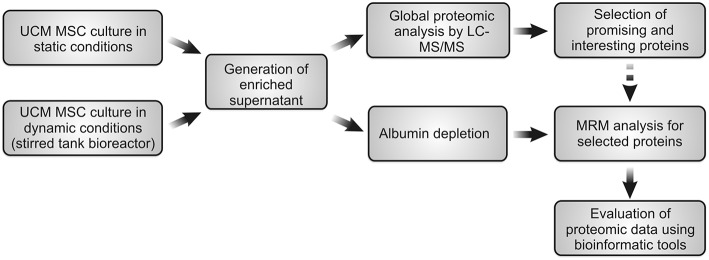
Schematic representation of global experimental procedure used in this work.

UCM MSC was cultured under static conditions and the secretome was analyzed using LC-MS/MS for global profile of secreted proteins. Afterwards, the most promising and interesting proteins were selected for further identification in the bioreactor supernatant by MRM experiments in a time-dependent manner (during time of bioreactor-culture). The schematic procedure is summarized in the Figure 1.

#### UCM MSC Culture Under Static Conditions

Static cultures (T-flasks) (*n* = 3, three different donors) were performed to generate enriched supernatants with proteins. For this, UCM MSC were culture plated at 3,000 cells/cm^2^ on 75 cm^2^ T-flasks with culture medium supplemented with 10% FBS. Upon reaching 70–80%, cells were washed with pre-warmed PBS and the media was replaced by alpha-MEM without FBS. Supernatant was collected after 48 h of culture for proteomic analysis.

#### UCM MSC Culture Under Dynamic Conditions

Stirred tank bioreactor was used to generate enriched supernatant released by UCM MSC in a closed and controlled bioprocess. The experimental procedure was described in detail in our previous report (Mizukami et al., 2018). Briefly, 5,000 cells/cm^2^ of UCM MSC (*n* = 3, three different donors) was inoculated with 2.0 g/L Cultispher^TM^ S microcarriers (Sigma Aldrich, Saint Louis, USA) in 800 mL of working volume. Fifty percentage of culture medium change was performed daily after 96 h. Thus, this remaining CM after medium exchange was used for MRM analysis. The cell culture parameters used were: pH 7.30, 20% of dissolved oxygen (DO) by headspace aeration (N_2_, O_2_ and air) and 37°C (water jacket).

### Sample Processing for Proteomic Analysis

After collecting CM (static and dynamic conditions), 5% v/v protease inhibitor mixture (product number P8340; Sigma-Aldrich, St. Louis, USA) was added. Then, the supernatant was centrifuged at 4,700 rpm for 30 min and concentrated in a centricon filter (5 kDa MWCO) (GE Healthcare, Buckinghamshire, UK). Samples were diluted in 200 μL of Urea 0.1 M/Tris-HCl 0.1 M, pH 8.0, and the total proteins were quantified by the Bradford method (Bio-Rad, Hercules, CA, USA).

#### Static Conditions: In-Gel Digestion and LC-MS/MS Analysis

After Bradford quantification, 30 μg of protein were dissolved in 30 μL Laemmli sample buffer containing dithiothreitol (1 mg/mg of total protein), boiled for 5 min to reduce disulfide bonds and alkylated with iodoacetamide (5 mg/mg of total protein). Samples were separated using a 10% SDS-PAGE (Bio-Rad, Hercules, CA, USA). Then, each gel sequence was sectioned into 6 equal fragments of 1 cm, washed 3 times with 50% acetonitrile in 50 mM ammonium bicarbonate and held overnight. The samples were then washed for 5 min with 100% acetonitrile and dried for 30 min by SpeedVac. Samples were rehydrated in 20 μL of 100 mM ammonium bicarbonate solution containing 0.5 μg of trypsin (Promega, Madison, USA). Tryptic peptides were successively extracted with 50% acetonitrile/0.1% formic acid, 70% acetonitrile/0.1% formic acid, and 100% ACN/1% formic acid. The peptide extraction solutions were dried in SpeedVac for further analysis by mass spectrometry.

Samples were analyzed in triplicate by LC-MS/MS using LTQ-ORBITRAP (Elite Model—Thermo-Finnigan) mass spectrometer coupled with a nano-LC. The liquid chromatographic separation was performed in a 25 cm column (Picofrit 75 μm ID, New Objectives), packed in the laboratory using Magic C18 resin. The separation was performed with a gradient of 5–40% acetonitrile/0.1% formic acid at a flow rate of 250 nL/min. The spectra were acquired in data-dependent mode in the m/z 400–1,800 range with selection of the 5 most abundant ions with +2 or +3 charges of each mass spectrum for the ms/ms analysis. The parameters of the mass spectrometer were: capillary voltage of 2.0 KV, capillary temperature of 200°C, resolution of 100,000 and “FT target value” of 2 × 10^6^ ions.

#### Dynamic Conditions: Multiple Reaction Monitoring (MRM) Analysis

After quantification, 200 μg of protein were dried and dissolved using buffer solution (Urea 0.1 M/Tris-HCl 0.1 M, pH 8.0). Since Fetal Bovine Serum (FBS) was used for culture medium supplementation in the bioreactor culture, an additional step of albumin depletion was performed, according to the protocol described by Kay et al. (2008). Briefly, 20 μL of the bioreactor sample was diluted with 40 μL of deionized water and vortexed. Afterwards, 90 μL of acetonitrile was added, sonicated for 10 min and centrifuged at 12,000 × g for 10 min. The supernatant was transferred to a new tube and dried by SpeedVac (Kay et al., 2008). Depletion of albumin was confirmed by SDS-PAGE gel. Then, samples were reduced, alkylated, proteins separated (SDS-PAGE) and digested (trypsin) using the same protocol described in the item 2.3.1. Samples were then desalted using solid-phase extraction columns Oasis HLB (Waters) according to the manufacturer's instructions. Peptides were eluted in 50% ACN/0.1% formic acid and dried in SpeedVac. Samples were reconstituted in 50 μL of 5% acetonitrile/0.1% formic acid solution before MRM analysis. Each sample was injected in triplicate through the LC-MS/MS Xevo TQs system (Waters). Chromatographic separation was performed in a UPLC (I-class, Waters) using a C18 column (1.8 μm particle size, 100 Å pore size, 1 mm × 150 mm, Waters) in a linear gradient of 5 to 30% acetonitrile over 15 min at 100 μL/min in a formic acid:acetonitrile:water solvent system.

### Data Analysis

The high throughput data obtained for static conditions of cell culture were processed using the Labkey suite (Rauch et al., 2006). Peptides and proteins were identified using the X!Tandem search engine (2013.2.01 release) (MacLean et al., 2006), and Peptide Prophet (Nesvizhskii et al., 2003) and Protein Prophet (Keller et al., 2002) algorithms for statistical validation of data and protein grouping. MS data were searched against a human proteome database (Uniprot April 2014 − 68,561 entries). Search parameters for tryptic peptides included up to two missed cleavages, mass tolerance of 0.5 Da for fragment ions, fixed cysteine modification with carbamidomethylation (+57.02146), variable methionine oxidation (+15.99491). Only peptides with a Peptide Prophet score above 0.90 and precursor ions with delta mass <20 ppm were considered for protein quantification. The list of proteins was generated with a Protein Prophet cut-off value of 0.90, representing an overall protein false discovery rate of approximately 1% based on the Protein Prophet estimation. The MRM data was analyzed using the Skyline v3.1 software (Pino et al., 2017). Protein concentrations were estimated based on the analysis of a standard protein solution (Bovine Serum Albumin, BSA, Biorad). For this, different concentrations (0, 1, 10, 100 pg/μL and 1 and 10 ng/μL) of BSA were diluted in an equivalent sample matrix and two calibration curves were plotted based on the monitoring of two peptides (AEFVEVTK, SLHTLFGDELCK). The linear correlation signal (peak area) × concentration was obtained by the resulting bisector of the curves from the analyzed BSA standards. Proteotypic peptides were selected for each protein based on their identification in our high throughput data and supported by SRMAtlas (http://www.srmatlas.org/). For differentially abundant proteins selected in our study (*n* = 21), MRM quantification was based on the average of total peak areas for each peptide (composed by the sum of areas of all the transitions). Supplementary Table 2 and Supplementary Figure 1 in Data Sheet 2 present detail information for the method developed. Maximum productivity of secreted proteins (ng/mL/day) was calculated based on the ratio of maximum concentration (ng/mL) divided by the corresponding time of culture. In addition, data was evaluated by several bioinformatic tools for protein-protein interactions and networks using STRING-DB (string-db.org) (Szklarczyk et al., 2018) as well as by gene ontology annotation using PANTHER tool (phanterdb.org) (Mi et al., 2017).

## Results

### Global Proteomic Profile of UCM MSC Grown (Cultivated) Under Static Conditions

As previously described in the section UCM MSC culture under static conditions, static cultures (T-flasks) (*n* = 3, three different donors) were performed to generate supernatants enriched with proteins. Then, the concentration of total protein obtained was quantified using the Bradford assay and the results obtained can be found in Table 1.

**Table 1:** Protein quantification of UCM MSC culture supernatant (*n* = 3, three independent donors) in static conditions by the Bradford method.

	**Total volume (μL)**	**Concentration (μg/μL)**	**Total mass (μg)**
Donor #1	100	1.21	121
Donor #2	100	2.49	249
Donor #3	100	1.35	135

**Figure 2:**
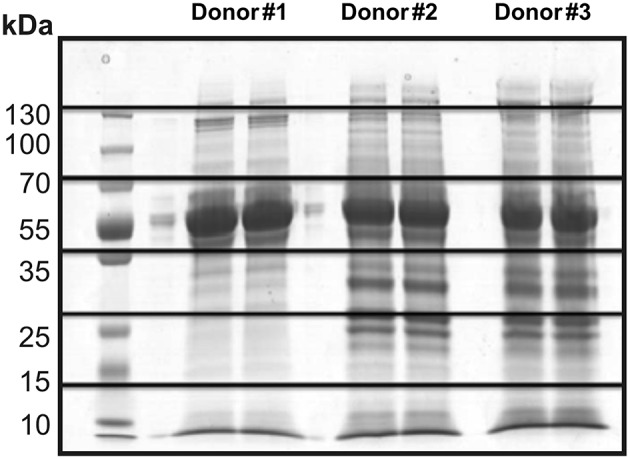
SDS-PAGE electrophoresis of the culture supernatant in static conditions from three different donors (duplicate). Illustration of the gel fragments generated after the sectioning of each sample into six equal parts.

Thus, 30 μg of proteins from each sample (duplicate) were submitted to SDS-PAGE electrophoresis for separation and characterization of the secreted proteins. As expected, a similarity in the pattern of bands was observed between the three different donors (Figure 2). Therefore, gel lanes were cut into six fragments of 1 cm each to increase the sampling of ions (peptides) detected by the mass spectrometer, thus allowing for the identification of low abundance proteins. Each fraction was subjected to in-gel trypsin digestion and individually analyzed by LC-MS/MS.

**Figure 3:**
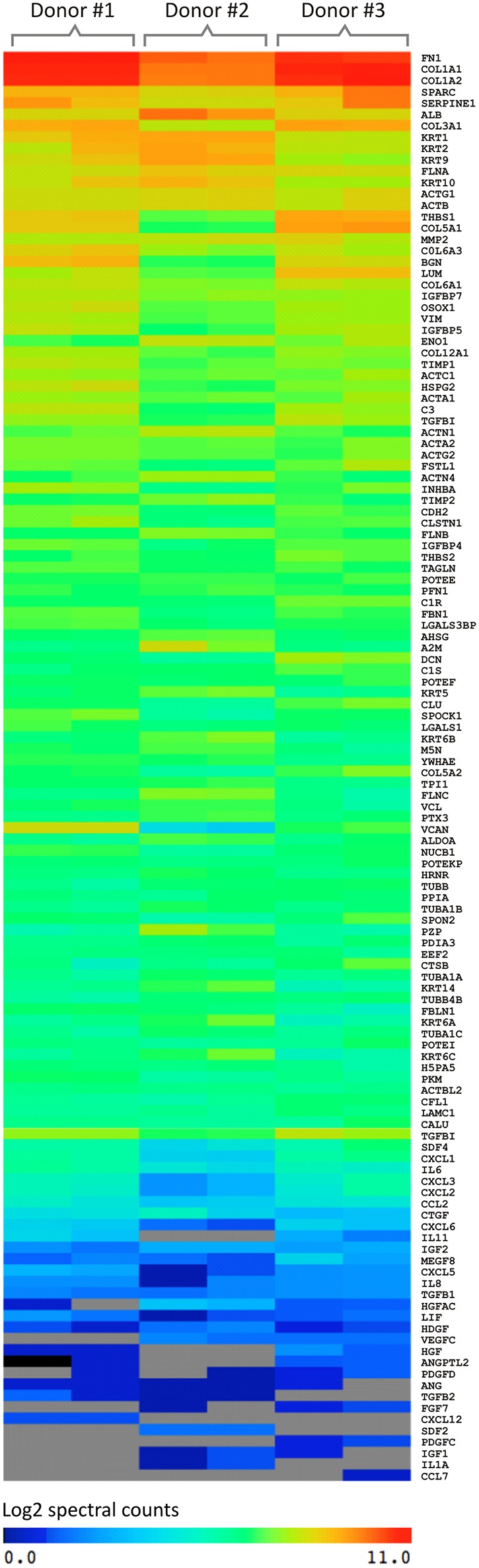
Graphical representation of the spectral count result of proteins secreted by UCM MSC using three different donors (in duplicate) analyzed by mass spectrometry (MS). The software used for this analysis was MeV (MultiExperiment Viewer). The color of the scale illustrates the relative expression of the proteins: blue denotes low expression and red, high expression.

After mass spectrometry analyses, it was possible to identify ~2,400 proteins. The complete list of protein identification is presented in Supplementary Table 1. In order to further explore the results obtained, the secreted proteins were clustered between the three different donors analyzed in duplicate based on the relative abundance (Figure 3).

A similar pattern of absolute amount of proteins secreted was observed among the donors. In addition, the secretory pattern between the replicates was very similar, increasing the reliability of the analyses. Thus, based on the spectral counting criteria, the secreted proteins were divided into 3 groups: (I) proteins of greater abundance (log 2 = 8–11), (II) intermediate abundance (log 2 = 4–8) and (III) low abundance (log 2 = 0–4).

The proteins with higher relative abundance refer mainly to extracellular matrix proteins (ECM) including COL1A2 and COL3A1 (Collagen type α-1 and 2), FN (Fibronectin), LUM (Lumican), VCAN (Versican), BGN (Biglican), DCN (Decorin), FBLN1 (Fibrilin-1), SPARC (Secreted Protein Acidic and Cysteine Rich), and TGF-β1 (Transforming Growth Factor Beta 1). Also involved in the dynamic processes of ECM production and degradation, MMPs (Metalloproteinases), and TIMPs (Inhibitors of metalloproteinases) were identified with high abundance.

Chemokines and the growth factors are among the proteins showing intermediate abundance. The major chemokines identified were CCL2 (Monocyte chemotactic protein-1), CCL6 (Small-inducible cytokine A6), CCL8 [Monocyte chemoattractant protein 2 (MCP-2)], CXCL2 [Macrophage inflammatory protein 2a (MIP2a)], CXCL5 (ENA-78), CXCL6 [Granulocyte chemotactic protein 2 (GCP-2)], and CXCL12 [Stromal cell-derived factor 1 (SDF1)]. Although IL-6 and IL-8 interleukins have been secreted in low abundance, many studies describe them as the most expressed cytokines by MSC from a variety of sources (Liu and Hwang, 2005; Hwang et al., 2009; Blaber et al., 2012). The growth factors detected were: IGF-1 and 2 (Insulin-like growth factor) and IGFBP −2, −3, −4, −5, −6, −7 (Insulin-like growth factor-binding protein).

### Monitoring of Selected Proteins Through UCM MSC Culture Under Bioreactor-Based System

Based on the list of global proteins identified in the static experiments, a set of proteins with known biological relevance was selected for further time-course quantification in the bioreactor supernatant using MRM mass spectrometric approach. The selected proteins were: COL1A2, COL3A1, COL1A1, SPARC, FN1, TGFBI, TIMP1, TIMP2, MMP1, MMP14, CCL2, CXCL1, CXCL3, IL-6, THBS1, IGFBP2, IGFBP3, IGFBP4, IGFBP5, IGFBP6, and IGFBP7. Supernatants were quantified for time points of 96, 120, 144, and 168 h of bioreactor-culture. It is important to highlight that the same donors for the global protein experiments (static system) were used herein (*n* = 3). The cell growth kinetic of the three independent donors during the time in culture is presented in the Supplementary Figure 2.

**Figure 4:**
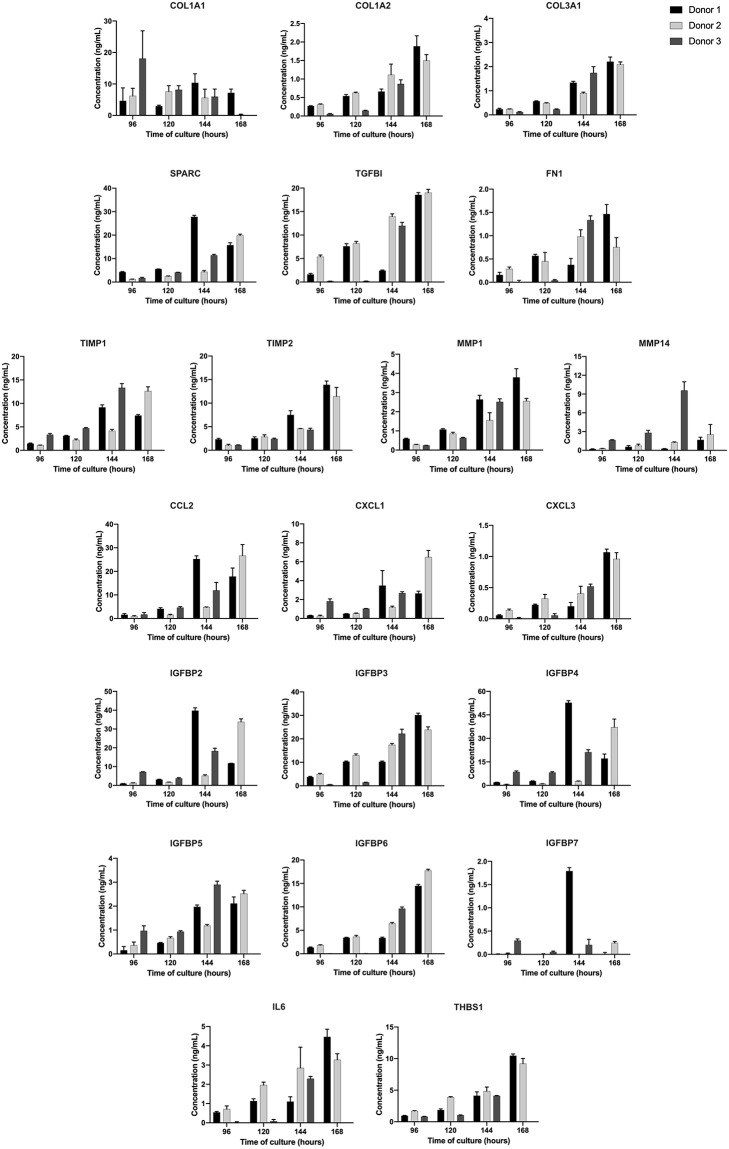
Concentration of each protein during UCM MSC expansion in stirred-tank bioreactor in the period analyzed (96, 120, 144, and 168 h). Results are expressed using three independent donors (*n* = 3). One hundred and sixty eight hours data point for donor 3 was missed.

Overall, the majority of the selected proteins were enriched during time in culture, i.e., higher concentrations at 144 and 168 h, as can be seen in Figure 4. It was observed that the rate of selected proteins secretion was varied between the donors, but the average of maximum productivity was around 2.0 ng/mL/day (Table 2).

**Table 2:** Maximum productivity (ng/mL/day) of selected proteins obtained for UCM MSC culture in the stirred-tank bioreactor (three independent donors).

	**Maximum Productivity (ng/mL/day)**		
**Proteins**	**Donor #1**	**Donor #2**	**Donor #3**
COL1A1	1.72	0.94	1.63
COL1A2	0.27	0.22	0.13
COL3A1	0.32	0.30	0.25
SPARC	4.63	2.84	1.62
TGFB1	2.65	2.71	1.71
FN1	0.21	0.11	0.19
TIMP1	1.52	1.80	1.90
TIMP2	1.98	1.63	0.62
MMP1	0.54	0.37	0.36
MMP14	0.24	0.36	1.37
CCL2	4.20	3.81	1.70
CXCL1	0.58	0.93	0.38
CXCL3	0.15	0.14	0.07
IGFBP2	6.62	4.82	2.61
IGFBP3	4.30	3.41	3.17
IGFBP4	8.78	5.29	3.03
IGFBP5	0.30	0.36	0.41
IGFBP6	2.07	2.53	1.37
IGFBP7	0.26	0.03	0.03
IL-6	0.64	0.47	0.33
THBS1	1.49	1.31	0.59

## Discussions

Over the past few years, MSC secretome has been attracting attention with its composition, since it contains many proteins involved in mechanisms of cell-cell interaction, cell-substrate adhesion, receiving and emitting signals that control and orchestrate cell migration, proliferation and differentiation. Because of such secretome relevance, these protein components necessitate a more detailed and analytically consistent quantification (Faça, 2012; Vizoso et al., 2017).

MSC derived-secretome has been explored in pre-clinical studies for treatment of fulminant hepatic failure, wound healing, periodontal tissue regeneration, bone defects and others (Parekkadan et al., 2007; Heo et al., 2011; Osugi et al., 2012; Kawai et al., 2015). However, until now, few human clinical studies have been described. Zhou et al. (2013) showed an enhancement of wound healing after fractional carbon dioxide laser resurfacing on human skin by using conditioned-medium from MSC. The first in-human clinical study was published in 2016 for alveolar bone regeneration using MSC-secretome. The results showed an initial bone formation in all cases, with no associated complications. The authors highlighted the importance of previously identified IGF-1, VEGF, TGF-β1, and HGF cytokines in the bone regeneration process and classified the approach used as safe and efficient to be used in bone regenerative medicine (Katagiri et al., 2016). One year later, the same research group published a paper on the use of MSC-secretome for maxillary sinus floor elevation (SFE) with promising outcomes (Katagiri et al., 2017). Due to a large number of approved clinical trials using MSC as a product, it is expected that MSC-secretome usage will increase rapidly, potentially being an alternative for regenerative therapy.

In this work, a proteomic approach using mass spectrometry (MS) technique allowed for the identification of thousands of proteins in the MSC supernatant under static conditions. As observed, the proteins of greater abundance are derived from the extracellular matrix (ECM), such as collagen, fibronectin, lumican, and versican, among others. These proteins actively contribute to the assembly and organization of ECM by providing a microenvironment in which signals from the cell-ECM interaction are functionally integrated to allow for the maintenance of stem-cell homeostasis (Gattazzo et al., 2014). Of note, SPARC was secreted at high levels by MSC, which has the ability to bind to several ECM resident proteins (collagen, vitronectin, thrombospondin) in order to affect the expression of MMPs and alter the cell format. Thus, SPARC acts to regulate the interaction of cells with the extracellular environment in response to injury (Bradshaw and Sage, 2001). In addition, some works have shown that SPARC could modulate osteoblast function (Chiellini et al., 2008; Choi et al., 2010). Indeed, deprivation of SPARC causes osteopenia due to decreased numbers of osteoblasts and osteoclasts, suggesting that SPARC may regulate the recruitment or proliferation of osteoblastic precursors, and therefore might represent a potential therapeutically applicable protein (Delany et al., 2000). In human tumors, SPARC could alter the activity of cancer cells, modulating cell growth, apoptosis, adhesion migration, and invasion (Han et al., 2016).

MMPs and TIMPs were also identified with high abundance in this work and actively participate in cellular physiology, such as cell communication, migration, and apoptosis. The balance between the activity of MMPs and TIMPs is involved in tissue remodeling, angiogenesis and hematopoiesis. MSC give rise to mature cells via paracrine mechanisms through the secretion of ECM that regulates cell differentiation by inter- and intra-cellular signaling. Such ECM components can coordinate the activation/deletion of genes from different MMPs capable of remodeling the matrix, culminating with cell migration to specific tissues or modulating cell grafting (Mannello, 2006; Bonnans et al., 2014). TNF is an important cytokine involved in inflammation initiation, but an excessive or prolonged release of this molecule underlies tissue damage, hemodynamic changes, organ failure, and other effects (Chen et al., 2018). TIMP3 has been postulated to protect the organism against an overactive innate immune response by inactivating this cytokine. Based on this finding, it was suggested that TIMP3-based therapies can be developed to control inflammation (Smookler et al., 2006). Although few studies have demonstrated the use of TIMPs for therapeutic interventions, their use in model systems gives essential information about the efficacy of metalloproteinase inhibitors in the resolution of disease (Murphy, 2011).

TGF-β1 protein identified in our study (intermediate abundance) is considered one of the most prominent immunomodulatory cytokines produced and constitutively secreted by MSC. TGF-β1 regulates multiple cell functions including proliferation, differentiation, migration, adhesion and apoptosis, affecting innumerable biological processes such as: scarring, carcinogenesis, angiogenesis, and immune response (Ghosh et al., 2017). The regulation of the immune response is its main activity, orchestrating the initiation and resolution of the inflammatory response, as well as the induction and maintenance of immune tolerance through leukocyte proliferation, differentiation, activation, and survival (Xu et al., 2014). This property can be seen by the inhibition of TGF-β1-mediated proliferation of T cells by MSC (Kyurkchiev et al., 2014). Nemeth et al. (2010) conducted an *in vivo* study using MSC from bone marrow in a murine model of asthma. They observed a considerable increase in the secretion of TGF-β1, which was able to suppress the allergenic response, evidencing its anti-inflammatory activity (Nemeth et al., 2010). By its ability to suppress the immune system, TGF-β could be used for therapeutic applications, especially for the treatment of wounds with impaired healing, ischemia-reperfusion injuries, mucositis, fractures, and others (Flanders and Burmester, 2003). TGF-β also display an important role in cancer progression and according to Haque and Morris, TGF-β inhibits cellular transformation and prevents cancer progression at early stages (Haque and Morris, 2017).

Thrombospondin-1 (TSP-1) has been identified as a critical factor for cell proliferation and migration. Belotti et al. (2016) described TSP-1 as being mostly responsible for proliferative capacity of MSC. This effect was mediated by TSP-1-induced activation of endogenous TGFβ. The authors found that their use could increase MSC expansion by 2–4-fold, facilitating the therapeutic application (Belotti et al., 2016). In our study, TSP-1 was secreted in a higher quantity at 168 h of culture, which was consistent with maximum cell growth (Supplementary Figure 2).

Different classes of chemokines were secreted with intermediate abundancy in this study. Chemokines are relatively small molecules (7.5–12.5 KDa) that participate in processes of inflammation, differentiation, angiogenesis, and migration of immune cells. Chemokines play an important role during *in vivo* immunomodulatory activities through the mediation of interactions between MSC and other immunocompetent cellular types (Kyurkchiev et al., 2014). MSC secrete chemokines in response to the pro-inflammatory stimulus in order to attract and activate neutrophils, monocytes, lymphocytes, NK cells, hematopoietic, endothelial progenitors, and other effector cells to the site of infection (Kyurkchiev et al., 2014). Ren et al. (2008) demonstrated that the chemokines CXCL9, CXCL10, and CXCL11 produced by MSC stimulate the migration of T cells, causing their response capacity to be suppressed by nitric oxide (NO). The results obtained by these researchers corroborate with several studies demonstrating that the chemokines released by MSC predominantly possess chemotactic activity, playing a direct role in the immunomodulation process (Ren et al., 2008).

IL-6 and IL-8 were secreted at a low abundance. IL-6 is a multifunctional cytokine with important role in the regulation of immune response and homeostasis process. In addition, IL-6 is involved in tissue remodeling, acting on connective tissue cells, proteases and protease inhibitors (Liu and Hwang, 2005). Park et al. (2009) evaluated 120 cytokines secreted by MSC at mRNA levels and demonstrated that IL-6 had the highest expression amongst all cytokines, leading to the conclusion that IL-6 is the main cytokine responsible for the immunoregulatory effects of MSC. Similarly, IL-8 (CXCL8) was the first chemokine described with pro-angiogenic properties, confirmed in several tumor types (Hou et al., 2014). A study headed by Wang et al. (2015) clearly demonstrated that MSC promoted tumor growth (colorectal cancer) by increasing angiogenesis by mechanisms dependent on IL-8 secretion in animal models. They also suggested that the disruption of tumor-stroma interactions and suppression of IL-8 secretion by MSC may represent an original approach for the treatment of colorectal cancer (Wang et al., 2015). In a similar study, researchers reported that IL-8 may potentiate tumor progression through the induction of the Epithelial-Mesenchymal Transition (EMT) process (Fernando et al., 2011).

With the increase of clinical trials using MSC secretome-derived products, an important key factor is the development of a reproducible, scalable, and well-controlled platform. The use of bioreactor meets these criteria, improving the predictability in the composition and function of secretome-derived products. We evaluated the secretion pattern of the selected proteins in the bioreactor-supernatant during 96, 120, 144, and 168 h of culture. We observed that the majority of proteins were enriched during the time, being the last day of culture the ideal time for supernatant collection. The average of maximum protein productivity was about 2.0 ng/mL/day, representing a total production of micrograms in the 800 mL-bioreactor vessel at 168 h of culture.

Corroborating with static results, proteins such as collagen, SPARC, TGFB1, TIMP-1, TIMP-2, MMP-14 were secreted at higher rates. Interestingly, certain chemokines (CCL2 and CXCL1) and growth factors (IGFBP-2,−4, and−6) were released with high abundance. It is well-known that the culture system used to expand MSC can modulate and impact the composition and bioactivity of the MSC secretome (Teixeira et al., 2015). For example, Teixeira et al. (2016) showed that culturing MSC using the same bioreactor used in this work enhanced the neuroregulatory profile of the secretome, compared with cells grown in static culture plates.

Of note, the rate of secreted proteins was slightly different between the donors, especially for the donor 3. It is well-known that inherent variability exists related to factors such as the age and health of the individual (Mendicino et al., 2014). Independently of that observation, the overall secretome was considered similar in standardized growth conditions, producing the same set proteins.

It is worth mentioning that FBS was used as culture medium supplementation, being necessary to perform an extra step of albumin depletion. FBS presents several additional drawbacks, such as batch-to-batch variability, risks of infection, high amount of proteins, compromising MSC use for therapeutic settings (Chase et al., 2010; Gottipamula et al., 2014). Thus, isolating/expanding MSC in a chemically defined culture medium is mandatory for the translation of secretome-derived products to the clinic (Phelps et al., 2018) and future experiments need to be performed.

## Conclusions

In conclusion, proteins secreted by MSC are involved in important biological processes and may represent a promising alternative to cell-based therapy. Global proteomic studies of MSC contribute significantly to the understanding of the regeneration processes and the characterization of these proteins will certainly expand the clinical applications of MSC. In this work, the analysis of the conditioned medium of MSC culture (secretome) showed the secretion of important proteins involved in different cellular processes, such as immunomodulatory activities, cell differentiation, tissue remodeling, angiogenesis and hematopoiesis. Also, MRM analysis allowed for monitoring the rate of accumulation of selected proteins in the bioreactor supernatant, representing an important analytical platform to guarantee reproducibility and robustness of bioreactor cell production. Finally and most importantly, relevant proteins monitored are secreted and concentrated in the bioreactor supernatant at an average rate of 2.0 ng/mL/day, representing a safe and efficient method of producing cell-free therapeutic products.

## Author Contributions

AM carried out the experiments of MSC expansion, proteomics, and wrote the manuscript. CT and GF prepared the samples for proteomic analysis. GF and GL analyzed the samples in the mass spectrometry. DC, KS, and VF supervised the project. SP designed and developed proteomic experiments. VF helped to design the experiments. All authors discussed the results and contributed to the final manuscript.

### Conflict of Interest Statement

The authors declare that the research was conducted in the absence of any commercial or financial relationships that could be construed as a potential conflict of interest.

## Supplementary Material

Supplementary Figure 1 Representative chromatogram for each representative peptide evaluated using Multiple Reaction Monitoring (MRM) methodology.

Supplementary Figure 2 Growth profile of UCM MSC cultured in stirred-tank bioreactor for three independent donors (*n* = 3).

Supplementary Table 1 Global list of protein identified after mass spectrometry analysis of UCM MSC cultured under static conditions (*n* = 3, three independent donors).

Supplementary Table 2 Multiple Reaction Monitoring (MRM) parameters for quantification of target proteins in cell supernatants.
