# Supplementary figures and images for: Proteomic Identification and Time-Course Monitoring of Secreted Proteins During Expansion of Human Mesenchymal Stem/Stromal in Stirred-Tank Bioreactor
Source: Front Bioeng Biotechnol. 2019 Jun 26;7:154. doi: 10.3389/fbioe.2019.00154 (PMC6607109; doi:10.3389/fbioe.2019.00154)

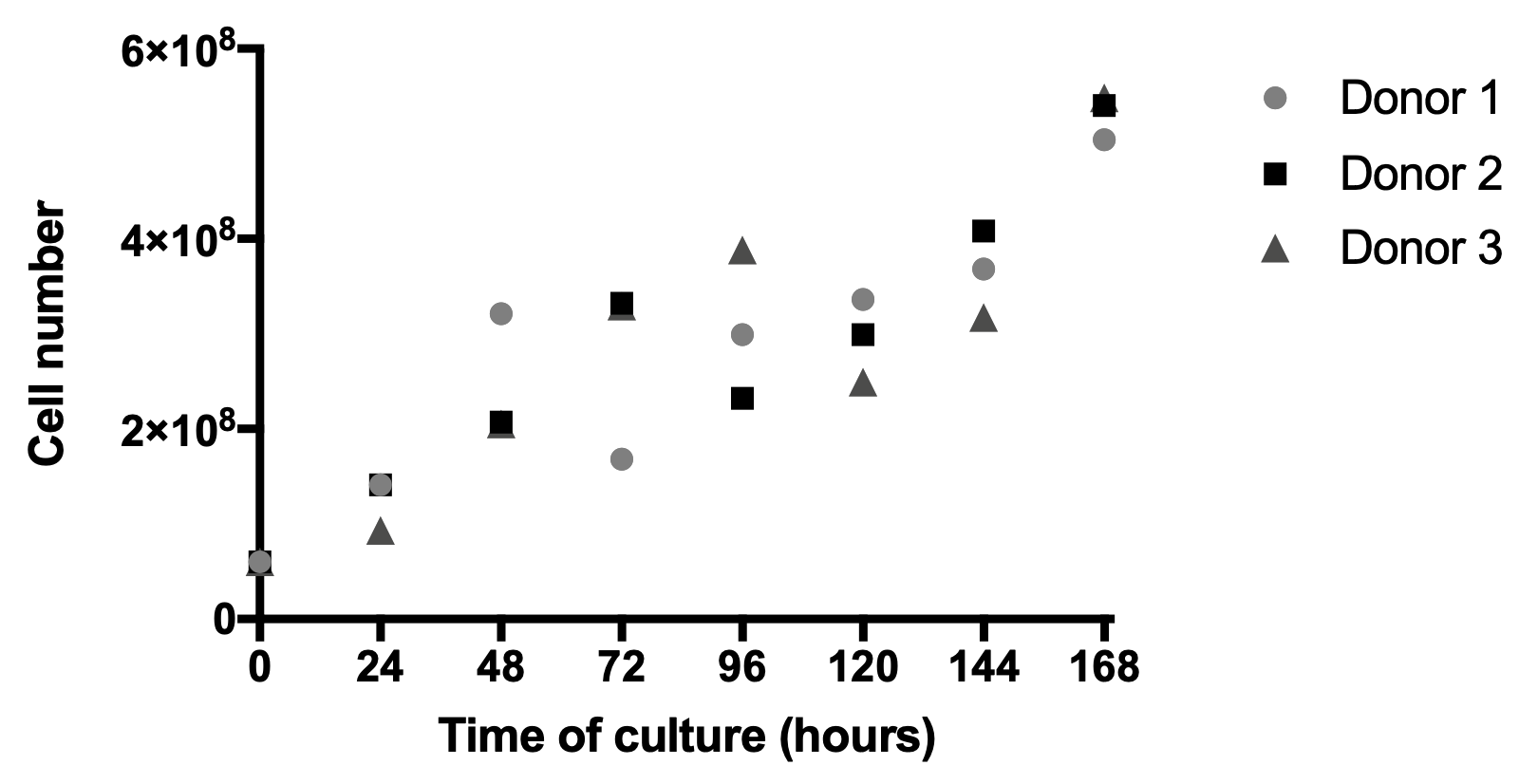

Supplement: Supplementary Figure 1 — Representative chromatogram for each representative peptide evaluated using Multiple Reaction Monitoring (MRM) methodology. [file Image_1.TIFF]
