# Supplementary material for: Proteomic Identification and Time-Course Monitoring of Secreted Proteins During Expansion of Human Mesenchymal Stem/Stromal in Stirred-Tank Bioreactor
Source: Front Bioeng Biotechnol. 2019 Jun 26;7:154. doi: 10.3389/fbioe.2019.00154 (PMC6607109; doi:10.3389/fbioe.2019.00154)

CCL2 MCP1 SCYA2 -EI**C**ADPK (416,9768++)

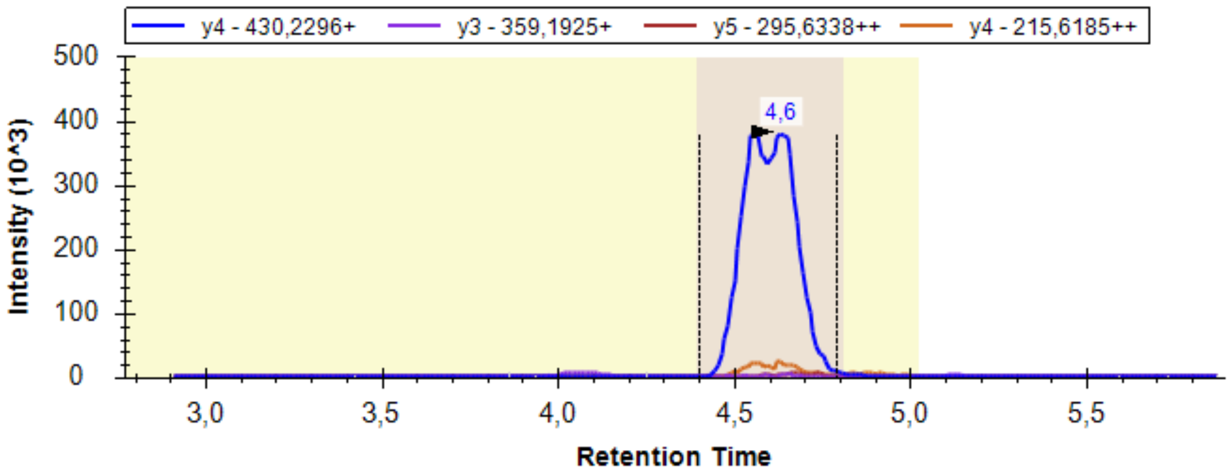

COL1A2 -GFPGTPGLPGFK (588,1854++)

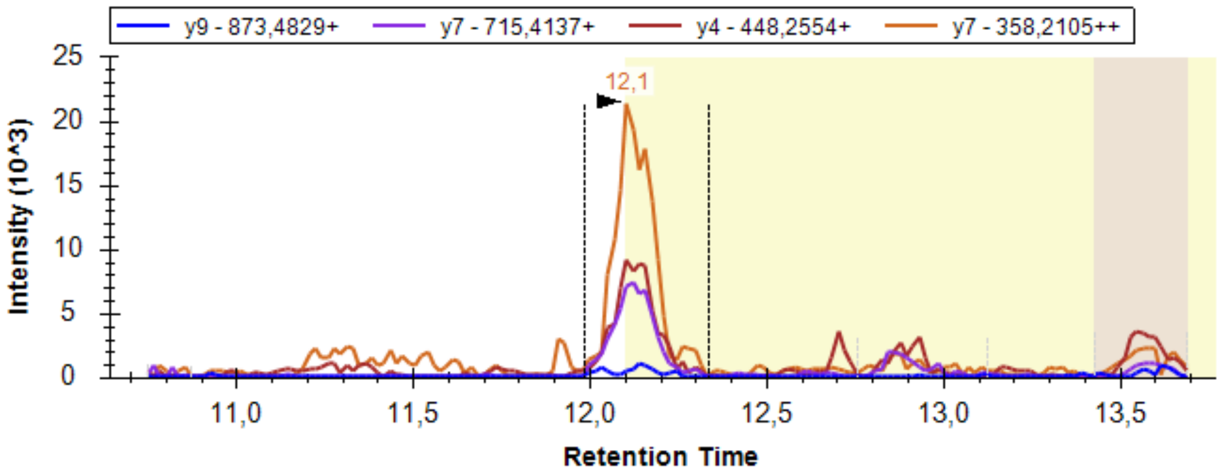

COL1A2 –GPAGPQGPR(418,9637++)

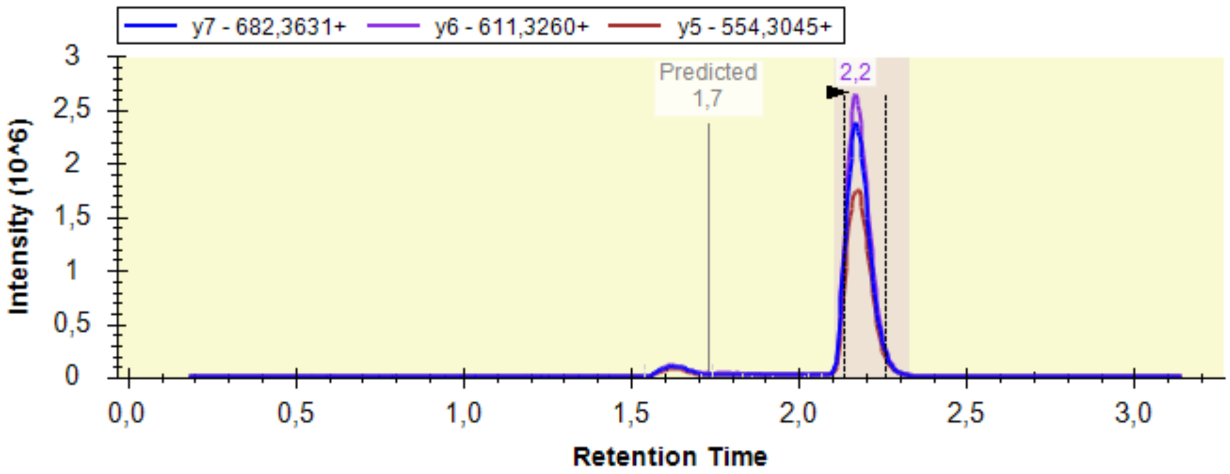

COL3A1-DGSPGEPGANGLPGAAGER (855,3830+
+)

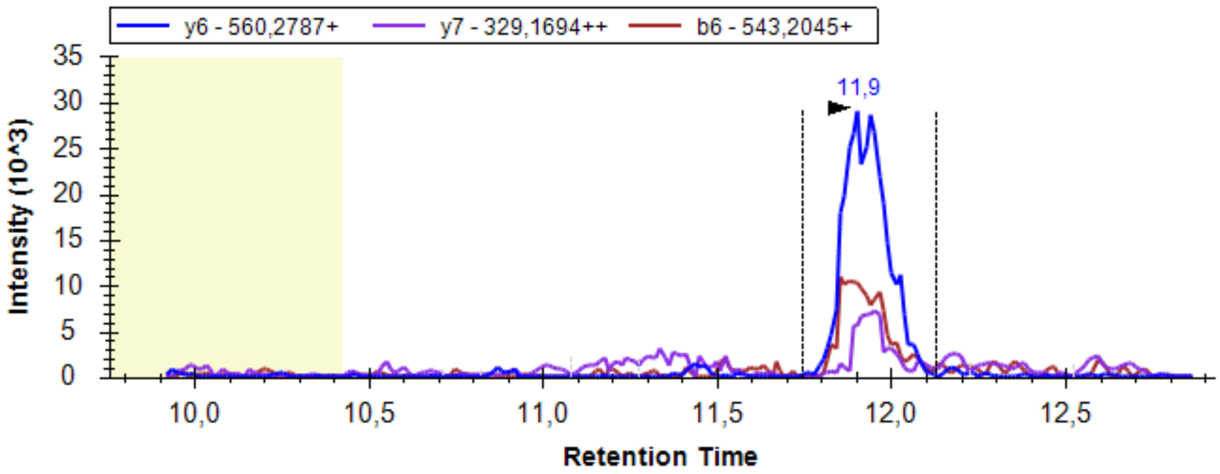

CXCL1-NIQSVNVK (451,5197++)

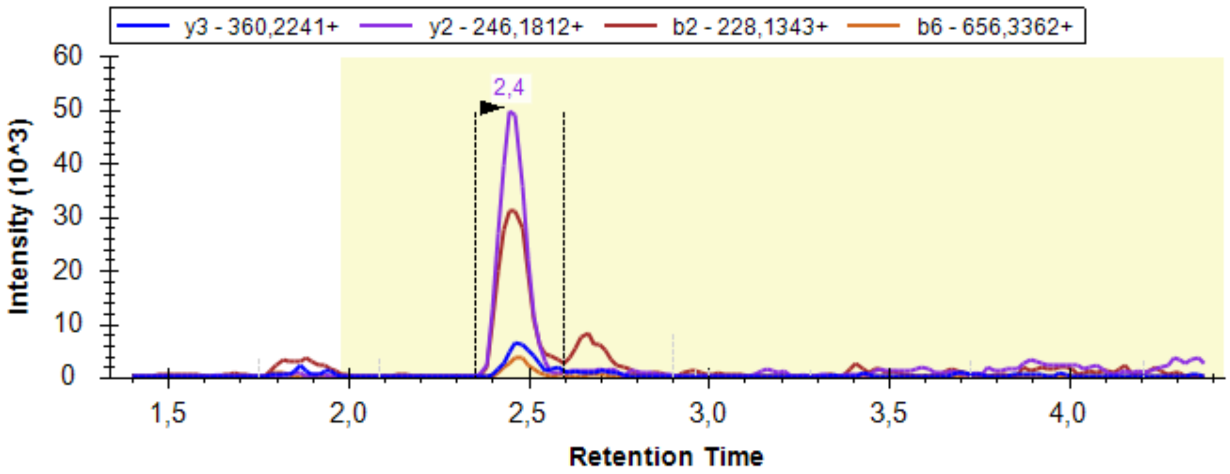

IGFBP2-TP**C**QQELDQVLER (539,5984+++)

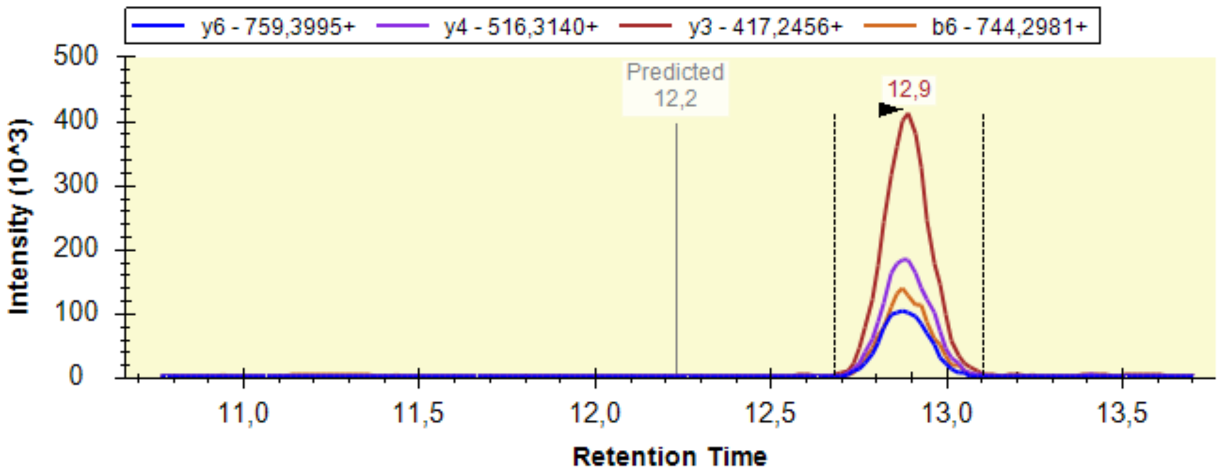

IGFBP3 -AYLLPAPPAPGNASESEEDR (695,7450+++)

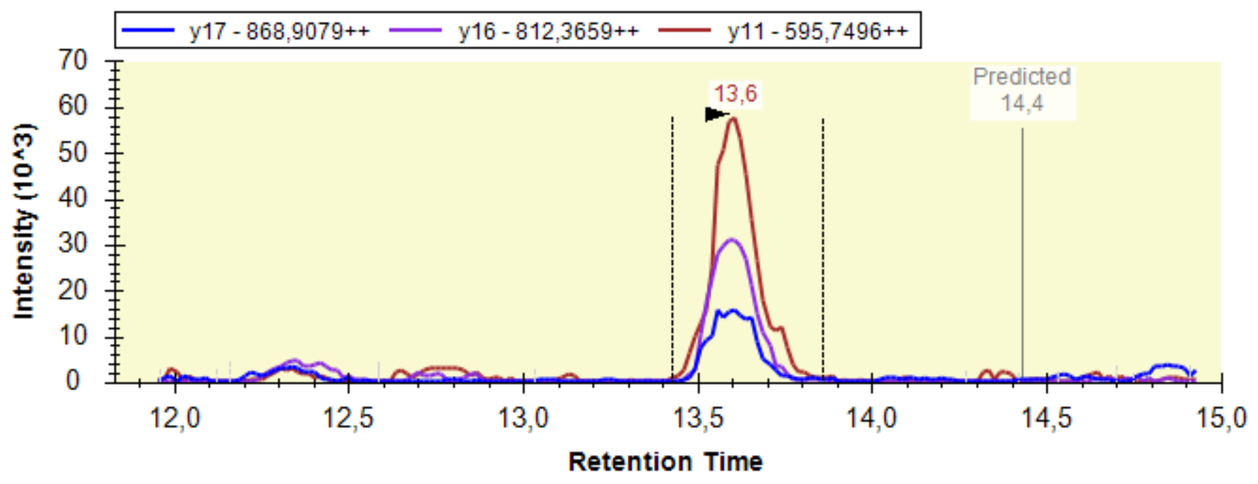

IGFBP4 - LPGGLEPK (405,9850++)

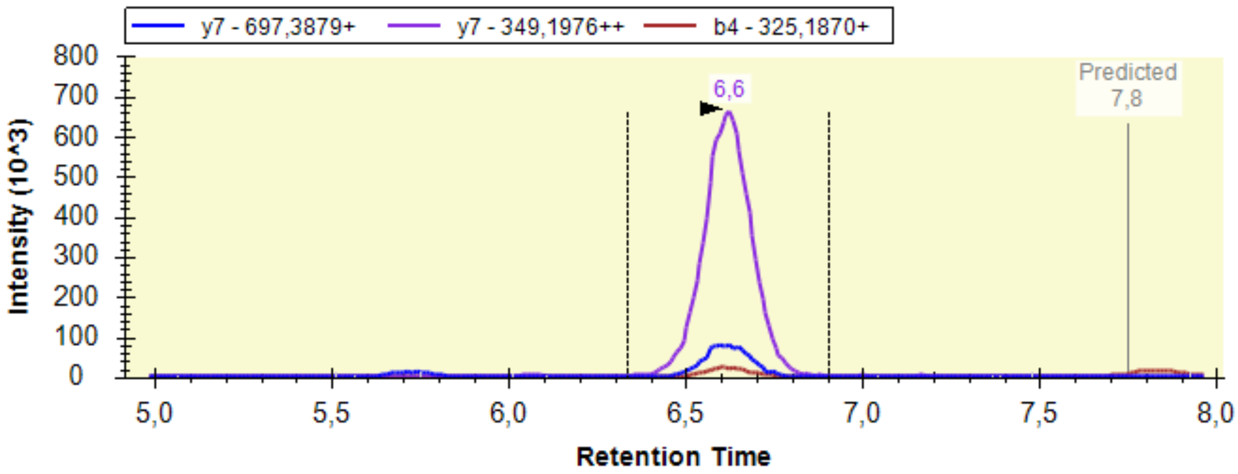

IGFBP5 - FVGGAENTAHPR (419,4564+++)

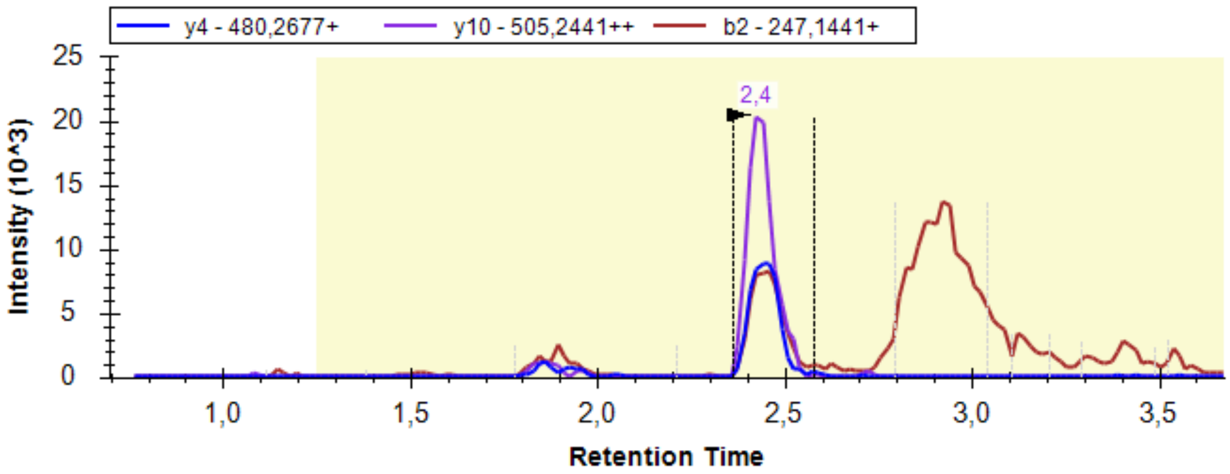

IGFBP6 - HLDSVLQQLQTEVYR (610,6842+++)

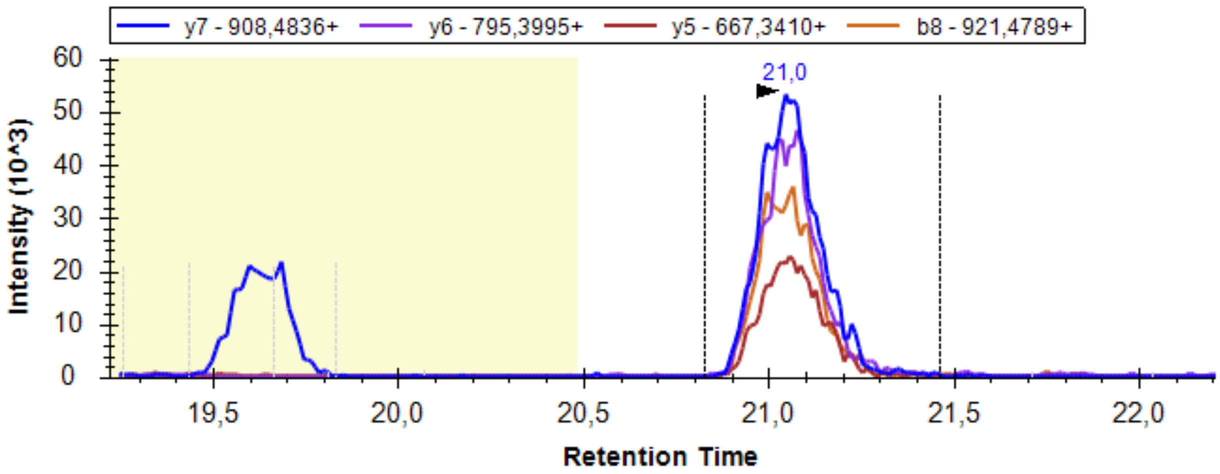

IBP7 - AGAAAGGPGVSGV**C**V**C**K (759,8768++)

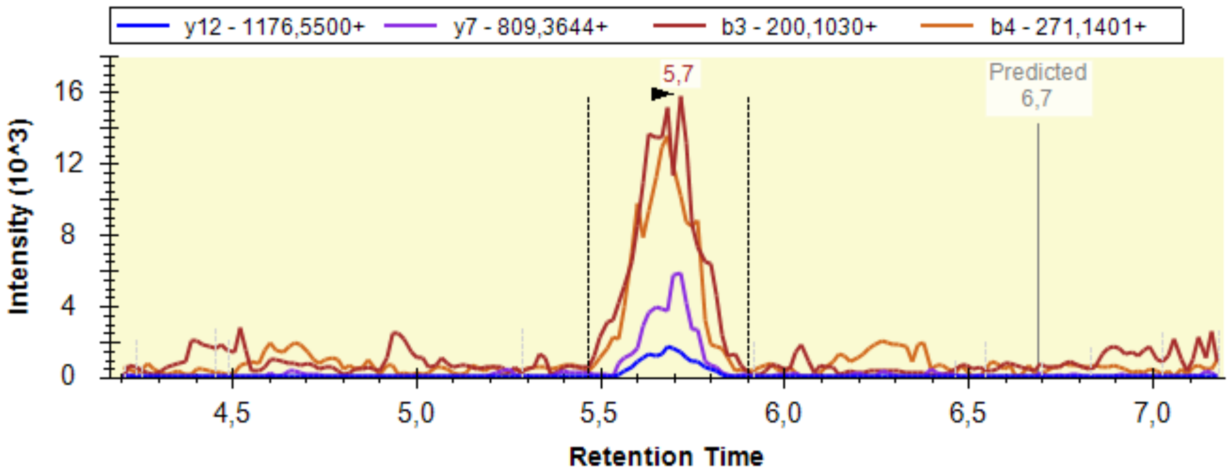

IL6 - NLDAITTPDPTTNASLLTK (663,0719+++)

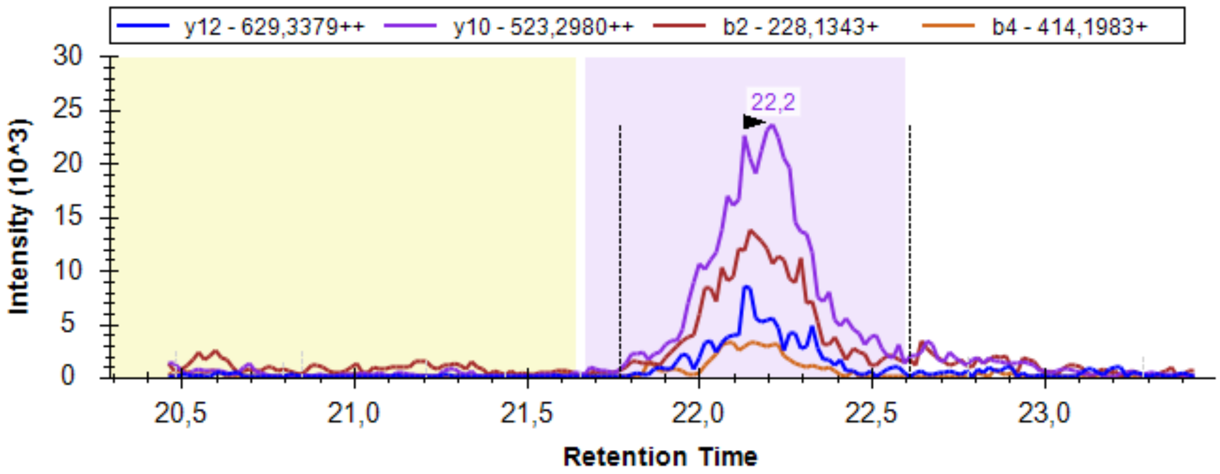

MMP1 – AFQLWSNVTPLTFTK (877,5163++)

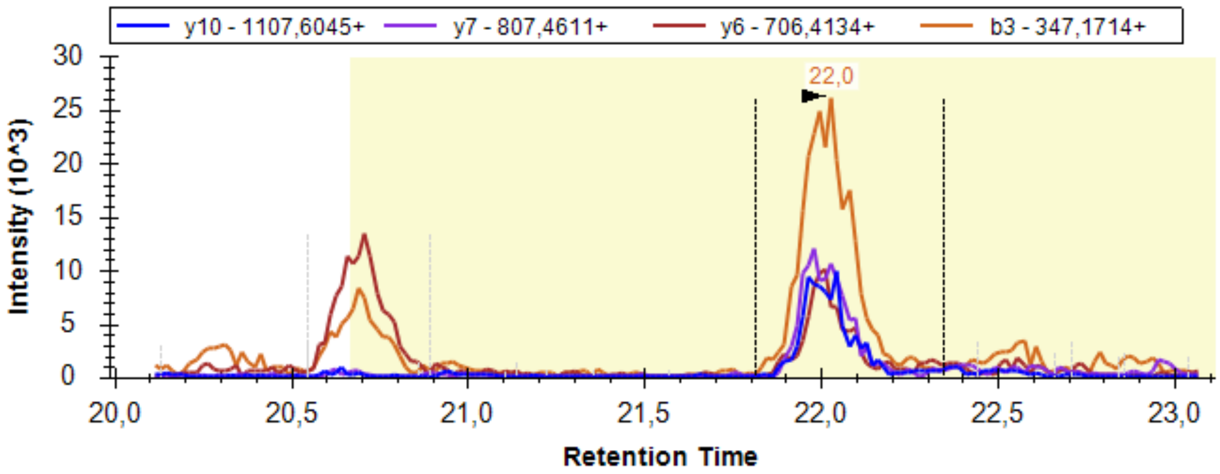

MMP14 - FYGLQVTGK (507,0904++)

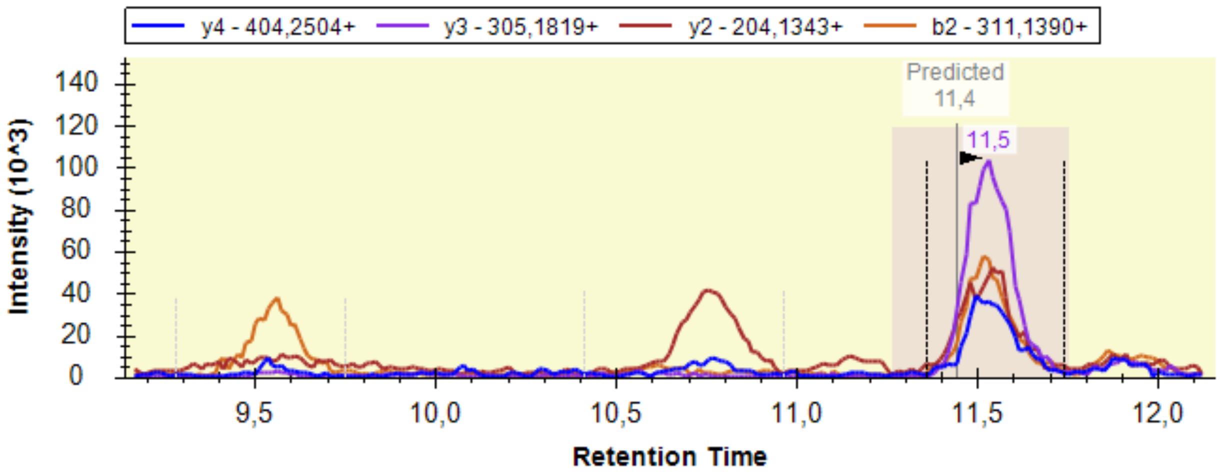

SPARC ON - LEAGDHPVELLAR (474,2043+++)

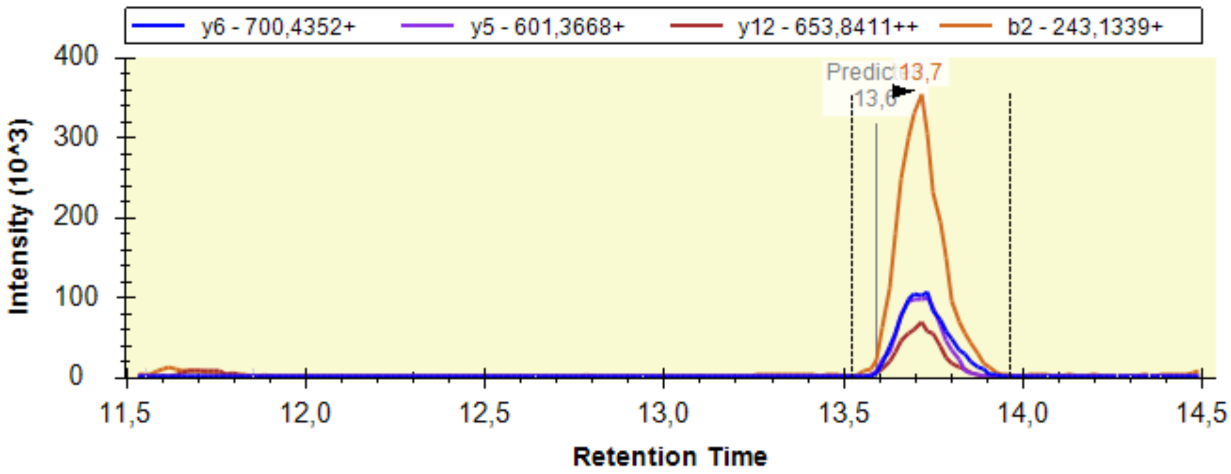

TGFBI - LTLLAPLNSVFK (658,8120++)

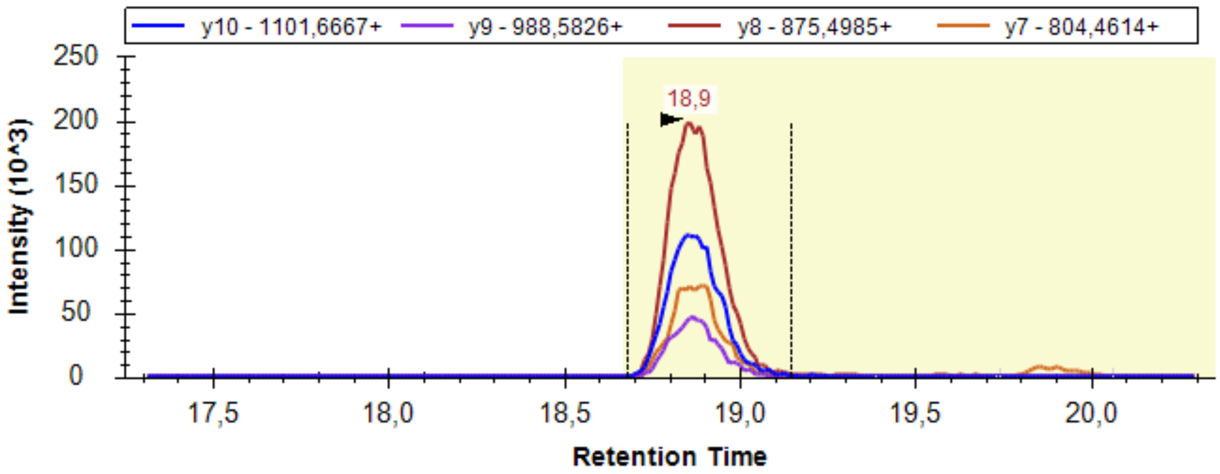

THBS1 -FTGSQPFGQGVEHATANK (626,3416+++)

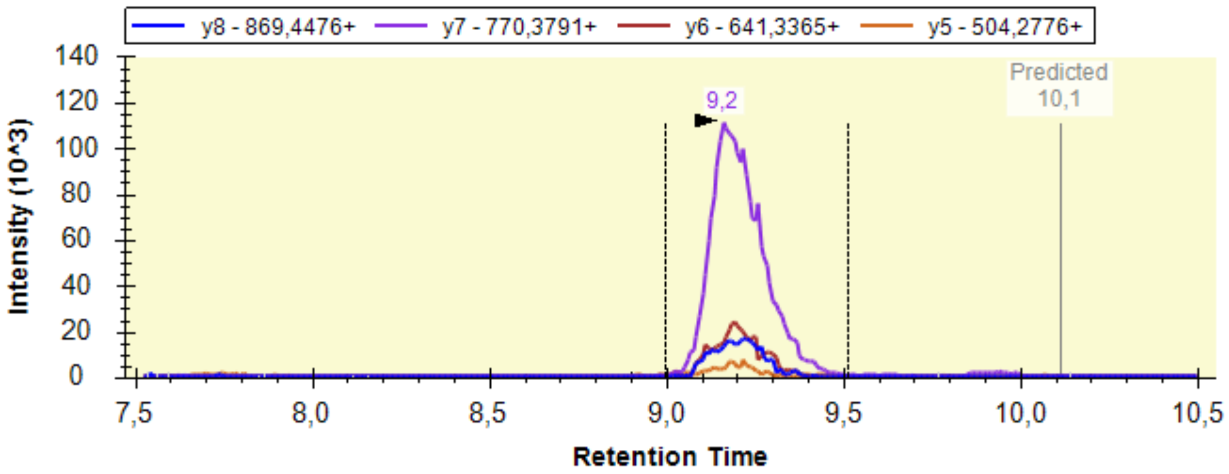

TIMP1 - GFQALGDAADIR (617,6765++)

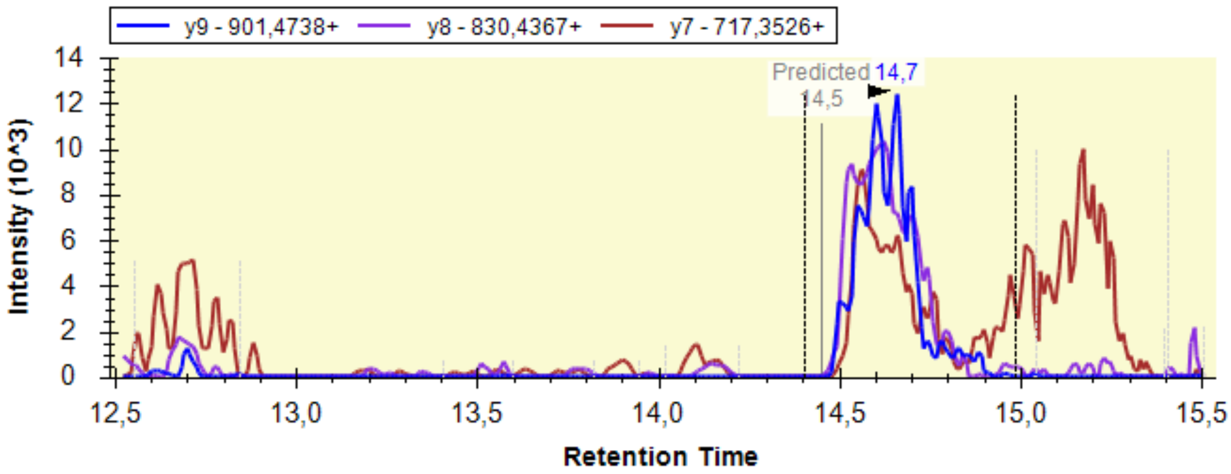

TIMP2 - QEFLDIEDP (553,5867++)

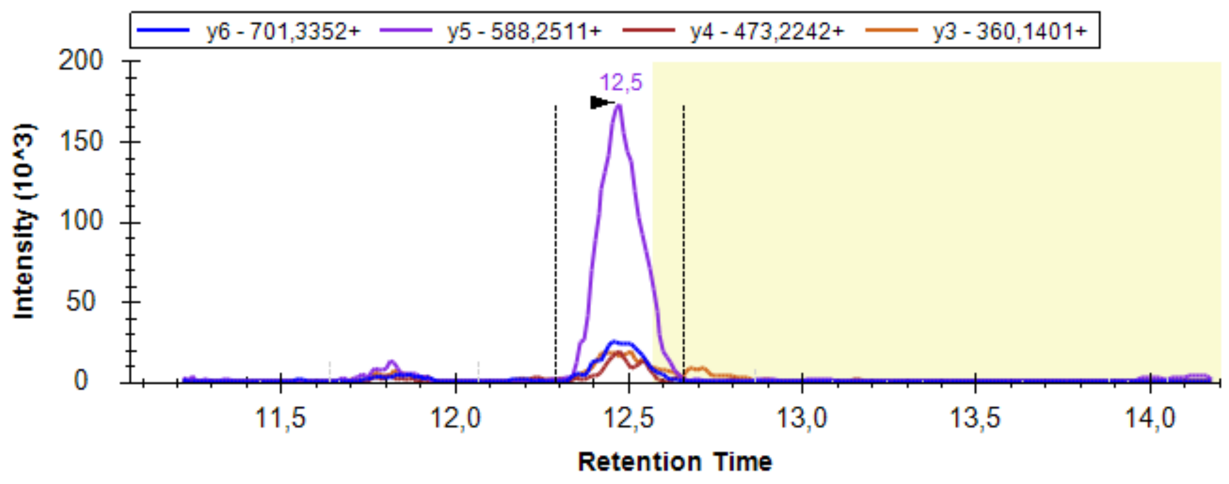

Supplement: Supplementary Figure 2 — Growth profile of UCM MSC cultured in stirred-tank bioreactor for three independent donors (n = 3). [file Data_Sheet_2.PDF]
