# Supplementary material for: Proteomic Identification and Time-Course Monitoring of Secreted Proteins During Expansion of Human Mesenchymal Stem/Stromal in Stirred-Tank Bioreactor
Source: Front Bioeng Biotechnol. 2019 Jun 26;7:154. doi: 10.3389/fbioe.2019.00154 (PMC6607109; doi:10.3389/fbioe.2019.00154)
Supplement: Supplementary Table 1 — Global list of protein identified after mass spectrometry analysis of UCM MSC cultured under static conditions (n = 3, three independent donors). [file Data_Sheet_1.PDF]

Protein	ID	Mass	Best Gene Name	Description	Num Runs	Donor#1 Total Filtered Peptides	Donor#1 Total Filtered Peptides	Donor#2 Total Filtered Peptides	Donor#2 Total Filtered Peptides	Donor#3 Total Filtered Peptides	Donor#3 Total Filtered Peptides
P02751-3	FINC_HUMAN	259216	FN1	Isoform 3 of Fibronectin OS=Homo sapiens GN=FN1	6	2476	2530	720	617	1284	1535
P02452	CO1A1_HUMAN	138941	COL1A1	Collagen alpha-1(I) chain OS=Homo sapiens GN=COL1A1 PE=1 SV=5	6	1695	1780	610	605	1548	2116
P08123	CO1A2_HUMAN	129314	COL1A2	Collagen alpha-2(I) chain OS=Homo sapiens GN=COL1A2 PE=1 SV=7	6	1650	1660	551	598	1310	2182
P05121	PAI1_HUMAN	45060	SERPINE1	Plasminogen activator inhibitor 1 OS=Homo sapiens GN=SERPINE1 PE=1 SV=1	6	641	493	233	218	340	986
P09486	SPRC_HUMAN	34632	SPARC	SPARC OS=Homo sapiens GN=SPARC PE=1 SV=1	6	496	501	235	254	441	953
P02461	CO3A1_HUMAN	138564	COL3A1	Collagen alpha-1(III) chain OS=Homo sapiens GN=COL3A1 PE=1 SV=4	6	536	569	191	184	524	637
P02768	ALBU_HUMAN	69367	ALB	Serum albumin OS=Homo sapiens GN=ALB PE=1 SV=2	6	342	355	625	458	320	401
P04264	K2C1_HUMAN	66039	KRT1	Keratin, type II cytoskeletal 1 OS=Homo sapiens GN=KRT1 PE=1 SV=6	6	406	539	405	397	270	326
P20908	CO5A1_HUMAN	183560	COL5A1	Collagen alpha-1(V) chain OS=Homo sapiens GN=COL5A1 PE=1 SV=3	6	393	401	78	84	527	713
P07996	TSP1_HUMAN	129383	THBS1	Thrombospondin-1 OS=Homo sapiens GN=THBS1 PE=1 SV=2	6	406	433	104	119	498	582
P35908	K22E_HUMAN	65433	KRT2	Keratin, type II cytoskeletal 2 epidermal OS=Homo sapiens GN=KRT2 PE=1 SV=2	6	293	496	427	341	249	318
P35527	K1C9_HUMAN	62064	KRT9	Keratin, type I cytoskeletal 9 OS=Homo sapiens GN=KRT9 PE=1 SV=3	6	340	433	416	398	211	236
P21333-2	FLNA_HUMAN	280018	FLNA	Isoform 2 of Filamin-A OS=Homo sapiens GN=FLNA	6	311	345	296	264	298	360
P13645	K1C10_HUMAN	58827	KRT10	Keratin, type I cytoskeletal 10 OS=Homo sapiens GN=KRT10 PE=1 SV=6	6	297	433	353	303	211	275
P63261	ACTG_HUMAN	41793	ACTG1	Actin, cytoplasmic 2 OS=Homo sapiens GN=ACTG1 PE=1 SV=1	6	329	332	245	254	248	426
P60709	ACTB_HUMAN	41737	ACTB	Actin, cytoplasmic 1 OS=Homo sapiens GN=ACTB PE=1 SV=1	6	328	333	241	253	248	429
P21810	PGS1_HUMAN	41654	BGN	Biglycan OS=Homo sapiens GN=BGN PE=1 SV=2	6	460	499	88	81	307	354
P51884	LUM_HUMAN	38429	LUM	Lumican OS=Homo sapiens GN=LUM PE=1 SV=2	6	249	306	106	100	413	527
P12111	CO6A3_HUMAN	343669	COL6A3	Collagen alpha-3(VI) chain OS=Homo sapiens GN=COL6A3 PE=1 SV=5	6	390	460	162	131	265	274
P08253	MMP2_HUMAN	73882	MMP2	72 kDa type IV collagenase OS=Homo sapiens GN=MMP2 PE=1 SV=2	6	271	274	201	222	338	291
P12109	CO6A1_HUMAN	108529	COL6A1	Collagen alpha-1(VI) chain OS=Homo sapiens GN=COL6A1 PE=1 SV=3	6	292	305	137	131	269	286
O00391	QSOX1_HUMAN	82578	QSOX1	Sulfhydryl oxidase 1 OS=Homo sapiens GN=QSOX1 PE=1 SV=3	6	286	340	107	114	216	253
Q16270-2	IBP7_HUMAN	28860	IGFBP7	Isoform 2 of Insulin-like growth factor-binding protein 7 OS=Homo sapiens GN=IGFBP7	6	267	271	129	145	193	260
P24593	IBP5_HUMAN	30570	IGFBP5	Insulin-like growth factor-binding protein 5 OS=Homo sapiens GN=IGFBP5 PE=1 SV=1	6	305	279	68	90	220	291
P08670	VIME_HUMAN	53652	VIM	Vimentin OS=Homo sapiens GN=VIM PE=1 SV=4	6	273	258	99	110	203	263
P01024	CO3_HUMAN	187148	C3	Complement C3 OS=Homo sapiens GN=C3 PE=1 SV=2	6	280	296	73	66	222	242
P98160	PGBM_HUMAN	468830	HSPG2	Basement membrane-specific heparan sulfate proteoglycan core protein OS=Homo sapiens GN=HSPG2 PE=1 SV=4	6	287	329	89	82	164	221
Q99715	COCA1_HUMAN	333147	COL12A1	Collagen alpha-1(XII) chain OS=Homo sapiens GN=COL12A1 PE=1 SV=2	6	245	259	114	94	190	225
P01033	TIMP1_HUMAN	23171	TIMP1	Metalloproteinase inhibitor 1 OS=Homo sapiens GN=TIMP1 PE=1 SV=1	6	281	272	89	113	177	191
P06733	ENOA_HUMAN	47169	ENO1	Alpha-enolase OS=Homo sapiens GN=ENO1 PE=1 SV=2	6	147	116	205	201	153	298
Q15582	BGH3_HUMAN	74681	TGFBI	Transforming growth factor-beta-induced protein ig-h3 OS=Homo sapiens GN=TGFBI PE=1 SV=1	6	222	217	81	85	253	249
P68032	ACTC_HUMAN	42019	ACTC1	Actin, alpha cardiac muscle 1 OS=Homo sapiens GN=ACTC1 PE=1 SV=1	6	229	221	108	119	138	283
P68133	ACTS_HUMAN	42051	ACTA1	Actin, alpha skeletal muscle OS=Homo sapiens GN=ACTA1 PE=1 SV=1	6	226	220	107	119	137	280
		24409		sp|P00761|TRYP_PIG Trypsin precursor (EC 3.4.21.4) - Sus scrofa (Pig).	6	258	240	131	123	101	123
P12814	ACTN1_HUMAN	103058	ACTN1	Alpha-actinin-1 OS=Homo sapiens GN=ACTN1 PE=1 SV=2	6	140	176	183	193	137	132
P13611	CSPG2_HUMAN	372820	VCAN	Versican core protein OS=Homo sapiens GN=VCAN PE=1 SV=3	6	340	329	16	12	98	159
P62736	ACTA_HUMAN	42009	ACTA2	Actin, aortic smooth muscle OS=Homo sapiens GN=ACTA2 PE=1 SV=1	6	191	193	104	109	114	231
P63267	ACTH_HUMAN	41877	ACTG2	Actin, gamma-enteric smooth muscle OS=Homo sapiens GN=ACTG2 PE=1 SV=1	6	188	189	103	108	110	226
Q12841	FSTL1_HUMAN	34986	FSTL1	Follistatin-related protein 1 OS=Homo sapiens GN=FSTL1 PE=1 SV=1	6	179	173	61	58	147	304
P08476	INHBA_HUMAN	47442	INHBA	Inhibin beta A chain OS=Homo sapiens GN=INHBA PE=1 SV=2	6	249	230	54	49	111	211
O94985	CSTN1_HUMAN	109793	CLSTN1	Calsyntenin-1 OS=Homo sapiens GN=CLSTN1 PE=1 SV=1	6	176	252	61	48	128	165
O43707	ACTN4_HUMAN	104854	ACTN4	Alpha-actinin-4 OS=Homo sapiens GN=ACTN4 PE=1 SV=2	6	110	143	164	158	116	100
P19022	CADH2_HUMAN	99809	CDH2	Cadherin-2 OS=Homo sapiens GN=CDH2 PE=1 SV=4	6	171	193	62	61	145	153
P22692	IBP4_HUMAN	27934	IGFBP4	Insulin-like growth factor-binding protein 4 OS=Homo sapiens GN=IGFBP4 PE=1 SV=2	6	173	164	52	72	134	168

P35442	TSP2_HUMAN	129991	THBS2	Thrombospondin-2 OS=Homo sapiens GN=THBS2 PE=1 SV=2	6	103	151	75	71	159	175
P07585	PGS2_HUMAN	39747	DCN	Decorin OS=Homo sapiens GN=DCN PE=1 SV=1	6	98	100	44	45	220	225
P16035	TIMP2_HUMAN	24399	TIMP2	Metalloproteinase inhibitor 2 OS=Homo sapiens GN=TIMP2 PE=1 SV=2	6	111	123	124	149	118	98
O75369-2	FLNB_HUMAN	275668	FLNB	Isoform 2 of Filamin-B OS=Homo sapiens GN=FLNB	6	102	107	143	133	112	113
Q01995	TAGL_HUMAN	22611	TAGLN	Transgelin OS=Homo sapiens GN=TAGLN PE=1 SV=4	6	158	157	74	72	125	119
P00736	C1R_HUMAN	80119	C1R	Complement C1r subcomponent OS=Homo sapiens GN=C1R PE=1 SV=2	6	107	107	63	61	152	199
P35555	FBN1_HUMAN	312237	FBN1	Fibrillin-1 OS=Homo sapiens GN=FBN1 PE=1 SV=3	6	150	166	55	52	128	136
Q6S8J3	POTEE_HUMAN	121363	POTEE	POTE ankyrin domain family member E OS=Homo sapiens GN=POTEE PE=1 SV=3	6	116	120	89	92	95	157
P01023	A2MG_HUMAN	163291	A2M	Alpha-2-macroglobulin OS=Homo sapiens GN=A2M PE=1 SV=3	6	70	82	222	133	71	82
Q08380	LG3BP_HUMAN	65331	LGALS3BP	Galectin-3-binding protein OS=Homo sapiens GN=LGALS3BP PE=1 SV=1	6	140	160	70	57	102	131
P07737	PROF1_HUMAN	15054	PFN1	Profilin-1 OS=Homo sapiens GN=PFN1 PE=1 SV=2	6	131	107	86	96	108	122
Q08629	TICN1_HUMAN	49124	SPOCK1	Testican-1 OS=Homo sapiens GN=SPOCK1 PE=1 SV=1	6	158	190	44	34	97	119
P10909-2	CLUS_HUMAN	57833	CLU	Isoform 2 of Clusterin OS=Homo sapiens GN=CLU	6	105	113	43	40	122	210
P05997	CO5A2_HUMAN	144910	COL5A2	Collagen alpha-2(V) chain OS=Homo sapiens GN=COL5A2 PE=1 SV=3	6	99	117	40	37	117	222
P02765	FETUA_HUMAN	39325	AHSG	Alpha-2-HS-glycoprotein OS=Homo sapiens GN=AHSG PE=1 SV=1	6	103	100	104	110	78	123
P09871	C1S_HUMAN	76684	C1S	Complement C1s subcomponent OS=Homo sapiens GN=C1S PE=1 SV=1	6	79	107	70	71	137	151
A5A3E0	POTEF_HUMAN	121445	POTEF	POTE ankyrin domain family member F OS=Homo sapiens GN=POTEF PE=1 SV=2	6	110	115	64	68	82	147
P13647	K2C5_HUMAN	62378	KRT5	Keratin, type II cytoskeletal 5 OS=Homo sapiens GN=KRT5 PE=1 SV=3	6	86	115	113	133	51	79
P09382	LEG1_HUMAN	14716	LGALS1	Galectin-1 OS=Homo sapiens GN=LGALS1 PE=1 SV=2	6	142	114	56	59	95	98
P04259	K2C6B_HUMAN	60067	KRT6B	Keratin, type II cytoskeletal 6B OS=Homo sapiens GN=KRT6B PE=1 SV=5	6	81	100	102	141	52	82
P26038	MOES_HUMAN	67820	MSN	Moesin OS=Homo sapiens GN=MSN PE=1 SV=3	6	117	94	95	98	76	63
P62258	1433E_HUMAN	29174	YWHAE	14-3-3 protein epsilon OS=Homo sapiens GN=YWHAE PE=1 SV=1	6	121	126	74	83	62	77
Q14315	FLNC_HUMAN	291022	FLNC	Filamin-C OS=Homo sapiens GN=FLNC PE=1 SV=3	6	74	75	143	125	68	57
Q02818	NUCB1_HUMAN	53879	NUCB1	Nucleobindin-1 OS=Homo sapiens GN=NUCB1 PE=1 SV=4	6	130	124	41	39	80	116
P18206-2	VINC_HUMAN	116722	VCL	Isoform 1 of Vinculin OS=Homo sapiens GN=VCL	6	94	116	93	92	70	58
P60174-1	TPIS_HUMAN	26669	TPI1	Isoform 2 of Triosephosphate isomerase OS=Homo sapiens GN=TPI1	6	100	105	78	93	66	77
P26022	PTX3_HUMAN	41976	PTX3	Pentraxin-related protein PTX3 OS=Homo sapiens GN=PTX3 PE=1 SV=3	6	78	73	91	99	65	105
Q9BUD6	SPON2_HUMAN	35846	SPON2	Spondin-2 OS=Homo sapiens GN=SPON2 PE=1 SV=3	6	98	99	34	36	77	167
P04075	ALDOA_HUMAN	39420	ALDOA	Fructose-bisphosphate aldolase A OS=Homo sapiens GN=ALDOA PE=1 SV=2	6	79	64	100	92	61	111
P07858	CATB_HUMAN	37822	CTSB	Cathepsin B OS=Homo sapiens GN=CTSB PE=1 SV=3	6	80	40	54	43	85	188
P20742	PZP_HUMAN	163863	PZP	Pregnancy zone protein OS=Homo sapiens GN=PZP PE=1 SV=4	6	48	59	171	99	52	56
Q9BYX7	ACTBM_HUMAN	42016	POTEKP	Putative beta-actin-like protein 3 OS=Homo sapiens GN=POTEKP PE=5 SV=1	6	91	94	52	58	70	116
P07093-2	GDN_HUMAN	44000	SERPINE2	Isoform 2 of Glia-derived nexin OS=Homo sapiens GN=SERPINE2	6	89	56	28	24	68	204
P07437	TBB5_HUMAN	49671	TUBB	Tubulin beta chain OS=Homo sapiens GN=TUBB PE=1 SV=2	6	70	56	65	66	86	121
P12107-2	COBA1_HUMAN	182422	COL11A1	Isoform B of Collagen alpha-1(XI) chain OS=Homo sapiens GN=COL11A1	6	91	77	11	8	113	162
P62937	PPIA_HUMAN	18012	PPIA	Peptidyl-prolyl cis-trans isomerase A OS=Homo sapiens GN=PPIA PE=1 SV=2	6	86	74	47	67	86	99
Q86YZ3	HORN_HUMAN	282390	HRNR	Hornerin OS=Homo sapiens GN=HRNR PE=1 SV=2	6	82	80	80	69	63	83
P68363	TBA1B_HUMAN	50152	TUBA1B	Tubulin alpha-1B chain OS=Homo sapiens GN=TUBA1B PE=1 SV=1	6	74	63	81	69	60	100
P08572	CO4A2_HUMAN	167553	COL4A2	Collagen alpha-2(IV) chain OS=Homo sapiens GN=COL4A2 PE=1 SV=4	6	112	103	19	26	77	107
P30101	PDIA3_HUMAN	56782	PDIA3	Protein disulfide-isomerase A3 OS=Homo sapiens GN=PDIA3 PE=1 SV=4	6	73	75	64	70	52	103
P02538	K2C6A_HUMAN	60045	KRT6A	Keratin, type II cytoskeletal 6A OS=Homo sapiens GN=KRT6A PE=1 SV=3	6	57	78	81	123	35	58
P13639	EF2_HUMAN	95338	EEF2	Elongation factor 2 OS=Homo sapiens GN=EEF2 PE=1 SV=4	6	78	86	58	62	80	68
P02533	K1C14_HUMAN	51561	KRT14	Keratin, type I cytoskeletal 14 OS=Homo sapiens GN=KRT14 PE=1 SV=4	6	66	73	84	115	37	55
P0CG38	POTEI_HUMAN	121282	POTEI	POTE ankyrin domain family member I OS=Homo sapiens GN=POTEI PE=3 SV=1	6	84	81	41	45	59	118
P48668	K2C6C_HUMAN	60025	KRT6C	Keratin, type II cytoskeletal 6C OS=Homo sapiens GN=KRT6C PE=1 SV=3	6	57	78	78	121	36	56
P23142	FBLN1_HUMAN	77214	FBLN1	Fibulin-1 OS=Homo sapiens GN=FBLN1 PE=1 SV=4	6	99	108	63	57	55	43
P14618	KPYM_HUMAN	57937	PKM	Pyruvate kinase PKM OS=Homo sapiens GN=PKM PE=1 SV=4	6	111	101	37	39	66	68
Q71U36-2	TBA1A_HUMAN	46297	TUBA1A	Isoform 2 of Tubulin alpha-1A chain OS=Homo sapiens GN=TUBA1A	6	73	57	76	63	57	92
P68371	TBB4B_HUMAN	49831	TUBB4B	Tubulin beta-4B chain OS=Homo sapiens GN=TUBB4B PE=1 SV=1	6	59	52	62	63	73	107
F5H5D3	F5H5D3_HUMAN	57730	TUBA1C	Tubulin alpha-1C chain OS=Homo sapiens GN=TUBA1C PE=2 SV=1	6	72	57	72	60	55	91
P11021	GRP78_HUMAN	72333	HSPA5	78 kDa glucose-regulated protein OS=Homo sapiens GN=HSPA5 PE=1 SV=2	6	89	85	52	59	60	57

P23528	COF1_HUMAN	18502	CFL1	Cofilin-1 OS=Homo sapiens GN=CFL1 PE=1 SV=3	6	67	61	35	46	82	106
O43852-3	CALU_HUMAN	38051	CALU	Isoform 3 of Calumenin OS=Homo sapiens GN=CALU	6	70	75	43	39	49	117
Q92626	PXDN_HUMAN	165275	PXDN	Peroxidasin homolog OS=Homo sapiens GN=PXDN PE=1 SV=2	6	88	97	18	23	81	86
P08779	K1C16_HUMAN	51268	KRT16	Keratin, type I cytoskeletal 16 OS=Homo sapiens GN=KRT16 PE=1 SV=4	6	52	53	66	151	29	38
Q562R1	ACTBL_HUMAN	42003	ACTBL2	Beta-actin-like protein 2 OS=Homo sapiens GN=ACTBL2 PE=1 SV=2	6	71	82	48	52	53	81
P55287-2	CAD11_HUMAN	76457	CDH11	Isoform 2 of Cadherin-11 OS=Homo sapiens GN=CDH11	6	87	95	19	30	74	80
P61769	B2MG_HUMAN	13715	B2M	Beta-2-microglobulin OS=Homo sapiens GN=B2M PE=1 SV=1	6	88	80	29	40	62	85
P11047	LAMC1_HUMAN	177603	LAMC1	Laminin subunit gamma-1 OS=Homo sapiens GN=LAMC1 PE=1 SV=3	6	61	62	48	40	81	87
Q14112	NID2_HUMAN	151254	NID2	Nidogen-2 OS=Homo sapiens GN=NID2 PE=1 SV=3	6	78	89	26	20	80	83
P36222	CH3L1_HUMAN	42625	CHI3L1	Chitinase-3-like protein 1 OS=Homo sapiens GN=CHI3L1 PE=1 SV=2	6	36	40	18	18	103	157
O75326	SEM7A_HUMAN	74824	SEMA7A	Semaphorin-7A OS=Homo sapiens GN=SEMA7A PE=1 SV=1	6	44	64	54	61	77	69
Q13885	TBB2A_HUMAN	49907	TUBB2A	Tubulin beta-2A chain OS=Homo sapiens GN=TUBB2A PE=1 SV=1	6	59	50	48	49	65	95
Q9BVA1	TBB2B_HUMAN	49953	TUBB2B	Tubulin beta-2B chain OS=Homo sapiens GN=TUBB2B PE=1 SV=1	6	59	50	48	49	65	95
O00468-6	AGRIN_HUMAN	214846	AGRN	Isoform 6 of Agrin OS=Homo sapiens GN=AGRN	6	109	122	29	27	22	48
P01034	CYTC_HUMAN	15799	CST3	Cystatin-C OS=Homo sapiens GN=CST3 PE=1 SV=1	6	87	77	23	27	63	79
P22392-2	NDKB_HUMAN	30137	NME2	Isoform 3 of Nucleoside diphosphate kinase B OS=Homo sapiens GN=NME2	6	73	62	47	63	53	56
Q32Q12	Q32Q12_HUMAN	32642	NME1-NME2	Nucleoside diphosphate kinase OS=Homo sapiens GN=NME1-NME2 PE=2 SV=1	6	73	62	47	63	53	56
P09936	UCHL1_HUMAN	24824	UCHL1	Ubiquitin carboxyl-terminal hydrolase isozyme L1 OS=Homo sapiens GN=UCHL1 PE=1 SV=2	6	68	77	47	59	51	46
Q14766-4	LTBP1_HUMAN	186867	LTBP1	Isoform 4 of Latent-transforming growth factor beta-binding protein 1 OS=Homo sapiens GN=LTBP1	6	67	79	22	19	78	77
Q16610	ECM1_HUMAN	60674	ECM1	Extracellular matrix protein 1 OS=Homo sapiens GN=ECM1 PE=1 SV=2	6	65	66	38	36	66	69
K7EL90	K7EL90_HUMAN	3404	LOC100653515	Protein LOC100653515 (Fragment) OS=Homo sapiens GN=LOC100653515 PE=2 SV=1	6	56	61	50	66	54	49
P04350	TBB4A_HUMAN	49586	TUBB4A	Tubulin beta-4A chain OS=Homo sapiens GN=TUBB4A PE=1 SV=2	6	53	44	52	54	56	72
P0CG39	POTEJ_HUMAN	117390	POTEJ	POTE ankyrin domain family member J OS=Homo sapiens GN=POTEJ PE=3 SV=1	6	66	63	35	37	44	82
P00558	PGK1_HUMAN	44615	PGK1	Phosphoglycerate kinase 1 OS=Homo sapiens GN=PGK1 PE=1 SV=3	6	62	54	48	43	40	79
P13797	PLST_HUMAN	70811	PLS3	Plastin-3 OS=Homo sapiens GN=PLS3 PE=1 SV=4	6	86	67	32	44	49	45
Q13748	TBA3C_HUMAN	49960	TUBA3C	Tubulin alpha-3C/D chain OS=Homo sapiens GN=TUBA3C PE=1 SV=3	6	53	38	64	53	41	71
P67936	TPM4_HUMAN	28522	TPM4	Tropomyosin alpha-4 chain OS=Homo sapiens GN=TPM4 PE=1 SV=3	6	80	74	28	27	45	61
Q9UI42	CBPA4_HUMAN	47351	CPA4	Carboxypeptidase A4 OS=Homo sapiens GN=CPA4 PE=1 SV=2	6	76	68	25	23	37	84
P07195	LDHB_HUMAN	36638	LDHB	L-lactate dehydrogenase B chain OS=Homo sapiens GN=LDHB PE=1 SV=2	6	54	49	51	55	49	53
Q6UVK1	CSPG4_HUMAN	250537	CSPG4	Chondroitin sulfate proteoglycan 4 OS=Homo sapiens GN=CSPG4 PE=1 SV=2	6	32	28	68	61	63	59
P07942	LAMB1_HUMAN	198038	LAMB1	Laminin subunit beta-1 OS=Homo sapiens GN=LAMB1 PE=1 SV=2	6	61	73	30	22	52	72
P68366	TBA4A_HUMAN	49924	TUBA4A	Tubulin alpha-4A chain OS=Homo sapiens GN=TUBA4A PE=1 SV=1	6	43	34	61	54	41	70
Q6PEY2	TBA3E_HUMAN	49859	TUBA3E	Tubulin alpha-3E chain OS=Homo sapiens GN=TUBA3E PE=1 SV=2	6	48	35	58	49	40	69
P11142	HSP7C_HUMAN	70898	HSPA8	Heat shock cognate 71 kDa protein OS=Homo sapiens GN=HSPA8 PE=1 SV=1	6	63	54	44	48	48	39
P23284	PPIB_HUMAN	23743	PPIB	Peptidyl-prolyl cis-trans isomerase B OS=Homo sapiens GN=PPIB PE=1 SV=2	6	64	61	32	36	52	51
Q9BRK5	CAB45_HUMAN	41807	SDF4	45 kDa calcium-binding protein OS=Homo sapiens GN=SDF4 PE=1 SV=1	6	56	55	14	19	41	106
P35579	MYH9_HUMAN	226532	MYH9	Myosin-9 OS=Homo sapiens GN=MYH9 PE=1 SV=4	6	39	41	62	65	46	35
P05787-2	K2C8_HUMAN	56608	KRT8	Isoform 2 of Keratin, type II cytoskeletal 8 OS=Homo sapiens GN=KRT8	6	35	47	62	62	39	42
P15531-2	NDKA_HUMAN	19654	NME1	Isoform 2 of Nucleoside diphosphate kinase A OS=Homo sapiens GN=NME1	6	57	55	35	49	40	45
Q86Y38	XYLT1_HUMAN	107569	XYLT1	Xylosyltransferase 1 OS=Homo sapiens GN=XYLT1 PE=1 SV=1	6	47	58	26	23	63	61
Q9HCU0	CD248_HUMAN	80859	CD248	Endosialin OS=Homo sapiens GN=CD248 PE=1 SV=1	6	46	43	31	20	64	70
P09341	GROA_HUMAN	11301	CXCL1	Growth-regulated alpha protein OS=Homo sapiens GN=CXCL1 PE=1 SV=1	6	64	54	13	12	46	83
O14786	NRP1_HUMAN	103134	NRP1	Neuropilin-1 OS=Homo sapiens GN=NRP1 PE=1 SV=3	6	36	30	38	32	65	70
P50395	GDIB_HUMAN	50663	GDI2	Rab GDP dissociation inhibitor beta OS=Homo sapiens GN=GDI2 PE=1 SV=2	6	36	39	44	42	33	76
Q9UBP4	DKK3_HUMAN	38390	DKK3	Dickkopf-related protein 3 OS=Homo sapiens GN=DKK3 PE=1 SV=2	6	65	65	24	21	50	43
P09211	GSTP1_HUMAN	23356	GSTP1	Glutathione S-transferase P OS=Homo sapiens GN=GSTP1 PE=1 SV=2	6	55	60	46	40	40	26
P22626	ROA2_HUMAN	37430	HNRNPA2B1	Heterogeneous nuclear ribonucleoproteins A2/B1 OS=Homo sapiens GN=HNRNPA2B1 PE=1 SV=2	6	59	57	35	39	35	42
P63104	1433Z_HUMAN	27745	YWHAZ	14-3-3 protein zeta/delta OS=Homo sapiens GN=YWHAZ PE=1 SV=1	6	56	60	33	33	39	42

Q13219	PAPP1_HUMAN	180973	PAPPA	Pappalysin-1 OS=Homo sapiens GN=PAPPA PE=1 SV=3	6	56	66	59	36	25	18
P07951-2	TPM2_HUMAN	32990	TPM2	Isoform 2 of Tropomyosin beta chain OS=Homo sapiens GN=TPM2	6	68	70	15	12	37	55
Q13509	TBB3_HUMAN	50433	TUBB3	Tubulin beta-3 chain OS=Homo sapiens GN=TUBB3 PE=1 SV=2	6	44	37	28	34	44	70
P37802	TAGL2_HUMAN	22391	TAGLN2	Transgelin-2 OS=Homo sapiens GN=TAGLN2 PE=1 SV=3	6	55	60	35	34	37	35
Q15113	PCOC1_HUMAN	47972	PCOLCE	Procollagen C-endopeptidase enhancer 1 OS=Homo sapiens GN=PCOLCE PE=1 SV=2	6	31	25	63	53	26	57
Q9Y4K0	LOXL2_HUMAN	86725	LOXL2	Lysyl oxidase homolog 2 OS=Homo sapiens GN=LOXL2 PE=1 SV=1	6	53	60	33	34	36	39
P18669	PGAM1_HUMAN	28804	PGAM1	Phosphoglycerate mutase 1 OS=Homo sapiens GN=PGAM1 PE=1 SV=2	6	45	44	46	45	40	34
P10915	HPLN1_HUMAN	40166	HAPLN1	Hyaluronan and proteoglycan link protein 1 OS=Homo sapiens GN=HAPLN1 PE=2 SV=2	5	82	76		2	37	54
P24592	IBP6_HUMAN	25322	IGFBP6	Insulin-like growth factor-binding protein 6 OS=Homo sapiens GN=IGFBP6 PE=1 SV=1	6	48	56	21	22	38	65
Q7Z3Y7	K1C28_HUMAN	50567	KRT28	Keratin, type I cytoskeletal 28 OS=Homo sapiens GN=KRT28 PE=1 SV=2	6	42	69	39	34	28	35
P02788-2	TRFL_HUMAN	73161	LTF	Isoform DeltaLf of Lactotransferrin OS=Homo sapiens GN=LTF	6	39	35	50	48	32	41
Q9NRN5	OLFL3_HUMAN	46010	OLFML3	Olfactomedin-like protein 3 OS=Homo sapiens GN=OLFML3 PE=2 SV=1	6	44	46	18	23	45	69
P29401-2	TKT_HUMAN	68814	TKT	Isoform 2 of Transketolase OS=Homo sapiens GN=TKT	6	54	49	35	35	37	32
P68104	EF1A1_HUMAN	50141	EEF1A1	Elongation factor 1-alpha 1 OS=Homo sapiens GN=EEF1A1 PE=1 SV=1	6	40	24	33	31	50	63
Q5VTE0	EF1A3_HUMAN	50185	EEF1A1P5	Putative elongation factor 1-alpha-like 3 OS=Homo sapiens GN=EEF1A1P5 PE=5 SV=1	6	40	24	33	31	50	63
P09651-2	ROA1_HUMAN	34196	HNRNPA1	Isoform A1-A of Heterogeneous nuclear ribonucleoprotein A1 OS=Homo sapiens GN=HNRNPA1	6	47	48	30	35	41	38
P05231	IL6_HUMAN	23718	IL6	Interleukin-6 OS=Homo sapiens GN=IL6 PE=1 SV=1	6	67	57	20	15	38	41
Q15063-3	POSTN_HUMAN	87254	POSTN	Isoform 3 of Periostin OS=Homo sapiens GN=POSTN	6	49	58	23	23	47	38
O95633-2	FSTL3_HUMAN	24960	FSTL3	Isoform 2 of Follistatin-related protein 3 OS=Homo sapiens GN=FSTL3	6	54	54	15	18	44	51
P00338	LDHA_HUMAN	36689	LDHA	L-lactate dehydrogenase A chain OS=Homo sapiens GN=LDHA PE=1 SV=2	6	43	38	36	40	37	41
P40926	MDHM_HUMAN	35503	MDH2	Malate dehydrogenase, mitochondrial OS=Homo sapiens GN=MDH2 PE=1 SV=3	6	33	33	43	47	31	48
Q9Y6C2	EMIL1_HUMAN	106667	EMILIN1	EMILIN-1 OS=Homo sapiens GN=EMILIN1 PE=1 SV=2	6	38	39	8	6	74	69
P63241	IF5A1_HUMAN	16832	EIF5A	Eukaryotic translation initiation factor 5A-1 OS=Homo sapiens GN=EIF5A PE=1 SV=2	6	48	53	23	25	35	49
P12110	CO6A2_HUMAN	108579	COL6A2	Collagen alpha-2(VI) chain OS=Homo sapiens GN=COL6A2 PE=1 SV=4	6	48	69	20	21	36	38
Q13308-6	PTK7_HUMAN	119197	PTK7	Isoform 6 of Inactive tyrosine-protein kinase 7 OS=Homo sapiens GN=PTK7	6	47	53	14	16	55	47
P55058	PLTP_HUMAN	54739	PLTP	Phospholipid transfer protein OS=Homo sapiens GN=PLTP PE=1 SV=1	6	62	78	12	12	32	33
P00751	CFAB_HUMAN	85533	CFB	Complement factor B OS=Homo sapiens GN=CFB PE=1 SV=2	6	46	40	23	16	49	54
P03956	MMP1_HUMAN	54007	MMP1	Interstitial collagenase OS=Homo sapiens GN=MMP1 PE=1 SV=3	6	81	83	6	5	19	28
O60462-3	NRP2_HUMAN	104283	NRP2	Isoform A17 of Neuropilin-2 OS=Homo sapiens GN=NRP2	6	47	52	19	18	35	50
Q14767	LTBP2_HUMAN	195052	LTBP2	Latent-transforming growth factor beta-binding protein 2 OS=Homo sapiens GN=LTBP2 PE=1 SV=3	6	44	49	25	19	44	38
Q14697	GANAB_HUMAN	106874	GANAB	Neutral alpha-glucosidase AB OS=Homo sapiens GN=GANAB PE=1 SV=3	6	35	39	16	21	57	49
Q9NY65-2	TBA8_HUMAN	42954	TUBA8	Isoform 2 of Tubulin alpha-8 chain OS=Homo sapiens GN=TUBA8	6	33	21	44	41	30	48
P06396	GELS_HUMAN	85698	GSN	Gelsolin OS=Homo sapiens GN=GSN PE=1 SV=1	6	36	40	35	38	29	38
P29966	MARCS_HUMAN	31555	MARCKS	Myristoylated alanine-rich C-kinase substrate OS=Homo sapiens GN=MARCKS PE=1 SV=4	6	27	27	33	32	42	55
F5H7N9	F5H7N9_HUMAN	42452	MFGE8	Lactadherin short form OS=Homo sapiens GN=MFGE8 PE=2 SV=1	6	45	35	13	16	34	73
O60361	NDK8_HUMAN	15529	NME2P1	Putative nucleoside diphosphate kinase OS=Homo sapiens GN=NME2P1 PE=5 SV=1	6	46	30	29	39	32	36
O95678	K2C75_HUMAN	59560	KRT75	Keratin, type II cytoskeletal 75 OS=Homo sapiens GN=KRT75 PE=1 SV=2	6	26	38	49	52	20	25
P04406	G3P_HUMAN	36053	GAPDH	Glyceraldehyde-3-phosphate dehydrogenase OS=Homo sapiens GN=GAPDH PE=1 SV=3	6	35	36	30	29	30	50
P09104	ENOG_HUMAN	47269	ENO2	Gamma-enolase OS=Homo sapiens GN=ENO2 PE=1 SV=3	6	40	13	37	39	22	58
P35609	ACTN2_HUMAN	103854	ACTN2	Alpha-actinin-2 OS=Homo sapiens GN=ACTN2 PE=1 SV=1	6	21	37	41	51	27	29
P15311	EZRI_HUMAN	69413	EZR	Ezrin OS=Homo sapiens GN=EZR PE=1 SV=4	6	46	35	35	37	29	23
P27797	CALR_HUMAN	48142	CALR	Calreticulin OS=Homo sapiens GN=CALR PE=1 SV=1	6	55	40	27	23	17	43
P61981	1433G_HUMAN	28303	YWHAG	14-3-3 protein gamma OS=Homo sapiens GN=YWHAG PE=1 SV=2	6	38	45	31	29	29	33
Q7Z3Y8	K1C27_HUMAN	49822	KRT27	Keratin, type I cytoskeletal 27 OS=Homo sapiens GN=KRT27 PE=1 SV=2	6	34	59	27	29	23	32
Q9Y490	TLN1_HUMAN	269767	TLN1	Talin-1 OS=Homo sapiens GN=TLN1 PE=1 SV=3	6	25	9	43	40	37	49
P02545	LMNA_HUMAN	74139	LMNA	Prelamin-A/C OS=Homo sapiens GN=LMNA PE=1 SV=1	6	26	26	48	49	26	26

P15291-2	B4GT1_HUMAN	42538	B4GALT1	Isoform Short of Beta-1,4-galactosyltransferase 1 OS=Homo sapiens GN=B4GALT1	6	41	30	9	7	35	76
P62979	RS27A_HUMAN	17965	RPS27A	Ubiquitin-40S ribosomal protein S27a OS=Homo sapiens GN=RPS27A PE=1 SV=2	6	43	43	22	24	29	36
Q15149	PLEC_HUMAN	531791	PLEC	Plectin OS=Homo sapiens GN=PLEC PE=1 SV=3	6	22	18	57	53	20	27
Q06830	PRDX1_HUMAN	22110	PRDX1	Peroxiredoxin-1 OS=Homo sapiens GN=PRDX1 PE=1 SV=1	6	32	31	40	43	30	20
P08603	CFAH_HUMAN	139096	CFH	Complement factor H OS=Homo sapiens GN=CFH PE=1 SV=4	6	37	30	33	33	35	27
P14543	NID1_HUMAN	136377	NID1	Nidogen-1 OS=Homo sapiens GN=NID1 PE=1 SV=3	6	27	31	29	30	41	37
Q6IS14	IF5AL_HUMAN	16773	EIF5AL1	Eukaryotic translation initiation factor 5A-1-like OS=Homo sapiens GN=EIF5AL1 PE=1 SV=2	6	46	44	17	20	26	42
P07237	PDIA1_HUMAN	57116	P4HB	Protein disulfide-isomerase OS=Homo sapiens GN=P4HB PE=1 SV=3	6	47	40	21	18	30	38
P50454	SERPH_HUMAN	46441	SERPINH1	Serpin H1 OS=Homo sapiens GN=SERPINH1 PE=1 SV=2	6	20	21	22	20	42	69
P0CG47	UBB_HUMAN	25762	UBB	Polyubiquitin-B OS=Homo sapiens GN=UBB PE=1 SV=1	6	42	43	22	24	28	34
P0CG48	UBC_HUMAN	77039	UBC	Polyubiquitin-C OS=Homo sapiens GN=UBC PE=1 SV=3	6	42	43	22	24	28	34
P13693	TCTP_HUMAN	19595	TPT1	Translationally-controlled tumor protein OS=Homo sapiens GN=TPT1 PE=1 SV=1	6	40	43	25	31	32	22
P62987	RL40_HUMAN	14728	UBA52	Ubiquitin-60S ribosomal protein L40 OS=Homo sapiens GN=UBA52 PE=1 SV=2	6	42	43	22	24	28	34
P02774-3	VTDB_HUMAN	55123	GC	Isoform 3 of Vitamin D-binding protein OS=Homo sapiens GN=GC	6	30	28	27	31	28	48
P07355	ANXA2_HUMAN	38604	ANXA2	Annexin A2 OS=Homo sapiens GN=ANXA2 PE=1 SV=2	6	32	32	39	41	20	25
Q7Z3Z0	K1C25_HUMAN	49318	KRT25	Keratin, type I cytoskeletal 25 OS=Homo sapiens GN=KRT25 PE=1 SV=1	6	33	45	27	29	23	32
Q8NBJ4-2	GOLM1_HUMAN	44273	GOLM1	Isoform 2 of Golgi membrane protein 1 OS=Homo sapiens GN=GOLM1	6	48	52	7	10	32	40
P09493-3	TPM1_HUMAN	32876	TPM1	Isoform 3 of Tropomyosin alpha-1 chain OS=Homo sapiens GN=TPM1	6	48	49	11	8	30	42
P19876	CXCL3_HUMAN	11342	CXCL3	C-X-C motif chemokine 3 OS=Homo sapiens GN=CXCL3 PE=1 SV=1	6	45	40	5	8	28	62
Q6YHK3	CD109_HUMAN	161689	CD109	CD109 antigen OS=Homo sapiens GN=CD109 PE=1 SV=2	6	23	22	21	21	50	51
Q504U3	Q504U3_HUMAN	40164	PKM2	Pyruvate kinase OS=Homo sapiens GN=PKM2 PE=2 SV=1	6	53	45	15	15	30	30
P13929-2	ENOB_HUMAN	44115	ENO3	Isoform 2 of Beta-enolase OS=Homo sapiens GN=ENO3	6	38	12	32	33	19	52
P02771	FETA_HUMAN	68678	AFP	Alpha-fetoprotein OS=Homo sapiens GN=AFP PE=1 SV=1	6	9	10	41	52	35	37
P27348	1433T_HUMAN	27764	YWHAQ	14-3-3 protein theta OS=Homo sapiens GN=YWHAQ PE=1 SV=1	6	33	35	30	29	28	28
P31946-2	1433B_HUMAN	27850	YWHAB	Isoform Short of 14-3-3 protein beta/alpha OS=Homo sapiens GN=YWHAB	6	41	35	23	23	28	33
P69905	HBA_HUMAN	15258	HBA1	Hemoglobin subunit alpha OS=Homo sapiens GN=HBA1 PE=1 SV=2	6	26	23	30	32	29	43
Q32P51	RA1L2_HUMAN	34225	HNRNPA1L2	Heterogeneous nuclear ribonucleoprotein A1-like 2 OS=Homo sapiens GN=HNRNPA1L2 PE=2 SV=2	6	41	44	21	26	26	25
P61916	NPC2_HUMAN	16570	NPC2	Epididymal secretory protein E1 OS=Homo sapiens GN=NPC2 PE=1 SV=1	6	44	39	22	19	24	33
Q5XKE5	K2C79_HUMAN	57836	KRT79	Keratin, type II cytoskeletal 79 OS=Homo sapiens GN=KRT79 PE=1 SV=2	6	22	36	44	40	16	23
Q13813-3	SPTN1_HUMAN	282282	SPTAN1	Isoform 3 of Spectrin alpha chain, non-erythrocytic 1 OS=Homo sapiens GN=SPTAN1	6	18	14	54	60	20	14
Q6EMK4	VASN_HUMAN	71713	VASN	Vasorin OS=Homo sapiens GN=VASN PE=1 SV=1	6	44	50	12	14	29	31
Q07954	LRP1_HUMAN	504606	LRP1	Prolow-density lipoprotein receptor-related protein 1 OS=Homo sapiens GN=LRP1 PE=1 SV=2	6	7	9	39	33	46	45
P00491	PNPH_HUMAN	32118	PNP	Purine nucleoside phosphorylase OS=Homo sapiens GN=PNP PE=1 SV=2	6	27	44	34	32	23	18
P13646	K1C13_HUMAN	49588	KRT13	Keratin, type I cytoskeletal 13 OS=Homo sapiens GN=KRT13 PE=1 SV=4	6	29	51	33	34	15	16
P19875	CXCL2_HUMAN	11389	CXCL2	C-X-C motif chemokine 2 OS=Homo sapiens GN=CXCL2 PE=1 SV=1	6	43	37	5	8	26	59
P06744	G6PI_HUMAN	63147	GPI	Glucose-6-phosphate isomerase OS=Homo sapiens GN=GPI PE=1 SV=4	6	26	16	35	36	21	42
P35241-5	RADI_HUMAN	71049	RDX	Isoform 5 of Radixin OS=Homo sapiens GN=RDX	6	39	29	30	30	28	20
O00462	MANBA_HUMAN	100895	MANBA	Beta-mannosidase OS=Homo sapiens GN=MANBA PE=1 SV=3	6	36	57	17	16	26	23
Q01546	K22O_HUMAN	65841	KRT76	Keratin, type II cytoskeletal 2 oral OS=Homo sapiens GN=KRT76 PE=1 SV=2	6	21	35	44	36	16	22
P30086	PEBP1_HUMAN	21057	PEBP1	Phosphatidylethanolamine-binding protein 1 OS=Homo sapiens GN=PEBP1 PE=1 SV=3	6	36	29	29	35	19	21
P80303-2	NUCB2_HUMAN	50201	NUCB2	Isoform 2 of Nucleobindin-2 OS=Homo sapiens GN=NUCB2	6	37	26	2	2	30	71
P68871	HBB_HUMAN	15998	HBB	Hemoglobin subunit beta OS=Homo sapiens GN=HBB PE=1 SV=2	6	27	31	34	24	27	21
Q10471	GALT2_HUMAN	64733	GALNT2	Polypeptide N-acetylgalactosaminyltransferase 2 OS=Homo sapiens GN=GALNT2 PE=1 SV=1	6	36	35	11	10	36	36
P34932	HSP74_HUMAN	94331	HSPA4	Heat shock 70 kDa protein 4 OS=Homo sapiens GN=HSPA4 PE=1 SV=4	6	28	34	26	30	25	20
P06753-2	TPM3_HUMAN	29033	TPM3	Isoform 2 of Tropomyosin alpha-3 chain OS=Homo sapiens GN=TPM3	6	39	37	15	14	22	34
P28300	LYOX_HUMAN	46944	LOX	Protein-lysine 6-oxidase OS=Homo sapiens GN=LOX PE=1 SV=2	6	26	22	6	7	46	54

J3QLP7	J3QLP7_HUMAN	25154	UBBP4	Protein UBBP4 OS=Homo sapiens GN=UBBP4 PE=2 SV=1	6	35	37	17	20	21	29
P01008	ANT3_HUMAN	52602	SERPINC1	Antithrombin-III OS=Homo sapiens GN=SERPINC1 PE=1 SV=1	6	12	9	40	37	24	36
Q16658	FSCN1_HUMAN	54530	FSCN1	Fascin OS=Homo sapiens GN=FSCN1 PE=1 SV=3	6	25	19	27	27	23	37
P31150	GDIA_HUMAN	50583	GDI1	Rab GDP dissociation inhibitor alpha OS=Homo sapiens GN=GDI1 PE=1 SV=2	6	21	21	26	26	22	40
Q93063-3	EXT2_HUMAN	85815	EXT2	Isoform 3 of Exostosin-2 OS=Homo sapiens GN=EXT2	6	36	33	14	17	29	26
O75083	WDR1_HUMAN	66194	WDR1	WD repeat-containing protein 1 OS=Homo sapiens GN=WDR1 PE=1 SV=4	6	27	24	35	30	19	18
Q92820	GGH_HUMAN	35964	GGH	Gamma-glutamyl hydrolase OS=Homo sapiens GN=GGH PE=1 SV=2	6	28	23	18	13	21	50
Q16706	MA2A1_HUMAN	131141	MAN2A1	Alpha-mannosidase 2 OS=Homo sapiens GN=MAN2A1 PE=1 SV=2	6	15	32	23	20	33	29
O00622	CYR61_HUMAN	42027	CYR61	Protein CYR61 OS=Homo sapiens GN=CYR61 PE=1 SV=1	6	36	31	15	17	18	32
O75635-2	SPB7_HUMAN	41174	SERPINB7	Isoform 2 of Serpin B7 OS=Homo sapiens GN=SERPINB7	6	8	8	13	13	32	75
Q7Z794	K2C1B_HUMAN	61901	KRT77	Keratin, type II cytoskeletal 1b OS=Homo sapiens GN=KRT77 PE=2 SV=3	6	22	28	35	26	18	20
Q9GZV4	IF5A2_HUMAN	16793	EIF5A2	Eukaryotic translation initiation factor 5A-2 OS=Homo sapiens GN=EIF5A2 PE=1 SV=3	6	39	37	10	13	17	33
Q8NBS9	TXND5_HUMAN	47629	TXNDC5	Thioredoxin domain-containing protein 5 OS=Homo sapiens GN=TXNDC5 PE=1 SV=2	6	27	25	17	22	19	38
P04792	HSPB1_HUMAN	22783	HSPB1	Heat shock protein beta-1 OS=Homo sapiens GN=HSPB1 PE=1 SV=2	6	35	36	7	8	29	32
P35556	FBN2_HUMAN	314775	FBN2	Fibrillin-2 OS=Homo sapiens GN=FBN2 PE=1 SV=3	6	25	27	13	11	35	36
F5GWP8	F5GWP8_HUMAN	66350	JUP	Junction plakoglobin OS=Homo sapiens GN=JUP PE=2 SV=1	6	18	25	38	42	11	13
P01871-2	IGHM_HUMAN	51790	IGHM	Isoform 2 of Ig mu chain C region OS=Homo sapiens GN=IGHM	4			46	63	29	9
P11717	MPRI_HUMAN	274375	IGF2R	Cation-independent mannose-6-phosphate receptor OS=Homo sapiens GN=IGF2R PE=1 SV=3	6	10	8	29	23	40	35
P13500	CCL2_HUMAN	11025	CCL2	C-C motif chemokine 2 OS=Homo sapiens GN=CCL2 PE=1 SV=1	6	37	28	11	12	24	33
P52565	GDIR1_HUMAN	23207	ARHGDIA	Rho GDP-dissociation inhibitor 1 OS=Homo sapiens GN=ARHGDIA PE=1 SV=3	6	23	27	22	24	21	27
Q04695	K1C17_HUMAN	48106	KRT17	Keratin, type I cytoskeletal 17 OS=Homo sapiens GN=KRT17 PE=1 SV=2	6	19	26	33	42	10	14
P17936-2	IBP3_HUMAN	32223	IGFBP3	Isoform 2 of Insulin-like growth factor-binding protein 3 OS=Homo sapiens GN=IGFBP3	6	21	27	17	16	24	38
Q13740-2	CD166_HUMAN	63665	ALCAM	Isoform 2 of CD166 antigen OS=Homo sapiens GN=ALCAM	6	15	20	28	27	26	27
Q9BUF5	TBB6_HUMAN	49857	TUBB6	Tubulin beta-6 chain OS=Homo sapiens GN=TUBB6 PE=1 SV=1	6	23	18	22	25	23	32
P14625	ENPL_HUMAN	92469	HSP90B1	Endoplasmin OS=Homo sapiens GN=HSP90B1 PE=1 SV=1	6	28	30	19	22	22	19
P21291	CSRP1_HUMAN	20567	CSRP1	Cysteine and glycine-rich protein 1 OS=Homo sapiens GN=CSRP1 PE=1 SV=3	6	26	27	24	20	23	20
Q02809	PLOD1_HUMAN	83550	PLOD1	Procollagen-lysine,2-oxoglutarate 5-dioxygenase 1 OS=Homo sapiens GN=PLOD1 PE=1 SV=2	6	18	33	22	16	26	25
Q14019	COTL1_HUMAN	15945	COTL1	Coactosin-like protein OS=Homo sapiens GN=COTL1 PE=1 SV=3	6	22	12	22	22	34	28
Q3ZCM7	TBB8_HUMAN	49776	TUBB8	Tubulin beta-8 chain OS=Homo sapiens GN=TUBB8 PE=1 SV=2	6	22	20	18	19	24	34
P30041	PRDX6_HUMAN	25035	PRDX6	Peroxiredoxin-6 OS=Homo sapiens GN=PRDX6 PE=1 SV=3	6	25	25	22	17	23	24
Q01469	FABP5_HUMAN	15164	FABP5	Fatty acid-binding protein, epidermal OS=Homo sapiens GN=FABP5 PE=1 SV=3	6	31	22	19	22	22	20
Q08043	ACTN3_HUMAN	103241	ACTN3	Alpha-actinin-3 OS=Homo sapiens GN=ACTN3 PE=1 SV=2	6	20	30	26	26	16	17
Q05682-4	CALD1_HUMAN	62663	CALD1	Isoform 4 of Caldesmon OS=Homo sapiens GN=CALD1	6	24	17	16	16	29	32
O00469-2	PLOD2_HUMAN	87098	PLOD2	Isoform 2 of Procollagen-lysine,2-oxoglutarate 5-dioxygenase 2 OS=Homo sapiens GN=PLOD2	6	20	19	16	12	32	34
P23526	SAHH_HUMAN	47716	AHCY	Adenosylhomocysteinase OS=Homo sapiens GN=AHCY PE=1 SV=4	6	14	16	23	26	19	35
P61158	ARP3_HUMAN	47371	ACTR3	Actin-related protein 3 OS=Homo sapiens GN=ACTR3 PE=1 SV=3	6	18	15	26	26	19	29
Q969H8	CS010_HUMAN	18795	C19orf10	UPF0556 protein C19orf10 OS=Homo sapiens GN=C19orf10 PE=1 SV=1	6	26	26	16	16	25	23
P54652	HSP72_HUMAN	70021	HSPA2	Heat shock-related 70 kDa protein 2 OS=Homo sapiens GN=HSPA2 PE=1 SV=1	6	31	24	16	20	19	21
P12035	K2C3_HUMAN	64417	KRT3	Keratin, type II cytoskeletal 3 OS=Homo sapiens GN=KRT3 PE=1 SV=3	6	10	22	42	33	13	10
P60660-2	MYL6_HUMAN	16961	MYL6	Isoform Smooth muscle of Myosin light polypeptide 6 OS=Homo sapiens GN=MYL6	6	27	29	21	15	16	22
P09603	CSF1_HUMAN	60179	CSF1	Macrophage colony-stimulating factor 1 OS=Homo sapiens GN=CSF1 PE=1 SV=2	6	17	16	22	20	29	25
Q16881-6	TRXR1_HUMAN	67116	TXNRD1	Isoform 6 of Thioredoxin reductase 1, cytoplasmic OS=Homo sapiens GN=TXNRD1	6	21	12	40	29	15	12
P02042	HBD_HUMAN	16055	HBD	Hemoglobin subunit delta OS=Homo sapiens GN=HBD PE=1 SV=2	6	21	24	23	20	21	17
P07900-2	HS90A_HUMAN	98161	HSP90AA1	Isoform 2 of Heat shock protein HSP 90-alpha OS=Homo sapiens GN=HSP90AA1	6	20	36	17	16	19	18
Q9UBR2	CATZ_HUMAN	33868	CTSZ	Cathepsin Z OS=Homo sapiens GN=CTSZ PE=1 SV=1	6	23	16	12	13	26	36
P02462	CO4A1_HUMAN	160615	COL4A1	Collagen alpha-1(IV) chain OS=Homo sapiens GN=COL4A1 PE=1 SV=3	6	40	26	7	8	26	18
P40925-3	MDHC_HUMAN	38628	MDH1	Isoform 3 of Malate dehydrogenase, cytoplasmic OS=Homo sapiens GN=MDH1	6	23	24	21	27	12	17

O75882-2	ATRN_HUMAN	141429	ATRN	Isoform 2 of Attractin OS=Homo sapiens GN=ATRN	6	20	23	11	10	29	30
O76061	STC2_HUMAN	33249	STC2	Stanniocalcin-2 OS=Homo sapiens GN=STC2 PE=1 SV=1	6	22	24	10	14	22	31
P22314	UBA1_HUMAN	117849	UBA1	Ubiquitin-like modifier-activating enzyme 1 OS=Homo sapiens GN=UBA1 PE=1 SV=3	6	23	27	19	15	20	19
P08238	HS90B_HUMAN	83264	HSP90AB1	Heat shock protein HSP 90-beta OS=Homo sapiens GN=HSP90AB1 PE=1 SV=4	6	18	34	21	18	16	15
Q01518-2	CAP1_HUMAN	51830	CAP1	Isoform 2 of Adenylyl cyclase-associated protein 1 OS=Homo sapiens GN=CAP1	6	16	17	26	23	14	25
P47756-2	CAPZB_HUMAN	30629	CAPZB	Isoform 2 of F-actin-capping protein subunit beta OS=Homo sapiens GN=CAPZB	6	17	25	24	24	15	15
P0C0L4-2	CO4A_HUMAN	187704	C4A	Isoform 2 of Complement C4-A OS=Homo sapiens GN=C4A	6	6	6	24	26	35	22
P62158	CALM_HUMAN	16838	CALM1	Calmodulin OS=Homo sapiens GN=CALM1 PE=1 SV=2	6	20	28	16	19	13	23
A6NMY6	AXA2L_HUMAN	38659	ANXA2P2	Putative annexin A2-like protein OS=Homo sapiens GN=ANXA2P2 PE=5 SV=2	6	19	20	26	28	11	14
P55072	TERA_HUMAN	89322	VCP	Transitional endoplasmic reticulum ATPase OS=Homo sapiens GN=VCP PE=1 SV=4	6	8	15	38	34	13	9
Q12805-2	FBLN3_HUMAN	53722	EFEMP1	Isoform 2 of EGF-containing fibulin-like extracellular matrix protein 1 OS=Homo sapiens GN=EFEMP1	6	14	11	17	13	36	26
P01130-3	LDLR_HUMAN	76953	LDLR	Isoform 3 of Low-density lipoprotein receptor OS=Homo sapiens GN=LDLR	6	21	27	15	11	19	23
Q15084-2	PDIA6_HUMAN	53901	PDIA6	Isoform 2 of Protein disulfide-isomerase A6 OS=Homo sapiens GN=PDIA6	6	20	20	14	14	16	32
P29279	CTGF_HUMAN	38091	CTGF	Connective tissue growth factor OS=Homo sapiens GN=CTGF PE=1 SV=2	6	23	26	26	13	11	16
P41219-2	PERI_HUMAN	53779	PRPH	Isoform 2 of Peripherin OS=Homo sapiens GN=PRPH	6	25	28	11	8	20	23
Q7Z3Y9	K1C26_HUMAN	51911	KRT26	Keratin, type I cytoskeletal 26 OS=Homo sapiens GN=KRT26 PE=1 SV=2	6	20	28	17	17	12	21
P46940	IQGA1_HUMAN	189252	IQGAP1	Ras GTPase-activating-like protein IQGAP1 OS=Homo sapiens GN=IQGAP1 PE=1 SV=1	6	14	13	30	21	23	13
Q9Y281	COF2_HUMAN	18737	CFL2	Cofilin-2 OS=Homo sapiens GN=CFL2 PE=1 SV=1	6	15	19	13	19	20	28
P02647	APOA1_HUMAN	30778	APOA1	Apolipoprotein A-I OS=Homo sapiens GN=APOA1 PE=1 SV=1	6	16	20	20	21	18	18
P35052	GPC1_HUMAN	61680	GPC1	Glypican-1 OS=Homo sapiens GN=GPC1 PE=1 SV=2	6	26	20	14	14	14	25
Q92859-2	NEO1_HUMAN	154304	NEO1	Isoform 2 of Neogenin OS=Homo sapiens GN=NEO1	6	10	14	26	23	23	16
Q99497	PARK7_HUMAN	19891	PARK7	Protein DJ-1 OS=Homo sapiens GN=PARK7 PE=1 SV=2	6	22	21	20	20	18	11
P19012	K1C15_HUMAN	49212	KRT15	Keratin, type I cytoskeletal 15 OS=Homo sapiens GN=KRT15 PE=1 SV=3	6	17	28	21	28	7	10
P36955	PEDF_HUMAN	46312	SERPINF1	Pigment epithelium-derived factor OS=Homo sapiens GN=SERPINF1 PE=1 SV=4	6	11	8	30	29	9	24
H0Y7A7	H0Y7A7_HUMAN	20762	CALM2	Calmodulin (Fragment) OS=Homo sapiens GN=CALM2 PE=2 SV=1	6	19	26	15	18	12	21
P04083	ANXA1_HUMAN	38714	ANXA1	Annexin A1 OS=Homo sapiens GN=ANXA1 PE=1 SV=2	6	14	11	18	24	21	21
P34931	HS71L_HUMAN	70375	HSPA1L	Heat shock 70 kDa protein 1-like OS=Homo sapiens GN=HSPA1L PE=1 SV=2	6	25	20	13	17	16	18
P37837	TALDO_HUMAN	37540	TALDO1	Transaldolase OS=Homo sapiens GN=TALDO1 PE=1 SV=2	6	13	16	23	24	12	21
P60842	IF4A1_HUMAN	46154	EIF4A1	Eukaryotic initiation factor 4A-I OS=Homo sapiens GN=EIF4A1 PE=1 SV=1	6	13	11	14	18	20	33
P10599	THIO_HUMAN	11737	TXN	Thioredoxin OS=Homo sapiens GN=TXN PE=1 SV=3	6	17	15	17	20	28	11
P19823	ITIH2_HUMAN	106463	ITIH2	Inter-alpha-trypsin inhibitor heavy chain H2 OS=Homo sapiens GN=ITIH2 PE=1 SV=2	6	12	17	30	23	15	11
K7ELL7	K7ELL7_HUMAN	60192	PRKCSH	Glucosidase 2 subunit beta OS=Homo sapiens GN=PRKCSH PE=2 SV=1	6	18	28	16	18	14	14
P16070-10	CD44_HUMAN	53411	CD44	Isoform 10 of CD44 antigen OS=Homo sapiens GN=CD44	6	6	15	23	23	20	20
P60981	DEST_HUMAN	18506	DSTN	Destrin OS=Homo sapiens GN=DSTN PE=1 SV=3	6	18	20	18	13	23	15
O00299	CLIC1_HUMAN	26923	CLIC1	Chloride intracellular channel protein 1 OS=Homo sapiens GN=CLIC1 PE=1 SV=4	6	21	19	17	14	19	16
P02787	TRFE_HUMAN	77064	TF	Serotransferrin OS=Homo sapiens GN=TF PE=1 SV=3	6	11	14	26	22	13	20
P32119	PRDX2_HUMAN	21892	PRDX2	Peroxiredoxin-2 OS=Homo sapiens GN=PRDX2 PE=1 SV=5	6	18	12	23	22	15	16
P34096	RNAS4_HUMAN	16840	RNASE4	Ribonuclease 4 OS=Homo sapiens GN=RNASE4 PE=1 SV=3	6	19	20	6	7	23	30
P52907	CAZA1_HUMAN	32923	CAPZA1	F-actin-capping protein subunit alpha-1 OS=Homo sapiens GN=CAPZA1 PE=1 SV=3	6	15	15	19	18	15	22
P35237	SPB6_HUMAN	42622	SERPINB6	Serpin B6 OS=Homo sapiens GN=SERPINB6 PE=1 SV=3	6	12	8	20	19	16	28
P09972	ALDOC_HUMAN	39456	ALDOC	Fructose-bisphosphate aldolase C OS=Homo sapiens GN=ALDOC PE=1 SV=2	6	16	14	15	17	14	26
P27695	APEX1_HUMAN	35555	APEX1	DNA-(apurinic or apyrimidinic site) lyase OS=Homo sapiens GN=APEX1 PE=1 SV=2	6	12	14	23	24	8	21
P52209	6PGD_HUMAN	53140	PGD	6-phosphogluconate dehydrogenase, decarboxylating OS=Homo sapiens GN=PGD PE=1 SV=3	6	17	14	6	8	21	36
P55083-2	MFAP4_HUMAN	33173	MFAP4	Isoform 2 of Microfibril-associated glycoprotein 4 OS=Homo sapiens GN=MFAP4	6	4	4	4	2	35	53
P00505	AATM_HUMAN	47518	GOT2	Aspartate aminotransferase, mitochondrial OS=Homo sapiens GN=GOT2 PE=1 SV=3	6	15	9	20	24	8	25
Q04917	1433F_HUMAN	28219	YWHAH	14-3-3 protein eta OS=Homo sapiens GN=YWHAH PE=1 SV=4	6	20	16	12	17	17	19
P0C0L5	CO4B_HUMAN	192751	C4B	Complement C4-B OS=Homo sapiens GN=C4B PE=1 SV=2	6	5	5	19	23	32	16
Q14118	DAG1_HUMAN	97441	DAG1	Dystroglycan OS=Homo sapiens GN=DAG1 PE=1 SV=2	6	12	13	9	10	24	32
Q8N6G6	ATL1_HUMAN	193409	ADAMTSL1	ADAMTS-like protein 1 OS=Homo sapiens GN=ADAMTSL1 PE=1 SV=4	6	28	22	9	11	9	21

P08727	K1C19_HUMAN	44106	KRT19	Keratin, type I cytoskeletal 19 OS=Homo sapiens GN=KRT19 PE=1 SV=4	6	15	15	22	31	8	8
P19013	K2C4_HUMAN	57285	KRT4	Keratin, type II cytoskeletal 4 OS=Homo sapiens GN=KRT4 PE=1 SV=4	6	16	20	19	18	10	16
P55786	PSA_HUMAN	103276	NPEPPS	Puromycin-sensitive aminopeptidase OS=Homo sapiens GN=NPEPPS PE=1 SV=2	6	16	19	23	23	10	8
Q96CG8	CTHR1_HUMAN	26224	CTHRC1	Collagen triple helix repeat-containing protein 1 OS=Homo sapiens GN=CTHRC1 PE=1 SV=1	6	14	19	15	15	23	13
Q16363-2	LAMA4_HUMAN	201776	LAMA4	Isoform 2 of Laminin subunit alpha-4 OS=Homo sapiens GN=LAMA4	6	24	27	4	2	23	18
Q16769	QPCT_HUMAN	40877	QPCT	Glutaminyl-peptide cyclotransferase OS=Homo sapiens GN=QPCT PE=1 SV=1	6	21	25	12	9	12	19
Q2M2I5	K1C24_HUMAN	55087	KRT24	Keratin, type I cytoskeletal 24 OS=Homo sapiens GN=KRT24 PE=1 SV=1	6	21	35	15	12	7	8
P02100	HBE_HUMAN	16203	HBE1	Hemoglobin subunit epsilon OS=Homo sapiens GN=HBE1 PE=1 SV=2	6	16	19	16	16	17	13
P06576	ATPB_HUMAN	56560	ATP5B	ATP synthase subunit beta, mitochondrial OS=Homo sapiens GN=ATP5B PE=1 SV=3	6	7	3	28	26	11	22
P30447	1A23_HUMAN	40733	HLA-A	HLA class I histocompatibility antigen, A-23 alpha chain OS=Homo sapiens GN=HLA-A PE=1 SV=1	6	10	7	7	8	26	39
P69891	HBG1_HUMAN	16140	HBG1	Hemoglobin subunit gamma-1 OS=Homo sapiens GN=HBG1 PE=1 SV=2	6	16	19	16	16	17	13
P69892	HBG2_HUMAN	16126	HBG2	Hemoglobin subunit gamma-2 OS=Homo sapiens GN=HBG2 PE=1 SV=2	6	16	19	16	16	17	13
Q7Z3B1	NEGR1_HUMAN	38719	NEGR1	Neuronal growth regulator 1 OS=Homo sapiens GN=NEGR1 PE=1 SV=3	6	21	16	4	5	22	29
B4E1F0	B4E1F0_HUMAN	55769	SERPING1	Plasma protease C1 inhibitor OS=Homo sapiens GN=SERPING1 PE=2 SV=1	6	22	30	9	11	11	14
P02458	CO2A1_HUMAN	141785	COL2A1	Collagen alpha-1(II) chain OS=Homo sapiens GN=COL2A1 PE=1 SV=3	6	18	21	12	9	17	19
O14950	ML12B_HUMAN	19779	MYL12B	Myosin regulatory light chain 12B OS=Homo sapiens GN=MYL12B PE=1 SV=2	6	22	24	9	7	16	17
O75368	SH3L1_HUMAN	12774	SH3BGRL	SH3 domain-binding glutamic acid-rich-like protein OS=Homo sapiens GN=SH3BGRL PE=1 SV=1	6	16	19	15	14	15	16
P15259	PGAM2_HUMAN	28766	PGAM2	Phosphoglycerate mutase 2 OS=Homo sapiens GN=PGAM2 PE=1 SV=3	6	21	18	16	15	12	13
P19105	ML12A_HUMAN	19794	MYL12A	Myosin regulatory light chain 12A OS=Homo sapiens GN=MYL12A PE=1 SV=2	6	22	24	9	7	16	17
P07339	CATD_HUMAN	44552	CTSD	Cathepsin D OS=Homo sapiens GN=CTSD PE=1 SV=1	6	14	10	18	12	16	24
Q9Y696	CLIC4_HUMAN	28772	CLIC4	Chloride intracellular channel protein 4 OS=Homo sapiens GN=CLIC4 PE=1 SV=4	6	20	17	11	13	16	17
Q9ULV4-2	COR1C_HUMAN	54067	CORO1C	Isoform 2 of Coronin-1C OS=Homo sapiens GN=CORO1C	6	8	6	20	14	14	31
O75874	IDHC_HUMAN	46659	IDH1	Isocitrate dehydrogenase [NADP] cytoplasmic OS=Homo sapiens GN=IDH1 PE=1 SV=2	6	14	10	12	11	14	30
P02753	RET4_HUMAN	23010	RBP4	Retinol-binding protein 4 OS=Homo sapiens GN=RBP4 PE=1 SV=3	6	7	6	36	23	9	10
P21399	ACOC_HUMAN	98399	ACO1	Cytoplasmic aconitate hydratase OS=Homo sapiens GN=ACO1 PE=1 SV=3	6	15	19	14	18	13	12
P53396-2	ACLY_HUMAN	119772	ACLY	Isoform 2 of ATP-citrate synthase OS=Homo sapiens GN=ACLY	6	10	11	20	26	11	13
Q14126	DSG2_HUMAN	122294	DSG2	Desmoglein-2 OS=Homo sapiens GN=DSG2 PE=1 SV=2	6	22	24	10	11	12	12
P99999	CYC_HUMAN	11749	CYCS	Cytochrome c OS=Homo sapiens GN=CYCS PE=1 SV=2	6	17	18	11	17	14	13
O15144	ARPC2_HUMAN	34333	ARPC2	Actin-related protein 2/3 complex subunit 2 OS=Homo sapiens GN=ARPC2 PE=1 SV=1	6	15	9	13	23	13	16
P05783	K1C18_HUMAN	48058	KRT18	Keratin, type I cytoskeletal 18 OS=Homo sapiens GN=KRT18 PE=1 SV=2	6	16	14	12	9	20	18
P55001-2	MFAP2_HUMAN	20754	MFAP2	Isoform A' of Microfibrillar-associated protein 2 OS=Homo sapiens GN=MFAP2	6	24	22	6	6	16	15
P68036-3	UB2L3_HUMAN	24004	UBE2L3	Isoform 3 of Ubiquitin-conjugating enzyme E2 L3 OS=Homo sapiens GN=UBE2L3	6	16	13	14	13	15	18
O14498	ISLR_HUMAN	45997	ISLR	Immunoglobulin superfamily containing leucine-rich repeat protein OS=Homo sapiens GN=ISLR PE=1 SV=1	6	8	4	4	2	23	47
P26599-3	PTBP1_HUMAN	59633	PTBP1	Isoform 3 of Polypyrimidine tract-binding protein 1 OS=Homo sapiens GN=PTBP1	6	16	7	14	19	15	17
P08107	HSP71_HUMAN	70052	HSPA1A	Heat shock 70 kDa protein 1A/1B OS=Homo sapiens GN=HSPA1A PE=1 SV=5	6	21	19	9	10	16	12
P17174	AATC_HUMAN	46248	GOT1	Aspartate aminotransferase, cytoplasmic OS=Homo sapiens GN=GOT1 PE=1 SV=3	6	11	3	16	22	10	25
P24844	MYL9_HUMAN	19827	MYL9	Myosin regulatory light polypeptide 9 OS=Homo sapiens GN=MYL9 PE=1 SV=4	6	19	23	9	7	15	14
P33908	MA1A1_HUMAN	72969	MAN1A1	Mannosyl-oligosaccharide 1,2-alpha-mannosidase IA OS=Homo sapiens GN=MAN1A1 PE=1 SV=3	6	18	19	9	8	21	12
Q00610-2	CLH1_HUMAN	187890	CLTC	Isoform 2 of Clathrin heavy chain 1 OS=Homo sapiens GN=CLTC	6	2	3	42	24	10	5
Q15262-2	PTPRK_HUMAN	162173	PTPRK	Isoform 2 of Receptor-type tyrosine-protein phosphatase kappa OS=Homo sapiens GN=PTPRK	6	13	20	11	8	15	19
Q9GZX9	TWSG1_HUMAN	25017	TWSG1	Twisted gastrulation protein homolog 1 OS=Homo sapiens GN=TWSG1 PE=2 SV=1	6	19	16	2	5	16	28
Q6IBS0	TWF2_HUMAN	39548	TWF2	Twinfilin-2 OS=Homo sapiens GN=TWF2 PE=1 SV=2	6	16	14	12	17	12	14
P14550	AK1A1_HUMAN	36573	AKR1A1	Alcohol dehydrogenase [NADP(+)] OS=Homo sapiens GN=AKR1A1 PE=1 SV=3	6	12	7	14	17	15	19

Q8N0Y7	PGAM4_HUMAN	28777	PGAM4	Probable phosphoglycerate mutase 4 OS=Homo sapiens GN=PGAM4 PE=2 SV=1	6	16	15	13	15	13	12
Q9Y240	CLC11_HUMAN	35695	CLEC11A	C-type lectin domain family 11 member A OS=Homo sapiens GN=CLEC11A PE=1 SV=1	6	10	9	18	17	14	16
Q9HBB2	Q9HBB2_HUMAN	87089	IRP1	Cytoplasmic aconitate hydratase OS=Homo sapiens GN=IRP1 PE=2 SV=1	6	15	18	13	15	12	11
P16152	CBR1_HUMAN	30375	CBR1	Carbonyl reductase [NADPH] 1 OS=Homo sapiens GN=CBR1 PE=1 SV=3	6	12	14	14	16	13	14
P61978-2	HNRPK_HUMAN	51028	HNRNPK	Isoform 2 of Heterogeneous nuclear ribonucleoprotein K OS=Homo sapiens GN=HNRNPK	6	19	18	9	9	10	18
Q01082	SPTB2_HUMAN	274609	SPTBN1	Spectrin beta chain, non-erythrocytic 1 OS=Homo sapiens GN=SPTBN1 PE=1 SV=2	6	4	3	26	33	8	9
P13489	RINI_HUMAN	49973	RNH1	Ribonuclease inhibitor OS=Homo sapiens GN=RNH1 PE=1 SV=2	6	10	14	15	12	7	24
P17661	DESM_HUMAN	53536	DES	Desmin OS=Homo sapiens GN=DES PE=1 SV=3	6	18	17	7	7	14	19
P49747	COMP_HUMAN	82860	COMP	Cartilage oligomeric matrix protein OS=Homo sapiens GN=COMP PE=1 SV=2	6	8	10	22	19	13	10
P00441	SODC_HUMAN	15936	SOD1	Superoxide dismutase [Cu-Zn] OS=Homo sapiens GN=SOD1 PE=1 SV=2	6	16	13	14	16	8	14
P25786-2	PSA1_HUMAN	30239	PSMA1	Isoform Long of Proteasome subunit alpha type-1 OS=Homo sapiens GN=PSMA1	6	11	9	20	21	9	11
P61160	ARP2_HUMAN	44761	ACTR2	Actin-related protein 2 OS=Homo sapiens GN=ACTR2 PE=1 SV=1	6	14	13	11	12	9	22
P78417	GSTO1_HUMAN	27566	GSTO1	Glutathione S-transferase omega-1 OS=Homo sapiens GN=GSTO1 PE=1 SV=2	6	16	21	11	11	10	12
Q13162	PRDX4_HUMAN	30540	PRDX4	Peroxiredoxin-4 OS=Homo sapiens GN=PRDX4 PE=1 SV=1	6	9	8	17	16	14	16
Q14240-2	IF4A2_HUMAN	46489	EIF4A2	Isoform 2 of Eukaryotic initiation factor 4A-II OS=Homo sapiens GN=EIF4A2	6	12	10	10	9	11	28
P49788	TIG1_HUMAN	33285	RARRES1	Retinoic acid receptor responder protein 1 OS=Homo sapiens GN=RARRES1 PE=1 SV=2	6	13	19	7	4	20	16
O14818	PSA7_HUMAN	27887	PSMA7	Proteasome subunit alpha type-7 OS=Homo sapiens GN=PSMA7 PE=1 SV=1	6	12	13	18	19	7	9
P80723	BASP1_HUMAN	22693	BASP1	Brain acid soluble protein 1 OS=Homo sapiens GN=BASP1 PE=1 SV=2	6	12	20	11	9	16	10
Q9H299	SH3L3_HUMAN	10438	SH3BGRL3	SH3 domain-binding glutamic acid-rich-like protein 3 OS=Homo sapiens GN=SH3BGRL3 PE=1 SV=1	6	14	18	10	13	13	10
Q9NR99	MXRA5_HUMAN	312150	MXRA5	Matrix-remodeling-associated protein 5 OS=Homo sapiens GN=MXRA5 PE=2 SV=3	6	9	11	3	2	28	25
P00747	PLMN_HUMAN	90569	PLG	Plasminogen OS=Homo sapiens GN=PLG PE=1 SV=2	6	8	9	19	16	12	13
P24043	LAMA2_HUMAN	343905	LAMA2	Laminin subunit alpha-2 OS=Homo sapiens GN=LAMA2 PE=1 SV=4	6	1	1	16	13	24	22
P28074	PSB5_HUMAN	28480	PSMB5	Proteasome subunit beta type-5 OS=Homo sapiens GN=PSMB5 PE=1 SV=3	6	9	12	17	22	9	8
Q32P28-3	P3H1_HUMAN	90616	LEPRE1	Isoform 3 of Prolyl 3-hydroxylase 1 OS=Homo sapiens GN=LEPRE1	6	12	11	9	11	14	20
P28066	PSA5_HUMAN	26411	PSMA5	Proteasome subunit alpha type-5 OS=Homo sapiens GN=PSMA5 PE=1 SV=3	6	13	17	17	16	8	5
Q05639	EF1A2_HUMAN	50470	EEF1A2	Elongation factor 1-alpha 2 OS=Homo sapiens GN=EEF1A2 PE=1 SV=1	6	11	12	9	11	17	16
Q15365	PCBP1_HUMAN	37498	PCBP1	Poly(rC)-binding protein 1 OS=Homo sapiens GN=PCBP1 PE=1 SV=2	6	11	14	11	14	11	15
G3V1N2	G3V1N2_HUMAN	11948	HBA2	HCG1745306, isoform CRA_a OS=Homo sapiens GN=HBA2 PE=2 SV=1	6	12	9	15	19	8	13
P15121	ALDR_HUMAN	35853	AKR1B1	Aldose reductase OS=Homo sapiens GN=AKR1B1 PE=1 SV=3	6	11	10	16	14	12	12
P49327	FAS_HUMAN	273427	FASN	Fatty acid synthase OS=Homo sapiens GN=FASN PE=1 SV=3	6	3	7	22	16	16	11
Q15818	NPTX1_HUMAN	47122	NPTX1	Neuronal pentraxin-1 OS=Homo sapiens GN=NPTX1 PE=2 SV=2	6	3	4	15	12	16	25
O60568	PLOD3_HUMAN	84785	PLOD3	Procollagen-lysine,2-oxoglutarate 5-dioxygenase 3 OS=Homo sapiens GN=PLOD3 PE=1 SV=1	6	13	11	13	10	14	13
P80162	CXCL6_HUMAN	11897	CXCL6	C-X-C motif chemokine 6 OS=Homo sapiens GN=CXCL6 PE=1 SV=4	6	19	17	3	2	17	16
Q86SR1-2	GLT10_HUMAN	61947	GALNT10	Isoform 2 of Polypeptide N-acetylgalactosaminyltransferase 10 OS=Homo sapiens GN=GALNT10	6	22	16	3	5	16	12
Q92743	HTRA1_HUMAN	51287	HTRA1	Serine protease HTRA1 OS=Homo sapiens GN=HTRA1 PE=1 SV=1	6	21	15	4	2	9	23
Q96KP4	CNDP2_HUMAN	52878	CNDP2	Cytosolic non-specific dipeptidase OS=Homo sapiens GN=CNDP2 PE=1 SV=2	6	6	8	14	11	12	23
Q9P2E7	PCD10_HUMAN	112936	PCDH10	Protocadherin-10 OS=Homo sapiens GN=PCDH10 PE=2 SV=2	6	16	17	6	6	15	14
O95084	PRS23_HUMAN	43001	PRSS23	Serine protease 23 OS=Homo sapiens GN=PRSS23 PE=1 SV=1	6	7	4	4	6	30	22
O95336	6PGL_HUMAN	27547	PGLS	6-phosphogluconolactonase OS=Homo sapiens GN=PGLS PE=1 SV=2	6	12	10	16	16	12	7
P04080	CYTB_HUMAN	11140	CSTB	Cystatin-B OS=Homo sapiens GN=CSTB PE=1 SV=2	6	11	15	8	10	12	17
P60900	PSA6_HUMAN	27399	PSMA6	Proteasome subunit alpha type-6 OS=Homo sapiens GN=PSMA6 PE=1 SV=1	6	11	11	21	19	7	4
P98095-2	FBLN2_HUMAN	131863	FBLN2	Isoform 2 of Fibulin-2 OS=Homo sapiens GN=FBLN2	6	20	15	4	6	16	12
P55145	MANF_HUMAN	20700	MANF	Mesencephalic astrocyte-derived neurotrophic factor OS=Homo sapiens GN=MANF PE=1 SV=3	6	10	11	12	9	17	13
P62942	FKB1A_HUMAN	11951	FKBP1A	Peptidyl-prolyl cis-trans isomerase FKBP1A OS=Homo sapiens GN=FKBP1A PE=1 SV=2	6	12	10	10	16	15	9
Q14847-2	LASP1_HUMAN	36014	LASP1	Isoform 2 of LIM and SH3 domain protein 1 OS=Homo sapiens GN=LASP1	6	9	13	9	10	15	16

O60664-4	PLIN3_HUMAN	45803	PLIN3	Isoform 4 of Perilipin-3 OS=Homo sapiens GN=PLIN3	6	13	11	6	8	11	22
P14174	MIF_HUMAN	12476	MIF	Macrophage migration inhibitory factor OS=Homo sapiens GN=MIF PE=1 SV=4	6	12	12	14	9	13	11
P61088	UBE2N_HUMAN	17138	UBE2N	Ubiquitin-conjugating enzyme E2 N OS=Homo sapiens GN=UBE2N PE=1 SV=1	6	17	17	7	9	10	11
P81605-2	DCD_HUMAN	12414	DCD	Isoform 2 of Dermcidin OS=Homo sapiens GN=DCD	6	10	9	14	17	8	13
Q16394	EXT1_HUMAN	86255	EXT1	Exostosin-1 OS=Homo sapiens GN=EXT1 PE=1 SV=2	6	13	15	8	10	15	9
Q9HC38-2	GLOD4_HUMAN	33233	GLOD4	Isoform 2 of Glyoxalase domain-containing protein 4 OS=Homo sapiens GN=GLOD4	6	8	8	17	17	12	8
M0QZ52	M0QZ52_HUMAN	9354	CALM3	Calmodulin OS=Homo sapiens GN=CALM3 PE=2 SV=1	6	12	19	8	11	7	13
O43396	TXNL1_HUMAN	32251	TXNL1	Thioredoxin-like protein 1 OS=Homo sapiens GN=TXNL1 PE=1 SV=3	6	10	6	19	19	9	6
P02786	TFR1_HUMAN	84871	TFRC	Transferrin receptor protein 1 OS=Homo sapiens GN=TFRC PE=1 SV=2	6	10	8	11	16	14	10
P08758	ANXA5_HUMAN	35937	ANXA5	Annexin A5 OS=Homo sapiens GN=ANXA5 PE=1 SV=2	6	6	6	20	21	8	8
P20618	PSB1_HUMAN	26489	PSMB1	Proteasome subunit beta type-1 OS=Homo sapiens GN=PSMB1 PE=1 SV=2	6	9	7	18	19	11	5
P25789	PSA4_HUMAN	29484	PSMA4	Proteasome subunit alpha type-4 OS=Homo sapiens GN=PSMA4 PE=1 SV=1	6	13	9	18	16	6	6
O15145	ARPC3_HUMAN	20547	ARPC3	Actin-related protein 2/3 complex subunit 3 OS=Homo sapiens GN=ARPC3 PE=1 SV=3	6	9	13	11	12	12	10
P00492	HPRT_HUMAN	24579	HPRT1	Hypoxanthine-guanine phosphoribosyltransferase OS=Homo sapiens GN=HPRT1 PE=1 SV=2	6	13	11	11	9	10	13
P04632	CPNS1_HUMAN	28316	CAPNS1	Calpain small subunit 1 OS=Homo sapiens GN=CAPNS1 PE=1 SV=1	6	8	6	19	12	14	8
P30040	ERP29_HUMAN	28993	ERP29	Endoplasmic reticulum resident protein 29 OS=Homo sapiens GN=ERP29 PE=1 SV=4	6	10	8	18	16	6	9
P31949	S10AB_HUMAN	11740	S100A11	Protein S100-A11 OS=Homo sapiens GN=S100A11 PE=1 SV=2	6	12	10	11	12	10	12
P09622	DLDH_HUMAN	54177	DLD	Dihydrolipoyl dehydrogenase, mitochondrial OS=Homo sapiens GN=DLD PE=1 SV=2	6	5	5	17	21	4	14
Q5D862	FILA2_HUMAN	248073	FLG2	Filaggrin-2 OS=Homo sapiens GN=FLG2 PE=1 SV=1	6	4	4	27	19	5	7
P07711	CATL1_HUMAN	37564	CTSL	Cathepsin L1 OS=Homo sapiens GN=CTSL PE=1 SV=2	6	11	9	5	3	12	25
P08697-2	A2AP_HUMAN	47907	SERPINF2	Isoform 2 of Alpha-2-antiplasmin OS=Homo sapiens GN=SERPINF2	6	4	5	18	17	12	9
P25398	RS12_HUMAN	14515	RPS12	40S ribosomal protein S12 OS=Homo sapiens GN=RPS12 PE=1 SV=3	6	13	13	7	5	16	11
P30508	1C12_HUMAN	40886	HLA-C	HLA class I histocompatibility antigen, Cw-12 alpha chain OS=Homo sapiens GN=HLA-C PE=2 SV=2	6	14	11	8	9	10	13
P48444	COPD_HUMAN	57210	ARCN1	Coatomer subunit delta OS=Homo sapiens GN=ARCN1 PE=1 SV=1	6	10	7	14	13	14	7
Q9P2E9-2	RRBP1_HUMAN	152189	RRBP1	Isoform 1 of Ribosome-binding protein 1 OS=Homo sapiens GN=RRBP1	6	10	6	17	15	8	9
P30046	DOPD_HUMAN	12712	DDT	D-dopachrome decarboxylase OS=Homo sapiens GN=DDT PE=1 SV=3	6	17	10	10	8	10	9
P39060-1	COIA1_HUMAN	154018	COL18A1	Isoform 2 of Collagen alpha-1(XVIII) chain OS=Homo sapiens GN=COL18A1	6	8	8	9	9	17	13
Q13443-2	ADAM9_HUMAN	72359	ADAM9	Isoform 2 of Disintegrin and metalloproteinase domain-containing protein 9 OS=Homo sapiens GN=ADAM9	5	16	15	1		15	17
P26641	EF1G_HUMAN	50119	EEF1G	Elongation factor 1-gamma OS=Homo sapiens GN=EEF1G PE=1 SV=3	5	7		17	14	13	13
Q04760-2	LGUL_HUMAN	19043	GLO1	Isoform 2 of Lactoylglutathione lyase OS=Homo sapiens GN=GLO1	6	10	11	13	14	8	7
P05156	CFAI_HUMAN	65750	CFI	Complement factor I OS=Homo sapiens GN=CFI PE=1 SV=2	6	12	8	4	4	14	20
P12004	PCNA_HUMAN	28769	PCNA	Proliferating cell nuclear antigen OS=Homo sapiens GN=PCNA PE=1 SV=1	6	6	5	14	19	7	11
P30085	KCY_HUMAN	22222	CMPK1	UMP-CMP kinase OS=Homo sapiens GN=CMPK1 PE=1 SV=3	6	11	8	14	13	10	6
Q14574-2	DSC3_HUMAN	93454	DSC3	Isoform 3B of Desmocollin-3 OS=Homo sapiens GN=DSC3	6	10	9	5	8	15	15
Q16543	CDC37_HUMAN	44468	CDC37	Hsp90 co-chaperone Cdc37 OS=Homo sapiens GN=CDC37 PE=1 SV=1	6	13	7	8	6	11	17
P00734	THRB_HUMAN	70037	F2	Prothrombin OS=Homo sapiens GN=F2 PE=1 SV=2	6	5	1	22	19	7	7
P08865	RSSA_HUMAN	32854	RPSA	40S ribosomal protein SA OS=Homo sapiens GN=RPSA PE=1 SV=4	6	7	5	14	15	9	11
P13796	PLSL_HUMAN	70288	LCP1	Plastin-2 OS=Homo sapiens GN=LCP1 PE=1 SV=6	6	15	15	5	10	8	8
P28070	PSB4_HUMAN	29204	PSMB4	Proteasome subunit beta type-4 OS=Homo sapiens GN=PSMB4 PE=1 SV=4	6	7	8	19	19	6	2
P49720	PSB3_HUMAN	22949	PSMB3	Proteasome subunit beta type-3 OS=Homo sapiens GN=PSMB3 PE=1 SV=2	6	10	13	14	15	6	3
Q9H013-2	ADA19_HUMAN	100848	ADAM19	Isoform B of Disintegrin and metalloproteinase domain-containing protein 19 OS=Homo sapiens GN=ADAM19	4	15	14			16	16
O43854-2	EDIL3_HUMAN	52748	EDIL3	Isoform 2 of EGF-like repeat and discoidin I-like domain-containing protein 3 OS=Homo sapiens GN=EDIL3	6	18	10	2	3	16	11
P10619	PPGB_HUMAN	54466	CTSA	Lysosomal protective protein OS=Homo sapiens GN=CTSA PE=1 SV=2	6	7	5	9	9	13	17
P61970	NTF2_HUMAN	14478	NUTF2	Nuclear transport factor 2 OS=Homo sapiens GN=NUTF2 PE=1 SV=1	6	4	4	14	15	13	10
Q14914-2	PTGR1_HUMAN	32895	PTGR1	Isoform 2 of Prostaglandin reductase 1 OS=Homo sapiens GN=PTGR1	6	8	2	14	13	11	12

Q9NYU2-2	UGGG1_HUMAN	174977	UGGT1	Isoform 2 of UDP-glucose:glycoprotein glucosyltransferase 1 OS=Homo sapiens GN=UGGT1	6	6	4	7	7	20	16
P01009	A1AT_HUMAN	46737	SERPINA1	Alpha-1-antitrypsin OS=Homo sapiens GN=SERPINA1 PE=1 SV=3	6	6	7	18	8	9	11
Q9H4B7	TBB1_HUMAN	50327	TUBB1	Tubulin beta-1 chain OS=Homo sapiens GN=TUBB1 PE=1 SV=1	6	11	11	6	6	10	15
Q9UQ80	PA2G4_HUMAN	43787	PA2G4	Proliferation-associated protein 2G4 OS=Homo sapiens GN=PA2G4 PE=1 SV=3	6	8	7	10	8	9	17
Q06033-2	ITIH3_HUMAN	99328	ITIH3	Isoform 2 of Inter-alpha-trypsin inhibitor heavy chain H3 OS=Homo sapiens GN=ITIH3	6	5	4	19	22	3	5
Q07812-2	BAX_HUMAN	24220	BAX	Isoform Beta of Apoptosis regulator BAX OS=Homo sapiens GN=BAX	6	14	13	6	6	10	9
Q12765-2	SCRN1_HUMAN	48714	SCRN1	Isoform 2 of Secernin-1 OS=Homo sapiens GN=SCRN1	6	7	8	11	18	5	9
Q15181	IPYR_HUMAN	32660	PPA1	Inorganic pyrophosphatase OS=Homo sapiens GN=PPA1 PE=1 SV=2	6	7	9	7	10	12	13
Q9UNW1	MINP1_HUMAN	55051	MINPP1	Multiple inositol polyphosphate phosphatase 1 OS=Homo sapiens GN=MINPP1 PE=1 SV=1	6	9	11	3	4	12	19
A6NHL2-2	TBAL3_HUMAN	45518	TUBAL3	Isoform 2 of Tubulin alpha chain-like 3 OS=Homo sapiens GN=TUBAL3	6	12	7	9	7	9	13
O14579	COPE_HUMAN	34482	COPE	Coatomer subunit epsilon OS=Homo sapiens GN=COPE PE=1 SV=3	6	8	11	10	12	8	8
O15511	ARPC5_HUMAN	16320	ARPC5	Actin-related protein 2/3 complex subunit 5 OS=Homo sapiens GN=ARPC5 PE=1 SV=3	6	10	11	10	8	10	8
P04179	SODM_HUMAN	24722	SOD2	Superoxide dismutase [Mn], mitochondrial OS=Homo sapiens GN=SOD2 PE=1 SV=2	6	14	15	5	15	4	4
P05067-8	A4_HUMAN	84819	APP	Isoform APP751 of Amyloid beta A4 protein OS=Homo sapiens GN=APP	6	8	10	9	8	13	9
P07954-2	FUMH_HUMAN	50213	FH	Isoform Cytoplasmic of Fumarate hydratase, mitochondrial OS=Homo sapiens GN=FH	6	7	6	16	14	3	11
P62633-2	CNBP_HUMAN	18742	CNBP	Isoform 2 of Cellular nucleic acid-binding protein OS=Homo sapiens GN=CNBP	6	9	11	10	9	8	10
Q15691	MARE1_HUMAN	29999	MAPRE1	Microtubule-associated protein RP/EB family member 1 OS=Homo sapiens GN=MAPRE1 PE=1 SV=3	6	10	9	9	10	9	10
O94907	DKK1_HUMAN	28672	DKK1	Dickkopf-related protein 1 OS=Homo sapiens GN=DKK1 PE=1 SV=1	6	4	5	7	5	16	19
P49746	TSP3_HUMAN	104201	THBS3	Thrombospondin-3 OS=Homo sapiens GN=THBS3 PE=2 SV=1	6	11	14	5	8	7	10
P62805	H4_HUMAN	11367	HIST1H4A	Histone H4 OS=Homo sapiens GN=HIST1H4A PE=1 SV=2	6	9	6	8	8	11	13
P07205	PGK2_HUMAN	44796	PGK2	Phosphoglycerate kinase 2 OS=Homo sapiens GN=PGK2 PE=1 SV=3	6	7	2	10	11	8	16
P14324-2	FPPS_HUMAN	40532	FDPS	Isoform 2 of Farnesyl pyrophosphate synthase OS=Homo sapiens GN=FDPS	6	10	12	5	6	4	17
P15144	AMPN_HUMAN	109540	ANPEP	Aminopeptidase N OS=Homo sapiens GN=ANPEP PE=1 SV=4	6	6	9	13	12	7	7
P17948-3	VGFR1_HUMAN	82124	FLT1	Isoform 3 of Vascular endothelial growth factor receptor 1 OS=Homo sapiens GN=FLT1	6	12	15	4	4	8	11
Q02413	DSG1_HUMAN	113748	DSG1	Desmoglein-1 OS=Homo sapiens GN=DSG1 PE=1 SV=2	6	2	1	21	15	8	7
Q12907	LMAN2_HUMAN	40229	LMAN2	Vesicular integral-membrane protein VIP36 OS=Homo sapiens GN=LMAN2 PE=1 SV=1	6	11	15	4	7	8	9
Q31612	1B73_HUMAN	40435	HLA-B	HLA class I histocompatibility antigen, B-73 alpha chain OS=Homo sapiens GN=HLA-B PE=1 SV=1	6	10	7	8	8	8	13
Q86UD1	OAF_HUMAN	30688	OAF	Out at first protein homolog OS=Homo sapiens GN=OAF PE=2 SV=1	6	8	6	4	5	13	18
Q9NY33-4	DPP3_HUMAN	79307	DPP3	Isoform 4 of Dipeptidyl peptidase 3 OS=Homo sapiens GN=DPP3	6	6	9	11	17	6	5
Q9UI15	TAGL3_HUMAN	22473	TAGLN3	Transgelin-3 OS=Homo sapiens GN=TAGLN3 PE=1 SV=2	6	13	11	10	10	6	4
P05452	TETN_HUMAN	22537	CLEC3B	Tetranectin OS=Homo sapiens GN=CLEC3B PE=1 SV=3	6	5	6	16	13	5	8
P05556	ITB1_HUMAN	88415	ITGB1	Integrin beta-1 OS=Homo sapiens GN=ITGB1 PE=1 SV=2	6	7	10	11	9	9	7
P09455	RET1_HUMAN	15850	RBP1	Retinol-binding protein 1 OS=Homo sapiens GN=RBP1 PE=1 SV=2	6	15	12	6	6	6	8
P10124	SRGN_HUMAN	17652	SRGN	Serglycin OS=Homo sapiens GN=SRGN PE=1 SV=3	6	14	17	1	4	5	12
P15924	DESP_HUMAN	331774	DSP	Desmoplakin OS=Homo sapiens GN=DSP PE=1 SV=3	6	2	2	17	23	5	4
P20700	LMNB1_HUMAN	66408	LMNB1	Lamin-B1 OS=Homo sapiens GN=LMNB1 PE=1 SV=2	6	8	8	8	12	10	7
Q15907	RB11B_HUMAN	24489	RAB11B	Ras-related protein Rab-11B OS=Homo sapiens GN=RAB11B PE=1 SV=4	6	7	8	11	11	11	5
Q8WVQ1	CANT1_HUMAN	44840	CANT1	Soluble calcium-activated nucleotidase 1 OS=Homo sapiens GN=CANT1 PE=1 SV=1	6	10	4	6	5	11	17
Q9UKU6	TRHDE_HUMAN	117000	TRHDE	Thyrotropin-releasing hormone-degrading ectoenzyme OS=Homo sapiens GN=TRHDE PE=2 SV=1	6	12	12	7	7	8	7
P31947-2	1433S_HUMAN	24336	SFN	Isoform 2 of 14-3-3 protein sigma OS=Homo sapiens GN=SFN	6	9	8	8	8	9	10
P33316-2	DUT_HUMAN	17748	DUT	Isoform 2 of Deoxyuridine 5'-triphosphate nucleotidohydrolase, mitochondrial	6	10	9	8	6	9	10

				OS=Homo sapiens GN=DUT							
P61204	ARF3_HUMAN	20601	ARF3	ADP-ribosylation factor 3 OS=Homo sapiens GN=ARF3 PE=1 SV=2	6	12	8	6	5	10	11
P63244	GBLP_HUMAN	35077	GNB2L1	Guanine nucleotide-binding protein subunit beta-2-like 1 OS=Homo sapiens GN=GNB2L1 PE=1 SV=3	6	6	8	10	10	11	7
P84077	ARF1_HUMAN	20697	ARF1	ADP-ribosylation factor 1 OS=Homo sapiens GN=ARF1 PE=1 SV=2	6	12	8	6	5	10	11
Q15819	UB2V2_HUMAN	16363	UBE2V2	Ubiquitin-conjugating enzyme E2 variant 2 OS=Homo sapiens GN=UBE2V2 PE=1 SV=4	6	8	12	6	7	9	10
Q8NC51-3	PAIRB_HUMAN	43135	SERBP1	Isoform 3 of Plasminogen activator inhibitor 1 RNA-binding protein OS=Homo sapiens GN=SERBP1	6	7	12	5	7	8	13
Q92520	FAM3C_HUMAN	24680	FAM3C	Protein FAM3C OS=Homo sapiens GN=FAM3C PE=1 SV=1	6	11	12	6	6	7	10
A6NE09	A6NE09_HUMAN	32909	RPSAP58	40S ribosomal protein SA OS=Homo sapiens GN=RPSAP58 PE=2 SV=1	6	7	5	10	12	8	10
Q96D15	RCN3_HUMAN	37493	RCN3	Reticulocalbin-3 OS=Homo sapiens GN=RCN3 PE=1 SV=1	6	9	7	9	8	4	14
E9PR17	E9PR17_HUMAN	14529	CD59	CD59 glycoprotein OS=Homo sapiens GN=CD59 PE=2 SV=1	6	12	6	8	10	6	9
O00410	IPO5_HUMAN	123630	IPO5	Importin-5 OS=Homo sapiens GN=IPO5 PE=1 SV=4	6	7	6	10	9	12	6
P01893	HLAH_HUMAN	40892	HLA-H	Putative HLA class I histocompatibility antigen, alpha chain H OS=Homo sapiens GN=HLA-H PE=5 SV=3	6	10	7	7	7	8	11
P07686	HEXB_HUMAN	63111	HEXB	Beta-hexosaminidase subunit beta OS=Homo sapiens GN=HEXB PE=1 SV=3	6	11	4	10	10	9	6
P62491-2	RB11A_HUMAN	17659	RAB11A	Isoform 2 of Ras-related protein Rab-11A OS=Homo sapiens GN=RAB11A	6	7	8	10	10	10	5
Q03405-3	UPAR_HUMAN	32016	PLAUR	Isoform 3 of Urokinase plasminogen activator surface receptor OS=Homo sapiens GN=PLAUR	6	10	7	7	5	10	11
Q15631	TSN_HUMAN	26183	TSN	Translin OS=Homo sapiens GN=TSN PE=1 SV=1	6	8	6	11	12	7	6
P20809	IL11_HUMAN	21429	IL11	Interleukin-11 OS=Homo sapiens GN=IL11 PE=1 SV=1	4	20	15			9	6
P19021-3	AMD_HUMAN	100818	PAM	Isoform 3 of Peptidyl-glycine alpha-amidating monooxygenase OS=Homo sapiens GN=PAM	4			2	1	24	23
P12955	PEPD_HUMAN	54548	PEPD	Xaa-Pro dipeptidase OS=Homo sapiens GN=PEPD PE=1 SV=3	6	8	3	14	9	3	12
P30048	PRDX3_HUMAN	27693	PRDX3	Thioredoxin-dependent peroxide reductase, mitochondrial OS=Homo sapiens GN=PRDX3 PE=1 SV=3	6	7	6	9	15	8	4
P35749-2	MYH11_HUMAN	228090	MYH11	Isoform 2 of Myosin-11 OS=Homo sapiens GN=MYH11	6	6	7	11	9	9	7
P46821	MAP1B_HUMAN	270634	MAP1B	Microtubule-associated protein 1B OS=Homo sapiens GN=MAP1B PE=1 SV=2	6	9	6	7	8	12	7
Q15366-2	PCBP2_HUMAN	38651	PCBP2	Isoform 2 of Poly(rC)-binding protein 2 OS=Homo sapiens GN=PCBP2	6	7	11	7	7	9	8
A6NEC2	PSAL_HUMAN	53747	NPEPPSL1	Puromycin-sensitive aminopeptidase-like protein OS=Homo sapiens GN=NPEPPSL1 PE=2 SV=3	6	9	9	11	10	4	5
O43505	B3GN1_HUMAN	47119	B3GNT1	N-acetyllactosaminide beta-1,3-N-acetylglucosaminyltransferase OS=Homo sapiens GN=B3GNT1 PE=1 SV=1	6	8	3	6	6	10	15
P02795	MT2_HUMAN	6042	MT2A	Metallothionein-2 OS=Homo sapiens GN=MT2A PE=1 SV=1	6	3	5	6	8	12	14
P08729	K2C7_HUMAN	51386	KRT7	Keratin, type II cytoskeletal 7 OS=Homo sapiens GN=KRT7 PE=1 SV=5	6	6	12	10	9	5	6
P35580-2	MYH10_HUMAN	230779	MYH10	Isoform 2 of Myosin-10 OS=Homo sapiens GN=MYH10	6	6	9	12	8	10	3
Q02790	FKBP4_HUMAN	51805	FKBP4	Peptidyl-prolyl cis-trans isomerase FKBP4 OS=Homo sapiens GN=FKBP4 PE=1 SV=3	6	8	5	10	9	6	10
Q14974	IMB1_HUMAN	97170	KPNB1	Importin subunit beta-1 OS=Homo sapiens GN=KPNB1 PE=1 SV=2	6	9	7	12	10	5	5
Q58FF7	H90B3_HUMAN	68325	HSP90AB3P	Putative heat shock protein HSP 90-beta-3 OS=Homo sapiens GN=HSP90AB3P PE=5 SV=1	6	11	15	7	5	5	5
Q8NBP7	PCSK9_HUMAN	74286	PCSK9	Proprotein convertase subtilisin/kexin type 9 OS=Homo sapiens GN=PCSK9 PE=1 SV=3	6	12	10	1	3	11	11
Q9NSB2	KRT84_HUMAN	64842	KRT84	Keratin, type II cuticular Hb4 OS=Homo sapiens GN=KRT84 PE=2 SV=2	6	6	12	10	9	5	6
Q9NZ08-2	ERAP1_HUMAN	107841	ERAP1	Isoform 2 of Endoplasmic reticulum aminopeptidase 1 OS=Homo sapiens GN=ERAP1	6	15	17	4	5	3	4
E5RJ24	E5RJ24_HUMAN	53145	LOC100510707	Protein LOC100510707 (Fragment) OS=Homo sapiens GN=LOC100510707 PE=2 SV=1	6	9	9	11	10	4	5
O15031	PLXB2_HUMAN	205127	PLXNB2	Plexin-B2 OS=Homo sapiens GN=PLXNB2 PE=1 SV=3	5	10	12	3		12	11
P48307	TFPI2_HUMAN	26934	TFPI2	Tissue factor pathway inhibitor 2 OS=Homo sapiens GN=TFPI2 PE=1 SV=1	5	17	18	3		6	4
Q15904	VAS1_HUMAN	52026	ATP6AP1	V-type proton ATPase subunit S1 OS=Homo sapiens GN=ATP6AP1 PE=1 SV=2	5	11	14		1	9	13
P31153	METK2_HUMAN	43661	MAT2A	S-adenosylmethionine synthase isoform type-2 OS=Homo sapiens GN=MAT2A PE=1 SV=1	5	6		8	12	7	15

Q9H1B5	XYLT2_HUMAN	96767	XYLT2	Xylosyltransferase 2 OS=Homo sapiens GN=XYLT2 PE=2 SV=2	5	4		7	7	17	13
P00750	TPA_HUMAN	62917	PLAT	Tissue-type plasminogen activator OS=Homo sapiens GN=PLAT PE=1 SV=1	6	2	2	13	12	9	9
P22061-2	PIMT_HUMAN	24679	PCMT1	Isoform 2 of Protein-L-isoaspartate(D-aspartate) O-methyltransferase OS=Homo sapiens GN=PCMT1	6	7	9	9	8	9	5
P29400-2	CO4A5_HUMAN	161632	COL4A5	Isoform 2 of Collagen alpha-5(IV) chain OS=Homo sapiens GN=COL4A5	6	19	12	2	1	9	4
Q9H6X2-2	ANTR1_HUMAN	41157	ANTXR1	Isoform 2 of Anthrax toxin receptor 1 OS=Homo sapiens GN=ANTXR1	6	5	5	4	3	12	18
Q9Y266	NUDC_HUMAN	38243	NUDC	Nuclear migration protein nudC OS=Homo sapiens GN=NUDC PE=1 SV=1	6	10	8	6	11	2	10
A6NHG4	DDTL_HUMAN	14195	DDTL	D-dopachrome decarboxylase-like protein OS=Homo sapiens GN=DDTL PE=2 SV=1	6	14	7	7	5	7	6
O15067	PUR4_HUMAN	144734	PFAS	Phosphoribosylformylglycinamidine synthase OS=Homo sapiens GN=PFAS PE=1 SV=4	6	8	7	9	12	7	3
P06748-2	NPM_HUMAN	29465	NPM1	Isoform 2 of Nucleophosmin OS=Homo sapiens GN=NPM1	6	6	2	12	10	8	8
P0C0S8	H2A1_HUMAN	14091	HIST1H2AG	Histone H2A type 1 OS=Homo sapiens GN=HIST1H2AG PE=1 SV=2	6	10	10	6	6	7	7
P23396-2	RS3_HUMAN	28487	RPS3	Isoform 2 of 40S ribosomal protein S3 OS=Homo sapiens GN=RPS3	6	7	9	9	11	6	4
P30044-2	PRDX5_HUMAN	17031	PRDX5	Isoform Cytoplasmic+peroxisomal of Peroxiredoxin-5, mitochondrial OS=Homo sapiens GN=PRDX5	6	12	10	2	3	8	11
Q06323	PSME1_HUMAN	28723	PSME1	Proteasome activator complex subunit 1 OS=Homo sapiens GN=PSME1 PE=1 SV=1	6	9	11	8	8	5	5
Q14CN4-2	K2C72_HUMAN	55749	KRT72	Isoform 2 of Keratin, type II cytoskeletal 72 OS=Homo sapiens GN=KRT72	6	4	10	10	11	6	5
Q16777	H2A2C_HUMAN	13988	HIST2H2AC	Histone H2A type 2-C OS=Homo sapiens GN=HIST2H2AC PE=1 SV=4	6	10	10	6	6	7	7
Q6FI13	H2A2A_HUMAN	14095	HIST2H2AA3	Histone H2A type 2-A OS=Homo sapiens GN=HIST2H2AA3 PE=1 SV=3	6	10	10	6	6	7	7
Q93077	H2A1C_HUMAN	14105	HIST1H2AC	Histone H2A type 1-C OS=Homo sapiens GN=HIST1H2AC PE=1 SV=3	6	10	10	6	6	7	7
Q96KK5	H2A1H_HUMAN	13906	HIST1H2AH	Histone H2A type 1-H OS=Homo sapiens GN=HIST1H2AH PE=1 SV=3	6	10	10	6	6	7	7
Q99878	H2A1J_HUMAN	13936	HIST1H2AJ	Histone H2A type 1-J OS=Homo sapiens GN=HIST1H2AJ PE=1 SV=3	6	10	10	6	6	7	7
Q9BTM1	H2AJ_HUMAN	14019	H2AFJ	Histone H2A.J OS=Homo sapiens GN=H2AFJ PE=1 SV=1	6	10	10	6	6	7	7
Q9Y5S9	RBM8A_HUMAN	19889	RBM8A	RNA-binding protein 8A OS=Homo sapiens GN=RBM8A PE=1 SV=1	6	8	8	5	6	11	8
O00339-2	MATN2_HUMAN	104775	MATN2	Isoform 2 of Matrilin-2 OS=Homo sapiens GN=MATN2	6	4	6	8	3	12	12
O60814	H2B1K_HUMAN	13890	HIST1H2BK	Histone H2B type 1-K OS=Homo sapiens GN=HIST1H2BK PE=1 SV=3	6	7	9	9	7	6	7
P04156-2	PRIO_HUMAN	26885	PRNP	Isoform 2 of Major prion protein OS=Homo sapiens GN=PRNP	6	10	14	8	5	4	4
P04908	H2A1B_HUMAN	14135	HIST1H2AB	Histone H2A type 1-B/E OS=Homo sapiens GN=HIST1H2AB PE=1 SV=2	6	9	10	6	6	7	7
P20671	H2A1D_HUMAN	14107	HIST1H2AD	Histone H2A type 1-D OS=Homo sapiens GN=HIST1H2AD PE=1 SV=2	6	9	10	6	6	7	7
P24534	EF1B_HUMAN	24764	EEF1B2	Elongation factor 1-beta OS=Homo sapiens GN=EEF1B2 PE=1 SV=3	6	9	11	5	6	5	9
P49721	PSB2_HUMAN	22836	PSMB2	Proteasome subunit beta type-2 OS=Homo sapiens GN=PSMB2 PE=1 SV=1	6	5	7	13	13	4	3
P57053	H2BFS_HUMAN	13944	H2BFS	Histone H2B type F-S OS=Homo sapiens GN=H2BFS PE=1 SV=2	6	7	9	9	7	6	7
P58876	H2B1D_HUMAN	13936	HIST1H2BD	Histone H2B type 1-D OS=Homo sapiens GN=HIST1H2BD PE=1 SV=2	6	7	9	9	7	6	7
P62807	H2B1C_HUMAN	13906	HIST1H2BC	Histone H2B type 1-C/E/F/G/I OS=Homo sapiens GN=HIST1H2BC PE=1 SV=4	6	7	9	9	7	6	7
P62826	RAN_HUMAN	24423	RAN	GTP-binding nuclear protein Ran OS=Homo sapiens GN=RAN PE=1 SV=3	6	10	12	7	4	6	6
Q15293	RCN1_HUMAN	38890	RCN1	Reticulocalbin-1 OS=Homo sapiens GN=RCN1 PE=1 SV=1	6	3	5	9	9	6	13
Q5QNW6-2	H2B2F_HUMAN	14841	HIST2H2BF	Isoform 2 of Histone H2B type 2-F OS=Homo sapiens GN=HIST2H2BF	6	7	9	9	7	6	7
Q7L7L0	H2A3_HUMAN	14121	HIST3H2A	Histone H2A type 3 OS=Homo sapiens GN=HIST3H2A PE=1 SV=3	6	9	10	6	6	7	7
Q86Y46	K2C73_HUMAN	58923	KRT73	Keratin, type II cytoskeletal 73 OS=Homo sapiens GN=KRT73 PE=1 SV=1	6	3	7	12	12	5	6
Q93079	H2B1H_HUMAN	13892	HIST1H2BH	Histone H2B type 1-H OS=Homo sapiens GN=HIST1H2BH PE=1 SV=3	6	7	9	9	7	6	7
Q99877	H2B1N_HUMAN	13922	HIST1H2BN	Histone H2B type 1-N OS=Homo sapiens GN=HIST1H2BN PE=1 SV=3	6	7	9	9	7	6	7
Q99879	H2B1M_HUMAN	13989	HIST1H2BM	Histone H2B type 1-M OS=Homo sapiens GN=HIST1H2BM PE=1 SV=3	6	7	9	9	7	6	7
Q99880	H2B1L_HUMAN	13952	HIST1H2BL	Histone H2B type 1-L OS=Homo sapiens GN=HIST1H2BL PE=1 SV=3	6	7	9	9	7	6	7
Q9UNN8	EPCR_HUMAN	26671	PROCR	Endothelial protein C receptor OS=Homo sapiens GN=PROCR PE=1 SV=1	6	7	5	5	7	10	11
O14672	ADA10_HUMAN	84142	ADAM10	Disintegrin and metalloproteinase domain-containing protein 10 OS=Homo sapiens GN=ADAM10 PE=1 SV=1	5	11	8	1		15	10
P01344-2	IGF2_HUMAN	20477	IGF2	Isoform 2 of Insulin-like growth factor II OS=Homo sapiens GN=IGF2	6	7	5	7	7	7	11
P38159	RBMX_HUMAN	42332	RBMX	RNA-binding motif protein, X chromosome OS=Homo sapiens GN=RBMX PE=1 SV=3	6	9	9	8	7	5	6
P63279	UBC9_HUMAN	18007	UBE2I	SUMO-conjugating enzyme UBC9 OS=Homo sapiens GN=UBE2I PE=1 SV=1	6	6	4	5	11	9	9
Q14195-2	DPYL3_HUMAN	73911	DPYSL3	Isoform LCRMP-4 of Dihydropyrimidinase-related protein 3 OS=Homo sapiens GN=DPYSL3	6	12	11	4	3	9	5

Q9BS26	ERP44_HUMAN	46971	ERP44	Endoplasmic reticulum resident protein 44 OS=Homo sapiens GN=ERP44 PE=1 SV=1	6	7	9	2	4	8	14
Q9BTY2	FUCO2_HUMAN	54067	FUCA2	Plasma alpha-L-fucosidase OS=Homo sapiens GN=FUCA2 PE=1 SV=2	5	14	9		1	10	10
P10253	LYAG_HUMAN	105324	GAA	Lysosomal alpha-glucosidase OS=Homo sapiens GN=GAA PE=1 SV=4	4	8	19			8	9
P05543	THBG_HUMAN	46325	SERPINA7	Thyroxine-binding globulin OS=Homo sapiens GN=SERPINA7 PE=1 SV=2	6	7	5	9	10	5	7
P09960	LKHA4_HUMAN	69285	LTA4H	Leukotriene A-4 hydrolase OS=Homo sapiens GN=LTA4H PE=1 SV=2	6	8	6	8	7	11	3
P25787	PSA2_HUMAN	25899	PSMA2	Proteasome subunit alpha type-2 OS=Homo sapiens GN=PSMA2 PE=1 SV=2	6	5	4	13	13	5	3
P25788-2	PSA3_HUMAN	27647	PSMA3	Isoform 2 of Proteasome subunit alpha type-3 OS=Homo sapiens GN=PSMA3	6	6	5	13	10	4	5
P63220	RS21_HUMAN	9111	RPS21	40S ribosomal protein S21 OS=Homo sapiens GN=RPS21 PE=1 SV=1	6	5	4	8	7	10	9
Q03252	LMNB2_HUMAN	67689	LMNB2	Lamin-B2 OS=Homo sapiens GN=LMNB2 PE=1 SV=3	6	7	7	7	9	7	6
Q12792	TWF1_HUMAN	40283	TWF1	Twinfilin-1 OS=Homo sapiens GN=TWF1 PE=1 SV=3	6	5	6	9	7	5	11
Q7RTS7	K2C74_HUMAN	57865	KRT74	Keratin, type II cytoskeletal 74 OS=Homo sapiens GN=KRT74 PE=1 SV=2	6	4	7	10	12	5	5
Q96FW1	OTUB1_HUMAN	31284	OTUB1	Ubiquitin thioesterase OTUB1 OS=Homo sapiens GN=OTUB1 PE=1 SV=2	6	6	4	11	12	6	4
P48740-2	MASP1_HUMAN	81860	MASP1	Isoform 2 of Mannan-binding lectin serine protease 1 OS=Homo sapiens GN=MASP1	5	4	6		1	15	17
O95865	DDAH2_HUMAN	29644	DDAH2	N(G),N(G)-dimethylarginine dimethylaminohydrolase 2 OS=Homo sapiens GN=DDAH2 PE=1 SV=1	6	3	5	11	8	9	6
P16930	FAAA_HUMAN	46374	FAH	Fumarylacetoacetase OS=Homo sapiens GN=FAH PE=1 SV=2	6	5	3	10	12	5	7
Q07021	C1QBP_HUMAN	31362	C1QBP	Complement component 1 Q subcomponent-binding protein, mitochondrial OS=Homo sapiens GN=C1QBP PE=1 SV=1	6	7	6	10	10	4	5
Q3SY84	K2C71_HUMAN	57292	KRT71	Keratin, type II cytoskeletal 71 OS=Homo sapiens GN=KRT71 PE=1 SV=3	6	3	7	11	10	5	6
Q7Z7M0	MEGF8_HUMAN	303100	MEGF8	Multiple epidermal growth factor-like domains protein 8 OS=Homo sapiens GN=MEGF8 PE=1 SV=2	6	4	6	3	2	17	10
Q9UJJ9	GNPTG_HUMAN	33974	GNPTG	N-acetylglucosamine-1-phosphotransferase subunit gamma OS=Homo sapiens GN=GNPTG PE=1 SV=1	5	7	5		1	9	20
P16949	STMN1_HUMAN	17303	STMN1	Stathmin OS=Homo sapiens GN=STMN1 PE=1 SV=3	4	12	13			7	10
O94760	DDAH1_HUMAN	31122	DDAH1	N(G),N(G)-dimethylarginine dimethylaminohydrolase 1 OS=Homo sapiens GN=DDAH1 PE=1 SV=3	6	3	1	12	11	6	8
P56537	IF6_HUMAN	26599	EIF6	Eukaryotic translation initiation factor 6 OS=Homo sapiens GN=EIF6 PE=1 SV=1	6	5	3	11	10	6	6
Q00688	FKBP3_HUMAN	25177	FKBP3	Peptidyl-prolyl cis-trans isomerase FKBP3 OS=Homo sapiens GN=FKBP3 PE=1 SV=1	6	8	8	8	3	7	7
Q15121	PEA15_HUMAN	15040	PEA15	Astrocytic phosphoprotein PEA-15 OS=Homo sapiens GN=PEA15 PE=1 SV=2	6	3	8	5	7	9	9
Q16531	DDB1_HUMAN	126968	DDB1	DNA damage-binding protein 1 OS=Homo sapiens GN=DDB1 PE=1 SV=1	6	2	5	15	13	5	1
Q99729-3	ROAA_HUMAN	30588	HNRNPAB	Isoform 3 of Heterogeneous nuclear ribonucleoprotein A/B OS=Homo sapiens GN=HNRNPAB	6	7	3	7	9	3	12
Q9H4A4	AMPB_HUMAN	72596	RNPEP	Aminopeptidase B OS=Homo sapiens GN=RNPEP PE=1 SV=2	6	9	6	6	5	10	5
Q9Y5Z4	HEBP2_HUMAN	22875	HEBP2	Heme-binding protein 2 OS=Homo sapiens GN=HEBP2 PE=1 SV=1	6	8	9	8	8	3	5
O43184-2	ADA12_HUMAN	80403	ADAM12	Isoform 2 of Disintegrin and metalloproteinase domain-containing protein 12 OS=Homo sapiens GN=ADAM12	5	10	15		2	7	7
P06865	HEXA_HUMAN	60703	HEXA	Beta-hexosaminidase subunit alpha OS=Homo sapiens GN=HEXA PE=1 SV=2	6	9	8	2	2	13	6
P18085	ARF4_HUMAN	20511	ARF4	ADP-ribosylation factor 4 OS=Homo sapiens GN=ARF4 PE=1 SV=3	6	8	6	6	6	7	7
P40121-2	CAPG_HUMAN	36857	CAPG	Isoform 2 of Macrophage-capping protein OS=Homo sapiens GN=CAPG	6	5	3	7	8	5	12
P46926	GNPI1_HUMAN	32669	GNPDA1	Glucosamine-6-phosphate isomerase 1 OS=Homo sapiens GN=GNPDA1 PE=1 SV=1	6	5	5	11	11	5	3
P52823	STC1_HUMAN	27621	STC1	Stanniocalcin-1 OS=Homo sapiens GN=STC1 PE=1 SV=1	6	8	8	3	3	11	7
P54819-2	KAD2_HUMAN	25615	AK2	Isoform 2 of Adenylate kinase 2, mitochondrial OS=Homo sapiens GN=AK2	6	3	6	9	14	5	3
Q9BRF8	CPPED_HUMAN	35548	CPPED1	Calcineurin-like phosphoesterase domain-containing protein 1 OS=Homo sapiens GN=CPPED1 PE=1 SV=3	6	5	5	8	10	5	7
P00367	DHE3_HUMAN	61398	GLUD1	Glutamate dehydrogenase 1, mitochondrial OS=Homo sapiens GN=GLUD1 PE=1 SV=2	5	2		14	16	3	5
P05388	RLA0_HUMAN	34274	RPLP0	60S acidic ribosomal protein P0 OS=Homo sapiens GN=RPLP0 PE=1 SV=1	6	9	5	7	7	6	5
P06899	H2B1J_HUMAN	13904	HIST1H2BJ	Histone H2B type 1-J OS=Homo sapiens GN=HIST1H2BJ PE=1 SV=3	6	6	8	7	6	5	7
P07108	ACBP_HUMAN	10044	DBI	Acyl-CoA-binding protein OS=Homo sapiens GN=DBI PE=1 SV=2	6	7	5	8	8	5	6
P15374	UCHL3_HUMAN	26183	UCHL3	Ubiquitin carboxyl-terminal hydrolase isozyme L3 OS=Homo sapiens GN=UCHL3 PE=1 SV=1	6	5	6	9	8	6	5

P23527	H2B1O_HUMAN	13906	HIST1H2BO	Histone H2B type 1-O OS=Homo sapiens GN=HIST1H2BO PE=1 SV=3	6	6	8	7	6	5	7
P33778	H2B1B_HUMAN	13950	HIST1H2BB	Histone H2B type 1-B OS=Homo sapiens GN=HIST1H2BB PE=1 SV=2	6	6	8	7	6	5	7
P42830	CXCL5_HUMAN	11972	CXCL5	C-X-C motif chemokine 5 OS=Homo sapiens GN=CXCL5 PE=1 SV=1	6	12	10	1	2	6	8
P43251	BTD_HUMAN	61133	BTD	Biotinidase OS=Homo sapiens GN=BTD PE=1 SV=2	6	7	8	5	5	10	4
Q16778	H2B2E_HUMAN	13920	HIST2H2BE	Histone H2B type 2-E OS=Homo sapiens GN=HIST2H2BE PE=1 SV=3	6	6	8	7	6	5	7
Q8N257	H2B3B_HUMAN	13908	HIST3H2BB	Histone H2B type 3-B OS=Homo sapiens GN=HIST3H2BB PE=1 SV=3	6	6	8	7	6	5	7
Q8TAA3-2	PSA7L_HUMAN	23702	PSMA8	Isoform 2 of Proteasome subunit alpha type-7-like OS=Homo sapiens GN=PSMA8	6	5	7	10	9	4	4
Q99439	CNN2_HUMAN	33697	CNN2	Calponin-2 OS=Homo sapiens GN=CNN2 PE=1 SV=4	6	7	9	5	3	9	6
Q9NUQ9	FA49B_HUMAN	36748	FAM49B	Protein FAM49B OS=Homo sapiens GN=FAM49B PE=1 SV=1	6	8	5	5	7	8	6
Q9UHY7	ENOPH_HUMAN	28933	ENOPH1	Enolase-phosphatase E1 OS=Homo sapiens GN=ENOPH1 PE=1 SV=1	6	4	4	11	8	7	5
Q9UKK9	NUDT5_HUMAN	24328	NUDT5	ADP-sugar pyrophosphatase OS=Homo sapiens GN=NUDT5 PE=1 SV=1	6	2	2	10	11	7	7
Q9Y4L1	HYOU1_HUMAN	111335	HYOU1	Hypoxia up-regulated protein 1 OS=Homo sapiens GN=HYOU1 PE=1 SV=1	6	6	6	4	2	13	8
O43155	FLRT2_HUMAN	74049	FLRT2	Leucine-rich repeat transmembrane protein FLRT2 OS=Homo sapiens GN=FLRT2 PE=1 SV=1	6	7	5	5	5	8	8
P35754	GLRX1_HUMAN	11776	GLRX	Glutaredoxin-1 OS=Homo sapiens GN=GLRX PE=1 SV=2	6	11	8	4	4	6	5
P54687	BCAT1_HUMAN	42966	BCAT1	Branched-chain-amino-acid aminotransferase, cytosolic OS=Homo sapiens GN=BCAT1 PE=1 SV=3	6	6	2	5	9	7	9
Q16555-2	DPYL2_HUMAN	58163	DPYSL2	Isoform 2 of Dihydropyrimidinase-related protein 2 OS=Homo sapiens GN=DPYSL2	6	4	4	13	9	5	3
Q9NZV1	CRIM1_HUMAN	113738	CRIM1	Cysteine-rich motor neuron 1 protein OS=Homo sapiens GN=CRIM1 PE=1 SV=1	6	8	7	4	3	4	12
Q9Y237-2	PIN4_HUMAN	16608	PIN4	Isoform 2 of Peptidyl-prolyl cis-trans isomerase NIMA-interacting 4 OS=Homo sapiens GN=PIN4	6	7	4	5	7	8	7
D3YTG3	D3YTG3_HUMAN	195324	ABI3BP	Target of Nesh-SH3 OS=Homo sapiens GN=ABI3BP PE=2 SV=1	6	5	3	9	9	8	4
P07910-2	HNRPC_HUMAN	32338	HNRNPC	Isoform C1 of Heterogeneous nuclear ribonucleoproteins C1/C2 OS=Homo sapiens GN=HNRNPC	6	8	4	7	7	5	6
P09919-2	CSF3_HUMAN	21978	CSF3	Isoform Short of Granulocyte colony-stimulating factor OS=Homo sapiens GN=CSF3	6	13	11	3	2	4	4
P13667	PDIA4_HUMAN	72932	PDIA4	Protein disulfide-isomerase A4 OS=Homo sapiens GN=PDIA4 PE=1 SV=2	6	11	3	4	7	7	5
P18065	IBP2_HUMAN	34814	IGFBP2	Insulin-like growth factor-binding protein 2 OS=Homo sapiens GN=IGFBP2 PE=1 SV=2	6	7	6	6	5	6	7
Q641Q3	METRL_HUMAN	34398	METRNL	Meteorin-like protein OS=Homo sapiens GN=METRNL PE=2 SV=1	6	7	10	3	2	6	9
Q1JUQ5	Q1JUQ5_HUMAN	10119	FKBP12-Exip2	Peptidyl-prolyl cis-trans isomerase OS=Homo sapiens GN=FKBP12-Exip2 PE=2 SV=1	6	8	7	4	6	7	5
P13497-2	BMP1_HUMAN	82900	BMP1	Isoform BMP1-1 of Bone morphogenetic protein 1 OS=Homo sapiens GN=BMP1	5	9	10		1	12	5
O75347	TBCA_HUMAN	12855	TBCA	Tubulin-specific chaperone A OS=Homo sapiens GN=TBCA PE=1 SV=3	6	7	6	5	8	5	5
P35080-2	PROF2_HUMAN	15088	PFN2	Isoform IIb of Profilin-2 OS=Homo sapiens GN=PFN2	6	4	4	8	8	8	4
P35443	TSP4_HUMAN	105869	THBS4	Thrombospondin-4 OS=Homo sapiens GN=THBS4 PE=1 SV=2	6	5	5	9	9	5	3
P49773	HINT1_HUMAN	13802	HINT1	Histidine triad nucleotide-binding protein 1 OS=Homo sapiens GN=HINT1 PE=1 SV=2	6	9	10	2	2	5	8
P59998-2	ARPC4_HUMAN	71719	ARPC4	Isoform 2 of Actin-related protein 2/3 complex subunit 4 OS=Homo sapiens GN=ARPC4	6	4	6	7	8	7	4
P84085	ARF5_HUMAN	20530	ARF5	ADP-ribosylation factor 5 OS=Homo sapiens GN=ARF5 PE=1 SV=2	6	6	3	6	5	9	7
Q14393-2	GAS6_HUMAN	74925	GAS6	Isoform 2 of Growth arrest-specific protein 6 OS=Homo sapiens GN=GAS6	6	5	7	5	5	6	8
Q16643-3	DREB_HUMAN	76300	DBN1	Isoform 3 of Drebrin OS=Homo sapiens GN=DBN1	6	8	5	1	3	11	8
Q92692-2	PVRL2_HUMAN	51359	PVRL2	Isoform Alpha of Poliovirus receptor-related protein 2 OS=Homo sapiens GN=PVRL2	6	6	5	5	4	5	11
Q96AY3	FKB10_HUMAN	64245	FKBP10	Peptidyl-prolyl cis-trans isomerase FKBP10 OS=Homo sapiens GN=FKBP10 PE=1 SV=1	6	6	9	3	5	10	3
Q99436	PSB7_HUMAN	29965	PSMB7	Proteasome subunit beta type-7 OS=Homo sapiens GN=PSMB7 PE=1 SV=1	6	4	4	10	12	4	2
Q9BRA2	TXD17_HUMAN	13941	TXNDC17	Thioredoxin domain-containing protein 17 OS=Homo sapiens GN=TXNDC17 PE=1 SV=1	6	5	6	6	7	8	4
Q9UBQ6	EXTL2_HUMAN	37466	EXTL2	Exostosin-like 2 OS=Homo sapiens GN=EXTL2 PE=1 SV=1	6	5	3	1	2	10	15
Q9UJ70	NAGK_HUMAN	37376	NAGK	N-acetyl-D-glucosamine kinase OS=Homo sapiens GN=NAGK PE=1 SV=4	6	2	3	9	10	7	5
F8WCF6	F8WCF6_HUMAN	21059	ARPC4-TTLL3	Protein ARPC4-TTLL3 OS=Homo sapiens GN=ARPC4-TTLL3 PE=2 SV=1	6	4	6	7	8	7	4
Q13126	MTAP_HUMAN	31236	MTAP	S-methyl-5'-thioadenosine phosphorylase OS=Homo sapiens GN=MTAP PE=1 SV=2	5	2	4	12	14	4	
P04732	MT1E_HUMAN	6014	MT1E	Metallothionein-1E OS=Homo sapiens GN=MT1E PE=1 SV=1	6	3	5	6	4	9	8
P07864	LDHC_HUMAN	36311	LDHC	L-lactate dehydrogenase C chain OS=Homo sapiens GN=LDHC PE=1 SV=4	6	7	7	4	4	5	8
P10809	CH60_HUMAN	61055	HSPD1	60 kDa heat shock protein, mitochondrial OS=Homo sapiens GN=HSPD1 PE=1 SV=2	6	8	8	5	5	4	5

P13640-2	MT1G_HUMAN	6070	MT1G	Isoform 2 of Metallothionein-1G OS=Homo sapiens GN=MT1G	6	3	5	6	4	9	8
P14649	MYL6B_HUMAN	22764	MYL6B	Myosin light chain 6B OS=Homo sapiens GN=MYL6B PE=1 SV=1	6	8	9	4	4	5	5
P30050	RL12_HUMAN	17819	RPL12	60S ribosomal protein L12 OS=Homo sapiens GN=RPL12 PE=1 SV=1	6	7	3	4	4	10	7
P60033	CD81_HUMAN	25809	CD81	CD81 antigen OS=Homo sapiens GN=CD81 PE=1 SV=1	6	7	5	8	6	3	6
P61604	CH10_HUMAN	10932	HSPE1	10 kDa heat shock protein, mitochondrial OS=Homo sapiens GN=HSPE1 PE=1 SV=2	6	7	9	3	4	5	7
P80297	MT1X_HUMAN	6068	MT1X	Metallothionein-1X OS=Homo sapiens GN=MT1X PE=1 SV=1	6	3	5	6	4	9	8
Q13404	UB2V1_HUMAN	16495	UBE2V1	Ubiquitin-conjugating enzyme E2 variant 1 OS=Homo sapiens GN=UBE2V1 PE=1 SV=2	6	7	4	4	4	9	7
Q6ZMR3	LDH6A_HUMAN	36507	LDHAL6A	L-lactate dehydrogenase A-like 6A OS=Homo sapiens GN=LDHAL6A PE=2 SV=1	6	7	7	4	4	5	8
Q9BWS9-3	CHID1_HUMAN	41678	CHID1	Isoform 3 of Chitinase domain-containing protein 1 OS=Homo sapiens GN=CHID1	6	6	8	3	2	7	9
Q9Y678	COPG1_HUMAN	97718	COPG1	Coatomer subunit gamma-1 OS=Homo sapiens GN=COPG1 PE=1 SV=1	6	4	7	8	9	2	5
Q07092-2	COGA1_HUMAN	157557	COL16A1	Isoform 2 of Collagen alpha-1(XVI) chain OS=Homo sapiens GN=COL16A1	4	10	8			9	8
P00390-2	GSHR_HUMAN	51701	GSR	Isoform Cytoplasmic of Glutathione reductase, mitochondrial OS=Homo sapiens GN=GSR	6	4	4	11	10	2	3
P04004	VTNC_HUMAN	54306	VTN	Vitronectin OS=Homo sapiens GN=VTN PE=1 SV=1	6	2	4	7	6	8	7
P12081-4	SYHC_HUMAN	54847	HARS	Isoform 4 of Histidine--tRNA ligase, cytoplasmic OS=Homo sapiens GN=HARS	6	2	1	9	3	7	12
P17096	HMGA1_HUMAN	11676	HMGA1	High mobility group protein HMG-I/HMG-Y OS=Homo sapiens GN=HMGA1 PE=1 SV=3	6	4	4	8	9	4	5
P58546	MTPN_HUMAN	12895	MTPN	Myotrophin OS=Homo sapiens GN=MTPN PE=1 SV=2	6	7	9	4	3	7	4
P62857	RS28_HUMAN	7841	RPS28	40S ribosomal protein S28 OS=Homo sapiens GN=RPS28 PE=1 SV=1	6	9	5	3	4	7	6
P63010-2	AP2B1_HUMAN	105692	AP2B1	Isoform 2 of AP-2 complex subunit beta OS=Homo sapiens GN=AP2B1	6	6	7	6	7	4	4
Q02388-2	CO7A1_HUMAN	292267	COL7A1	Isoform 2 of Collagen alpha-1(VII) chain OS=Homo sapiens GN=COL7A1	6	2	8	4	1	14	5
Q09028-3	RBBP4_HUMAN	46158	RBBP4	Isoform 3 of Histone-binding protein RBBP4 OS=Homo sapiens GN=RBBP4	6	4	2	6	7	5	10
Q09666	AHNK_HUMAN	629101	AHNAK	Neuroblast differentiation-associated protein AHNAK OS=Homo sapiens GN=AHNAK PE=1 SV=2	6	8	3	4	2	10	7
Q8NHW5	RLA0L_HUMAN	34364	RPLP0P6	60S acidic ribosomal protein P0-like OS=Homo sapiens GN=RPLP0P6 PE=5 SV=1	6	8	4	6	6	5	5
Q99426	TBCB_HUMAN	27326	TBCB	Tubulin-folding cofactor B OS=Homo sapiens GN=TBCB PE=1 SV=2	6	5	7	4	8	8	2
Q9NRX4	PHP14_HUMAN	13833	PHPT1	14 kDa phosphohistidine phosphatase OS=Homo sapiens GN=PHPT1 PE=1 SV=1	6	3	4	5	7	9	6
Q9Y2B0	CNPY2_HUMAN	20652	CNPY2	Protein canopy homolog 2 OS=Homo sapiens GN=CNPY2 PE=1 SV=1	6	6	4	4	5	8	7
Q9Y3B8	ORN_HUMAN	26833	REXO2	Oligoribonuclease, mitochondrial OS=Homo sapiens GN=REXO2 PE=1 SV=3	6	5	6	8	6	5	4
Q9Y625	GPC6_HUMAN	62736	GPC6	Glypican-6 OS=Homo sapiens GN=GPC6 PE=1 SV=1	6	8	6	4	1	6	9
G3V2F7	G3V2F7_HUMAN	42209	TMEM189	HCG2044781 OS=Homo sapiens GN=TMEM189 PE=2 SV=1	6	7	4	4	4	8	7
O00584	RNT2_HUMAN	29481	RNASET2	Ribonuclease T2 OS=Homo sapiens GN=RNASET2 PE=1 SV=2	5	9		1	4	11	9
Q76M96-2	CCD80_HUMAN	109492	CCDC80	Isoform 2 of Coiled-coil domain-containing protein 80 OS=Homo sapiens GN=CCDC80	5		1	5	6	13	9
O14561	ACPM_HUMAN	17417	NDUFAB1	Acyl carrier protein, mitochondrial OS=Homo sapiens GN=NDUFAB1 PE=1 SV=3	6	7	4	5	6	6	5
O15143	ARC1B_HUMAN	40950	ARPC1B	Actin-related protein 2/3 complex subunit 1B OS=Homo sapiens GN=ARPC1B PE=1 SV=3	6	3	1	11	6	5	7
O76015	KRT38_HUMAN	50480	KRT38	Keratin, type I cuticular Ha8 OS=Homo sapiens GN=KRT38 PE=2 SV=3	6	8	9	6	4	3	3
P10145-2	IL8_HUMAN	11338	IL8	Isoform 2 of Interleukin-8 OS=Homo sapiens GN=IL8	6	7	7	1	4	6	8
P17066	HSP76_HUMAN	71028	HSPA6	Heat shock 70 kDa protein 6 OS=Homo sapiens GN=HSPA6 PE=1 SV=2	6	10	7	3	2	7	4
P28161-2	GSTM2_HUMAN	22644	GSTM2	Isoform 2 of Glutathione S-transferase Mu 2 OS=Homo sapiens GN=GSTM2	6	4	4	7	5	5	8
P31431-2	SDC4_HUMAN	16545	SDC4	Isoform 2 of Syndecan-4 OS=Homo sapiens GN=SDC4	6	7	13	2	3	4	4
P47755	CAZA2_HUMAN	32949	CAPZA2	F-actin-capping protein subunit alpha-2 OS=Homo sapiens GN=CAPZA2 PE=1 SV=3	6	7	2	9	7	3	5
Q14192	FHL2_HUMAN	32193	FHL2	Four and a half LIM domains protein 2 OS=Homo sapiens GN=FHL2 PE=1 SV=3	6	5	4	7	7	5	5
Q15942	ZYX_HUMAN	61277	ZYX	Zyxin OS=Homo sapiens GN=ZYX PE=1 SV=1	6	8	7	5	4	7	2
Q53FA7	QORX_HUMAN	35536	TP53I3	Quinone oxidoreductase PIG3 OS=Homo sapiens GN=TP53I3 PE=1 SV=2	6	1	2	13	13	3	1
Q92764	KRT35_HUMAN	50361	KRT35	Keratin, type I cuticular Ha5 OS=Homo sapiens GN=KRT35 PE=2 SV=5	6	8	8	6	4	3	4
Q96HC4	PDLI5_HUMAN	63945	PDLIM5	PDZ and LIM domain protein 5 OS=Homo sapiens GN=PDLIM5 PE=1 SV=5	6	4	1	9	10	4	5
Q9Y3C6	PPIL1_HUMAN	18237	PPIL1	Peptidyl-prolyl cis-trans isomerase-like 1 OS=Homo sapiens GN=PPIL1 PE=1 SV=1	6	4	4	6	7	4	8
Q53XA7	Q53XA7_HUMAN	38614	DKFZp686F132	Fumarylacetoacetase OS=Homo sapiens GN=DKFZp686F13224 PE=2 SV=1	6	4	3	7	9	4	6

			24								
P55290-4	CAD13_HUMAN	83397	CDH13	Isoform 4 of Cadherin-13 OS=Homo sapiens GN=CDH13	5	5	8	9	8		3
O75830	SPI2_HUMAN	46145	SERPINI2	Serpin I2 OS=Homo sapiens GN=SERPINI2 PE=1 SV=1	6	1	3	6	4	5	13
O76013-2	KRT36_HUMAN	47485	KRT36	Isoform 2 of Keratin, type I cuticular Ha6 OS=Homo sapiens GN=KRT36	6	8	8	6	4	3	3
O76014	KRT37_HUMAN	49747	KRT37	Keratin, type I cuticular Ha7 OS=Homo sapiens GN=KRT37 PE=2 SV=3	6	8	8	6	4	3	3
P01137	TGFB1_HUMAN	44341	TGFB1	Transforming growth factor beta-1 OS=Homo sapiens GN=TGFB1 PE=1 SV=2	6	6	5	3	4	6	8
P28799	GRN_HUMAN	63544	GRN	Granulins OS=Homo sapiens GN=GRN PE=1 SV=2	6	5	5	3	2	8	9
P30520	PURA2_HUMAN	50097	ADSS	Adenylosuccinate synthetase isozyme 2 OS=Homo sapiens GN=ADSS PE=1 SV=3	6	5	3	7	4	4	9
P55285-2	CADH6_HUMAN	73864	CDH6	Isoform 2 of Cadherin-6 OS=Homo sapiens GN=CDH6	6	8	3	6	4	7	4
Q12905	ILF2_HUMAN	43062	ILF2	Interleukin enhancer-binding factor 2 OS=Homo sapiens GN=ILF2 PE=1 SV=2	6	3	5	7	9	4	4
Q14525	KT33B_HUMAN	46214	KRT33B	Keratin, type I cuticular Ha3-II OS=Homo sapiens GN=KRT33B PE=2 SV=3	6	8	8	6	4	3	3
Q14532	K1H2_HUMAN	50343	KRT32	Keratin, type I cuticular Ha2 OS=Homo sapiens GN=KRT32 PE=1 SV=3	6	8	8	6	4	3	3
Q14651	PLSI_HUMAN	70253	PLS1	Plastin-1 OS=Homo sapiens GN=PLS1 PE=1 SV=2	6	9	6	4	5	3	5
Q15323	K1H1_HUMAN	47237	KRT31	Keratin, type I cuticular Ha1 OS=Homo sapiens GN=KRT31 PE=2 SV=3	6	8	8	6	4	3	3
Q92688-2	AN32B_HUMAN	22277	ANP32B	Isoform 2 of Acidic leucine-rich nuclear phosphoprotein 32 family member B OS=Homo sapiens GN=ANP32B	6	7	6	6	4	5	4
Q96RW7	HMCN1_HUMAN	613390	HMCN1	Hemicentin-1 OS=Homo sapiens GN=HMCN1 PE=1 SV=2	6	6	6	2	6	9	3
Q13438-4	OS9_HUMAN	73821	OS9	Isoform 4 of Protein OS-9 OS=Homo sapiens GN=OS9	4	3	4			13	12
O00560	SDCB1_HUMAN	32444	SDCBP	Syntenin-1 OS=Homo sapiens GN=SDCBP PE=1 SV=1	6	3	6	5	8	5	4
P05161	ISG15_HUMAN	17888	ISG15	Ubiquitin-like protein ISG15 OS=Homo sapiens GN=ISG15 PE=1 SV=5	6	2	4	9	7	4	5
P30405	PPIF_HUMAN	22040	PPIF	Peptidyl-prolyl cis-trans isomerase F, mitochondrial OS=Homo sapiens GN=PPIF PE=1 SV=1	6	3	2	11	9	4	2
P60983	GMFB_HUMAN	16713	GMFB	Glia maturation factor beta OS=Homo sapiens GN=GMFB PE=1 SV=2	6	4	3	7	6	5	6
P63208	SKP1_HUMAN	18658	SKP1	S-phase kinase-associated protein 1 OS=Homo sapiens GN=SKP1 PE=1 SV=2	6	5	5	5	7	6	3
Q14103-3	HNRPD_HUMAN	32835	HNRNPD	Isoform 3 of Heterogeneous nuclear ribonucleoprotein D0 OS=Homo sapiens GN=HNRNPD	6	3	7	1	5	8	7
Q15404	RSU1_HUMAN	31540	RSU1	Ras suppressor protein 1 OS=Homo sapiens GN=RSU1 PE=1 SV=3	6	5	4	6	6	5	5
Q49A17-2	GLTL6_HUMAN	67589	GALNTL6	Isoform 2 of Polypeptide N-acetylgalactosaminyltransferase-like 6 OS=Homo sapiens GN=GALNTL6	6	9	5	2	3	6	6
Q8N339	MT1M_HUMAN	6110	MT1M	Metallothionein-1M OS=Homo sapiens GN=MT1M PE=2 SV=2	6	3	5	5	3	8	7
Q13838-2	DX39B_HUMAN	50679	DDX39B	Isoform 2 of Spliceosome RNA helicase DDX39B OS=Homo sapiens GN=DDX39B	5	6		5	5	6	9
P26639	SYTC_HUMAN	83435	TARS	Threonine--tRNA ligase, cytoplasmic OS=Homo sapiens GN=TARS PE=1 SV=3	4			10	7	10	4
P24821-4	TENA_HUMAN	230867	TNC	Isoform 4 of Tenascin OS=Homo sapiens GN=TNC	3	13	17				1
P09429	HMGB1_HUMAN	24894	HMGB1	High mobility group protein B1 OS=Homo sapiens GN=HMGB1 PE=1 SV=3	6	3	1	10	10	5	1
P11940-2	PABP1_HUMAN	61181	PABPC1	Isoform 2 of Polyadenylate-binding protein 1 OS=Homo sapiens GN=PABPC1	6	11	2	5	6	5	1
P14854	CX6B1_HUMAN	10192	COX6B1	Cytochrome c oxidase subunit 6B1 OS=Homo sapiens GN=COX6B1 PE=1 SV=2	6	5	6	4	4	5	6
P57721-2	PCBP3_HUMAN	36817	PCBP3	Isoform 2 of Poly(rC)-binding protein 3 OS=Homo sapiens GN=PCBP3	6	6	8	3	4	4	5
Q13283	G3BP1_HUMAN	52164	G3BP1	Ras GTPase-activating protein-binding protein 1 OS=Homo sapiens GN=G3BP1 PE=1 SV=1	6	6	5	2	6	6	5
Q8NBJ7	SUMF2_HUMAN	33843	SUMF2	Sulfatase-modifying factor 2 OS=Homo sapiens GN=SUMF2 PE=1 SV=2	6	5	5	5	5	5	5
Q9BQT9-2	CSTN3_HUMAN	107033	CLSTN3	Isoform 2 of Calsyntenin-3 OS=Homo sapiens GN=CLSTN3	6	6	6	4	3	5	6
Q9BSJ8-2	ESYT1_HUMAN	124003	ESYT1	Isoform 2 of Extended synaptotagmin-1 OS=Homo sapiens GN=ESYT1	6	3	3	6	6	6	6
D6REA1	D6REA1_HUMAN	52748	SIL1	Nucleotide exchange factor SIL1 OS=Homo sapiens GN=SIL1 PE=2 SV=1	6	3	5	3	3	9	7
P28838	AMPL_HUMAN	56166	LAP3	Cytosol aminopeptidase OS=Homo sapiens GN=LAP3 PE=1 SV=3	5	2		9	7	5	7
O00233	PSMD9_HUMAN	24682	PSMD9	26S proteasome non-ATPase regulatory subunit 9 OS=Homo sapiens GN=PSMD9 PE=1 SV=3	6	5	3	6	3	6	6
O75223	GGCT_HUMAN	21008	GGCT	Gamma-glutamylcyclotransferase OS=Homo sapiens GN=GGCT PE=1 SV=1	6	4	4	8	6	4	3
P06132	DCUP_HUMAN	40787	UROD	Uroporphyrinogen decarboxylase OS=Homo sapiens GN=UROD PE=1 SV=2	6	6	3	5	5	3	7
P20933	ASPG_HUMAN	37208	AGA	N(4)-(beta-N-acetylglucosaminyl)-L-asparaginase OS=Homo sapiens GN=AGA PE=1 SV=2	6	5	4	3	2	7	8
P23381-2	SYWC_HUMAN	48180	WARS	Isoform 2 of Tryptophan--tRNA ligase, cytoplasmic OS=Homo sapiens GN=WARS	6	4	4	6	7	3	5

P26885	FKBP2_HUMAN	15649	FKBP2	Peptidyl-prolyl cis-trans isomerase FKBP2 OS=Homo sapiens GN=FKBP2 PE=1 SV=2	6	4	5	3	6	7	4
P62873	GBB1_HUMAN	37377	GNB1	Guanine nucleotide-binding protein G(I)/G(S)/G(T) subunit beta-1 OS=Homo sapiens GN=GNB1 PE=1 SV=3	6	3	1	7	6	9	3
Q58FF6	H90B4_HUMAN	58264	HSP90AB4P	Putative heat shock protein HSP 90-beta 4 OS=Homo sapiens GN=HSP90AB4P PE=5 SV=1	6	6	9	3	3	4	4
Q7KZF4	SND1_HUMAN	101997	SND1	Staphylococcal nuclease domain-containing protein 1 OS=Homo sapiens GN=SND1 PE=1 SV=1	6	2	3	9	9	4	2
Q9BS40	LXN_HUMAN	25750	LXN	Latexin OS=Homo sapiens GN=LXN PE=1 SV=2	6	3	5	6	6	3	6
Q9BZM5	N2DL2_HUMAN	27368	ULBP2	NKG2D ligand 2 OS=Homo sapiens GN=ULBP2 PE=1 SV=1	6	5	7	2	4	7	4
Q9NZP8	C1RL_HUMAN	53498	C1RL	Complement C1r subcomponent-like protein OS=Homo sapiens GN=C1RL PE=1 SV=2	6	6	7	2	2	8	4
Q9UJU6	DBNL_HUMAN	48207	DBNL	Drebrin-like protein OS=Homo sapiens GN=DBNL PE=1 SV=1	6	7	7	3	1	6	5
P02794	FRIH_HUMAN	21226	FTH1	Ferritin heavy chain OS=Homo sapiens GN=FTH1 PE=1 SV=2	5	1		11	9	4	4
P50452	SPB8_HUMAN	42767	SERPINB8	Serpin B8 OS=Homo sapiens GN=SERPINB8 PE=1 SV=2	5	1		8	9	5	6
P07225	PROS_HUMAN	75123	PROS1	Vitamin K-dependent protein S OS=Homo sapiens GN=PROS1 PE=1 SV=1	6	8	2	3	5	3	7
P36639-2	8ODP_HUMAN	20296	NUDT1	Isoform p22 of 7,8-dihydro-8-oxoguanine triphosphatase OS=Homo sapiens GN=NUDT1	6	4	5	3	5	6	5
P55735	SEC13_HUMAN	35541	SEC13	Protein SEC13 homolog OS=Homo sapiens GN=SEC13 PE=1 SV=3	6	3	3	5	6	6	5
P84090	ERH_HUMAN	12259	ERH	Enhancer of rudimentary homolog OS=Homo sapiens GN=ERH PE=1 SV=1	6	4	4	5	5	5	5
Q05707-2	COEA1_HUMAN	191903	COL14A1	Isoform 2 of Collagen alpha-1(XIV) chain OS=Homo sapiens GN=COL14A1	6	4	1	5	7	6	5
Q13185	CBX3_HUMAN	20811	CBX3	Chromobox protein homolog 3 OS=Homo sapiens GN=CBX3 PE=1 SV=4	6	6	5	3	4	3	7
Q14993	COJA1_HUMAN	115221	COL19A1	Collagen alpha-1(XIX) chain OS=Homo sapiens GN=COL19A1 PE=1 SV=3	6	4	5	3	2	5	9
Q15369	ELOC_HUMAN	12473	TCEB1	Transcription elongation factor B polypeptide 1 OS=Homo sapiens GN=TCEB1 PE=1 SV=1	6	4	4	5	5	5	5
Q8NCW5-2	NNRE_HUMAN	20431	APOA1BP	Isoform 2 of NAD(P)H-hydrate epimerase OS=Homo sapiens GN=APOA1BP	6	4	3	6	6	5	4
Q96C19	EFHD2_HUMAN	26697	EFHD2	EF-hand domain-containing protein D2 OS=Homo sapiens GN=EFHD2 PE=1 SV=1	6	8	6	5	5	2	2
Q06828	FMOD_HUMAN	43179	FMOD	Fibromodulin OS=Homo sapiens GN=FMOD PE=1 SV=2	5	7	5		3	7	6
P04114	APOB_HUMAN	515605	APOB	Apolipoprotein B-100 OS=Homo sapiens GN=APOB PE=1 SV=2	5	4		7	6	7	4
P05120	PAI2_HUMAN	46596	SERPINB2	Plasminogen activator inhibitor 2 OS=Homo sapiens GN=SERPINB2 PE=1 SV=2	2			18	10		
O75436	VP26A_HUMAN	38170	VPS26A	Vacuolar protein sorting-associated protein 26A OS=Homo sapiens GN=VPS26A PE=1 SV=2	6	2	3	6	5	4	7
P05387	RLA2_HUMAN	11665	RPLP2	60S acidic ribosomal protein P2 OS=Homo sapiens GN=RPLP2 PE=1 SV=1	6	5	7	3	5	4	3
P10768	ESTD_HUMAN	31463	ESD	S-formylglutathione hydrolase OS=Homo sapiens GN=ESD PE=1 SV=2	6	3	2	6	7	6	3
P13798	ACPH_HUMAN	81225	APEH	Acylamino-acid-releasing enzyme OS=Homo sapiens GN=APEH PE=1 SV=4	6	4	1	7	8	6	1
P23588	IF4B_HUMAN	69151	EIF4B	Eukaryotic translation initiation factor 4B OS=Homo sapiens GN=EIF4B PE=1 SV=2	6	9	9	2	1	1	5
P24666	PPAC_HUMAN	18042	ACP1	Low molecular weight phosphotyrosine protein phosphatase OS=Homo sapiens GN=ACP1 PE=1 SV=3	6	5	4	2	4	8	4
P31948	STIP1_HUMAN	62639	STIP1	Stress-induced-phosphoprotein 1 OS=Homo sapiens GN=STIP1 PE=1 SV=1	6	6	6	2	3	5	5
P61956	SUMO2_HUMAN	10871	SUMO2	Small ubiquitin-related modifier 2 OS=Homo sapiens GN=SUMO2 PE=1 SV=3	6	5	8	5	5	2	2
P62249	RS16_HUMAN	16445	RPS16	40S ribosomal protein S16 OS=Homo sapiens GN=RPS16 PE=1 SV=2	6	7	3	3	4	5	5
Q9NR12	PDLI7_HUMAN	49845	PDLIM7	PDZ and LIM domain protein 7 OS=Homo sapiens GN=PDLIM7 PE=1 SV=1	6	4	3	4	7	4	5
Q9UJ72	ANX10_HUMAN	37278	ANXA10	Annexin A10 OS=Homo sapiens GN=ANXA10 PE=1 SV=3	6	3	3	7	7	2	5
Q9UL46	PSME2_HUMAN	27402	PSME2	Proteasome activator complex subunit 2 OS=Homo sapiens GN=PSME2 PE=1 SV=4	6	4	3	4	10	3	3
P53621-2	COPA_HUMAN	139324	COPA	Isoform 2 of Coatomer subunit alpha OS=Homo sapiens GN=COPA	5	4		7	6	6	4
Q04756	HGFA_HUMAN	70682	HGFAC	Hepatocyte growth factor activator OS=Homo sapiens GN=HGFAC PE=1 SV=1	5	2		10	8	3	4
Q15436	SC23A_HUMAN	86161	SEC23A	Protein transport protein Sec23A OS=Homo sapiens GN=SEC23A PE=1 SV=2	3			15	11	1	
O75396	SC22B_HUMAN	24593	SEC22B	Vesicle-trafficking protein SEC22b OS=Homo sapiens GN=SEC22B PE=1 SV=4	6	3	2	3	5	7	6
P04216	THY1_HUMAN	17935	THY1	Thy-1 membrane glycoprotein OS=Homo sapiens GN=THY1 PE=1 SV=2	6	5	3	5	4	6	3
P06703	S10A6_HUMAN	10180	S100A6	Protein S100-A6 OS=Homo sapiens GN=S100A6 PE=1 SV=1	6	3	4	6	5	5	3
P08648	ITA5_HUMAN	114536	ITGA5	Integrin alpha-5 OS=Homo sapiens GN=ITGA5 PE=1 SV=2	6	3	5	6	6	3	3
P29692-2	EF1D_HUMAN	71408	EEF1D	Isoform 2 of Elongation factor 1-delta OS=Homo sapiens GN=EEF1D	6	2	2	6	7	6	3

P35606	COPB2_HUMAN	102487	COPB2	Coatomer subunit beta' OS=Homo sapiens GN=COPB2 PE=1 SV=2	6	4	2	6	6	5	3
P61586	RHOA_HUMAN	21768	RHOA	Transforming protein RhoA OS=Homo sapiens GN=RHOA PE=1 SV=1	6	4	2	5	6	5	4
P62879	GBB2_HUMAN	37331	GNB2	Guanine nucleotide-binding protein G(I)/G(S)/G(T) subunit beta-2 OS=Homo sapiens GN=GNB2 PE=1 SV=3	6	2	1	6	8	6	3
P67775	PP2AA_HUMAN	35594	PPP2CA	Serine/threonine-protein phosphatase 2A catalytic subunit alpha isoform OS=Homo sapiens GN=PPP2CA PE=1 SV=1	6	5	4	6	5	3	3
Q8TDQ7-3	GNPI2_HUMAN	29173	GNPDA2	Isoform 3 of Glucosamine-6-phosphate isomerase 2 OS=Homo sapiens GN=GNPDA2	6	4	4	5	7	4	2
Q96CN7	ISOC1_HUMAN	32237	ISOC1	Isochorismatase domain-containing protein 1 OS=Homo sapiens GN=ISOC1 PE=1 SV=3	6	4	3	6	7	3	3
Q99523	SORT_HUMAN	92068	SORT1	Sortilin OS=Homo sapiens GN=SORT1 PE=1 SV=3	6	4	4	4	2	6	6
Q99798	ACON_HUMAN	85425	ACO2	Aconitate hydratase, mitochondrial OS=Homo sapiens GN=ACO2 PE=1 SV=2	6	4	1	10	6	3	2
Q6FGY5	Q6FGY5_HUMAN	21801	CKAP1	CKAP1 protein OS=Homo sapiens GN=CKAP1 PE=2 SV=1	6	5	5	3	7	4	2
P02792	FRIL_HUMAN	20020	FTL	Ferritin light chain OS=Homo sapiens GN=FTL PE=1 SV=2	5	2	4	7	6	7	
P10646	TFPI1_HUMAN	35015	TFPI	Tissue factor pathway inhibitor OS=Homo sapiens GN=TFPI PE=1 SV=1	4	7	8			5	6
P21741	MK_HUMAN	15585	MDK	Midkine OS=Homo sapiens GN=MDK PE=1 SV=1	4	8	8			4	6
Q9UBG0	MRC2_HUMAN	166674	MRC2	C-type mannose receptor 2 OS=Homo sapiens GN=MRC2 PE=1 SV=2	4			10	8	5	3
O95967	FBLN4_HUMAN	49405	EFEMP2	EGF-containing fibulin-like extracellular matrix protein 2 OS=Homo sapiens GN=EFEMP2 PE=1 SV=3	6	7	5	4	4	3	2
P48741	HSP77_HUMAN	40244	HSPA7	Putative heat shock 70 kDa protein 7 OS=Homo sapiens GN=HSPA7 PE=5 SV=2	6	7	5	3	2	5	3
P52799	EFNB2_HUMAN	36923	EFNB2	Ephrin-B2 OS=Homo sapiens GN=EFNB2 PE=1 SV=1	6	5	6	2	1	6	5
P54802	ANAG_HUMAN	82266	NAGLU	Alpha-N-acetylglucosaminidase OS=Homo sapiens GN=NAGLU PE=1 SV=2	6	1	5	1	4	7	7
Q01105-2	SET_HUMAN	32234	SET	Isoform 2 of Protein SET OS=Homo sapiens GN=SET	6	5	4	4	4	3	5
Q08257-3	QOR_HUMAN	31528	CRYZ	Isoform 3 of Quinone oxidoreductase OS=Homo sapiens GN=CRYZ	6	3	2	6	6	4	4
Q14247-3	SRC8_HUMAN	57467	CTTN	Isoform 3 of Src substrate cortactin OS=Homo sapiens GN=CTTN	6	4	4	2	2	5	8
Q16527	CSRP2_HUMAN	20954	CSRP2	Cysteine and glycine-rich protein 2 OS=Homo sapiens GN=CSRP2 PE=1 SV=3	6	3	3	4	4	5	6
Q9BQ16-7	TICN3_HUMAN	36153	SPOCK3	Isoform 7 of Testican-3 OS=Homo sapiens GN=SPOCK3	6	2	7	5	3	4	4
Q92598-2	HS105_HUMAN	92116	HSPH1	Isoform Beta of Heat shock protein 105 kDa OS=Homo sapiens GN=HSPH1	5	3	5	4	5	8	
P54727	RD23B_HUMAN	43171	RAD23B	UV excision repair protein RAD23 homolog B OS=Homo sapiens GN=RAD23B PE=1 SV=1	5	8	4		4	5	4
Q10472	GALT1_HUMAN	64219	GALNT1	Polypeptide N-acetylgalactosaminyltransferase 1 OS=Homo sapiens GN=GALNT1 PE=1 SV=1	5	9	6		2	6	2
P29373	RABP2_HUMAN	15693	CRABP2	Cellular retinoic acid-binding protein 2 OS=Homo sapiens GN=CRABP2 PE=1 SV=2	5		2	6	6	3	8
E9PAV3	NACAM_HUMAN	205422	NACA	Nascent polypeptide-associated complex subunit alpha, muscle-specific form OS=Homo sapiens GN=NACA PE=1 SV=1	6	3	3	6	5	2	5
P15018	LIF_HUMAN	22008	LIF	Leukemia inhibitory factor OS=Homo sapiens GN=LIF PE=1 SV=1	6	8	5	1	2	3	5
P23434	GCSH_HUMAN	18885	GCSH	Glycine cleavage system H protein, mitochondrial OS=Homo sapiens GN=GCSH PE=1 SV=2	6	4	4	3	6	3	4
P28072	PSB6_HUMAN	25358	PSMB6	Proteasome subunit beta type-6 OS=Homo sapiens GN=PSMB6 PE=1 SV=4	6	2	2	6	5	6	3
P55854	SUMO3_HUMAN	11637	SUMO3	Small ubiquitin-related modifier 3 OS=Homo sapiens GN=SUMO3 PE=1 SV=2	6	4	5	5	5	2	3
Q13907	IDI1_HUMAN	26319	IDI1	Isopentenyl-diphosphate Delta-isomerase 1 OS=Homo sapiens GN=IDI1 PE=1 SV=2	6	2	3	5	8	2	4
Q6UX71	PXDC2_HUMAN	59583	PLXDC2	Plexin domain-containing protein 2 OS=Homo sapiens GN=PLXDC2 PE=1 SV=1	6	4	4	3	3	4	6
Q96C90	PP14B_HUMAN	15911	PPP1R14B	Protein phosphatase 1 regulatory subunit 14B OS=Homo sapiens GN=PPP1R14B PE=1 SV=3	6	5	5	3	3	2	6
Q9NS15-2	LTBP3_HUMAN	134292	LTBP3	Isoform 2 of Latent-transforming growth factor beta-binding protein 3 OS=Homo sapiens GN=LTBP3	6	2	1	4	2	9	6
S4R3N1	S4R3N1_HUMAN	29737	HSPE1-MOB4	Protein HSPE1-MOB4 OS=Homo sapiens GN=HSPE1-MOB4 PE=3 SV=1	6	5	7	3	2	3	4
O75787	RENR_HUMAN	39008	ATP6AP2	Renin receptor OS=Homo sapiens GN=ATP6AP2 PE=1 SV=2	5	3	4	1		10	6
O60565	GREM1_HUMAN	20697	GREM1	Gremlin-1 OS=Homo sapiens GN=GREM1 PE=1 SV=1	5	6	6		3	5	4
P50453	SPB9_HUMAN	42404	SERPINB9	Serpin B9 OS=Homo sapiens GN=SERPINB9 PE=1 SV=1	5	1		6	7	4	6
P20930	FILA_HUMAN	435170	FLG	Filaggrin OS=Homo sapiens GN=FLG PE=1 SV=3	3			15	7		2
O14979-2	HNRDL_HUMAN	33589	HNRNPDL	Isoform 2 of Heterogeneous nuclear ribonucleoprotein D-like OS=Homo sapiens	6	6	2	3	4	2	6

				GN=HNRNPDL							
P02749	APOH_HUMAN	38298	APOH	Beta-2-glycoprotein 1 OS=Homo sapiens GN=APOH PE=1 SV=3	6	3	2	6	4	4	4
P43034	LIS1_HUMAN	46638	PAFAH1B1	Platelet-activating factor acetylhydrolase IB subunit alpha OS=Homo sapiens GN=PAFAH1B1 PE=1 SV=2	6	3	3	4	5	3	5
P52888	THOP1_HUMAN	78840	THOP1	Thimet oligopeptidase OS=Homo sapiens GN=THOP1 PE=1 SV=2	6	1	3	7	7	3	2
P62714	PP2AB_HUMAN	35575	PPP2CB	Serine/threonine-protein phosphatase 2A catalytic subunit beta isoform OS=Homo sapiens GN=PPP2CB PE=1 SV=1	6	4	4	6	5	2	2
P67809	YBOX1_HUMAN	35924	YBX1	Nuclease-sensitive element-binding protein 1 OS=Homo sapiens GN=YBX1 PE=1 SV=3	6	4	4	4	5	1	5
Q99873-2	ANM1_HUMAN	39929	PRMT1	Isoform 2 of Protein arginine N-methyltransferase 1 OS=Homo sapiens GN=PRMT1	6	4	4	4	5	2	4
Q9NZL9-2	MAT2B_HUMAN	36401	MAT2B	Isoform 2 of Methionine adenosyltransferase 2 subunit beta OS=Homo sapiens GN=MAT2B	6	1	1	4	6	4	7
P48637	GSHB_HUMAN	52385	GSS	Glutathione synthetase OS=Homo sapiens GN=GSS PE=1 SV=1	5	4	1	7	9		2
Q14444-2	CAPR1_HUMAN	76862	CAPRIN1	Isoform 2 of Caprin-1 OS=Homo sapiens GN=CAPRIN1	5	4	3	8	7		1
O14773	TPP1_HUMAN	61248	TPP1	Tripeptidyl-peptidase 1 OS=Homo sapiens GN=TPP1 PE=1 SV=2	5	6	4	2		8	3
Q13561-2	DCTN2_HUMAN	44820	DCTN2	Isoform 2 of Dynactin subunit 2 OS=Homo sapiens GN=DCTN2	5	3		3	4	6	7
Q9H853	TBA4B_HUMAN	27551	TUBA4B	Putative tubulin-like protein alpha-4B OS=Homo sapiens GN=TUBA4B PE=5 SV=2	4			7	9	2	5
O60869-2	EDF1_HUMAN	15481	EDF1	Isoform 2 of Endothelial differentiation-related factor 1 OS=Homo sapiens GN=EDF1	6	8	3	2	2	2	5
P07602-2	SAP_HUMAN	58356	PSAP	Isoform Sap-mu-6 of Prosaposin OS=Homo sapiens GN=PSAP	6	3	5	2	5	6	1
P11413-2	G6PD_HUMAN	63827	G6PD	Isoform Long of Glucose-6-phosphate 1-dehydrogenase OS=Homo sapiens GN=G6PD	6	1	1	5	5	4	6
P19623	SPEE_HUMAN	33825	SRM	Spermidine synthase OS=Homo sapiens GN=SRM PE=1 SV=1	6	1	3	4	3	5	6
P29218-3	IMPA1_HUMAN	36695	IMPA1	Isoform 3 of Inositol monophosphatase 1 OS=Homo sapiens GN=IMPA1	6	5	4	3	6	2	2
P30043	BLVRB_HUMAN	22119	BLVRB	Flavin reductase (NADPH) OS=Homo sapiens GN=BLVRB PE=1 SV=3	6	2	1	6	7	4	2
P61019	RAB2A_HUMAN	23546	RAB2A	Ras-related protein Rab-2A OS=Homo sapiens GN=RAB2A PE=1 SV=1	6	4	2	4	3	6	3
P62269	RS18_HUMAN	17719	RPS18	40S ribosomal protein S18 OS=Homo sapiens GN=RPS18 PE=1 SV=3	6	3	3	5	3	3	5
Q10567-2	AP1B1_HUMAN	103921	AP1B1	Isoform B of AP-1 complex subunit beta-1 OS=Homo sapiens GN=AP1B1	6	2	2	5	6	3	4
Q58FF8	H90B2_HUMAN	44349	HSP90AB2P	Putative heat shock protein HSP 90-beta 2 OS=Homo sapiens GN=HSP90AB2P PE=1 SV=2	6	5	5	4	2	2	4
Q96G03	PGM2_HUMAN	68283	PGM2	Phosphoglucomutase-2 OS=Homo sapiens GN=PGM2 PE=1 SV=4	6	4	4	3	2	7	2
Q9UHX1-2	PUF60_HUMAN	58171	PUF60	Isoform 2 of Poly(U)-binding-splicing factor PUF60 OS=Homo sapiens GN=PUF60	6	3	3	5	5	4	2
Q9UKM7	MA1B1_HUMAN	79580	MAN1B1	Endoplasmic reticulum mannosyl-oligosaccharide 1,2-alpha-mannosidase OS=Homo sapiens GN=MAN1B1 PE=1 SV=2	6	5	5	2	2	5	3
P36871	PGM1_HUMAN	61449	PGM1	Phosphoglucomutase-1 OS=Homo sapiens GN=PGM1 PE=1 SV=3	5	5	5	2		5	5
P43490	NAMPT_HUMAN	55521	NAMPT	Nicotinamide phosphoribosyltransferase OS=Homo sapiens GN=NAMPT PE=1 SV=1	5	2		5	7	3	5
P40189	IL6RB_HUMAN	103537	IL6ST	Interleukin-6 receptor subunit beta OS=Homo sapiens GN=IL6ST PE=1 SV=2	5		2	6	5	7	2
Q9BVH7	SIA7E_HUMAN	38443	ST6GALNAC5	Alpha-N-acetylgalactosaminide alpha-2,6-sialyltransferase 5 OS=Homo sapiens GN=ST6GALNAC5 PE=2 SV=1	4	4	1			6	11
P00352	AL1A1_HUMAN	54862	ALDH1A1	Retinal dehydrogenase 1 OS=Homo sapiens GN=ALDH1A1 PE=1 SV=2	4			8	5	6	3
Q13332-2	PTPRS_HUMAN	215980	PTPRS	Isoform PTPS-MEA of Receptor-type tyrosine-protein phosphatase S OS=Homo sapiens GN=PTPRS	4			5	5	7	5
Q7Z7M9	GALT5_HUMAN	106266	GALNT5	Polypeptide N-acetylgalactosaminyltransferase 5 OS=Homo sapiens GN=GALNT5 PE=1 SV=1	4			1	2	9	10
Q9BWD1	THIC_HUMAN	41351	ACAT2	Acetyl-CoA acetyltransferase, cytosolic OS=Homo sapiens GN=ACAT2 PE=1 SV=2	4			3	6	7	6
O00115	DNS2A_HUMAN	39581	DNASE2	Deoxyribonuclease-2-alpha OS=Homo sapiens GN=DNASE2 PE=1 SV=2	6	3	2	2	1	5	8
O43493-2	TGON2_HUMAN	45880	TGOLN2	Isoform TGN46 of Trans-Golgi network integral membrane protein 2 OS=Homo sapiens GN=TGOLN2	6	4	3	1	2	5	6
P52434	RPAB3_HUMAN	17143	POLR2H	DNA-directed RNA polymerases I, II, and III subunit RPABC3 OS=Homo sapiens GN=POLR2H PE=1 SV=4	6	2	2	7	6	2	2
P63313	TYB10_HUMAN	5026	TMSB10	Thymosin beta-10 OS=Homo sapiens GN=TMSB10 PE=1 SV=2	6	4	5	3	4	3	2
Q10588	BST1_HUMAN	35724	BST1	ADP-ribosyl cyclase 2 OS=Homo sapiens GN=BST1 PE=1 SV=2	6	4	2	4	3	3	5
Q5T013	HYI_HUMAN	30406	HYI	Putative hydroxypyruvate isomerase OS=Homo sapiens GN=HYI PE=1 SV=2	6	2	3	2	5	5	4

P14866	HNRPL_HUMAN	64133	HNRNPL	Heterogeneous nuclear ribonucleoprotein L OS=Homo sapiens GN=HNRNPL PE=1 SV=2	5	5	2	6	5	3	
P17900	SAP3_HUMAN	20838	GM2A	Ganglioside GM2 activator OS=Homo sapiens GN=GM2A PE=1 SV=4	5	4	5	2		5	5
Q99538	LGMN_HUMAN	49411	LGMN	Legumain OS=Homo sapiens GN=LGMN PE=1 SV=1	5	8	6	2		2	3
O75094	SLIT3_HUMAN	167713	SLIT3	Slit homolog 3 protein OS=Homo sapiens GN=SLIT3 PE=2 SV=3	5	7	5		1	4	4
P78324-2	SHPS1_HUMAN	55394	SIRPA	Isoform 2 of Tyrosine-protein phosphatase non-receptor type substrate 1 OS=Homo sapiens GN=SIRPA	5	4	3		2	5	7
P21266	GSTM3_HUMAN	26560	GSTM3	Glutathione S-transferase Mu 3 OS=Homo sapiens GN=GSTM3 PE=1 SV=3	5		3	6	4	3	5
P14136-2	GFAP_HUMAN	50289	GFAP	Isoform 2 of Glial fibrillary acidic protein OS=Homo sapiens GN=GFAP	4	8	7			2	4
O14974-5	MYPT1_HUMAN	105641	PPP1R12A	Isoform 5 of Protein phosphatase 1 regulatory subunit 12A OS=Homo sapiens GN=PPP1R12A	6	5	4	2	2	3	4
O60812	HNRCL_HUMAN	32142	HNRNPCL1	Heterogeneous nuclear ribonucleoprotein C-like 1 OS=Homo sapiens GN=HNRNPCL1 PE=1 SV=1	6	4	2	3	4	3	4
P06454-2	PTMA_HUMAN	12074	PTMA	Isoform 2 of Prothymosin alpha OS=Homo sapiens GN=PTMA	6	3	3	3	5	4	2
P09619	PGFRB_HUMAN	123968	PDGFRB	Platelet-derived growth factor receptor beta OS=Homo sapiens GN=PDGFRB PE=1 SV=1	6	3	2	6	4	4	1
P12036-2	NFH_HUMAN	105639	NEFH	Isoform 2 of Neurofilament heavy polypeptide OS=Homo sapiens GN=NEFH	6	5	3	2	3	4	3
P20962	PTMS_HUMAN	11530	PTMS	Parathymosin OS=Homo sapiens GN=PTMS PE=1 SV=2	6	3	3	4	2	5	3
P51149	RAB7A_HUMAN	23490	RAB7A	Ras-related protein Rab-7a OS=Homo sapiens GN=RAB7A PE=1 SV=1	6	1	1	6	7	4	1
P53675-2	CLH2_HUMAN	180296	CLTCL1	Isoform 2 of Clathrin heavy chain 2 OS=Homo sapiens GN=CLTCL1	6	1	2	8	4	4	1
P60953	CDC42_HUMAN	21259	CDC42	Cell division control protein 42 homolog OS=Homo sapiens GN=CDC42 PE=1 SV=2	6	2	6	3	4	4	1
P61086	UBE2K_HUMAN	22407	UBE2K	Ubiquitin-conjugating enzyme E2 K OS=Homo sapiens GN=UBE2K PE=1 SV=3	6	2	4	2	5	5	2
P62820	RAB1A_HUMAN	22678	RAB1A	Ras-related protein Rab-1A OS=Homo sapiens GN=RAB1A PE=1 SV=3	6	4	5	3	2	5	1
Q14568	HS902_HUMAN	39365	HSP90AA2	Putative heat shock protein HSP 90-alpha A2 OS=Homo sapiens GN=HSP90AA2 PE=1 SV=2	6	3	6	3	2	2	4
Q15417	CNN3_HUMAN	36414	CNN3	Calponin-3 OS=Homo sapiens GN=CNN3 PE=1 SV=1	6	4	3	3	2	5	3
Q16775-2	GLO2_HUMAN	28860	HAGH	Isoform 2 of Hydroxyacylglutathione hydrolase, mitochondrial OS=Homo sapiens GN=HAGH	6	3	2	5	8	1	1
Q16851-2	UGPA_HUMAN	55677	UGP2	Isoform 2 of UTP--glucose-1-phosphate uridylyltransferase OS=Homo sapiens GN=UGP2	6	5	3	3	3	2	4
Q96E39	RMXL1_HUMAN	42142	RBMXL1	RNA binding motif protein, X-linked-like-1 OS=Homo sapiens GN=RBMXL1 PE=1 SV=1	6	5	4	3	3	2	3
P13010	XRCC5_HUMAN	82705	XRCC5	X-ray repair cross-complementing protein 5 OS=Homo sapiens GN=XRCC5 PE=1 SV=3	5	3	4	5	4	4	
P30530-2	UFO_HUMAN	97377	AXL	Isoform Short of Tyrosine-protein kinase receptor UFO OS=Homo sapiens GN=AXL	5	3	2	6	3	6	
Q24JP5-2	T132A_HUMAN	110197	TMEM132A	Isoform 2 of Transmembrane protein 132A OS=Homo sapiens GN=TMEM132A	5	5	6	4	3		2
P30613-2	KPYR_HUMAN	58494	PKLR	Isoform L-type of Pyruvate kinase PKLR OS=Homo sapiens GN=PKLR	5	6	5		4	1	4
P41567	EIF1_HUMAN	12732	EIF1	Eukaryotic translation initiation factor 1 OS=Homo sapiens GN=EIF1 PE=1 SV=1	5	5	5		1	3	6
O95782-2	AP2A1_HUMAN	105361	AP2A1	Isoform B of AP-2 complex subunit alpha-1 OS=Homo sapiens GN=AP2A1	5	2		6	6	1	5
P49448	DHE4_HUMAN	61434	GLUD2	Glutamate dehydrogenase 2, mitochondrial OS=Homo sapiens GN=GLUD2 PE=1 SV=2	5	1		5	9	1	4
P53634	CATC_HUMAN	51854	CTSC	Dipeptidyl peptidase 1 OS=Homo sapiens GN=CTSC PE=1 SV=2	5	2		4	7	5	2
Q13228	SBP1_HUMAN	52391	SELENBP1	Selenium-binding protein 1 OS=Homo sapiens GN=SELENBP1 PE=1 SV=2	5	1		6	5	5	3
P78527-2	PRKDC_HUMAN	465501	PRKDC	Isoform 2 of DNA-dependent protein kinase catalytic subunit OS=Homo sapiens GN=PRKDC	4	3	2	7	8		
P15151-2	PVR_HUMAN	40125	PVR	Isoform Beta of Poliovirus receptor OS=Homo sapiens GN=PVR	4	4	6			5	5
Q9UHI8	ATS1_HUMAN	105358	ADAMTS1	A disintegrin and metalloproteinase with thrombospondin motifs 1 OS=Homo sapiens GN=ADAMTS1 PE=1 SV=4	4			4	4	6	6
P08134	RHOC_HUMAN	22006	RHOC	Rho-related GTP-binding protein RhoC OS=Homo sapiens GN=RHOC PE=1 SV=1	6	2	2	4	4	4	3
P39687	AN32A_HUMAN	28585	ANP32A	Acidic leucine-rich nuclear phosphoprotein 32 family member A OS=Homo sapiens GN=ANP32A PE=1 SV=1	6	3	3	4	4	4	1
P55957-2	BID_HUMAN	26836	BID	Isoform 2 of BH3-interacting domain death agonist OS=Homo sapiens GN=BID	6	4	5	3	3	1	3
P62993	GRB2_HUMAN	25206	GRB2	Growth factor receptor-bound protein 2 OS=Homo sapiens GN=GRB2 PE=1 SV=1	6	1	4	6	5	2	1

P63167	DYL1_HUMAN	10366	DYNLL1	Dynein light chain 1, cytoplasmic OS=Homo sapiens GN=DYNLL1 PE=1 SV=1	6	5	1	1	3	6	3
Q00839	HNRPU_HUMAN	90584	HNRNPU	Heterogeneous nuclear ribonucleoprotein U OS=Homo sapiens GN=HNRNPU PE=1 SV=6	6	1	2	5	8	2	1
Q03591	FHR1_HUMAN	37651	CFHR1	Complement factor H-related protein 1 OS=Homo sapiens GN=CFHR1 PE=1 SV=2	6	3	4	3	3	4	2
Q58FG0	HS905_HUMAN	38738	HSP90AA5P	Putative heat shock protein HSP 90-alpha A5 OS=Homo sapiens GN=HSP90AA5P PE=1 SV=1	6	2	4	4	3	3	3
Q99536	VAT1_HUMAN	41920	VAT1	Synaptic vesicle membrane protein VAT-1 homolog OS=Homo sapiens GN=VAT1 PE=1 SV=2	6	2	3	3	4	2	5
Q9HAV0	GBB4_HUMAN	37567	GNB4	Guanine nucleotide-binding protein subunit beta-4 OS=Homo sapiens GN=GNB4 PE=1 SV=3	6	2	1	4	5	4	3
Q99598	TSNAX_HUMAN	33112	TSNAX	Translin-associated protein X OS=Homo sapiens GN=TSNAX PE=1 SV=1	5	3	3	6	5		2
P15559-2	NQO1_HUMAN	27295	NQO1	Isoform 2 of NAD(P)H dehydrogenase [quinone] 1 OS=Homo sapiens GN=NQO1	5	3		5	5	3	3
P19320	VCAM1_HUMAN	81276	VCAM1	Vascular cell adhesion protein 1 OS=Homo sapiens GN=VCAM1 PE=1 SV=1	4	3	7			5	4
Q2TAA2	IAH1_HUMAN	27599	IAH1	Isoamyl acetate-hydrolyzing esterase 1 homolog OS=Homo sapiens GN=IAH1 PE=1 SV=1	4			5	9	3	2
Q96SM3	CPXM1_HUMAN	81668	CPXM1	Probable carboxypeptidase X1 OS=Homo sapiens GN=CPXM1 PE=2 SV=2	4			8	5	5	1
Q9BRK3-2	MXRA8_HUMAN	49620	MXRA8	Isoform 2 of Matrix-remodeling-associated protein 8 OS=Homo sapiens GN=MXRA8	4			7	7	2	3
Q9UKY7	CDV3_HUMAN	27335	CDV3	Protein CDV3 homolog OS=Homo sapiens GN=CDV3 PE=1 SV=1	4			4	8	3	4
Q14624-2	ITIH4_HUMAN	101241	ITIH4	Isoform 2 of Inter-alpha-trypsin inhibitor heavy chain H4 OS=Homo sapiens GN=ITIH4	3		5	7	7		
A6NDG6	PGP_HUMAN	34006	PGP	Phosphoglycolate phosphatase OS=Homo sapiens GN=PGP PE=1 SV=1	6	2	2	5	1	5	3
O76003	GLRX3_HUMAN	37432	GLRX3	Glutaredoxin-3 OS=Homo sapiens GN=GLRX3 PE=1 SV=2	6	3	2	2	2	4	5
O95433	AHSA1_HUMAN	38274	AHSA1	Activator of 90 kDa heat shock protein ATPase homolog 1 OS=Homo sapiens GN=AHSA1 PE=1 SV=1	6	1	2	4	4	5	2
P02649	APOE_HUMAN	36154	APOE	Apolipoprotein E OS=Homo sapiens GN=APOE PE=1 SV=1	6	3	3	1	3	5	3
P05114	HMGN1_HUMAN	10659	HMGN1	Non-histone chromosomal protein HMG-14 OS=Homo sapiens GN=HMGN1 PE=1 SV=3	6	1	2	4	4	3	4
P07196	NFL_HUMAN	61517	NEFL	Neurofilament light polypeptide OS=Homo sapiens GN=NEFL PE=1 SV=3	6	5	3	2	1	4	3
P07197-2	NFM_HUMAN	59599	NEFM	Isoform 2 of Neurofilament medium polypeptide OS=Homo sapiens GN=NEFM	6	5	3	2	1	4	3
P15586	GNS_HUMAN	62082	GNS	N-acetylglucosamine-6-sulfatase OS=Homo sapiens GN=GNS PE=1 SV=3	6	2	2	5	5	3	1
P16234-3	PGFRA_HUMAN	82809	PDGFRA	Isoform 3 of Platelet-derived growth factor receptor alpha OS=Homo sapiens GN=PDGFRA	6	4	3	1	1	3	6
P19827	ITIH1_HUMAN	101389	ITIH1	Inter-alpha-trypsin inhibitor heavy chain H1 OS=Homo sapiens GN=ITIH1 PE=1 SV=3	6	2	2	6	5	1	2
P61960	UFM1_HUMAN	9118	UFM1	Ubiquitin-fold modifier 1 OS=Homo sapiens GN=UFM1 PE=1 SV=1	6	3	2	1	3	4	5
Q16352	AINX_HUMAN	55391	INA	Alpha-internexin OS=Homo sapiens GN=INA PE=1 SV=2	6	5	3	2	1	4	3
Q96DG6	CMBL_HUMAN	28048	CMBL	Carboxymethylenebutenolidase homolog OS=Homo sapiens GN=CMBL PE=1 SV=1	6	2	3	5	6	1	1
Q96FQ6	S10AG_HUMAN	11801	S100A16	Protein S100-A16 OS=Homo sapiens GN=S100A16 PE=1 SV=1	6	3	2	3	4	4	2
Q9NQR4	NIT2_HUMAN	30608	NIT2	Omega-amidase NIT2 OS=Homo sapiens GN=NIT2 PE=1 SV=1	6	2	2	5	5	2	2
Q9UN70-2	PCDGK_HUMAN	94458	PCDHGC3	Isoform 2 of Protocadherin gamma-C3 OS=Homo sapiens GN=PCDHGC3	6	6	3	1	1	3	4
Q9Y3F4	STRAP_HUMAN	38438	STRAP	Serine-threonine kinase receptor-associated protein OS=Homo sapiens GN=STRAP PE=1 SV=1	6	5	1	2	3	4	3
E9PL57	E9PL57_HUMAN	19536	NEDD8-MDP1	Protein NEDD8-MDP1 (Fragment) OS=Homo sapiens GN=NEDD8-MDP1 PE=2 SV=1	6	4	2	3	3	3	3
H0YBX2	H0YBX2_HUMAN	22671	TATDN1	Putative deoxyribonuclease TATDN1 (Fragment) OS=Homo sapiens GN=TATDN1 PE=4 SV=1	6	6	3	3	1	2	3
Q6MZI6	Q6MZI6_HUMAN	11295	DKFZp686D09174	Proteasome (Prosome, macropain) subunit, alpha type, 2, isoform CRA_a OS=Homo sapiens GN=DKFZp686D09174 PE=2 SV=1	6	4	2	3	3	3	3
Q04446	GLGB_HUMAN	80474	GBE1	1,4-alpha-glucan-branching enzyme OS=Homo sapiens GN=GBE1 PE=1 SV=3	5	2	2	5	6	3	
Q15717	ELAV1_HUMAN	36092	ELAVL1	ELAV-like protein 1 OS=Homo sapiens GN=ELAVL1 PE=1 SV=2	5	3	3	4	5	3	
Q96IU4	ABHEB_HUMAN	22346	ABHD14B	Alpha/beta hydrolase domain-containing protein 14B OS=Homo sapiens GN=ABHD14B PE=1 SV=1	5	2	4	4	6	2	
P00450	CERU_HUMAN	122205	CP	Ceruloplasmin OS=Homo sapiens GN=CP PE=1 SV=1	5	1	3	6	4		4

Q15393	SF3B3_HUMAN	135577	SF3B3	Splicing factor 3B subunit 3 OS=Homo sapiens GN=SF3B3 PE=1 SV=4	5	2	3	6	6		1
P19883-2	FST_HUMAN	34803	FST	Isoform 2 of Follistatin OS=Homo sapiens GN=FST	5	1		3	4	3	7
P62280	RS11_HUMAN	18431	RPS11	40S ribosomal protein S11 OS=Homo sapiens GN=RPS11 PE=1 SV=3	5	2		5	4	5	2
Q9NWV4	CA123_HUMAN	18048	C1orf123	UPF0587 protein C1orf123 OS=Homo sapiens GN=C1orf123 PE=1 SV=1	5	3		5	4	3	3
Q5SYT8	Q5SYT8_HUMAN	53393	NAMPTL	Protein NAMPTL (Fragment) OS=Homo sapiens GN=NAMPTL PE=2 SV=1	5	2		4	5	3	4
Q6P587-2	FAHD1_HUMAN	24910	FAHD1	Isoform 2 of Acylpyruvase FAHD1, mitochondrial OS=Homo sapiens GN=FAHD1	5		1	6	5	3	3
Q96S86	HPLN3_HUMAN	40894	HAPLN3	Hyaluronan and proteoglycan link protein 3 OS=Homo sapiens GN=HAPLN3 PE=2 SV=1	4	4	10			1	3
Q99969	RARR2_HUMAN	18618	RARRES2	Retinoic acid receptor responder protein 2 OS=Homo sapiens GN=RARRES2 PE=1 SV=1	4	4	2			6	6
O95881	TXD12_HUMAN	19206	TXNDC12	Thioredoxin domain-containing protein 12 OS=Homo sapiens GN=TXNDC12 PE=1 SV=1	4	3		5	5	5	
P05362	ICAM1_HUMAN	57825	ICAM1	Intercellular adhesion molecule 1 OS=Homo sapiens GN=ICAM1 PE=1 SV=2	3	8	9				1
Q8NCH0	CHSTE_HUMAN	42997	CHST14	Carbohydrate sulfotransferase 14 OS=Homo sapiens GN=CHST14 PE=1 SV=2	3		2			6	10
P55268	LAMB2_HUMAN	195981	LAMB2	Laminin subunit beta-2 OS=Homo sapiens GN=LAMB2 PE=1 SV=2	3			1		12	5
A8MUU1	FB5L3_HUMAN	11299	FABP5P3	Putative fatty acid-binding protein 5-like protein 3 OS=Homo sapiens GN=FABP5P3 PE=5 SV=1	6	3	5	3	2	1	3
O75390	CISY_HUMAN	51712	CS	Citrate synthase, mitochondrial OS=Homo sapiens GN=CS PE=1 SV=2	6	2	2	4	3	3	3
P54578-2	UBP14_HUMAN	52386	USP14	Isoform 2 of Ubiquitin carboxyl-terminal hydrolase 14 OS=Homo sapiens GN=USP14	6	3	4	2	3	2	3
P68402	PA1B2_HUMAN	25569	PAFAH1B2	Platelet-activating factor acetylhydrolase IB subunit beta OS=Homo sapiens GN=PAFAH1B2 PE=1 SV=1	6	3	2	3	4	3	2
Q04837	SSBP_HUMAN	17260	SSBP1	Single-stranded DNA-binding protein, mitochondrial OS=Homo sapiens GN=SSBP1 PE=1 SV=1	6	3	1	6	5	1	1
Q12904-2	AIMP1_HUMAN	37039	AIMP1	Isoform 2 of Aminoacyl tRNA synthase complex-interacting multifunctional protein 1 OS=Homo sapiens GN=AIMP1	6	1	2	5	5	1	3
Q6EEV6	SUMO4_HUMAN	10685	SUMO4	Small ubiquitin-related modifier 4 OS=Homo sapiens GN=SUMO4 PE=1 SV=2	6	3	5	2	3	2	2
Q8IWE2	NXP20_HUMAN	60742	FAM114A1	Protein NOXP20 OS=Homo sapiens GN=FAM114A1 PE=1 SV=2	6	2	2	2	3	3	5
Q8WXX5	DNJC9_HUMAN	29910	DNAJC9	DnaJ homolog subfamily C member 9 OS=Homo sapiens GN=DNAJC9 PE=1 SV=1	6	2	4	3	3	1	4
Q96QV6	H2A1A_HUMAN	14234	HIST1H2AA	Histone H2A type 1-A OS=Homo sapiens GN=HIST1H2AA PE=1 SV=3	6	4	4	2	2	3	2
Q9H2H8	PPIL3_HUMAN	18155	PPIL3	Peptidyl-prolyl cis-trans isomerase-like 3 OS=Homo sapiens GN=PPIL3 PE=1 SV=1	6	4	3	2	2	3	3
Q9NP97	DLRB1_HUMAN	10922	DYNLRB1	Dynein light chain roadblock-type 1 OS=Homo sapiens GN=DYNLRB1 PE=1 SV=3	6	2	2	2	3	6	2
Q9P1F3	ABRAL_HUMAN	9056	ABRACL	Costars family protein ABRACL OS=Homo sapiens GN=ABRACL PE=1 SV=1	6	2	2	3	3	2	5
Q9Y547	IFT25_HUMAN	16297	HSPB11	Intraflagellar transport protein 25 homolog OS=Homo sapiens GN=HSPB11 PE=1 SV=1	6	1	2	3	3	4	4
Q6MZV4	Q6MZV4_HUMAN	39993	DKFZp686F20250	Protein NOXP20 OS=Homo sapiens GN=DKFZp686F20250 PE=2 SV=1	6	2	2	2	3	3	5
Q5JPC9	Q5JPC9_HUMAN	110681	DKFZp667H216	ABI gene family, member 3 (NESH) binding protein, isoform CRA_d OS=Homo sapiens GN=DKFZp667H216 PE=2 SV=1	5	2	2	4	4	5	
P04040	CATA_HUMAN	59756	CAT	Catalase OS=Homo sapiens GN=CAT PE=1 SV=3	5	3	2	7	3		2
O60506-2	HNRPQ_HUMAN	65682	SYNCRIP	Isoform 2 of Heterogeneous nuclear ribonucleoprotein Q OS=Homo sapiens GN=SYNCRIP	5	5	2	3		2	5
O14907	TX1B3_HUMAN	13735	TAX1BP3	Tax1-binding protein 3 OS=Homo sapiens GN=TAX1BP3 PE=1 SV=2	5	4		3	2	3	5
Q8IV08	PLD3_HUMAN	54705	PLD3	Phospholipase D3 OS=Homo sapiens GN=PLD3 PE=1 SV=1	5	2		3	3	4	5
Q9NX46	ARHL2_HUMAN	38947	ADPRHL2	Poly(ADP-ribose) glycohydrolase ARH3 OS=Homo sapiens GN=ADPRHL2 PE=1 SV=1	5	4		2	5	2	4
Q9UMY4-2	SNX12_HUMAN	18885	SNX12	Isoform 2 of Sorting nexin-12 OS=Homo sapiens GN=SNX12	5	4		2	3	5	3
P54826	GAS1_HUMAN	35693	GAS1	Growth arrest-specific protein 1 OS=Homo sapiens GN=GAS1 PE=1 SV=2	4	1	4			7	5
P98179	RBM3_HUMAN	17170	RBM3	Putative RNA-binding protein 3 OS=Homo sapiens GN=RBM3 PE=1 SV=1	4	5	4			5	3
O95497	VNN1_HUMAN	57012	VNN1	Pantetheinase OS=Homo sapiens GN=VNN1 PE=1 SV=2	4			4	5	4	4
Q9H4D0	CSTN2_HUMAN	107006	CLSTN2	Calsyntenin-2 OS=Homo sapiens GN=CLSTN2 PE=1 SV=2	2	6	11				
P16104	H2AX_HUMAN	15145	H2AFX	Histone H2AX OS=Homo sapiens GN=H2AFX PE=1 SV=2	6	3	4	2	2	3	2

P17301	ITA2_HUMAN	129295	ITGA2	Integrin alpha-2 OS=Homo sapiens GN=ITGA2 PE=1 SV=1	6	3	2	4	3	3	1
P27658	CO8A1_HUMAN	73364	COL8A1	Collagen alpha-1(VIII) chain OS=Homo sapiens GN=COL8A1 PE=1 SV=2	6	3	2	3	4	2	2
P40261	NNMT_HUMAN	29574	NNMT	Nicotinamide N-methyltransferase OS=Homo sapiens GN=NNMT PE=1 SV=1	6	2	5	1	2	3	3
P43121	MUC18_HUMAN	71607	MCAM	Cell surface glycoprotein MUC18 OS=Homo sapiens GN=MCAM PE=1 SV=2	6	1	5	1	1	4	4
P51858-2	HDGF_HUMAN	25538	HDGF	Isoform 2 of Hepatoma-derived growth factor OS=Homo sapiens GN=HDGF	6	3	2	3	4	1	3
P61077-2	UB2D3_HUMAN	16785	UBE2D3	Isoform 2 of Ubiquitin-conjugating enzyme E2 D3 OS=Homo sapiens GN=UBE2D3	6	4	3	1	1	4	3
P62837-2	UB2D2_HUMAN	13635	UBE2D2	Isoform 2 of Ubiquitin-conjugating enzyme E2 D2 OS=Homo sapiens GN=UBE2D2	6	4	3	1	1	4	3
P63000-2	RAC1_HUMAN	23467	RAC1	Isoform B of Ras-related C3 botulinum toxin substrate 1 OS=Homo sapiens GN=RAC1	6	3	3	3	1	3	3
Q15185	TEBP_HUMAN	18697	PTGES3	Prostaglandin E synthase 3 OS=Homo sapiens GN=PTGES3 PE=1 SV=1	6	2	4	3	2	2	3
Q15843	NEDD8_HUMAN	9072	NEDD8	NEDD8 OS=Homo sapiens GN=NEDD8 PE=1 SV=1	6	4	2	3	2	2	3
Q32MZ4-2	LRRF1_HUMAN	86404	LRRFIP1	Isoform 2 of Leucine-rich repeat flightless-interacting protein 1 OS=Homo sapiens GN=LRRFIP1	6	3	3	5	2	1	2
Q9H3K6	BOLA2_HUMAN	10117	BOLA2	BolA-like protein 2 OS=Homo sapiens GN=BOLA2 PE=1 SV=1	6	5	4	1	1	3	2
P49321	NASP_HUMAN	85238	NASP	Nuclear autoantigenic sperm protein OS=Homo sapiens GN=NASP PE=1 SV=2	5	3	3	6	2	2	
O75487	GPC4_HUMAN	62412	GPC4	Glypican-4 OS=Homo sapiens GN=GPC4 PE=1 SV=4	5	4	4	1		2	5
Q9HAT2	SIAE_HUMAN	58315	SIAE	Sialate O-acetylesterase OS=Homo sapiens GN=SIAE PE=1 SV=1	5	3	4		1	2	6
O00148	DX39A_HUMAN	49130	DDX39A	ATP-dependent RNA helicase DDX39A OS=Homo sapiens GN=DDX39A PE=1 SV=2	5	3		4	2	3	4
O00154-4	BACH_HUMAN	37420	ACOT7	Isoform 4 of Cytosolic acyl coenzyme A thioester hydrolase OS=Homo sapiens GN=ACOT7	5	3		3	5	1	4
P04899-2	GNAI2_HUMAN	38473	GNAI2	Isoform 2 of Guanine nucleotide-binding protein G(i) subunit alpha-2 OS=Homo sapiens GN=GNAI2	5	4		3	3	3	3
Q9NTK5	OLA1_HUMAN	44744	OLA1	Obg-like ATPase 1 OS=Homo sapiens GN=OLA1 PE=1 SV=2	5	2		4	3	3	4
O75828	CBR3_HUMAN	30850	CBR3	Carbonyl reductase [NADPH] 3 OS=Homo sapiens GN=CBR3 PE=1 SV=3	5		1	6	5	1	3
P48739-2	PIPNB_HUMAN	31638	PITPNB	Isoform 2 of Phosphatidylinositol transfer protein beta isoform OS=Homo sapiens GN=PITPNB	5		1	3	3	5	4
O43399-2	TPD54_HUMAN	19901	TPD52L2	Isoform 2 of Tumor protein D54 OS=Homo sapiens GN=TPD52L2	4	5	4			3	4
Q9NPR2-2	SEM4B_HUMAN	81403	SEMA4B	Isoform 2 of Semaphorin-4B OS=Homo sapiens GN=SEMA4B	4	4	6			3	3
P10586-2	PTPRF_HUMAN	211687	PTPRF	Isoform 2 of Receptor-type tyrosine-protein phosphatase F OS=Homo sapiens GN=PTPRF	4			3	2	6	5
P49767	VEGFC_HUMAN	46883	VEGFC	Vascular endothelial growth factor C OS=Homo sapiens GN=VEGFC PE=1 SV=1	4			4	3	4	5
Q01813	K6PP_HUMAN	85596	PFKP	6-phosphofructokinase type C OS=Homo sapiens GN=PFKP PE=1 SV=2	4			5	5	5	1
O43617	TPPC3_HUMAN	20274	TRAPPC3	Trafficking protein particle complex subunit 3 OS=Homo sapiens GN=TRAPPC3 PE=1 SV=1	6	4	3	1	3	2	2
P11279	LAMP1_HUMAN	44882	LAMP1	Lysosome-associated membrane glycoprotein 1 OS=Homo sapiens GN=LAMP1 PE=1 SV=3	6	3	4	2	2	2	2
P23246	SFPQ_HUMAN	76149	SFPQ	Splicing factor, proline- and glutamine-rich OS=Homo sapiens GN=SFPQ PE=1 SV=2	6	2	1	3	2	3	4
P31939-2	PUR9_HUMAN	64524	ATIC	Isoform 2 of Bifunctional purine biosynthesis protein PURH OS=Homo sapiens GN=ATIC	6	4	1	3	2	4	1
P32321-2	DCTD_HUMAN	21014	DCTD	Isoform 2 of Deoxycytidylate deaminase OS=Homo sapiens GN=DCTD	6	2	1	2	5	3	2
P35900	K1C20_HUMAN	48487	KRT20	Keratin, type I cytoskeletal 20 OS=Homo sapiens GN=KRT20 PE=1 SV=1	6	1	3	3	5	1	2
P53999	TCP4_HUMAN	14395	SUB1	Activated RNA polymerase II transcriptional coactivator p15 OS=Homo sapiens GN=SUB1 PE=1 SV=3	6	1	2	2	3	3	4
P60763	RAC3_HUMAN	21379	RAC3	Ras-related C3 botulinum toxin substrate 3 OS=Homo sapiens GN=RAC3 PE=1 SV=1	6	3	3	2	1	3	3
P62913-2	RL11_HUMAN	20124	RPL11	Isoform 2 of 60S ribosomal protein L11 OS=Homo sapiens GN=RPL11	6	3	1	4	2	2	3
Q14061	COX17_HUMAN	6915	COX17	Cytochrome c oxidase copper chaperone OS=Homo sapiens GN=COX17 PE=1 SV=2	6	2	2	1	3	4	3
Q14696	MESD_HUMAN	26077	MESDC2	LDLR chaperone MESD OS=Homo sapiens GN=MESDC2 PE=1 SV=2	6	2	3	2	4	3	1
Q5JXB2	UE2NL_HUMAN	17377	UBE2NL	Putative ubiquitin-conjugating enzyme E2 N-like OS=Homo sapiens GN=UBE2NL PE=1 SV=1	6	2	3	3	3	3	1
Q7Z406-2	MYH14_HUMAN	232011	MYH14	Isoform 2 of Myosin-14 OS=Homo sapiens GN=MYH14	6	1	2	4	4	1	3
Q8N1A0-2	KT222_HUMAN	29414	KRT222	Isoform 2 of Keratin-like protein KRT222 OS=Homo sapiens GN=KRT222	6	1	3	3	5	1	2
Q96A72	MGN2_HUMAN	17276	MAGOHB	Protein mago nashi homolog 2 OS=Homo sapiens GN=MAGOHB PE=1 SV=1	6	2	1	3	2	5	2

O75663	TIPRL_HUMAN	31444	TIPRL	TIP41-like protein OS=Homo sapiens GN=TIPRL PE=1 SV=2	5	1	1	5	6	2	
P08133	ANXA6_HUMAN	75873	ANXA6	Annexin A6 OS=Homo sapiens GN=ANXA6 PE=1 SV=3	5	2	2	5	4	2	
P46108	CRK_HUMAN	33831	CRK	Adapter molecule crk OS=Homo sapiens GN=CRK PE=1 SV=2	5	3	3	4	4	1	
Q13310-2	PABP4_HUMAN	69579	PABPC4	Isoform 2 of Polyadenylate-binding protein 4 OS=Homo sapiens GN=PABPC4	5	4	1	3	3	4	
Q99714	HCD2_HUMAN	26923	HSD17B10	3-hydroxyacyl-CoA dehydrogenase type-2 OS=Homo sapiens GN=HSD17B10 PE=1 SV=3	5	1	1	6	6	1	
C4P0D6	C4P0D6_HUMAN	21427	DISC1	Disrupted in schizophrenia 1 isoform 49 OS=Homo sapiens GN=DISC1 PE=2 SV=1	5	3	3	4	4		1
P54725	RD23A_HUMAN	39609	RAD23A	UV excision repair protein RAD23 homolog A OS=Homo sapiens GN=RAD23A PE=1 SV=1	5	4	3	3		2	3
O95861-2	BPNT1_HUMAN	35744	BPNT1	Isoform 2 of 3'(2'),5'-bisphosphate nucleotidase 1 OS=Homo sapiens GN=BPNT1	5	2		3	2	3	5
Q96EK6	GNA1_HUMAN	20749	GNPNAT1	Glucosamine 6-phosphate N-acetyltransferase OS=Homo sapiens GN=GNPNAT1 PE=1 SV=1	5	1		5	6	2	1
Q9Y617	SERC_HUMAN	40423	PSAT1	Phosphoserine aminotransferase OS=Homo sapiens GN=PSAT1 PE=1 SV=2	5	2		4	3	3	3
P52597	HNRPF_HUMAN	45672	HNRNPF	Heterogeneous nuclear ribonucleoprotein F OS=Homo sapiens GN=HNRNPF PE=1 SV=3	5		2	5	3	4	1
Q9P2B2	FPRP_HUMAN	98556	PTGFRN	Prostaglandin F2 receptor negative regulator OS=Homo sapiens GN=PTGFRN PE=1 SV=2	4	4	6	3	2		
P00568	KAD1_HUMAN	21635	AK1	Adenylate kinase isoenzyme 1 OS=Homo sapiens GN=AK1 PE=1 SV=3	4			4	5	4	2
Q13642-1	FHL1_HUMAN	31895	FHL1	Isoform 1 of Four and a half LIM domains protein 1 OS=Homo sapiens GN=FHL1	4			4	5	5	1
Q13867	BLMH_HUMAN	52562	BLMH	Bleomycin hydrolase OS=Homo sapiens GN=BLMH PE=1 SV=1	4			6	5	1	3
Q9HB71-3	CYBP_HUMAN	21228	CACYBP	Isoform 3 of Calcyclin-binding protein OS=Homo sapiens GN=CACYBP	4			6	6	2	1
P01857	IGHG1_HUMAN	36106	IGHG1	Ig gamma-1 chain C region OS=Homo sapiens GN=IGHG1 PE=1 SV=1	2			8	7		
Q6N022	TEN4_HUMAN	307957	TENM4	Teneurin-4 OS=Homo sapiens GN=TENM4 PE=1 SV=2	2			8	7		
P0C0S5	H2AZ_HUMAN	13553	H2AFZ	Histone H2A.Z OS=Homo sapiens GN=H2AFZ PE=1 SV=2	6	3	4	2	1	2	2
P19338	NUCL_HUMAN	76614	NCL	Nucleolin OS=Homo sapiens GN=NCL PE=1 SV=3	6	2	2	2	3	3	2
P27816-6	MAP4_HUMAN	119958	MAP4	Isoform 6 of Microtubule-associated protein 4 OS=Homo sapiens GN=MAP4	6	1	4	1	1	4	3
P45974-2	UBP5_HUMAN	93308	USP5	Isoform Short of Ubiquitin carboxyl-terminal hydrolase 5 OS=Homo sapiens GN=USP5	6	3	2	1	3	3	2
P50552	VASP_HUMAN	39830	VASP	Vasodilator-stimulated phosphoprotein OS=Homo sapiens GN=VASP PE=1 SV=3	6	2	1	1	3	3	4
P51148-2	RAB5C_HUMAN	27036	RAB5C	Isoform 2 of Ras-related protein Rab-5C OS=Homo sapiens GN=RAB5C	6	3	2	2	3	3	1
P53004	BIEA_HUMAN	33428	BLVRA	Biliverdin reductase A OS=Homo sapiens GN=BLVRA PE=1 SV=2	6	2	2	3	3	1	3
P61289-2	PSME3_HUMAN	30887	PSME3	Isoform 2 of Proteasome activator complex subunit 3 OS=Homo sapiens GN=PSME3	6	4	2	2	2	2	2
Q16576-2	RBBP7_HUMAN	52314	RBBP7	Isoform 2 of Histone-binding protein RBBP7 OS=Homo sapiens GN=RBBP7	6	2	2	1	3	3	3
Q71UI9-2	H2AV_HUMAN	12146	H2AFV	Isoform 2 of Histone H2A.V OS=Homo sapiens GN=H2AFV	6	3	4	2	1	2	2
Q86SX6	GLRX5_HUMAN	16628	GLRX5	Glutaredoxin-related protein 5, mitochondrial OS=Homo sapiens GN=GLRX5 PE=1 SV=2	6	1	3	2	2	3	3
Q8WUD1	RAB2B_HUMAN	24214	RAB2B	Ras-related protein Rab-2B OS=Homo sapiens GN=RAB2B PE=1 SV=1	6	3	2	2	2	3	2
Q99584	S10AD_HUMAN	11471	S100A13	Protein S100-A13 OS=Homo sapiens GN=S100A13 PE=1 SV=1	6	2	3	2	4	1	2
Q9H361	PABP3_HUMAN	70031	PABPC3	Polyadenylate-binding protein 3 OS=Homo sapiens GN=PABPC3 PE=1 SV=2	6	6	1	2	2	2	1
Q9H773	DCTP1_HUMAN	18681	DCTPP1	dCTP pyrophosphatase 1 OS=Homo sapiens GN=DCTPP1 PE=1 SV=1	6	2	1	4	4	1	2
Q9UBX5	FBLN5_HUMAN	50180	FBLN5	Fibulin-5 OS=Homo sapiens GN=FBLN5 PE=1 SV=1	6	3	2	1	1	5	2
P61224-3	RAP1B_HUMAN	18778	RAP1B	Isoform 3 of Ras-related protein Rap-1b OS=Homo sapiens GN=RAP1B	5	2	2	6	2	2	
Q12884	SEPR_HUMAN	87713	FAP	Seprase OS=Homo sapiens GN=FAP PE=1 SV=5	5	2	2	1		7	2
P98066	TSG6_HUMAN	31203	TNFAIP6	Tumor necrosis factor-inducible gene 6 protein OS=Homo sapiens GN=TNFAIP6 PE=1 SV=2	5	2	3		2	3	4
Q13409-2	DC1I2_HUMAN	70645	DYNC1I2	Isoform 2B of Cytoplasmic dynein 1 intermediate chain 2 OS=Homo sapiens GN=DYNC1I2	5	3	3		3	3	2
Q9UNZ2-5	NSF1C_HUMAN	40816	NSFL1C	Isoform 3 of NSFL1 cofactor p47 OS=Homo sapiens GN=NSFL1C	5	2	3		2	2	5
P11766	ADHX_HUMAN	39724	ADH5	Alcohol dehydrogenase class-3 OS=Homo sapiens GN=ADH5 PE=1 SV=4	5	2		2	4	4	2
P17050	NAGAB_HUMAN	46565	NAGA	Alpha-N-acetylgalactosaminidase OS=Homo sapiens GN=NAGA PE=1 SV=2	5	2		1	2	3	6
P22234-2	PUR6_HUMAN	47958	PAICS	Isoform 2 of Multifunctional protein ADE2 OS=Homo sapiens GN=PAICS	5	3		3	5	1	2

P30740	ILEU_HUMAN	42742	SERPINB1	Leukocyte elastase inhibitor OS=Homo sapiens GN=SERPINB1 PE=1 SV=1	5	2		4	2	2	4
P41091	IF2G_HUMAN	51109	EIF2S3	Eukaryotic translation initiation factor 2 subunit 3 OS=Homo sapiens GN=EIF2S3 PE=1 SV=3	5	2		2	4	2	4
Q01581	HMCS1_HUMAN	57294	HMGCS1	Hydroxymethylglutaryl-CoA synthase, cytoplasmic OS=Homo sapiens GN=HMGCS1 PE=1 SV=2	5	1		4	5	1	3
Q15257-2	PTPA_HUMAN	36775	PPP2R4	Isoform 1 of Serine/threonine-protein phosphatase 2A activator OS=Homo sapiens GN=PPP2R4	5	2		5	1	3	3
Q8N6T3-2	ARFG1_HUMAN	45676	ARFGAP1	Isoform 2 of ADP-ribosylation factor GTPase-activating protein 1 OS=Homo sapiens GN=ARFGAP1	5	1		4	3	3	3
P42126-2	ECI1_HUMAN	30896	ECI1	Isoform 2 of Enoyl-CoA delta isomerase 1, mitochondrial OS=Homo sapiens GN=ECI1	4	1	1	6	6		
P14210-3	HGF_HUMAN	82602	HGF	Isoform 3 of Hepatocyte growth factor OS=Homo sapiens GN=HGF	4	2	2			6	4
P58215	LOXL3_HUMAN	83166	LOXL3	Lysyl oxidase homolog 3 OS=Homo sapiens GN=LOXL3 PE=2 SV=1	4			5	4	2	3
Q13347	EIF3I_HUMAN	36502	EIF3I	Eukaryotic translation initiation factor 3 subunit I OS=Homo sapiens GN=EIF3I PE=1 SV=1	4			5	2	4	3
Q14257	RCN2_HUMAN	36876	RCN2	Reticulocalbin-2 OS=Homo sapiens GN=RCN2 PE=1 SV=1	4			5	3	1	5
Q92896-2	GSLG1_HUMAN	137222	GLG1	Isoform 2 of Golgi apparatus protein 1 OS=Homo sapiens GN=GLG1	4			6	3	1	4
O60507	TPST1_HUMAN	42188	TPST1	Protein-tyrosine sulfotransferase 1 OS=Homo sapiens GN=TPST1 PE=2 SV=1	3	1				4	9
P26927	HGFL_HUMAN	80320	MST1	Hepatocyte growth factor-like protein OS=Homo sapiens GN=MST1 PE=1 SV=2	3			6	7	1	
Q14517	FAT1_HUMAN	506273	FAT1	Protocadherin Fat 1 OS=Homo sapiens GN=FAT1 PE=1 SV=2	3			6	5	3	
P01859	IGHG2_HUMAN	35901	IGHG2	Ig gamma-2 chain C region OS=Homo sapiens GN=IGHG2 PE=1 SV=2	2			12	2		
B2RPK0	HGB1A_HUMAN	24238	HMGB1P1	Putative high mobility group protein B1-like 1 OS=Homo sapiens GN=HMGB1P1 PE=5 SV=1	6	2	1	3	4	2	1
O00764	PDXK_HUMAN	35102	PDXK	Pyridoxal kinase OS=Homo sapiens GN=PDXK PE=1 SV=1	6	3	2	3	1	2	2
O14737	PDCD5_HUMAN	14285	PDCD5	Programmed cell death protein 5 OS=Homo sapiens GN=PDCD5 PE=1 SV=3	6	1	4	2	1	3	2
P05455	LA_HUMAN	46837	SSB	Lupus La protein OS=Homo sapiens GN=SSB PE=1 SV=2	6	2	1	2	3	3	2
P08962-2	CD63_HUMAN	23430	CD63	Isoform 2 of CD63 antigen OS=Homo sapiens GN=CD63	6	1	3	2	1	3	3
P15153	RAC2_HUMAN	21429	RAC2	Ras-related C3 botulinum toxin substrate 2 OS=Homo sapiens GN=RAC2 PE=1 SV=1	6	3	3	1	1	3	2
P18827	SDC1_HUMAN	32462	SDC1	Syndecan-1 OS=Homo sapiens GN=SDC1 PE=1 SV=3	6	3	3	1	2	1	3
P31943	HNRH1_HUMAN	49229	HNRNPH1	Heterogeneous nuclear ribonucleoprotein H OS=Homo sapiens GN=HNRNPH1 PE=1 SV=4	6	2	3	2	1	3	2
P61923	COPZ1_HUMAN	20198	COPZ1	Coatomer subunit zeta-1 OS=Homo sapiens GN=COPZ1 PE=1 SV=1	6	3	2	2	3	2	1
P62701	RS4X_HUMAN	29598	RPS4X	40S ribosomal protein S4, X isoform OS=Homo sapiens GN=RPS4X PE=1 SV=2	6	3	2	2	2	1	3
Q8IUE6	H2A2B_HUMAN	13995	HIST2H2AB	Histone H2A type 2-B OS=Homo sapiens GN=HIST2H2AB PE=1 SV=3	6	3	3	1	2	2	2
Q9NRV9	HEBP1_HUMAN	21097	HEBP1	Heme-binding protein 1 OS=Homo sapiens GN=HEBP1 PE=1 SV=1	6	3	3	2	2	1	2
Q9Y333	LSM2_HUMAN	10835	LSM2	U6 snRNA-associated Sm-like protein LSm2 OS=Homo sapiens GN=LSM2 PE=1 SV=1	6	1	4	3	2	2	1
P62834	RAP1A_HUMAN	20987	RAP1A	Ras-related protein Rap-1A OS=Homo sapiens GN=RAP1A PE=1 SV=1	5	2	2	5	2	2	
O43583	DENR_HUMAN	22092	DENR	Density-regulated protein OS=Homo sapiens GN=DENR PE=1 SV=2	5	5	3	3	1		1
P20810-5	ICAL_HUMAN	81169	CAST	Isoform 5 of Calpastatin OS=Homo sapiens GN=CAST	5	1	1	5	4		2
P07311	ACYP1_HUMAN	11261	ACYP1	Acylphosphatase-1 OS=Homo sapiens GN=ACYP1 PE=1 SV=2	5	1		3	3	3	3
P49006	MRP_HUMAN	19529	MARCKSL1	MARCKS-related protein OS=Homo sapiens GN=MARCKSL1 PE=1 SV=2	5	1		3	2	1	6
P38919	IF4A3_HUMAN	46871	EIF4A3	Eukaryotic initiation factor 4A-III OS=Homo sapiens GN=EIF4A3 PE=1 SV=4	5		2	2	1	2	6
Q96QK1	VPS35_HUMAN	91707	VPS35	Vacuolar protein sorting-associated protein 35 OS=Homo sapiens GN=VPS35 PE=1 SV=2	5		2	3	3	4	1
O95450-2	ATS2_HUMAN	61756	ADAMTS2	Isoform SpNPI of A disintegrin and metalloproteinase with thrombospondin motifs 2 OS=Homo sapiens GN=ADAMTS2	4	4	1			3	5
Q9H7C9	AAMDC_HUMAN	13332	AAMDC	Mth938 domain-containing protein OS=Homo sapiens GN=AAMDC PE=1 SV=1	4	4	3			2	4
Q9H254-2	SPTN4_HUMAN	146169	SPTBN4	Isoform 2 of Spectrin beta chain, non-erythrocytic 4 OS=Homo sapiens GN=SPTBN4	4		4	4	3	2	
P49189	AL9A1_HUMAN	53802	ALDH9A1	4-trimethylaminobutyraldehyde dehydrogenase OS=Homo sapiens GN=ALDH9A1 PE=1 SV=3	4			4	2	3	4
P52926	HMGA2_HUMAN	11832	HMGA2	High mobility group protein HMGI-C OS=Homo sapiens GN=HMGA2 PE=1 SV=1	4			4	5	1	3
Q86V81	THOC4_HUMAN	26888	ALYREF	THO complex subunit 4 OS=Homo sapiens GN=ALYREF PE=1 SV=3	4			3	3	5	2

Q92747	ARC1A_HUMAN	41569	ARPC1A	Actin-related protein 2/3 complex subunit 1A OS=Homo sapiens GN=ARPC1A PE=1 SV=2	4			6	1	4	2
O94766	B3GA3_HUMAN	37122	B3GAT3	Galactosylgalactosylxylosylprotein 3-beta-glucuronosyltransferase 3 OS=Homo sapiens GN=B3GAT3 PE=1 SV=2	3	1				5	7
P26583	HMGB2_HUMAN	24034	HMGB2	High mobility group protein B2 OS=Homo sapiens GN=HMGB2 PE=1 SV=2	3			6	5	2	
P52566	GDIR2_HUMAN	22988	ARHGDIB	Rho GDP-dissociation inhibitor 2 OS=Homo sapiens GN=ARHGDIB PE=1 SV=3	3			6	6	1	
Q96JF0	SIAT2_HUMAN	60158	ST6GAL2	Beta-galactoside alpha-2,6-sialyltransferase 2 OS=Homo sapiens GN=ST6GAL2 PE=1 SV=2	2	8	5				
P22102	PUR2_HUMAN	107767	GART	Trifunctional purine biosynthetic protein adenosine-3 OS=Homo sapiens GN=GART PE=1 SV=1	2			8	5		
P36551	HEM6_HUMAN	50152	CPOX	Coproporphyrinogen-III oxidase, mitochondrial OS=Homo sapiens GN=CPOX PE=1 SV=3	2			7	6		
H7BYS2	H7BYS2_HUMAN	31537	SERPINB10	Serpin B10 (Fragment) OS=Homo sapiens GN=SERPINB10 PE=3 SV=1	2			8	5		
P41271-2	NBL1_HUMAN	23169	NBL1	Isoform 2 of Neuroblastoma suppressor of tumorigenicity 1 OS=Homo sapiens GN=NBL1	6	3	2	1	1	2	3
P62306	RUXF_HUMAN	9725	SNRPF	Small nuclear ribonucleoprotein F OS=Homo sapiens GN=SNRPF PE=1 SV=1	6	3	2	2	1	1	3
Q13643	FHL3_HUMAN	31192	FHL3	Four and a half LIM domains protein 3 OS=Homo sapiens GN=FHL3 PE=1 SV=4	6	2	2	1	3	1	3
Q15102	PA1B3_HUMAN	25734	PAFAH1B3	Platelet-activating factor acetylhydrolase IB subunit gamma OS=Homo sapiens GN=PAFAH1B3 PE=1 SV=1	6	3	2	1	2	2	2
Q8TF09	DLRB2_HUMAN	10855	DYNLRB2	Dynein light chain roadblock-type 2 OS=Homo sapiens GN=DYNLRB2 PE=1 SV=1	6	2	2	2	2	3	1
Q96A08	H2B1A_HUMAN	14167	HIST1H2BA	Histone H2B type 1-A OS=Homo sapiens GN=HIST1H2BA PE=1 SV=3	6	2	4	1	1	1	3
Q96AC1-2	FERM2_HUMAN	72397	FERMT2	Isoform 2 of Fermitin family homolog 2 OS=Homo sapiens GN=FERMT2	6	1	1	2	3	3	2
Q9P0L0	VAPA_HUMAN	27893	VAPA	Vesicle-associated membrane protein-associated protein A OS=Homo sapiens GN=VAPA PE=1 SV=3	6	2	2	2	3	1	2
Q9UBI6	GBG12_HUMAN	8006	GNG12	Guanine nucleotide-binding protein G(I)/G(S)/G(O) subunit gamma-12 OS=Homo sapiens GN=GNG12 PE=1 SV=3	6	2	3	2	2	2	1
O95479	G6PE_HUMAN	88893	H6PD	GDH/6PGL endoplasmic bifunctional protein OS=Homo sapiens GN=H6PD PE=1 SV=2	5	3	1	3	3	2	
P42574	CASP3_HUMAN	31608	CASP3	Caspase-3 OS=Homo sapiens GN=CASP3 PE=1 SV=2	5	1	3	4	2	2	
Q9HAV7	GRPE1_HUMAN	24279	GRPEL1	GrpE protein homolog 1, mitochondrial OS=Homo sapiens GN=GRPEL1 PE=1 SV=2	5	2	3	3	3	1	
P62328	TYB4_HUMAN	5053	TMSB4X	Thymosin beta-4 OS=Homo sapiens GN=TMSB4X PE=1 SV=2	5	3	4	1	2		2
Q5T4B6	Q5T4B6_HUMAN	5113	TMSB4XP4	HCG1780554 OS=Homo sapiens GN=TMSB4XP4 PE=4 SV=1	5	3	4	1	2		2
Q15370-2	ELOB_HUMAN	17911	TCEB2	Isoform 2 of Transcription elongation factor B polypeptide 2 OS=Homo sapiens GN=TCEB2	5	3	2	4		2	1
O43556-3	SGCE_HUMAN	48574	SGCE	Isoform 2 of Epsilon-sarcoglycan OS=Homo sapiens GN=SGCE	5	3	1		1	5	2
Q9H4G4	GAPR1_HUMAN	17218	GLIPR2	Golgi-associated plant pathogenesis-related protein 1 OS=Homo sapiens GN=GLIPR2 PE=1 SV=3	5	2	2		3	3	2
P09488-2	GSTM1_HUMAN	21253	GSTM1	Isoform 2 of Glutathione S-transferase Mu 1 OS=Homo sapiens GN=GSTM1	5	1		2	1	5	3
P45877	PPIC_HUMAN	22763	PPIC	Peptidyl-prolyl cis-trans isomerase C OS=Homo sapiens GN=PPIC PE=1 SV=1	5	1		2	2	4	3
Q03013-2	GSTM4_HUMAN	22829	GSTM4	Isoform 2 of Glutathione S-transferase Mu 4 OS=Homo sapiens GN=GSTM4	5	1		2	1	4	4
Q14011	CIRBP_HUMAN	18648	CIRBP	Cold-inducible RNA-binding protein OS=Homo sapiens GN=CIRBP PE=1 SV=1	5	2		1	1	5	3
Q8TEA8	DTD1_HUMAN	23424	DTD1	D-tyrosyl-tRNA(Tyr) deacylase 1 OS=Homo sapiens GN=DTD1 PE=1 SV=2	5	1		4	3	3	1
Q9BV57	MTND_HUMAN	21498	ADI1	1,2-dihydroxy-3-keto-5-methylthiopentene dioxygenase OS=Homo sapiens GN=ADI1 PE=1 SV=1	5	3		1	3	3	2
P62877	RBX1_HUMAN	12274	RBX1	E3 ubiquitin-protein ligase RBX1 OS=Homo sapiens GN=RBX1 PE=1 SV=1	5		2	2	1	3	4
Q9NVD7	PARVA_HUMAN	42244	PARVA	Alpha-parvin OS=Homo sapiens GN=PARVA PE=1 SV=1	5		2	3	4	2	1
Q12906-2	ILF3_HUMAN	76033	ILF3	Isoform 2 of Interleukin enhancer-binding factor 3 OS=Homo sapiens GN=ILF3	4	3	2	3	4		
P26572	MGAT1_HUMAN	50878	MGAT1	Alpha-1,3-mannosyl-glycoprotein 2-beta-N-acetylglucosaminyltransferase OS=Homo sapiens GN=MGAT1 PE=2 SV=2	4	2	2			3	5
Q6UX72	B3GN9_HUMAN	43751	B3GNT9	UDP-GlcNAc:betaGal beta-1,3-N-acetylglucosaminyltransferase 9 OS=Homo sapiens GN=B3GNT9 PE=2 SV=1	4	1	2			4	5
P35613-4	BASI_HUMAN	22764	BSG	Isoform 4 of Basigin OS=Homo sapiens GN=BSG	4		2	3	5		2
P62195-2	PRS8_HUMAN	44784	PSMC5	Isoform 2 of 26S protease regulatory subunit 8 OS=Homo sapiens GN=PSMC5	4			2	4	3	3

Q9GZT8-2	GTPC1_HUMAN	38984	NIF3L1	Isoform 2 of Putative GTP cyclohydrolase 1 type 2 NIF3L1 OS=Homo sapiens GN=NIF3L1	4			3	3	2	4
P07384	CAN1_HUMAN	81890	CAPN1	Calpain-1 catalytic subunit OS=Homo sapiens GN=CAPN1 PE=1 SV=1	3		1	5	6		
O14657	TOR1B_HUMAN	37979	TOR1B	Torsin-1B OS=Homo sapiens GN=TOR1B PE=1 SV=2	3		2			3	7
O75821	EIF3G_HUMAN	35611	EIF3G	Eukaryotic translation initiation factor 3 subunit G OS=Homo sapiens GN=EIF3G PE=1 SV=2	6	1	2	2	2	2	2
P17655	CAN2_HUMAN	79995	CAPN2	Calpain-2 catalytic subunit OS=Homo sapiens GN=CAPN2 PE=1 SV=6	6	1	1	2	2	4	1
P26006-1	ITA3_HUMAN	118756	ITGA3	Isoform 2 of Integrin alpha-3 OS=Homo sapiens GN=ITGA3	6	2	2	3	2	1	1
P61326	MGN_HUMAN	17164	MAGOH	Protein mago nashi homolog OS=Homo sapiens GN=MAGOH PE=1 SV=1	6	2	1	2	1	4	1
Q96F85-2	CNRP1_HUMAN	14224	CNRIP1	Isoform 2 of CB1 cannabinoid receptor-interacting protein 1 OS=Homo sapiens GN=CNRIP1	6	2	1	2	2	2	2
Q9NX55	HYPK_HUMAN	14665	HYPK	Huntingtin-interacting protein K OS=Homo sapiens GN=HYPK PE=1 SV=2	6	1	2	3	2	2	1
P22304	IDS_HUMAN	61873	IDS	Iduronate 2-sulfatase OS=Homo sapiens GN=IDS PE=1 SV=1	5	2	3	2	1	3	
Q92890-1	UFD1_HUMAN	38725	UFD1L	Isoform Long of Ubiquitin fusion degradation protein 1 homolog OS=Homo sapiens GN=UFD1L	5	1	2	3	3	2	
Q9H0U4	RAB1B_HUMAN	22171	RAB1B	Ras-related protein Rab-1B OS=Homo sapiens GN=RAB1B PE=1 SV=1	5	2	3	2	2	2	
Q9Y680-2	FKBP7_HUMAN	25794	FKBP7	Isoform 2 of Peptidyl-prolyl cis-trans isomerase FKBP7 OS=Homo sapiens GN=FKBP7	5	1	1	3	5	1	
A1L4H1	SRCRL_HUMAN	165743	SSC5D	Soluble scavenger receptor cysteine-rich domain-containing protein SSC5D OS=Homo sapiens GN=SSC5D PE=2 SV=3	5	1	1	2		3	4
P69849	NOMO3_HUMAN	134134	NOMO3	Nodal modulator 3 OS=Homo sapiens GN=NOMO3 PE=2 SV=2	5	2	1	1		2	5
Q15155	NOMO1_HUMAN	134324	NOMO1	Nodal modulator 1 OS=Homo sapiens GN=NOMO1 PE=1 SV=5	5	2	1	1		2	5
Q5JPE7-2	NOMO2_HUMAN	134192	NOMO2	Isoform 2 of Nodal modulator 2 OS=Homo sapiens GN=NOMO2	5	2	1	1		2	5
P47813	IF1AX_HUMAN	16460	EIF1AX	Eukaryotic translation initiation factor 1A, X-chromosomal OS=Homo sapiens GN=EIF1AX PE=1 SV=2	5	2		2	3	2	2
P50281	MMP14_HUMAN	65894	MMP14	Matrix metalloproteinase-14 OS=Homo sapiens GN=MMP14 PE=1 SV=3	5	1		2	3	2	3
Q8WUM4-2	PDC6I_HUMAN	96772	PDCD6IP	Isoform 2 of Programmed cell death 6-interacting protein OS=Homo sapiens GN=PDCD6IP	5	2		1	2	5	1
Q9H993	CF211_HUMAN	51172	C6orf211	UPF0364 protein C6orf211 OS=Homo sapiens GN=C6orf211 PE=1 SV=1	5	2		2	2	3	2
Q9NNW7-2	TRXR2_HUMAN	53819	TXNRD2	Isoform 2 of Thioredoxin reductase 2, mitochondrial OS=Homo sapiens GN=TXNRD2	5	2		4	1	2	2
Q8N1N4-2	K2C78_HUMAN	45017	KRT78	Isoform 2 of Keratin, type II cytoskeletal 78 OS=Homo sapiens GN=KRT78	4	1	2	4	4		
Q96HE7	ERO1A_HUMAN	54393	ERO1L	ERO1-like protein alpha OS=Homo sapiens GN=ERO1L PE=1 SV=2	4	4	2	2		3	
O60330-2	PCDGC_HUMAN	89582	PCDHGA12	Isoform 2 of Protocadherin gamma-A12 OS=Homo sapiens GN=PCDHGA12	4	2	2			2	5
Q58FG1	HS904_HUMAN	47712	HSP90AA4P	Putative heat shock protein HSP 90-alpha A4 OS=Homo sapiens GN=HSP90AA4P PE=5 SV=1	4	3	6			1	1
Q9Y646	CBPQ_HUMAN	51888	CPQ	Carboxypeptidase Q OS=Homo sapiens GN=CPQ PE=1 SV=1	4	1	1			6	3
Q16181-2	SEPT7_HUMAN	50581	SEPT7	Isoform 2 of Septin-7 OS=Homo sapiens GN=SEPT7	4	1		3	5	2	
O75937	DNJC8_HUMAN	29842	DNAJC8	DnaJ homolog subfamily C member 8 OS=Homo sapiens GN=DNAJC8 PE=1 SV=2	4		2	3	3	3	
P62244	RS15A_HUMAN	14840	RPS15A	40S ribosomal protein S15a OS=Homo sapiens GN=RPS15A PE=1 SV=2	4		2	2	3	4	
P31944	CASPE_HUMAN	27680	CASP14	Caspase-14 OS=Homo sapiens GN=CASP14 PE=1 SV=2	4			5	1	2	3
Q02952-2	AKA12_HUMAN	181690	AKAP12	Isoform 2 of A-kinase anchor protein 12 OS=Homo sapiens GN=AKAP12	3	5	4				2
P62906	RL10A_HUMAN	24831	RPL10A	60S ribosomal protein L10a OS=Homo sapiens GN=RPL10A PE=1 SV=2	3		2			6	3
P46109	CRKL_HUMAN	33777	CRKL	Crk-like protein OS=Homo sapiens GN=CRKL PE=1 SV=1	3			5	4	2	
Q8NCC3	PAG15_HUMAN	46658	PLA2G15	Group XV phospholipase A2 OS=Homo sapiens GN=PLA2G15 PE=1 SV=2	3			4	4		3
Q99757	THIOM_HUMAN	18383	TXN2	Thioredoxin, mitochondrial OS=Homo sapiens GN=TXN2 PE=1 SV=2	3			5		3	3
P54289-2	CA2D1_HUMAN	123183	CACNA2D1	Isoform 2 of Voltage-dependent calcium channel subunit alpha-2/delta-1 OS=Homo sapiens GN=CACNA2D1	2			6	5		
Q08554-2	DSC1_HUMAN	93835	DSC1	Isoform 1B of Desmocollin-1 OS=Homo sapiens GN=DSC1	2			6	5		
O75526	RMXL2_HUMAN	42814	RBMXL2	RNA-binding motif protein, X-linked-like-2 OS=Homo sapiens GN=RBMXL2 PE=1 SV=3	6	2	2	2	2	1	1
P19438	TNR1A_HUMAN	50495	TNFRSF1A	Tumor necrosis factor receptor superfamily member 1A OS=Homo sapiens GN=TNFRSF1A PE=1 SV=1	6	2	3	2	1	1	1

P43487	RANG_HUMAN	23310	RANBP1	Ran-specific GTPase-activating protein OS=Homo sapiens GN=RANBP1 PE=1 SV=1	6	1	1	2	2	2	2
P62745	RHOB_HUMAN	22123	RHOB	Rho-related GTP-binding protein RhoB OS=Homo sapiens GN=RHOB PE=1 SV=1	6	1	1	2	2	2	2
Q8WZ82	OVCA2_HUMAN	24418	OVCA2	Ovarian cancer-associated gene 2 protein OS=Homo sapiens GN=OVCA2 PE=1 SV=1	6	1	2	2	2	2	1
H3BV85	H3BV85_HUMAN	9743	BOLA2B	BolA-like protein 2 (Fragment) OS=Homo sapiens GN=BOLA2B PE=2 SV=1	6	3	3	1	1	1	1
Q14194-2	DPYL1_HUMAN	74262	CRMP1	Isoform LCRMP-1 of Dihydropyrimidinase-related protein 1 OS=Homo sapiens GN=CRMP1	5	2	3	3	1	1	
Q9GZZ1-2	NAA50_HUMAN	9465	NAA50	Isoform 2 of N-alpha-acetyltransferase 50 OS=Homo sapiens GN=NAA50	5	2	2	1		3	2
Q9UHV9	PFD2_HUMAN	16648	PFDN2	Prefoldin subunit 2 OS=Homo sapiens GN=PFDN2 PE=1 SV=1	5	3	4	1		1	1
O14602	IF1AY_HUMAN	16442	EIF1AY	Eukaryotic translation initiation factor 1A, Y-chromosomal OS=Homo sapiens GN=EIF1AY PE=1 SV=4	5	2		2	3	1	2
P19367-2	HXK1_HUMAN	102201	HK1	Isoform 2 of Hexokinase-1 OS=Homo sapiens GN=HK1	5	1		3	2	2	2
P62253	UB2G1_HUMAN	19509	UBE2G1	Ubiquitin-conjugating enzyme E2 G1 OS=Homo sapiens GN=UBE2G1 PE=1 SV=3	5	1		2	3	2	2
P62263	RS14_HUMAN	16273	RPS14	40S ribosomal protein S14 OS=Homo sapiens GN=RPS14 PE=1 SV=3	5	1		2	2	3	2
Q9UHD9	UBQL2_HUMAN	65696	UBQLN2	Ubiquilin-2 OS=Homo sapiens GN=UBQLN2 PE=1 SV=2	5	1		2	2	2	3
O60234	GMFG_HUMAN	16801	GMFG	Glia maturation factor gamma OS=Homo sapiens GN=GMFG PE=1 SV=1	5		1	2	2	4	1
Q96DC0	Q96DC0_HUMAN	26982	DCI	DCI protein OS=Homo sapiens GN=DCI PE=2 SV=1	4	1	1	5	3		
O60739	EIF1B_HUMAN	12824	EIF1B	Eukaryotic translation initiation factor 1b OS=Homo sapiens GN=EIF1B PE=1 SV=2	4	3	3			2	2
O95394-3	AGM1_HUMAN	62342	PGM3	Isoform 2 of Phosphoacetylglucosamine mutase OS=Homo sapiens GN=PGM3	4	2	3			4	1
Q9UKU9	ANGL2_HUMAN	57104	ANGPTL2	Angiopoietin-related protein 2 OS=Homo sapiens GN=ANGPTL2 PE=2 SV=1	4	1	2			3	4
O75063	XYLK_HUMAN	46432	FAM20B	Glycosaminoglycan xylosylkinase OS=Homo sapiens GN=FAM20B PE=1 SV=1	4	3		2	1	4	
Q96TA1-2	NIBL1_HUMAN	82683	FAM129B	Isoform 2 of Niban-like protein 1 OS=Homo sapiens GN=FAM129B	4	2		2	4	2	
O43765	SGTA_HUMAN	34063	SGTA	Small glutamine-rich tetratricopeptide repeat-containing protein alpha OS=Homo sapiens GN=SGTA PE=1 SV=1	4	4		2	2		2
P17813-2	EGLN_HUMAN	67542	ENG	Isoform Short of Endoglin OS=Homo sapiens GN=ENG	4		1	1	1	7	
P05413	FABPH_HUMAN	14858	FABP3	Fatty acid-binding protein, heart OS=Homo sapiens GN=FABP3 PE=1 SV=4	4			3	2	2	3
P06276	CHLE_HUMAN	68418	BCHE	Cholinesterase OS=Homo sapiens GN=BCHE PE=1 SV=1	4			3	3	3	1
P21246	PTN_HUMAN	18942	PTN	Pleiotrophin OS=Homo sapiens GN=PTN PE=1 SV=1	4			1	1	5	3
P62316	SMD2_HUMAN	13527	SNRPD2	Small nuclear ribonucleoprotein Sm D2 OS=Homo sapiens GN=SNRPD2 PE=1 SV=1	4			2	3	3	2
Q00169	PIPNA_HUMAN	31806	PITPNA	Phosphatidylinositol transfer protein alpha isoform OS=Homo sapiens GN=PITPNA PE=1 SV=2	4			3	3	3	1
Q9Y4G6	TLN2_HUMAN	271613	TLN2	Talin-2 OS=Homo sapiens GN=TLN2 PE=1 SV=4	4			4	2	1	3
P01011	AACT_HUMAN	47651	SERPINA3	Alpha-1-antichymotrypsin OS=Homo sapiens GN=SERPINA3 PE=1 SV=2	3	3		2		5	
P42785	PCP_HUMAN	55800	PRCP	Lysosomal Pro-X carboxypeptidase OS=Homo sapiens GN=PRCP PE=1 SV=1	3	3			1	6	
P31937	3HIDH_HUMAN	35329	HIBADH	3-hydroxyisobutyrate dehydrogenase, mitochondrial OS=Homo sapiens GN=HIBADH PE=1 SV=2	3		1	4	5		
Q9NP79	VTA1_HUMAN	33879	VTA1	Vacuolar protein sorting-associated protein VTA1 homolog OS=Homo sapiens GN=VTA1 PE=1 SV=1	3		3			4	3
P13591-1	NCAM1_HUMAN	93361	NCAM1	Isoform 2 of Neural cell adhesion molecule 1 OS=Homo sapiens GN=NCAM1	3			3	4	3	
P27708	PYR1_HUMAN	242984	CAD	CAD protein OS=Homo sapiens GN=CAD PE=1 SV=3	3			4	3	3	
Q9H2U2-2	IPYR2_HUMAN	39638	PPA2	Isoform 2 of Inorganic pyrophosphatase 2, mitochondrial OS=Homo sapiens GN=PPA2	3			3	5	2	
P09958	FURIN_HUMAN	86678	FURIN	Furin OS=Homo sapiens GN=FURIN PE=1 SV=2	3			2		2	6
Q9UFN0	NPS3A_HUMAN	28467	NIPSNAP3A	Protein NipSnap homolog 3A OS=Homo sapiens GN=NIPSNAP3A PE=1 SV=2	2			4	6		
P50895	BCAM_HUMAN	67405	BCAM	Basal cell adhesion molecule OS=Homo sapiens GN=BCAM PE=1 SV=2	2					5	5
P58107	EPIPL_HUMAN	555621	EPPK1	Epiplakin OS=Homo sapiens GN=EPPK1 PE=1 SV=2	6	2	2	2	1	1	1
P78318	IGBP1_HUMAN	39222	IGBP1	Immunoglobulin-binding protein 1 OS=Homo sapiens GN=IGBP1 PE=1 SV=1	6	1	1	1	1	2	3
Q13630	FCL_HUMAN	35893	TSTA3	GDP-L-fucose synthase OS=Homo sapiens GN=TSTA3 PE=1 SV=1	6	1	1	2	2	1	2
Q4VXU2	PAP1L_HUMAN	68392	PABPC1L	Polyadenylate-binding protein 1-like OS=Homo sapiens GN=PABPC1L PE=2 SV=1	6	3	1	1	1	2	1
Q96BJ3	AIDA_HUMAN	35023	AIDA	Axin interactor, dorsalization-associated protein OS=Homo sapiens GN=AIDA PE=1 SV=1	6	1	1	2	2	1	2
Q9NQ88	TIGAR_HUMAN	30063	TIGAR	Fructose-2,6-bisphosphatase TIGAR OS=Homo sapiens GN=TIGAR PE=1 SV=1	6	2	2	1	2	1	1

Q9P1U1-2	ARP3B_HUMAN	38031	ACTR3B	Isoform 2 of Actin-related protein 3B OS=Homo sapiens GN=ACTR3B	6	1	2	2	1	2	1
Q9UIJ7-2	KAD3_HUMAN	18323	AK3	Isoform 2 of GTP:AMP phosphotransferase AK3, mitochondrial OS=Homo sapiens GN=AK3	6	1	1	2	3	1	1
P61026	RAB10_HUMAN	22541	RAB10	Ras-related protein Rab-10 OS=Homo sapiens GN=RAB10 PE=1 SV=1	5	1	2	2	2	2	
Q9BPX5	ARP5L_HUMAN	16941	ARPC5L	Actin-related protein 2/3 complex subunit 5-like protein OS=Homo sapiens GN=ARPC5L PE=1 SV=1	5	1	1	2	2	3	
P35268	RL22_HUMAN	14787	RPL22	60S ribosomal protein L22 OS=Homo sapiens GN=RPL22 PE=1 SV=2	5	1		1	2	2	3
P54920	SNAA_HUMAN	33233	NAPA	Alpha-soluble NSF attachment protein OS=Homo sapiens GN=NAPA PE=1 SV=3	5	1		1	3	2	2
P61457	PHS_HUMAN	12000	PCBD1	Pterin-4-alpha-carbinolamine dehydratase OS=Homo sapiens GN=PCBD1 PE=1 SV=2	5	1		2	3	1	2
Q2VIR3	IF2GL_HUMAN	51229	EIF2S3L	Putative eukaryotic translation initiation factor 2 subunit 3-like protein OS=Homo sapiens GN=EIF2S3L PE=5 SV=2	5	1		1	3	2	2
Q9UBS4	DJB11_HUMAN	40514	DNAJB11	DnaJ homolog subfamily B member 11 OS=Homo sapiens GN=DNAJB11 PE=1 SV=1	5	1		1	1	2	4
O75173	ATS4_HUMAN	90197	ADAMTS4	A disintegrin and metalloproteinase with thrombospondin motifs 4 OS=Homo sapiens GN=ADAMTS4 PE=1 SV=3	4	2	2			2	3
P05023-3	AT1A1_HUMAN	109550	ATP1A1	Isoform 3 of Sodium/potassium-transporting ATPase subunit alpha-1 OS=Homo sapiens GN=ATP1A1	4	3	2			2	2
P16278-3	BGAL_HUMAN	72751	GLB1	Isoform 3 of Beta-galactosidase OS=Homo sapiens GN=GLB1	4	1	3			3	2
O75531	BAF_HUMAN	10059	BANF1	Barrier-to-autointegration factor OS=Homo sapiens GN=BANF1 PE=1 SV=1	4	3		2	2	2	
P61758	PFD3_HUMAN	22658	VBP1	Prefoldin subunit 3 OS=Homo sapiens GN=VBP1 PE=1 SV=3	4	1		2	3	3	
P50897	PPT1_HUMAN	34193	PPT1	Palmitoyl-protein thioesterase 1 OS=Homo sapiens GN=PPT1 PE=1 SV=1	4	2			1	3	3
Q5TFQ8	SIRBL_HUMAN	43359	SIRPB1	Signal-regulatory protein beta-1 isoform 3 OS=Homo sapiens GN=SIRPB1 PE=1 SV=1	4	1			2	3	3
Q8NHP8	PLBL2_HUMAN	65472	PLBD2	Putative phospholipase B-like 2 OS=Homo sapiens GN=PLBD2 PE=1 SV=2	4		3	2	1	3	
P78330	SERB_HUMAN	25008	PSPH	Phosphoserine phosphatase OS=Homo sapiens GN=PSPH PE=1 SV=2	4		1	4	2		2
Q8N612-2	F16A2_HUMAN	107081	FAM160A2	Isoform 2 of FTS and Hook-interacting protein OS=Homo sapiens GN=FAM160A2	4		3	2	3		1
Q9GZP0-2	PDGFD_HUMAN	42167	PDGFD	Isoform 2 of Platelet-derived growth factor D OS=Homo sapiens GN=PDGFD	4		2		1	2	4
P17931	LEG3_HUMAN	26152	LGALS3	Galectin-3 OS=Homo sapiens GN=LGALS3 PE=1 SV=5	4			1	3	3	2
P46777	RL5_HUMAN	34363	RPL5	60S ribosomal protein L5 OS=Homo sapiens GN=RPL5 PE=1 SV=3	4			3	3	1	2
P62140	PP1B_HUMAN	37187	PPP1CB	Serine/threonine-protein phosphatase PP1-beta catalytic subunit OS=Homo sapiens GN=PPP1CB PE=1 SV=3	4			3	2	3	1
Q14353	GAMT_HUMAN	26318	GAMT	Guanidinoacetate N-methyltransferase OS=Homo sapiens GN=GAMT PE=1 SV=1	4			3	3	2	1
Q9BY44	EIF2A_HUMAN	64990	EIF2A	Eukaryotic translation initiation factor 2A OS=Homo sapiens GN=EIF2A PE=1 SV=3	4			2	3	2	2
P41222	PTGDS_HUMAN	21029	PTGDS	Prostaglandin-H2 D-isomerase OS=Homo sapiens GN=PTGDS PE=1 SV=1	3	4	4			1	
P13637-2	AT1A3_HUMAN	113134	ATP1A3	Isoform 2 of Sodium/potassium-transporting ATPase subunit alpha-3 OS=Homo sapiens GN=ATP1A3	3	4	3				2
Q15046-2	SYK_HUMAN	71497	KARS	Isoform Mitochondrial of Lysine--tRNA ligase OS=Homo sapiens GN=KARS	3	2		3		4	
P09496-2	CLCA_HUMAN	23662	CLTA	Isoform Non-brain of Clathrin light chain A OS=Homo sapiens GN=CLTA	3		3			3	3
P03952	KLKB1_HUMAN	71370	KLKB1	Plasma kallikrein OS=Homo sapiens GN=KLKB1 PE=1 SV=1	3			4	3	2	
Q08188	TGM3_HUMAN	76632	TGM3	Protein-glutamine gamma-glutamyltransferase E OS=Homo sapiens GN=TGM3 PE=1 SV=4	3			6	2	1	
Q09328	MGT5A_HUMAN	84543	MGAT5	Alpha-1,6-mannosylglycoprotein 6-beta-N-acetylglucosaminyltransferase A OS=Homo sapiens GN=MGAT5 PE=1 SV=1	3			3	3	3	
Q13822-2	ENPP2_HUMAN	105201	ENPP2	Isoform 2 of Ectonucleotide pyrophosphatase/phosphodiesterase family member 2 OS=Homo sapiens GN=ENPP2	3			3	4	2	
Q9P258	RCC2_HUMAN	56085	RCC2	Protein RCC2 OS=Homo sapiens GN=RCC2 PE=1 SV=2	3			3	4	2	
P25705	ATPA_HUMAN	59751	ATP5A1	ATP synthase subunit alpha, mitochondrial OS=Homo sapiens GN=ATP5A1 PE=1 SV=1	3			3	3		3
P01042	KNG1_HUMAN	71957	KNG1	Kininogen-1 OS=Homo sapiens GN=KNG1 PE=1 SV=2	2			6	3		
P27707	DCK_HUMAN	30519	DCK	Deoxycytidine kinase OS=Homo sapiens GN=DCK PE=1 SV=1	2			3	6		
Q13011	ECH1_HUMAN	35816	ECH1	Delta(3,5)-Delta(2,4)-dienoyl-CoA isomerase, mitochondrial OS=Homo sapiens GN=ECH1 PE=1 SV=2	2			5	4		
Q8IUX7-2	AEBP1_HUMAN	82183	AEBP1	Isoform 2 of Adipocyte enhancer-binding protein 1 OS=Homo sapiens GN=AEBP1	2			5	4		

C9JCS0	C9JCS0_HUMAN	22375	NR1H3	Oxysterols receptor LXR-alpha (Fragment) OS=Homo sapiens GN=NR1H3 PE=2 SV=1	2			3	6		
P08473	NEP_HUMAN	85514	MME	Neprilysin OS=Homo sapiens GN=MME PE=1 SV=2	2					5	4
O15372	EIF3H_HUMAN	39930	EIF3H	Eukaryotic translation initiation factor 3 subunit H OS=Homo sapiens GN=EIF3H PE=1 SV=1	6	1	1	1	2	1	2
P55795	HNRH2_HUMAN	49264	HNRNPH2	Heterogeneous nuclear ribonucleoprotein H2 OS=Homo sapiens GN=HNRNPH2 PE=1 SV=1	6	2	2	1	1	1	1
Q06481-2	APLP2_HUMAN	80721	APLP2	Isoform 2 of Amyloid-like protein 2 OS=Homo sapiens GN=APLP2	6	1	2	2	1	1	1
Q13951-2	PEBB_HUMAN	21991	CBFB	Isoform 2 of Core-binding factor subunit beta OS=Homo sapiens GN=CBFB	6	1	1	2	1	2	1
Q16698	DECR_HUMAN	36068	DECR1	2,4-dienoyl-CoA reductase, mitochondrial OS=Homo sapiens GN=DECR1 PE=1 SV=1	6	1	2	2	1	1	1
Q9BTM9-2	URM1_HUMAN	15815	URM1	Isoform 2 of Ubiquitin-related modifier 1 OS=Homo sapiens GN=URM1	6	1	1	1	1	2	2
B3KS98	B3KS98_HUMAN	41581	EIF3S3	Eukaryotic translation initiation factor 3 subunit H OS=Homo sapiens GN=EIF3S3 PE=2 SV=1	6	1	1	1	2	1	2
P03950	ANGI_HUMAN	16550	ANG	Angiogenin OS=Homo sapiens GN=ANG PE=1 SV=1	5	2	2	1	1	2	
P41227-2	NAA10_HUMAN	24784	NAA10	Isoform 2 of N-alpha-acetyltransferase 10 OS=Homo sapiens GN=NAA10	5	2	1	1	3	1	
P61006	RAB8A_HUMAN	23668	RAB8A	Ras-related protein Rab-8A OS=Homo sapiens GN=RAB8A PE=1 SV=1	5	1	2	3	1	1	
Q92928	RAB1C_HUMAN	22017	RAB1C	Putative Ras-related protein Rab-1C OS=Homo sapiens GN=RAB1C PE=5 SV=2	5	1	2	2	1	2	
P49458	SRP09_HUMAN	10112	SRP9	Signal recognition particle 9 kDa protein OS=Homo sapiens GN=SRP9 PE=1 SV=2	5	2	2	1	1		2
Q03393	PTPS_HUMAN	16386	PTS	6-pyruvoyl tetrahydrobiopterin synthase OS=Homo sapiens GN=PTS PE=1 SV=1	5	1	2	1	3		1
B1ANH6	B1ANH6_HUMAN	26279	GUK1	Guanylate kinase OS=Homo sapiens GN=GUK1 PE=2 SV=1	5	1	3	1	2		1
Q99471	PFD5_HUMAN	17328	PFDN5	Prefoldin subunit 5 OS=Homo sapiens GN=PFDN5 PE=1 SV=2	5	1	3		1	1	2
O43684-2	BUB3_HUMAN	36955	BUB3	Isoform 2 of Mitotic checkpoint protein BUB3 OS=Homo sapiens GN=BUB3	5	2		1	2	1	2
P60520	GBRL2_HUMAN	13667	GABARAPL2	Gamma-aminobutyric acid receptor-associated protein-like 2 OS=Homo sapiens GN=GABARAPL2 PE=1 SV=1	5	1		1	1	2	3
P61812-2	TGFB2_HUMAN	50573	TGFB2	Isoform B of Transforming growth factor beta-2 OS=Homo sapiens GN=TGFB2	4	4	2	1	1		
O60493	SNX3_HUMAN	18762	SNX3	Sorting nexin-3 OS=Homo sapiens GN=SNX3 PE=1 SV=3	4	2	1			3	2
Q9NX62	IMPA3_HUMAN	38681	IMPAD1	Inositol monophosphatase 3 OS=Homo sapiens GN=IMPAD1 PE=1 SV=1	4	1	1			3	3
P41250	SYG_HUMAN	83166	GARS	Glycine--tRNA ligase OS=Homo sapiens GN=GARS PE=1 SV=3	4	1		2	1	4	
Q9NP84	TNR12_HUMAN	13911	TNFRSF12A	Tumor necrosis factor receptor superfamily member 12A OS=Homo sapiens GN=TNFRSF12A PE=1 SV=1	4	2		1	2	3	
P55884-2	EIF3B_HUMAN	99029	EIF3B	Isoform 2 of Eukaryotic translation initiation factor 3 subunit B OS=Homo sapiens GN=EIF3B	4	2		1	3		2
P83916	CBX1_HUMAN	21418	CBX1	Chromobox protein homolog 1 OS=Homo sapiens GN=CBX1 PE=1 SV=1	4	1		2	2		3
Q8NCL4	GALT6_HUMAN	71159	GALNT6	Polypeptide N-acetylgalactosaminyltransferase 6 OS=Homo sapiens GN=GALNT6 PE=2 SV=2	4	2			2	3	1
Q9BRP8-2	WIBG_HUMAN	22705	WIBG	Isoform 2 of Partner of Y14 and mago OS=Homo sapiens GN=WIBG	4	2			1	3	2
Q9NPH3-2	IL1AP_HUMAN	41019	IL1RAP	Isoform 2 of Interleukin-1 receptor accessory protein OS=Homo sapiens GN=IL1RAP	4	2			2	2	2
Q96GG9	DCNL1_HUMAN	30124	DCUN1D1	DCN1-like protein 1 OS=Homo sapiens GN=DCUN1D1 PE=1 SV=1	4		1	2	3	2	
Q16836-2	HCDH_HUMAN	42140	HADH	Isoform 2 of Hydroxyacyl-coenzyme A dehydrogenase, mitochondrial OS=Homo sapiens GN=HADH	4		2	2	2		2
Q6ZRP7	QSOX2_HUMAN	77529	QSOX2	Sulfhydryl oxidase 2 OS=Homo sapiens GN=QSOX2 PE=1 SV=3	4		1		2	4	1
P28482-2	MK01_HUMAN	36432	MAPK1	Isoform 2 of Mitogen-activated protein kinase 1 OS=Homo sapiens GN=MAPK1	4			2	3	2	1
Q13591	SEM5A_HUMAN	120615	SEMA5A	Semaphorin-5A OS=Homo sapiens GN=SEMA5A PE=1 SV=3	4			2	1	3	2
Q6UXH9-2	PAMR1_HUMAN	81943	PAMR1	Isoform 2 of Inactive serine protease PAMR1 OS=Homo sapiens GN=PAMR1	4			3	3	1	1
Q9NYL9	TMOD3_HUMAN	39595	TMOD3	Tropomodulin-3 OS=Homo sapiens GN=TMOD3 PE=1 SV=1	4			2	2	2	2
Q6UWH4-2	F198B_HUMAN	58507	FAM198B	Isoform 2 of Protein FAM198B OS=Homo sapiens GN=FAM198B	3	2				3	3
P49903	SPS1_HUMAN	42911	SEPHS1	Selenide, water dikinase 1 OS=Homo sapiens GN=SEPHS1 PE=1 SV=2	3		1	4	3		
Q9H0R4-2	HDHD2_HUMAN	18533	HDHD2	Isoform 2 of Haloacid dehalogenase-like hydrolase domain-containing protein 2 OS=Homo sapiens GN=HDHD2	3		1	3	4		
P49588	SYAC_HUMAN	106810	AARS	Alanine--tRNA ligase, cytoplasmic OS=Homo sapiens GN=AARS PE=1 SV=2	3		1			4	3
O14817	TSN4_HUMAN	26118	TSPAN4	Tetraspanin-4 OS=Homo sapiens GN=TSPAN4 PE=1 SV=1	3			3	3	2	

P09417	DHPR_HUMAN	25790	QDPR	Dihydropteridine reductase OS=Homo sapiens GN=QDPR PE=1 SV=2	3			4	2	2	
P13473-2	LAMP2_HUMAN	44956	LAMP2	Isoform LAMP-2B of Lysosome-associated membrane glycoprotein 2 OS=Homo sapiens GN=LAMP2	3			3	4	1	
P52788	SPSY_HUMAN	41268	SMS	Spermine synthase OS=Homo sapiens GN=SMS PE=1 SV=2	3			4	3	1	
P62241	RS8_HUMAN	24205	RPS8	40S ribosomal protein S8 OS=Homo sapiens GN=RPS8 PE=1 SV=2	3			3	3	2	
Q8IXB1-2	DJC10_HUMAN	86136	DNAJC10	Isoform 2 of DnaJ homolog subfamily C member 10 OS=Homo sapiens GN=DNAJC10	3			2	4	2	
O14558	HSPB6_HUMAN	17136	HSPB6	Heat shock protein beta-6 OS=Homo sapiens GN=HSPB6 PE=1 SV=2	3			1		5	2
Q9Y287	ITM2B_HUMAN	30338	ITM2B	Integral membrane protein 2B OS=Homo sapiens GN=ITM2B PE=1 SV=1	2	3				5	
O75581	LRP6_HUMAN	180429	LRP6	Low-density lipoprotein receptor-related protein 6 OS=Homo sapiens GN=LRP6 PE=1 SV=2	2			4	4		
Q6UWP8	SBSN_HUMAN	60541	SBSN	Suprabasin OS=Homo sapiens GN=SBSN PE=2 SV=2	2			5	3		
F5H736	F5H736_HUMAN	7003	NDUFS7	NADH dehydrogenase [ubiquinone] iron-sulfur protein 7, mitochondrial OS=Homo sapiens GN=NDUFS7 PE=2 SV=1	2				3		5
Q86UW8	HPLN4_HUMAN	42801	HAPLN4	Hyaluronan and proteoglycan link protein 4 OS=Homo sapiens GN=HAPLN4 PE=2 SV=1	1		8				
Q9BUP0-2	EFHD1_HUMAN	16145	EFHD1	Isoform 2 of EF-hand domain-containing protein D1 OS=Homo sapiens GN=EFHD1	6	2	1	1	1	1	1
P59190-2	RAB15_HUMAN	23518	RAB15	Isoform 2 of Ras-related protein Rab-15 OS=Homo sapiens GN=RAB15	5	1	2	1	1	2	
Q58FF3	ENPLL_HUMAN	45859	HSP90B2P	Putative endoplasmin-like protein OS=Homo sapiens GN=HSP90B2P PE=5 SV=1	5	2	1	1	1	2	
Q92930	RAB8B_HUMAN	23584	RAB8B	Ras-related protein Rab-8B OS=Homo sapiens GN=RAB8B PE=1 SV=2	5	1	2	2	1	1	
Q9H2D6-2	TARA_HUMAN	250724	TRIOBP	Isoform 3 of TRIO and F-actin-binding protein OS=Homo sapiens GN=TRIOBP	5	1	1	2	2	1	
Q9P121-2	NTRI_HUMAN	37954	NTM	Isoform 2 of Neurotrimin OS=Homo sapiens GN=NTM	5	1	2	1	1		2
Q9UPY8-2	MARE3_HUMAN	30380	MAPRE3	Isoform 2 of Microtubule-associated protein RP/EB family member 3 OS=Homo sapiens GN=MAPRE3	5	1	2	1	1		2
P20339	RAB5A_HUMAN	23659	RAB5A	Ras-related protein Rab-5A OS=Homo sapiens GN=RAB5A PE=1 SV=2	5	2	1		2	1	1
P61020-2	RAB5B_HUMAN	19090	RAB5B	Isoform 2 of Ras-related protein Rab-5B OS=Homo sapiens GN=RAB5B	5	2	1		2	1	1
O60613	SEP15_HUMAN	17640	SEP15	15 kDa selenoprotein OS=Homo sapiens GN=SEP15 PE=1 SV=3	5	1		2	1	1	2
Q9H3P7	GCP60_HUMAN	60593	ACBD3	Golgi resident protein GCP60 OS=Homo sapiens GN=ACBD3 PE=1 SV=4	5	1		2	2	1	1
O15400-2	STX7_HUMAN	27400	STX7	Isoform 2 of Syntaxin-7 OS=Homo sapiens GN=STX7	4	1	2	2	2		
Q9BXJ0	C1QT5_HUMAN	25298	C1QTNF5	Complement C1q tumor necrosis factor-related protein 5 OS=Homo sapiens GN=C1QTNF5 PE=1 SV=1	4	1	2	3	1		
P22307-2	NLTP_HUMAN	15401	SCP2	Isoform SCP2 of Non-specific lipid-transfer protein OS=Homo sapiens GN=SCP2	4	2	1		1	3	
Q13435	SF3B2_HUMAN	100228	SF3B2	Splicing factor 3B subunit 2 OS=Homo sapiens GN=SF3B2 PE=1 SV=2	4	2		1	2	2	
B4DFL2	B4DFL2_HUMAN	45180	IDH2	Isocitrate dehydrogenase [NADP] OS=Homo sapiens GN=IDH2 PE=2 SV=1	4	2		1	1		3
P08195-2	4F2_HUMAN	57945	SLC3A2	Isoform 2 of 4F2 cell-surface antigen heavy chain OS=Homo sapiens GN=SLC3A2	4	1		1		3	2
B1Q2N1	B1Q2N1_HUMAN	30488	DDX39	ATP-dependent RNA helicase DDX39A OS=Homo sapiens GN=DDX39 PE=2 SV=1	4	2		1		1	3
P18077	RL35A_HUMAN	12538	RPL35A	60S ribosomal protein L35a OS=Homo sapiens GN=RPL35A PE=1 SV=2	4	2			1	2	2
O95980	RECK_HUMAN	106457	RECK	Reversion-inducing cysteine-rich protein with Kazal motifs OS=Homo sapiens GN=RECK PE=1 SV=1	4		1	1		3	2
Q9Y4Y9	LSM5_HUMAN	9937	LSM5	U6 snRNA-associated Sm-like protein LSm5 OS=Homo sapiens GN=LSM5 PE=1 SV=3	4		2		2	2	1
O43390-3	HNRPR_HUMAN	66980	HNRNPR	Isoform 3 of Heterogeneous nuclear ribonucleoprotein R OS=Homo sapiens GN=HNRNPR	4			4	1	1	1
P10412	H14_HUMAN	21865	HIST1H1E	Histone H1.4 OS=Homo sapiens GN=HIST1H1E PE=1 SV=2	4			1	1	2	3
P16402	H13_HUMAN	22350	HIST1H1D	Histone H1.3 OS=Homo sapiens GN=HIST1H1D PE=1 SV=2	4			1	1	2	3
P16403	H12_HUMAN	21365	HIST1H1C	Histone H1.2 OS=Homo sapiens GN=HIST1H1C PE=1 SV=2	4			1	1	2	3
P36873-2	PP1G_HUMAN	38518	PPP1CC	Isoform Gamma-2 of Serine/threonine-protein phosphatase PP1-gamma catalytic subunit OS=Homo sapiens GN=PPP1CC	4			3	1	2	1
P46782	RS5_HUMAN	22876	RPS5	40S ribosomal protein S5 OS=Homo sapiens GN=RPS5 PE=1 SV=4	4			2	2	2	1
P62136-2	PP1A_HUMAN	38631	PPP1CA	Isoform 2 of Serine/threonine-protein phosphatase PP1-alpha catalytic subunit OS=Homo sapiens GN=PPP1CA	4			3	1	2	1
Q5SW79-2	CE170_HUMAN	161408	CEP170	Isoform 2 of Centrosomal protein of 170 kDa OS=Homo sapiens GN=CEP170	4			3	2	1	1
S4R435	S4R435_HUMAN	32596	RPS10-NUDT3	Protein RPS10-NUDT3 (Fragment) OS=Homo sapiens GN=RPS10-NUDT3 PE=3 SV=1	4			2	2	1	2

P12956	XRCC6_HUMAN	69843	XRCC6	X-ray repair cross-complementing protein 6 OS=Homo sapiens GN=XRCC6 PE=1 SV=2	3	2	3			2	
P14209-3	CD99_HUMAN	17128	CD99	Isoform 3 of CD99 antigen OS=Homo sapiens GN=CD99	3	2		2	3		
O75503	CLN5_HUMAN	41497	CLN5	Ceroid-lipofuscinosis neuronal protein 5 OS=Homo sapiens GN=CLN5 PE=1 SV=2	3	3				2	2
P21980	TGM2_HUMAN	77329	TGM2	Protein-glutamine gamma-glutamyltransferase 2 OS=Homo sapiens GN=TGM2 PE=1 SV=2	3	1				3	3
P51668	UB2D1_HUMAN	16602	UBE2D1	Ubiquitin-conjugating enzyme E2 D1 OS=Homo sapiens GN=UBE2D1 PE=1 SV=1	3	2				3	2
Q9Y2X8	UB2D4_HUMAN	16649	UBE2D4	Ubiquitin-conjugating enzyme E2 D4 OS=Homo sapiens GN=UBE2D4 PE=1 SV=1	3	2				3	2
P78371	TCPB_HUMAN	57488	CCT2	T-complex protein 1 subunit beta OS=Homo sapiens GN=CCT2 PE=1 SV=4	3			4	1	2	
Q7RTY9	PRS41_HUMAN	35078	PRSS41	Putative serine protease 41 OS=Homo sapiens GN=PRSS41 PE=5 SV=1	3			4	1	2	
Q92954-2	PRG4_HUMAN	146518	PRG4	Isoform B of Proteoglycan 4 OS=Homo sapiens GN=PRG4	3			2	3	2	
Q9Y6M0-2	TEST_HUMAN	34634	PRSS21	Isoform 2 of Testisin OS=Homo sapiens GN=PRSS21	3			4	1	2	
Q04828	AK1C1_HUMAN	36788	AKR1C1	Aldo-keto reductase family 1 member C1 OS=Homo sapiens GN=AKR1C1 PE=1 SV=1	3			3	2		2
Q15274	NADC_HUMAN	30846	QPRT	Nicotinate-nucleotide pyrophosphorylase [carboxylating] OS=Homo sapiens GN=QPRT PE=1 SV=3	3			2	3		2
P08708	RS17_HUMAN	15550	RPS17	40S ribosomal protein S17 OS=Homo sapiens GN=RPS17 PE=1 SV=2	3				3	3	1
P62495	ERF1_HUMAN	49031	ETF1	Eukaryotic peptide chain release factor subunit 1 OS=Homo sapiens GN=ETF1 PE=1 SV=3	3				2	2	3
O60333-2	KIF1B_HUMAN	199263	KIF1B	Isoform 2 of Kinesin-like protein KIF1B OS=Homo sapiens GN=KIF1B	2	5			2		
O95831-3	AIFM1_HUMAN	66295	AIFM1	Isoform 3 of Apoptosis-inducing factor 1, mitochondrial OS=Homo sapiens GN=AIFM1	2			3	4		
Q08397	LOXL1_HUMAN	63110	LOXL1	Lysyl oxidase homolog 1 OS=Homo sapiens GN=LOXL1 PE=1 SV=2	2			2	5		
Q14980-2	NUMA1_HUMAN	236516	NUMA1	Isoform 2 of Nuclear mitotic apparatus protein 1 OS=Homo sapiens GN=NUMA1	2			3	4		
Q9ULA0	DNPEP_HUMAN	52428	DNPEP	Aspartyl aminopeptidase OS=Homo sapiens GN=DNPEP PE=1 SV=1	2			4	3		
O60888-2	CUTA_HUMAN	20925	CUTA	Isoform A of Protein CutA OS=Homo sapiens GN=CUTA	2			3		4	
O15394	NCAM2_HUMAN	93046	NCAM2	Neural cell adhesion molecule 2 OS=Homo sapiens GN=NCAM2 PE=1 SV=2	2					5	2
P01210	PENK_HUMAN	30787	PENK	Proenkephalin-A OS=Homo sapiens GN=PENK PE=1 SV=1	2					5	2
Q01459	DIAC_HUMAN	43760	CTBS	Di-N-acetylchitobiase OS=Homo sapiens GN=CTBS PE=1 SV=1	2					3	4
Q6NW40	RGMB_HUMAN	47547	RGMB	RGM domain family member B OS=Homo sapiens GN=RGMB PE=1 SV=3	2					5	2
Q9H0B8-2	CRLD2_HUMAN	51044	CRISPLD2	Isoform 2 of Cysteine-rich secretory protein LCCL domain-containing 2 OS=Homo sapiens GN=CRISPLD2	2					4	3
P02656	APOC3_HUMAN	10852	APOC3	Apolipoprotein C-III OS=Homo sapiens GN=APOC3 PE=1 SV=1	5	1	1	1	1	2	
P48147	PPCE_HUMAN	80700	PREP	Prolyl endopeptidase OS=Homo sapiens GN=PREP PE=1 SV=2	5	2	1	1	1	1	
P51153	RAB13_HUMAN	22774	RAB13	Ras-related protein Rab-13 OS=Homo sapiens GN=RAB13 PE=1 SV=1	5	1	2	1	1	1	
O95292-2	VAPB_HUMAN	11152	VAPB	Isoform 2 of Vesicle-associated membrane protein-associated protein B/C OS=Homo sapiens GN=VAPB	5	1	2	1	1		1
Q13145	BAMBI_HUMAN	29108	BAMBI	BMP and activin membrane-bound inhibitor homolog OS=Homo sapiens GN=BAMBI PE=1 SV=1	5	1	2		1	1	1
Q8N7X1	RMXL3_HUMAN	114938	RBMXL3	RNA-binding motif protein, X-linked-like-3 OS=Homo sapiens GN=RBMXL3 PE=2 SV=2	5	1	2		1	1	1
O00203	AP3B1_HUMAN	121320	AP3B1	AP-3 complex subunit beta-1 OS=Homo sapiens GN=AP3B1 PE=1 SV=3	5	1		1	1	2	1
Q5JQF8	PAP1M_HUMAN	22799	PABPC1L2A	Polyadenylate-binding protein 1-like 2 OS=Homo sapiens GN=PABPC1L2A PE=2 SV=1	5	2		1	1	1	1
Q8NBK3-2	SUMF1_HUMAN	31900	SUMF1	Isoform 2 of Sulfatase-modifying factor 1 OS=Homo sapiens GN=SUMF1	5	1		1	1	1	2
Q6PIK3	Q6PIK3_HUMAN	13536	RAB4B	HCG1995540, isoform CRA_b OS=Homo sapiens GN=RAB4B PE=2 SV=1	5	1		2	1	1	1
O43665-2	RGS10_HUMAN	19608	RGS10	Isoform 2 of Regulator of G-protein signaling 10 OS=Homo sapiens GN=RGS10	4	1	3	1	1		
Q5JTJ3-2	COA6_HUMAN	18094	COA6	Isoform 2 of Cytochrome c oxidase assembly factor 6 homolog OS=Homo sapiens GN=COA6	4	1	1	2	2		
Q9UJ68-2	MSRA_HUMAN	18963	MSRA	Isoform 2 of Mitochondrial peptide methionine sulfoxide reductase OS=Homo sapiens GN=MSRA	4	1	1	2	2		
B7Z2C5	B7Z2C5_HUMAN	53058	CDKL3	Cyclin-dependent kinase-like 3 OS=Homo sapiens GN=CDKL3 PE=2 SV=1	4	1	1	2	2		
P29323-2	EPHB2_HUMAN	109874	EPHB2	Isoform 2 of Ephrin type-B receptor 2 OS=Homo sapiens GN=EPHB2	4	1	2		2	1	
P22004	BMP6_HUMAN	57226	BMP6	Bone morphogenetic protein 6 OS=Homo sapiens GN=BMP6 PE=1 SV=1	4	2	1			1	2
P52758	UK114_HUMAN	14494	HRSP12	Ribonuclease UK114 OS=Homo sapiens GN=HRSP12 PE=1 SV=1	4	2	1			1	2

Q13361	MFAP5_HUMAN	19612	MFAP5	Microfibrillar-associated protein 5 OS=Homo sapiens GN=MFAP5 PE=1 SV=1	4	1	1			3	1
Q7Z490	Q7Z490_HUMAN	18608	MAGP2	Microfibril-associated glycoprotein 2 OS=Homo sapiens GN=MAGP2 PE=2 SV=1	4	1	1			3	1
Q9Y5A9-2	YTHD2_HUMAN	56877	YTHDF2	Isoform 2 of YTH domain-containing family protein 2 OS=Homo sapiens GN=YTHDF2	4	1		1	3	1	
P27482	CALL3_HUMAN	16891	CALML3	Calmodulin-like protein 3 OS=Homo sapiens GN=CALML3 PE=1 SV=2	4	2		1	1		2
O60925	PFD1_HUMAN	14210	PFDN1	Prefoldin subunit 1 OS=Homo sapiens GN=PFDN1 PE=1 SV=2	4	3		1		1	1
O15514	RPB4_HUMAN	16311	POLR2D	DNA-directed RNA polymerase II subunit RPB4 OS=Homo sapiens GN=POLR2D PE=1 SV=1	4		1	1	1	3	
P46439	GSTM5_HUMAN	25675	GSTM5	Glutathione S-transferase Mu 5 OS=Homo sapiens GN=GSTM5 PE=1 SV=3	4		2	1	1	2	
Q8NDH3-2	PEPL1_HUMAN	44126	NPEPL1	Isoform 2 of Probable aminopeptidase NPEPL1 OS=Homo sapiens GN=NPEPL1	4		1	1	2		2
Q9C075	K1C23_HUMAN	48131	KRT23	Keratin, type I cytoskeletal 23 OS=Homo sapiens GN=KRT23 PE=1 SV=2	4		1	1	2		2
Q96GD0	PLPP_HUMAN	31698	PDXP	Pyridoxal phosphate phosphatase OS=Homo sapiens GN=PDXP PE=1 SV=2	4		1		1	2	2
P12236	ADT3_HUMAN	32866	SLC25A6	ADP/ATP translocase 3 OS=Homo sapiens GN=SLC25A6 PE=1 SV=4	4			2	2	1	1
P27824	CALX_HUMAN	67568	CANX	Calnexin OS=Homo sapiens GN=CANX PE=1 SV=2	4			2	1	1	2
Q15836	VAMP3_HUMAN	11309	VAMP3	Vesicle-associated membrane protein 3 OS=Homo sapiens GN=VAMP3 PE=1 SV=3	4			1	1	2	2
Q92187	SIA8D_HUMAN	41295	ST8SIA4	CMP-N-acetylneuraminate-poly-alpha-2,8-sialyltransferase OS=Homo sapiens GN=ST8SIA4 PE=1 SV=1	4			1	2	2	1
Q96FJ2	DYL2_HUMAN	10350	DYNLL2	Dynein light chain 2, cytoplasmic OS=Homo sapiens GN=DYNLL2 PE=1 SV=1	4			1	1	3	1
Q9GZN8-2	CT027_HUMAN	21645	C20orf27	Isoform 2 of UPF0687 protein C20orf27 OS=Homo sapiens GN=C20orf27	4			1	3	1	1
Q9H0W9-2	CK054_HUMAN	33098	C11orf54	Isoform 2 of Ester hydrolase C11orf54 OS=Homo sapiens GN=C11orf54	4			2	1	2	1
P0CG29	GST2_HUMAN	27506	GSTT2	Glutathione S-transferase theta-2 OS=Homo sapiens GN=GSTT2 PE=1 SV=1	3	2	3	1			
P0CG30	GSTT2_HUMAN	27507	GSTT2B	Glutathione S-transferase theta-2B OS=Homo sapiens GN=GSTT2B PE=1 SV=1	3	2	3	1			
Q6ICJ4	Q6ICJ4_HUMAN	25948	Em:AP000351.3	Em:AP000351.3 protein OS=Homo sapiens GN=Em:AP000351.3 PE=2 SV=1	3	2	3	1			
O95816	BAG2_HUMAN	23772	BAG2	BAG family molecular chaperone regulator 2 OS=Homo sapiens GN=BAG2 PE=1 SV=1	3	3	2		1		
Q12931	TRAP1_HUMAN	80110	TRAP1	Heat shock protein 75 kDa, mitochondrial OS=Homo sapiens GN=TRAP1 PE=1 SV=3	3	2	2		2		
Q6FI81-3	CPIN1_HUMAN	32213	CIAPIN1	Isoform 3 of Anamorsin OS=Homo sapiens GN=CIAPIN1	3	1	2		3		
P55283	CADH4_HUMAN	100281	CDH4	Cadherin-4 OS=Homo sapiens GN=CDH4 PE=2 SV=2	3	3	1			2	
O43897-2	TLL1_HUMAN	44186	TLL1	Isoform 2 of Tolloid-like protein 1 OS=Homo sapiens GN=TLL1	3	2	2				2
Q9UHD1	CHRD1_HUMAN	37490	CHORDC1	Cysteine and histidine-rich domain-containing protein 1 OS=Homo sapiens GN=CHORDC1 PE=1 SV=2	3	1		2	3		
P05089-2	ARGI1_HUMAN	35664	ARG1	Isoform 2 of Arginase-1 OS=Homo sapiens GN=ARG1	3		1	4	1		
P16083	NQO2_HUMAN	25919	NQO2	Ribosyldihydronicotinamide dehydrogenase [quinone] OS=Homo sapiens GN=NQO2 PE=1 SV=5	3		1	2	3		
P49459	UBE2A_HUMAN	17315	UBE2A	Ubiquitin-conjugating enzyme E2 A OS=Homo sapiens GN=UBE2A PE=1 SV=2	3		2	2	2		
P50990	TCPQ_HUMAN	59621	CCT8	T-complex protein 1 subunit theta OS=Homo sapiens GN=CCT8 PE=1 SV=4	3		3	1		2	
P68431	H31_HUMAN	15404	HIST1H3A	Histone H3.1 OS=Homo sapiens GN=HIST1H3A PE=1 SV=2	3		1		2		3
P84243	H33_HUMAN	15328	H3F3A	Histone H3.3 OS=Homo sapiens GN=H3F3A PE=1 SV=2	3		1		2		3
Q16695	H31T_HUMAN	15508	HIST3H3	Histone H3.1t OS=Homo sapiens GN=HIST3H3 PE=1 SV=3	3		1		2		3
Q6NXT2	H3C_HUMAN	15214	H3F3C	Histone H3.3C OS=Homo sapiens GN=H3F3C PE=1 SV=3	3		1		2		3
Q71DI3	H32_HUMAN	15388	HIST2H3A	Histone H3.2 OS=Homo sapiens GN=HIST2H3A PE=1 SV=3	3		1		2		3
K7EK07	K7EK07_HUMAN	14914	H3F3B	Histone H3 (Fragment) OS=Homo sapiens GN=H3F3B PE=2 SV=1	3		1		2		3
Q5TEC6	Q5TEC6_HUMAN	15430	HIST2H3PS2	Histone H3 OS=Homo sapiens GN=HIST2H3PS2 PE=1 SV=1	3		1		2		3
P01834	IGKC_HUMAN	11609	IGKC	Ig kappa chain C region OS=Homo sapiens GN=IGKC PE=1 SV=1	3			2	3	1	
P22413	ENPP1_HUMAN	104924	ENPP1	Ectonucleotide pyrophosphatase/phosphodiesterase family member 1 OS=Homo sapiens GN=ENPP1 PE=1 SV=2	3			2	2	2	
Q53H82	LACB2_HUMAN	32806	LACTB2	Beta-lactamase-like protein 2 OS=Homo sapiens GN=LACTB2 PE=1 SV=2	3			2	2	2	
Q99417	MYCBP_HUMAN	11967	MYCBP	C-Myc-binding protein OS=Homo sapiens GN=MYCBP PE=1 SV=3	3			2	2	2	
Q9GZP4-2	PITH1_HUMAN	24121	PITHD1	Isoform 2 of PITH domain-containing protein 1 OS=Homo sapiens GN=PITHD1	3			2	3	1	
Q9UBQ0-2	VPS29_HUMAN	20927	VPS29	Isoform 2 of Vacuolar protein sorting-associated protein 29 OS=Homo sapiens GN=VPS29	3			2	2	2	

O94973-2	AP2A2_HUMAN	104090	AP2A2	Isoform 2 of AP-2 complex subunit alpha-2 OS=Homo sapiens GN=AP2A2	3			3	2		1
P42330	AK1C3_HUMAN	36853	AKR1C3	Aldo-keto reductase family 1 member C3 OS=Homo sapiens GN=AKR1C3 PE=1 SV=4	3			2	2		2
P62318	SMD3_HUMAN	13916	SNRPD3	Small nuclear ribonucleoprotein Sm D3 OS=Homo sapiens GN=SNRPD3 PE=1 SV=1	3			2	2		2
Q96C86	DCPS_HUMAN	38609	DCPS	m7GpppX diphosphatase OS=Homo sapiens GN=DCPS PE=1 SV=2	3			4	1		1
P21781	FGF7_HUMAN	22509	FGF7	Fibroblast growth factor 7 OS=Homo sapiens GN=FGF7 PE=1 SV=1	3			1		2	3
P49590	SYHM_HUMAN	56888	HARS2	Probable histidine--tRNA ligase, mitochondrial OS=Homo sapiens GN=HARS2 PE=1 SV=1	3			2		2	2
Q6UVY6	MOXD1_HUMAN	69652	MOXD1	DBH-like monooxygenase protein 1 OS=Homo sapiens GN=MOXD1 PE=2 SV=1	3			1		4	1
P48061-2	SDF1_HUMAN	10103	CXCL12	Isoform Alpha of Stromal cell-derived factor 1 OS=Homo sapiens GN=CXCL12	2	3	3				
Q15758	AAAT_HUMAN	56598	SLC1A5	Neutral amino acid transporter B(0) OS=Homo sapiens GN=SLC1A5 PE=1 SV=2	2	3	3				
Q8N8Z6-2	DCBD1_HUMAN	59196	DCBLD1	Isoform 2 of Discoidin, CUB and LCCL domain-containing protein 1 OS=Homo sapiens GN=DCBLD1	2		2			4	
O14556	G3PT_HUMAN	44501	GAPDHS	Glyceraldehyde-3-phosphate dehydrogenase, testis-specific OS=Homo sapiens GN=GAPDHS PE=1 SV=2	2			3	3		
P01031	CO5_HUMAN	188305	C5	Complement C5 OS=Homo sapiens GN=C5 PE=1 SV=4	2			4	2		
P15880	RS2_HUMAN	31324	RPS2	40S ribosomal protein S2 OS=Homo sapiens GN=RPS2 PE=1 SV=2	2			2	4		
P34897-2	GLYM_HUMAN	54863	SHMT2	Isoform 2 of Serine hydroxymethyltransferase, mitochondrial OS=Homo sapiens GN=SHMT2	2			3	3		
Q99470	SDF2_HUMAN	23026	SDF2	Stromal cell-derived factor 2 OS=Homo sapiens GN=SDF2 PE=1 SV=2	2			3	3		
Q9NUJ1	ABHDA_HUMAN	33933	ABHD10	Mycophenolic acid acyl-glucuronide esterase, mitochondrial OS=Homo sapiens GN=ABHD10 PE=1 SV=1	2			2	4		
P00742	FA10_HUMAN	54732	F10	Coagulation factor X OS=Homo sapiens GN=F10 PE=1 SV=2	2					3	3
Q8IVM0-2	CCD50_HUMAN	56340	CCDC50	Isoform 2 of Coiled-coil domain-containing protein 50 OS=Homo sapiens GN=CCDC50	2					2	4
Q9NPF2-2	CHSTB_HUMAN	41003	CHST11	Isoform 2 of Carbohydrate sulfotransferase 11 OS=Homo sapiens GN=CHST11	2					3	3
P00738	HPT_HUMAN	45205	HP	Haptoglobin OS=Homo sapiens GN=HP PE=1 SV=1	1			6			
P28062-2	PSB8_HUMAN	29770	PSMB8	Isoform 2 of Proteasome subunit beta type-8 OS=Homo sapiens GN=PSMB8	1				6		
H3BSW8	H3BSW8_HUMAN	9652	MTHFSD	Methenyltetrahydrofolate synthase domain-containing protein OS=Homo sapiens GN=MTHFSD PE=2 SV=1	1						6
Q9UK76-2	HN1_HUMAN	19916	HN1	Isoform 2 of Hematological and neurological expressed 1 protein OS=Homo sapiens GN=HN1	5	1	1	1	1		1
Q7RTV0	PHF5A_HUMAN	12405	PHF5A	PHD finger-like domain-containing protein 5A OS=Homo sapiens GN=PHF5A PE=1 SV=1	5	1	1	1		1	1
P46060	RAGP1_HUMAN	63542	RANGAP1	Ran GTPase-activating protein 1 OS=Homo sapiens GN=RANGAP1 PE=1 SV=1	5	1	1		1	1	1
Q9Y376	CAB39_HUMAN	39869	CAB39	Calcium-binding protein 39 OS=Homo sapiens GN=CAB39 PE=1 SV=1	5	1	1		1	1	1
Q9Y2Z0-2	SUGT1_HUMAN	37805	SUGT1	Isoform 2 of Suppressor of G2 allele of SKP1 homolog OS=Homo sapiens GN=SUGT1	4	2	1	1	1		
Q15029-2	U5S1_HUMAN	105384	EFTUD2	Isoform 2 of 116 kDa U5 small nuclear ribonucleoprotein component OS=Homo sapiens GN=EFTUD2	4	2	1	1			1
P57735	RAB25_HUMAN	23496	RAB25	Ras-related protein Rab-25 OS=Homo sapiens GN=RAB25 PE=1 SV=2	4	1	2		1		1
Q13200	PSMD2_HUMAN	100200	PSMD2	26S proteasome non-ATPase regulatory subunit 2 OS=Homo sapiens GN=PSMD2 PE=1 SV=3	4	1	1		2		1
Q9BWJ5	SF3B5_HUMAN	10135	SF3B5	Splicing factor 3B subunit 5 OS=Homo sapiens GN=SF3B5 PE=1 SV=1	4	2	1		1		1
O14964-2	HGS_HUMAN	76376	HGS	Isoform 2 of Hepatocyte growth factor-regulated tyrosine kinase substrate OS=Homo sapiens GN=HGS	4	1	1			1	2
P08294	SODE_HUMAN	25851	SOD3	Extracellular superoxide dismutase [Cu-Zn] OS=Homo sapiens GN=SOD3 PE=1 SV=2	4	1	1			1	2
P30626-2	SORCN_HUMAN	20345	SRI	Isoform 2 of Sorcin OS=Homo sapiens GN=SRI	4	2	1			1	1
Q9Y5H2-2	PCDGB_HUMAN	91608	PCDHGA11	Isoform 2 of Protocadherin gamma-A11 OS=Homo sapiens GN=PCDHGA11	4	1	1			1	2
O43324-2	MCA3_HUMAN	15548	EEF1E1	Isoform 2 of Eukaryotic translation elongation factor 1 epsilon-1 OS=Homo sapiens GN=EEF1E1	4	1		1	1	2	
P34059	GALNS_HUMAN	58026	GALNS	N-acetylgalactosamine-6-sulfatase OS=Homo sapiens GN=GALNS PE=1 SV=1	4	1		1	1	2	
C9J1V9	C9J1V9_HUMAN	17018	EEF1E1-BLOC1S5	HCG2043275 OS=Homo sapiens GN=EEF1E1-BLOC1S5 PE=4 SV=2	4	1		1	1	2	

Q9BRT3	MIEN1_HUMAN	12403	MIEN1	Migration and invasion enhancer 1 OS=Homo sapiens GN=MIEN1 PE=1 SV=1	4	1		1		2	1
P37108	SRP14_HUMAN	14570	SRP14	Signal recognition particle 14 kDa protein OS=Homo sapiens GN=SRP14 PE=1 SV=2	4		1	1		2	1
Q13449	LSAMP_HUMAN	37393	LSAMP	Limbic system-associated membrane protein OS=Homo sapiens GN=LSAMP PE=1 SV=2	4		1	1		2	1
O60245-2	PCDH7_HUMAN	116429	PCDH7	Isoform B of Protocadherin-7 OS=Homo sapiens GN=PCDH7	4		1		1	1	2
P04733	MT1F_HUMAN	6086	MT1F	Metallothionein-1F OS=Homo sapiens GN=MT1F PE=1 SV=1	4			2	1	1	1
P07305-2	H10_HUMAN	19167	H1F0	Isoform 2 of Histone H1.0 OS=Homo sapiens GN=H1F0	4			1	1	2	1
P07438	MT1B_HUMAN	6115	MT1B	Metallothionein-1B OS=Homo sapiens GN=MT1B PE=2 SV=1	4			1	1	2	1
P29144	TPP2_HUMAN	138350	TPP2	Tripeptidyl-peptidase 2 OS=Homo sapiens GN=TPP2 PE=1 SV=4	4			2	1	1	1
P62851	RS25_HUMAN	13742	RPS25	40S ribosomal protein S25 OS=Homo sapiens GN=RPS25 PE=1 SV=1	4			1	2	1	1
O95302	FKBP9_HUMAN	63084	FKBP9	Peptidyl-prolyl cis-trans isomerase FKBP9 OS=Homo sapiens GN=FKBP9 PE=1 SV=2	3	1	3			1	
P51911	CNN1_HUMAN	33170	CNN1	Calponin-1 OS=Homo sapiens GN=CNN1 PE=1 SV=2	3	1	2			2	
Q3LXA3	DHAK_HUMAN	58947	DAK	Bifunctional ATP-dependent dihydroxyacetone kinase/FAD-AMP lyase (cyclizing) OS=Homo sapiens GN=DAK PE=1 SV=2	3	2	2			1	
Q96AE4-2	FUBP1_HUMAN	68605	FUBP1	Isoform 2 of Far upstream element-binding protein 1 OS=Homo sapiens GN=FUBP1	3	2	1			2	
Q99622	C10_HUMAN	13178	C12orf57	Protein C10 OS=Homo sapiens GN=C12orf57 PE=1 SV=1	3	1	2			2	
P20849	CO9A1_HUMAN	91869	COL9A1	Collagen alpha-1(IX) chain OS=Homo sapiens GN=COL9A1 PE=1 SV=3	3	2		1	2		
P35637-2	FUS_HUMAN	53355	FUS	Isoform Short of RNA-binding protein FUS OS=Homo sapiens GN=FUS	3	1		2	2		
P49589-2	SYCC_HUMAN	82846	CARS	Isoform 2 of Cysteine--tRNA ligase, cytoplasmic OS=Homo sapiens GN=CARS	3	1		1	3		
Q9Y3C8	UFC1_HUMAN	19458	UFC1	Ubiquitin-fold modifier-conjugating enzyme 1 OS=Homo sapiens GN=UFC1 PE=1 SV=3	3	2		2		1	
O15232	MATN3_HUMAN	52817	MATN3	Matrilin-3 OS=Homo sapiens GN=MATN3 PE=1 SV=2	3	2		1			2
Q9H488	OFUT1_HUMAN	43956	POFUT1	GDP-fucose protein O-fucosyltransferase 1 OS=Homo sapiens GN=POFUT1 PE=1 SV=1	3	1			2	2	
P06280	AGAL_HUMAN	48767	GLA	Alpha-galactosidase A OS=Homo sapiens GN=GLA PE=1 SV=1	3	1				2	2
P43234	CATO_HUMAN	35958	CTSO	Cathepsin O OS=Homo sapiens GN=CTSO PE=2 SV=1	3	1				2	2
P51452	DUS3_HUMAN	20478	DUSP3	Dual specificity protein phosphatase 3 OS=Homo sapiens GN=DUSP3 PE=1 SV=1	3	2				2	1
Q96J84-2	KIRR1_HUMAN	85049	KIRREL	Isoform 2 of Kin of IRRE-like protein 1 OS=Homo sapiens GN=KIRREL	3	1				2	2
P14621	ACYP2_HUMAN	11140	ACYP2	Acylphosphatase-2 OS=Homo sapiens GN=ACYP2 PE=1 SV=2	3		2	1	2		
Q8IZA3	H1FOO_HUMAN	35813	H1FOO	Histone H1oo OS=Homo sapiens GN=H1FOO PE=2 SV=1	3		2	1	2		
Q96AG4	LRC59_HUMAN	34930	LRRC59	Leucine-rich repeat-containing protein 59 OS=Homo sapiens GN=LRRC59 PE=1 SV=1	3		1	1	3		
O95757	HS74L_HUMAN	94512	HSPA4L	Heat shock 70 kDa protein 4L OS=Homo sapiens GN=HSPA4L PE=1 SV=3	3		1	2		2	
Q86SJ2	AMGO2_HUMAN	57934	AMIGO2	Amphoterin-induced protein 2 OS=Homo sapiens GN=AMIGO2 PE=1 SV=1	3		1			3	1
Q9BZL1	UBL5_HUMAN	8547	UBL5	Ubiquitin-like protein 5 OS=Homo sapiens GN=UBL5 PE=1 SV=1	3		1			2	2
O00244	ATOX1_HUMAN	7402	ATOX1	Copper transport protein ATOX1 OS=Homo sapiens GN=ATOX1 PE=1 SV=1	3			1	1	3	
P06756-2	ITAV_HUMAN	112258	ITGAV	Isoform 2 of Integrin alpha-V OS=Homo sapiens GN=ITGAV	3			1	2	2	
P07814	SYEP_HUMAN	170591	EPRS	Bifunctional glutamate/proline--tRNA ligase OS=Homo sapiens GN=EPRS PE=1 SV=5	3			2	1	2	
P26368-2	U2AF2_HUMAN	53121	U2AF2	Isoform 2 of Splicing factor U2AF 65 kDa subunit OS=Homo sapiens GN=U2AF2	3			2	2	1	
P30042-2	ES1_HUMAN	24758	C21orf33	Isoform Short of ES1 protein homolog, mitochondrial OS=Homo sapiens GN=C21orf33	3			2	2	1	
P48556	PSMD8_HUMAN	39612	PSMD8	26S proteasome non-ATPase regulatory subunit 8 OS=Homo sapiens GN=PSMD8 PE=1 SV=2	3			2	2	1	
P50583	AP4A_HUMAN	16829	NUDT2	Bis(5'-nucleosyl)-tetraphosphatase [asymmetrical] OS=Homo sapiens GN=NUDT2 PE=1 SV=3	3			2	2	1	
P62081	RS7_HUMAN	22127	RPS7	40S ribosomal protein S7 OS=Homo sapiens GN=RPS7 PE=1 SV=1	3			2	2	1	
P62304	RUXE_HUMAN	10804	SNRPE	Small nuclear ribonucleoprotein E OS=Homo sapiens GN=SNRPE PE=1 SV=1	3			2	2	1	
P62847-2	RS24_HUMAN	15069	RPS24	Isoform 2 of 40S ribosomal protein S24 OS=Homo sapiens GN=RPS24	3			2	2	1	
Q5VW32	BROX_HUMAN	46476	BROX	BRO1 domain-containing protein BROX OS=Homo sapiens GN=BROX PE=1 SV=1	3			2	2	1	
Q9BYT8	NEUL_HUMAN	80652	NLN	Neurolysin, mitochondrial OS=Homo sapiens GN=NLN PE=1 SV=1	3			1	3	1	
Q9NQC3-2	RTN4_HUMAN	40318	RTN4	Isoform 2 of Reticulon-4 OS=Homo sapiens GN=RTN4	3			1	3	1	
Q9NVZ3-2	NECP2_HUMAN	29464	NECAP2	Isoform 2 of Adaptin ear-binding coat-associated protein 2 OS=Homo sapiens	3			2	2	1	

				GN=NECAP2								
Q9UHB6-2	LIMA1_HUMAN	67119	LIMA1	Isoform Alpha of LIM domain and actin-binding protein 1 OS=Homo sapiens GN=LIMA1	3			1	1	3		
Q9ULZ3-2	ASC_HUMAN	19969	PYCARD	Isoform 2 of Apoptosis-associated speck-like protein containing a CARD OS=Homo sapiens GN=PYCARD	3			2	2	1		
Q9H1B7	I2BPL_HUMAN	82659	IRF2BPL	Interferon regulatory factor 2-binding protein-like OS=Homo sapiens GN=IRF2BPL PE=1 SV=1	3			1	3		1	
Q9HCN8	SDF2L_HUMAN	23598	SDF2L1	Stromal cell-derived factor 2-like protein 1 OS=Homo sapiens GN=SDF2L1 PE=1 SV=2	3			1	2		2	
P25445-6	TNR6_HUMAN	35386	FAS	Isoform 6 of Tumor necrosis factor receptor superfamily member 6 OS=Homo sapiens GN=FAS	3			1		2	2	
P42166	LAP2A_HUMAN	75492	TMPO	Lamina-associated polypeptide 2, isoform alpha OS=Homo sapiens GN=TMPO PE=1 SV=2	3			2		2	1	
P39019	RS19_HUMAN	16060	RPS19	40S ribosomal protein S19 OS=Homo sapiens GN=RPS19 PE=1 SV=2	3				1	2	2	
Q13232	NDK3_HUMAN	19015	NME3	Nucleoside diphosphate kinase 3 OS=Homo sapiens GN=NME3 PE=1 SV=2	3				1	2	2	
Q9Y3Q3-2	TMED3_HUMAN	19635	TMED3	Isoform 2 of Transmembrane emp24 domain-containing protein 3 OS=Homo sapiens GN=TMED3	3				1	2	2	
H0YN88	H0YN88_HUMAN	15920	RPS17L	40S ribosomal protein S17 OS=Homo sapiens GN=RPS17L PE=2 SV=1	3				2	2	1	
Q8IWU6	SULF1_HUMAN	101027	SULF1	Extracellular sulfatase Sulf-1 OS=Homo sapiens GN=SULF1 PE=1 SV=1	2	3	2					
Q11201	SIA4A_HUMAN	39075	ST3GAL1	CMP-N-acetylneuraminate-beta-galactosamide-alpha-2,3-sialyltransferase 1 OS=Homo sapiens GN=ST3GAL1 PE=2 SV=1	2	3				2		
Q9H251-2	CAD23_HUMAN	369267	CDH23	Isoform 2 of Cadherin-23 OS=Homo sapiens GN=CDH23	2		4		1			
Q02487-2	DSC2_HUMAN	93769	DSC2	Isoform 2B of Desmocollin-2 OS=Homo sapiens GN=DSC2	2		4			1		
P02679-2	FIBG_HUMAN	49497	FGG	Isoform Gamma-A of Fibrinogen gamma chain OS=Homo sapiens GN=FGG	2			1	4			
P05062	ALDOB_HUMAN	39473	ALDOB	Fructose-bisphosphate aldolase B OS=Homo sapiens GN=ALDOB PE=1 SV=2	2			3	2			
P06730-2	IF4E_HUMAN	28778	EIF4E	Isoform 2 of Eukaryotic translation initiation factor 4E OS=Homo sapiens GN=EIF4E	2			3	2			
P09874	PARP1_HUMAN	113084	PARP1	Poly [ADP-ribose] polymerase 1 OS=Homo sapiens GN=PARP1 PE=1 SV=4	2			2	3			
P55809	SCOT1_HUMAN	56158	OXCT1	Succinyl-CoA:3-ketoacid coenzyme A transferase 1, mitochondrial OS=Homo sapiens GN=OXCT1 PE=1 SV=1	2			2	3			
Q13263-2	TIF1B_HUMAN	79474	TRIM28	Isoform 2 of Transcription intermediary factor 1-beta OS=Homo sapiens GN=TRIM28	2			3	2			
Q86UP2-2	KTN1_HUMAN	149611	KTN1	Isoform 2 of Kinectin OS=Homo sapiens GN=KTN1	2			2	3			
Q8IVD9	NUDC3_HUMAN	40822	NUDCD3	NudC domain-containing protein 3 OS=Homo sapiens GN=NUDCD3 PE=1 SV=3	2			3	2			
Q9H6Z4-2	RANB3_HUMAN	59694	RANBP3	Isoform 2 of Ran-binding protein 3 OS=Homo sapiens GN=RANBP3	2			2	3			
Q9UPN3-5	MACF1_HUMAN	670137	MACF1	Isoform 4 of Microtubule-actin cross-linking factor 1, isoforms 1/2/3/5 OS=Homo sapiens GN=MACF1	2			3	2			
P47224	MSS4_HUMAN	13839	RABIF	Guanine nucleotide exchange factor MSS4 OS=Homo sapiens GN=RABIF PE=1 SV=2	2			2		3		
Q6KB66-2	K2C80_HUMAN	47242	KRT80	Isoform 2 of Keratin, type II cytoskeletal 80 OS=Homo sapiens GN=KRT80	2			3		2		
Q8TF72	SHRM3_HUMAN	216857	SHROOM3	Protein Shroom3 OS=Homo sapiens GN=SHROOM3 PE=1 SV=2	2			2		3		
Q9BX68	HINT2_HUMAN	17162	HINT2	Histidine triad nucleotide-binding protein 2, mitochondrial OS=Homo sapiens GN=HINT2 PE=1 SV=1	2			3		2		
Q96QT4	TRPM7_HUMAN	212697	TRPM7	Transient receptor potential cation channel subfamily M member 7 OS=Homo sapiens GN=TRPM7 PE=1 SV=1	2				2	3		
P63151-2	2ABA_HUMAN	53000	PPP2R2A	Isoform 2 of Serine/threonine-protein phosphatase 2A 55 kDa regulatory subunit B alpha isoform OS=Homo sapiens GN=PPP2R2A	2				2		3	
Q9Y5Q8-2	TF3C5_HUMAN	58266	GTF3C5	Isoform 2 of General transcription factor 3C polypeptide 5 OS=Homo sapiens GN=GTF3C5	2				2		3	
Q02742	GCNT1_HUMAN	49799	GCNT1	Beta-1,3-galactosyl-O-glycosyl-glycoprotein beta-1,6-N-acetylglucosaminyltransferase OS=Homo sapiens GN=GCNT1 PE=1 SV=2	2					3	2	
Q4LDE5	SVEP1_HUMAN	390170	SVEP1	Sushi, von Willebrand factor type A, EGF and pentraxin domain-containing protein 1 OS=Homo sapiens GN=SVEP1 PE=1 SV=3	2					3	2	
Q6UXB8-2	PI16_HUMAN	29674	PI16	Isoform 2 of Peptidase inhibitor 16 OS=Homo sapiens GN=PI16	2					3	2	
Q70JA7	CHSS3_HUMAN	100284	CHSY3	Chondroitin sulfate synthase 3 OS=Homo sapiens GN=CHSY3 PE=2 SV=3	2					3	2	
Q86SQ4-2	GP126_HUMAN	133671	GPR126	Isoform 2 of G-protein coupled receptor 126 OS=Homo sapiens GN=GPR126	2					4	1	

Q9NRA1	PDGFC_HUMAN	39029	PDGFC	Platelet-derived growth factor C OS=Homo sapiens GN=PDGFC PE=1 SV=2	2					2	3
Q9NZJ7-2	MTCH1_HUMAN	39920	MTCH1	Isoform 2 of Mitochondrial carrier homolog 1 OS=Homo sapiens GN=MTCH1	2					2	3
Q8IUC8-2	GLT13_HUMAN	55984	GALNT13	Isoform 2 of Polypeptide N-acetylgalactosaminyltransferase 13 OS=Homo sapiens GN=GALNT13	4	1	1		1	1	
Q53GG5-2	PDLI3_HUMAN	34280	PDLIM3	Isoform 2 of PDZ and LIM domain protein 3 OS=Homo sapiens GN=PDLIM3	4	1		1	1	1	
Q9ULI3-2	HEG1_HUMAN	106446	HEG1	Isoform 2 of Protein HEG homolog 1 OS=Homo sapiens GN=HEG1	4	1		1	1	1	
Q04637-3	IF4G1_HUMAN	171514	EIF4G1	Isoform B of Eukaryotic translation initiation factor 4 gamma 1 OS=Homo sapiens GN=EIF4G1	4	1		1	1		1
P12259	FA5_HUMAN	251703	F5	Coagulation factor V OS=Homo sapiens GN=F5 PE=1 SV=4	4	1			1	1	1
P61966	AP1S1_HUMAN	18733	AP1S1	AP-1 complex subunit sigma-1A OS=Homo sapiens GN=AP1S1 PE=1 SV=1	4		1	1		1	1
P04278-2	SHBG_HUMAN	31829	SHBG	Isoform 2 of Sex hormone-binding globulin OS=Homo sapiens GN=SHBG	4			1	1	1	1
P04731	MT1A_HUMAN	6120	MT1A	Metallothionein-1A OS=Homo sapiens GN=MT1A PE=1 SV=2	4			1	1	1	1
P05141	ADT2_HUMAN	32852	SLC25A5	ADP/ATP translocase 2 OS=Homo sapiens GN=SLC25A5 PE=1 SV=7	4			1	1	1	1
P12235	ADT1_HUMAN	33064	SLC25A4	ADP/ATP translocase 1 OS=Homo sapiens GN=SLC25A4 PE=1 SV=4	4			1	1	1	1
P63027	VAMP2_HUMAN	12663	VAMP2	Vesicle-associated membrane protein 2 OS=Homo sapiens GN=VAMP2 PE=1 SV=3	4			1	1	1	1
P80294	MT1H_HUMAN	6039	MT1H	Metallothionein-1H OS=Homo sapiens GN=MT1H PE=1 SV=1	4			1	1	1	1
Q04721	NOTC2_HUMAN	265405	NOTCH2	Neurogenic locus notch homolog protein 2 OS=Homo sapiens GN=NOTCH2 PE=1 SV=3	4			1	1	1	1
Q93083	MT1L_HUMAN	6062	MT1L	Metallothionein-1L OS=Homo sapiens GN=MT1L PE=2 SV=1	4			1	1	1	1
Q9H0C2	ADT4_HUMAN	35022	SLC25A31	ADP/ATP translocase 4 OS=Homo sapiens GN=SLC25A31 PE=2 SV=1	4			1	1	1	1
O15230	LAMA5_HUMAN	399737	LAMA5	Laminin subunit alpha-5 OS=Homo sapiens GN=LAMA5 PE=1 SV=8	3	2	1			1	
O43242	PSMD3_HUMAN	60978	PSMD3	26S proteasome non-ATPase regulatory subunit 3 OS=Homo sapiens GN=PSMD3 PE=1 SV=2	3	2	1			1	
P08493-2	MGP_HUMAN	15325	MGP	Isoform 2 of Matrix Gla protein OS=Homo sapiens GN=MGP	3	2	1			1	
P42677	RS27_HUMAN	9461	RPS27	40S ribosomal protein S27 OS=Homo sapiens GN=RPS27 PE=1 SV=3	3	2	1			1	
P62310	LSM3_HUMAN	11845	LSM3	U6 snRNA-associated Sm-like protein LSm3 OS=Homo sapiens GN=LSM3 PE=1 SV=2	3	1	1			2	
Q71UM5	RS27L_HUMAN	9477	RPS27L	40S ribosomal protein S27-like OS=Homo sapiens GN=RPS27L PE=1 SV=3	3	2	1			1	
Q9Y5G4-2	PCDG9_HUMAN	91248	PCDHGA9	Isoform 2 of Protocadherin gamma-A9 OS=Homo sapiens GN=PCDHGA9	3	1	1				2
Q9Y5G7-2	PCDG6_HUMAN	89163	PCDHGA6	Isoform 2 of Protocadherin gamma-A6 OS=Homo sapiens GN=PCDHGA6	3	1	1				2
Q9Y5G8-2	PCDG5_HUMAN	88759	PCDHGA5	Isoform 2 of Protocadherin gamma-A5 OS=Homo sapiens GN=PCDHGA5	3	1	1				2
Q9Y5H0-2	PCDG3_HUMAN	90473	PCDHGA3	Isoform 2 of Protocadherin gamma-A3 OS=Homo sapiens GN=PCDHGA3	3	1	1				2
Q9Y5H3-2	PCDGA_HUMAN	92943	PCDHGA10	Isoform 2 of Protocadherin gamma-A10 OS=Homo sapiens GN=PCDHGA10	3	1	1				2
Q08209-2	PP2BA_HUMAN	57659	PPP3CA	Isoform 2 of Serine/threonine-protein phosphatase 2B catalytic subunit alpha isoform OS=Homo sapiens GN=PPP3CA	3	1		2	1		
Q6UXH1-4	CREL2_HUMAN	35288	CRELD2	Isoform 4 of Cysteine-rich with EGF-like domain protein 2 OS=Homo sapiens GN=CRELD2	3	1		2	1		
Q9HB07	MYG1_HUMAN	42449	C12orf10	UPF0160 protein MYG1, mitochondrial OS=Homo sapiens GN=C12orf10 PE=1 SV=2	3	1		1			2
Q9UBB4-2	ATX10_HUMAN	46286	ATXN10	Isoform 2 of Ataxin-10 OS=Homo sapiens GN=ATXN10	3	1		1			2
P53582	MAP11_HUMAN	43215	METAP1	Methionine aminopeptidase 1 OS=Homo sapiens GN=METAP1 PE=1 SV=2	3	1			1		2
Q9BY67-2	CADM1_HUMAN	36915	CADM1	Isoform 2 of Cell adhesion molecule 1 OS=Homo sapiens GN=CADM1	3		1	2	1		
P01106-2	MYC_HUMAN	50565	MYC	Isoform 2 of Myc proto-oncogene protein OS=Homo sapiens GN=MYC	3		2	1		1	
Q9Y536	PAL4A_HUMAN	18182	PPIAL4A	Peptidyl-prolyl cis-trans isomerase A-like 4A/B/C OS=Homo sapiens GN=PPIAL4A PE=2 SV=1	3		1	1			2
Q8WUJ3	K1199_HUMAN	152998	KIAA1199	Protein KIAA1199 OS=Homo sapiens GN=KIAA1199 PE=2 SV=2	3		1			2	1
Q9Y6N7-2	ROBO1_HUMAN	181328	ROBO1	Isoform 2 of Roundabout homolog 1 OS=Homo sapiens GN=ROBO1	3		2			1	1
O75340-2	PDCD6_HUMAN	21664	PDCD6	Isoform 2 of Programmed cell death protein 6 OS=Homo sapiens GN=PDCD6	3			1	2	1	
O95989	NUDT3_HUMAN	19471	NUDT3	Diphosphoinositol polyphosphate phosphohydrolase 1 OS=Homo sapiens GN=NUDT3 PE=1 SV=1	3			2	1	1	
P02748	CO9_HUMAN	63173	C9	Complement component C9 OS=Homo sapiens GN=C9 PE=1 SV=2	3			1	1	2	
P05019-2	IGF1_HUMAN	17026	IGF1	Isoform 2 of Insulin-like growth factor I OS=Homo sapiens GN=IGF1	3			1	2	1	
P25311	ZA2G_HUMAN	34259	AZGP1	Zinc-alpha-2-glycoprotein OS=Homo sapiens GN=AZGP1 PE=1 SV=2	3			2	1	1	

P35244	RFA3_HUMAN	13569	RPA3	Replication protein A 14 kDa subunit OS=Homo sapiens GN=RPA3 PE=1 SV=1	3			1	1	2	
P78559-2	MAP1A_HUMAN	305671	MAP1A	Isoform 2 of Microtubule-associated protein 1A OS=Homo sapiens GN=MAP1A	3			1	1	2	
Q96P70	IPO9_HUMAN	115963	IPO9	Importin-9 OS=Homo sapiens GN=IPO9 PE=1 SV=3	3			1	1	2	
Q99460-2	PSMD1_HUMAN	102258	PSMD1	Isoform 2 of 26S proteasome non-ATPase regulatory subunit 1 OS=Homo sapiens GN=PSMD1	3			2	1	1	
Q13429	Q13429_HUMAN	15611	IGF-I	Insulin-like growth factor I (Fragment) OS=Homo sapiens GN=IGF-I PE=2 SV=1	3			1	2	1	
O96033	MOC2A_HUMAN	9755	MOCS2	Molybdopterin synthase sulfur carrier subunit OS=Homo sapiens GN=MOCS2 PE=1 SV=1	3			1	2		1
P36957	ODO2_HUMAN	48755	DLST	Dihydrolipoyllysine-residue succinyltransferase component of 2-oxoglutarate dehydrogenase complex, mitochondrial OS=Homo sapiens GN=DLST PE=1 SV=4	3			1	2		1
P46783	RS10_HUMAN	18898	RPS10	40S ribosomal protein S10 OS=Homo sapiens GN=RPS10 PE=1 SV=1	3			1	1		2
P52895	AK1C2_HUMAN	36735	AKR1C2	Aldo-keto reductase family 1 member C2 OS=Homo sapiens GN=AKR1C2 PE=1 SV=3	3			2	1		1
Q01970-2	PLCB3_HUMAN	131205	PLCB3	Isoform 2 of 1-phosphatidylinositol 4,5-bisphosphate phosphodiesterase beta-3 OS=Homo sapiens GN=PLCB3	3			1	1		2
Q9NWY4	CD027_HUMAN	39436	C4orf27	UPF0609 protein C4orf27 OS=Homo sapiens GN=C4orf27 PE=1 SV=2	3			2	1		1
Q9UBU2	DKK2_HUMAN	28447	DKK2	Dickkopf-related protein 2 OS=Homo sapiens GN=DKK2 PE=1 SV=1	3			2		1	1
P62854	RS26_HUMAN	13015	RPS26	40S ribosomal protein S26 OS=Homo sapiens GN=RPS26 PE=1 SV=3	3				1	2	1
P50479	PDLI4_HUMAN	35398	PDLIM4	PDZ and LIM domain protein 4 OS=Homo sapiens GN=PDLIM4 PE=1 SV=2	2	1	3				
Q93045-2	STMN2_HUMAN	21802	STMN2	Isoform 2 of Stathmin-2 OS=Homo sapiens GN=STMN2	2	2	2				
Q99988	GDF15_HUMAN	34140	GDF15	Growth/differentiation factor 15 OS=Homo sapiens GN=GDF15 PE=1 SV=3	2	2	2				
O94903	PROSC_HUMAN	30344	PROSC	Proline synthase co-transcribed bacterial homolog protein OS=Homo sapiens GN=PROSC PE=1 SV=1	2	2				2	
P10606	COX5B_HUMAN	13696	COX5B	Cytochrome c oxidase subunit 5B, mitochondrial OS=Homo sapiens GN=COX5B PE=1 SV=2	2	2				2	
Q07960	RHG01_HUMAN	50436	ARHGAP1	Rho GTPase-activating protein 1 OS=Homo sapiens GN=ARHGAP1 PE=1 SV=1	2	2				2	
Q15056-2	IF4H_HUMAN	25200	EIF4H	Isoform Short of Eukaryotic translation initiation factor 4H OS=Homo sapiens GN=EIF4H	2	1				3	
P26447	S10A4_HUMAN	11729	S100A4	Protein S100-A4 OS=Homo sapiens GN=S100A4 PE=1 SV=1	2	1					3
P60903	S10AA_HUMAN	11203	S100A10	Protein S100-A10 OS=Homo sapiens GN=S100A10 PE=1 SV=2	2	2					2
Q9NR28-2	DBLOH_HUMAN	21233	DIABLO	Isoform 2 of Diablo homolog, mitochondrial OS=Homo sapiens GN=DIABLO	2		2				2
O00300	TR11B_HUMAN	46026	TNFRSF11B	Tumor necrosis factor receptor superfamily member 11B OS=Homo sapiens GN=TNFRSF11B PE=1 SV=3	2			2	2		
O00754-2	MA2B1_HUMAN	113616	MAN2B1	Isoform 2 of Lysosomal alpha-mannosidase OS=Homo sapiens GN=MAN2B1	2			2	2		
O43660-2	PLRG1_HUMAN	56290	PLRG1	Isoform 2 of Pleiotropic regulator 1 OS=Homo sapiens GN=PLRG1	2			2	2		
P02760	AMBP_HUMAN	38999	AMBP	Protein AMBP OS=Homo sapiens GN=AMBP PE=1 SV=1	2			2	2		
P04745	AMY1_HUMAN	57768	AMY1A	Alpha-amylase 1 OS=Homo sapiens GN=AMY1A PE=1 SV=2	2			1	3		
P06727	APOA4_HUMAN	45399	APOA4	Apolipoprotein A-IV OS=Homo sapiens GN=APOA4 PE=1 SV=3	2			1	3		
P15104	GLNA_HUMAN	42064	GLUL	Glutamine synthetase OS=Homo sapiens GN=GLUL PE=1 SV=4	2			2	2		
P19961	AMY2B_HUMAN	57710	AMY2B	Alpha-amylase 2B OS=Homo sapiens GN=AMY2B PE=1 SV=1	2			1	3		
P21281	VATB2_HUMAN	56501	ATP6V1B2	V-type proton ATPase subunit B, brain isoform OS=Homo sapiens GN=ATP6V1B2 PE=1 SV=3	2			3	1		
P31942-2	HNRH3_HUMAN	35239	HNRNPH3	Isoform 2 of Heterogeneous nuclear ribonucleoprotein H3 OS=Homo sapiens GN=HNRNPH3	2			1	3		
P32969	RL9_HUMAN	21863	RPL9	60S ribosomal protein L9 OS=Homo sapiens GN=RPL9 PE=1 SV=1	2			2	2		
P42765	THIM_HUMAN	41924	ACAA2	3-ketoacyl-CoA thiolase, mitochondrial OS=Homo sapiens GN=ACAA2 PE=1 SV=2	2			2	2		
P55060-3	XPO2_HUMAN	107778	CSE1L	Isoform 3 of Exportin-2 OS=Homo sapiens GN=CSE1L	2			2	2		
P60510	PP4C_HUMAN	35080	PPP4C	Serine/threonine-protein phosphatase 4 catalytic subunit OS=Homo sapiens GN=PPP4C PE=1 SV=1	2			2	2		
P62829	RL23_HUMAN	14865	RPL23	60S ribosomal protein L23 OS=Homo sapiens GN=RPL23 PE=1 SV=1	2			1	3		
Q01844-2	EWS_HUMAN	61217	EWSR1	Isoform EWS-B of RNA-binding protein EWS OS=Homo sapiens GN=EWSR1	2			3	1		
Q15493	RGN_HUMAN	33253	RGN	Regucalcin OS=Homo sapiens GN=RGN PE=1 SV=1	2			2	2		

Q6BCY4-2	NB5R2_HUMAN	27043	CYB5R2	Isoform 2 of NADH-cytochrome b5 reductase 2 OS=Homo sapiens GN=CYB5R2	2			2	2		
Q8N8S7-2	ENAH_HUMAN	63924	ENAH	Isoform 2 of Protein enabled homolog OS=Homo sapiens GN=ENAH	2			2	2		
Q92734-2	TFG_HUMAN	43020	TFG	Isoform 2 of Protein TFG OS=Homo sapiens GN=TFG	2			2	2		
Q96FE7-2	P3IP1_HUMAN	25320	PIK3IP1	Isoform 2 of Phosphoinositide-3-kinase-interacting protein 1 OS=Homo sapiens GN=PIK3IP1	2			2	2		
Q96GK7	FAH2A_HUMAN	34596	FAHD2A	Fumarylacetoacetate hydrolase domain-containing protein 2A OS=Homo sapiens GN=FAHD2A PE=1 SV=1	2			1	3		
Q9BXJ4-3	C1QT3_HUMAN	35183	C1QTNF3	Isoform 3 of Complement C1q tumor necrosis factor-related protein 3 OS=Homo sapiens GN=C1QTNF3	2			2	2		
Q9BZK7	TBL1R_HUMAN	55595	TBL1XR1	F-box-like/WD repeat-containing protein TBL1XR1 OS=Homo sapiens GN=TBL1XR1 PE=1 SV=1	2			1	3		
Q9NPH2-2	INO1_HUMAN	47146	ISYNA1	Isoform 2 of Inositol-3-phosphate synthase 1 OS=Homo sapiens GN=ISYNA1	2			3	1		
Q9NZM1-3	MYOF_HUMAN	233324	MYOF	Isoform 3 of Myoferlin OS=Homo sapiens GN=MYOF	2			2	2		
Q9UBF2	COPG2_HUMAN	97622	COPG2	Coatomer subunit gamma-2 OS=Homo sapiens GN=COPG2 PE=1 SV=1	2			2	2		
Q9UMS0-2	NFU1_HUMAN	12347	NFU1	Isoform 2 of NFU1 iron-sulfur cluster scaffold homolog, mitochondrial OS=Homo sapiens GN=NFU1	2			2	2		
Q9UMS4	PRP19_HUMAN	55181	PRPF19	Pre-mRNA-processing factor 19 OS=Homo sapiens GN=PRPF19 PE=1 SV=1	2			1	3		
Q9UNP9-2	PPIE_HUMAN	33085	PPIE	Isoform B of Peptidyl-prolyl cis-trans isomerase E OS=Homo sapiens GN=PPIE	2			1	3		
Q9Y275	TN13B_HUMAN	31223	TNFSF13B	Tumor necrosis factor ligand superfamily member 13B OS=Homo sapiens GN=TNFSF13B PE=1 SV=1	2			2	2		
O75629	CREG1_HUMAN	24075	CREG1	Protein CREG1 OS=Homo sapiens GN=CREG1 PE=1 SV=1	2			2		2	
P09497-2	CLCB_HUMAN	23181	CLTB	Isoform Non-brain of Clathrin light chain B OS=Homo sapiens GN=CLTB	2			2		2	
Q00325-2	MPCP_HUMAN	39959	SLC25A3	Isoform B of Phosphate carrier protein, mitochondrial OS=Homo sapiens GN=SLC25A3	2			3		1	
Q9NP61-2	ARFG3_HUMAN	51990	ARFGAP3	Isoform 2 of ADP-ribosylation factor GTPase-activating protein 3 OS=Homo sapiens GN=ARFGAP3	2				2	2	
P53602	MVD1_HUMAN	43405	MVD	Diphosphomevalonate decarboxylase OS=Homo sapiens GN=MVD PE=1 SV=1	2				2		2
Q9Y2G5-1	OFUT2_HUMAN	48896	POFUT2	Isoform A of GDP-fucose protein O-fucosyltransferase 2 OS=Homo sapiens GN=POFUT2	2				1		3
O75718	CRTAP_HUMAN	46562	CRTAP	Cartilage-associated protein OS=Homo sapiens GN=CRTAP PE=1 SV=1	2					1	3
P08254	MMP3_HUMAN	53977	MMP3	Stromelysin-1 OS=Homo sapiens GN=MMP3 PE=1 SV=2	2					1	3
Q10469	MGAT2_HUMAN	51550	MGAT2	Alpha-1,6-mannosyl-glycoprotein 2-beta-N-acetylglucosaminyltransferase OS=Homo sapiens GN=MGAT2 PE=1 SV=1	2					2	2
Q68BL8	OLM2B_HUMAN	83999	OLFML2B	Olfactomedin-like protein 2B OS=Homo sapiens GN=OLFML2B PE=2 SV=2	2					1	3
Q96HD1-2	CREL1_HUMAN	45942	CRELD1	Isoform 2 of Cysteine-rich with EGF-like domain protein 1 OS=Homo sapiens GN=CRELD1	2					3	1
Q96JY6-3	PDLI2_HUMAN	39181	PDLIM2	Isoform 3 of PDZ and LIM domain protein 2 OS=Homo sapiens GN=PDLIM2	2					2	2
Q9UBE0-2	SAE1_HUMAN	29811	SAE1	Isoform 2 of SUMO-activating enzyme subunit 1 OS=Homo sapiens GN=SAE1	2					2	2
P52746	ZN142_HUMAN	187880	ZNF142	Zinc finger protein 142 OS=Homo sapiens GN=ZNF142 PE=2 SV=4	1	4					
P01040	CYTA_HUMAN	11006	CSTA	Cystatin-A OS=Homo sapiens GN=CSTA PE=1 SV=1	1			4			
P02763	A1AG1_HUMAN	23512	ORM1	Alpha-1-acid glycoprotein 1 OS=Homo sapiens GN=ORM1 PE=1 SV=1	1			4			
Q5JRX3-2	PREP_HUMAN	117512	PITRM1	Isoform 2 of Presequence protease, mitochondrial OS=Homo sapiens GN=PITRM1	1			4			
O00151	PDLI1_HUMAN	36072	PDLIM1	PDZ and LIM domain protein 1 OS=Homo sapiens GN=PDLIM1 PE=1 SV=4	3	1	1	1			
Q08623-3	HDHD1_HUMAN	23283	HDHD1	Isoform 3 of Pseudouridine-5'-monophosphatase OS=Homo sapiens GN=HDHD1	3	1	1	1			
Q15286	RAB35_HUMAN	23025	RAB35	Ras-related protein Rab-35 OS=Homo sapiens GN=RAB35 PE=1 SV=1	3	1	1	1			
Q99456	K1C12_HUMAN	53511	KRT12	Keratin, type I cytoskeletal 12 OS=Homo sapiens GN=KRT12 PE=1 SV=1	3	1	1	1			
O95396	MOCS3_HUMAN	49669	MOCS3	Adenylyltransferase and sulfurtransferase MOCS3 OS=Homo sapiens GN=MOCS3 PE=1 SV=1	3	1	1		1		
O60763-2	USO1_HUMAN	109195	USO1	Isoform 2 of General vesicular transport factor p115 OS=Homo sapiens GN=USO1	3	1	1			1	
P20648	ATP4A_HUMAN	114119	ATP4A	Potassium-transporting ATPase alpha chain 1 OS=Homo sapiens GN=ATP4A PE=2 SV=5	3	1	1				1

P50993	AT1A2_HUMAN	112265	ATP1A2	Sodium/potassium-transporting ATPase subunit alpha-2 OS=Homo sapiens GN=ATP1A2 PE=1 SV=1	3	1	1				1
Q9Y5F9-2	PCDGI_HUMAN	89765	PCDHGB6	Isoform 2 of Protocadherin gamma-B6 OS=Homo sapiens GN=PCDHGB6	3	1	1				1
Q9Y5G1-2	PCDGF_HUMAN	89370	PCDHGB3	Isoform 2 of Protocadherin gamma-B3 OS=Homo sapiens GN=PCDHGB3	3	1	1				1
Q9Y5G6-2	PCDG7_HUMAN	89718	PCDHGA7	Isoform 2 of Protocadherin gamma-A7 OS=Homo sapiens GN=PCDHGA7	3	1	1				1
Q9Y5H1-2	PCDG2_HUMAN	90284	PCDHGA2	Isoform 2 of Protocadherin gamma-A2 OS=Homo sapiens GN=PCDHGA2	3	1	1				1
P14868	SYDC_HUMAN	57136	DARS	Aspartate--tRNA ligase, cytoplasmic OS=Homo sapiens GN=DARS PE=1 SV=2	3	1		1	1		
P16298-2	PP2BB_HUMAN	58013	PPP3CB	Isoform 2 of Serine/threonine-protein phosphatase 2B catalytic subunit beta isoform OS=Homo sapiens GN=PPP3CB	3	1		1	1		
Q8NG11-2	TSN14_HUMAN	28877	TSPAN14	Isoform 2 of Tetraspanin-14 OS=Homo sapiens GN=TSPAN14	3	1		1	1		
Q92804-2	RBP56_HUMAN	61558	TAF15	Isoform Short of TATA-binding protein-associated factor 2N OS=Homo sapiens GN=TAF15	3	1		1	1		
Q68CR9	Q68CR9_HUMAN	45771	DKFZp781B11202	Aspartate--tRNA ligase, cytoplasmic OS=Homo sapiens GN=DKFZp781B11202 PE=2 SV=1	3	1		1	1		
O14562	UBFD1_HUMAN	33382	UBFD1	Ubiquitin domain-containing protein UBFD1 OS=Homo sapiens GN=UBFD1 PE=1 SV=2	3	1		1		1	
Q8N196	SIX5_HUMAN	74562	SIX5	Homeobox protein SIX5 OS=Homo sapiens GN=SIX5 PE=1 SV=3	3	1		1			1
P08754	GNAI3_HUMAN	40532	GNAI3	Guanine nucleotide-binding protein G(k) subunit alpha OS=Homo sapiens GN=GNAI3 PE=1 SV=3	3	1			1	1	
Q9H0Q0	FA49A_HUMAN	37313	FAM49A	Protein FAM49A OS=Homo sapiens GN=FAM49A PE=1 SV=1	3	1			1	1	
Q5JXL7	Q5JXL7_HUMAN	10893	DKFZp586I031	Putative uncharacterized protein DKFZp586I031 OS=Homo sapiens GN=DKFZp586I031 PE=2 SV=1	3	1			1	1	
P08581-2	MET_HUMAN	157712	MET	Isoform 2 of Hepatocyte growth factor receptor OS=Homo sapiens GN=MET	3	1			1		1
O60220	TIM8A_HUMAN	10998	TIMM8A	Mitochondrial import inner membrane translocase subunit Tim8 A OS=Homo sapiens GN=TIMM8A PE=1 SV=1	3	1				1	1
P20290-2	BTF3_HUMAN	17699	BTF3	Isoform 2 of Transcription factor BTF3 OS=Homo sapiens GN=BTF3	3	1				1	1
Q9Y608-2	LRRF2_HUMAN	45414	LRRFIP2	Isoform 2 of Leucine-rich repeat flightless-interacting protein 2 OS=Homo sapiens GN=LRRFIP2	3	1				1	1
A2BFH1	PAL4G_HUMAN	18166	PPIAL4G	Peptidyl-prolyl cis-trans isomerase A-like 4G OS=Homo sapiens GN=PPIAL4G PE=2 SV=1	3		1	1			1
F5H284	PAL4D_HUMAN	18167	PPIAL4D	Peptidyl-prolyl cis-trans isomerase A-like 4D OS=Homo sapiens GN=PPIAL4D PE=3 SV=1	3		1	1			1
P61247	RS3A_HUMAN	29945	RPS3A	40S ribosomal protein S3a OS=Homo sapiens GN=RPS3A PE=1 SV=2	3		1		1	1	
O95490-2	LPHN2_HUMAN	157178	LPHN2	Isoform 2 of Latrophilin-2 OS=Homo sapiens GN=LPHN2	3		1			1	1
P48163	MAOX_HUMAN	64150	ME1	NADP-dependent malic enzyme OS=Homo sapiens GN=ME1 PE=1 SV=1	3		1			1	1
P16520	GBB3_HUMAN	37221	GNB3	Guanine nucleotide-binding protein G(I)/G(S)/G(T) subunit beta-3 OS=Homo sapiens GN=GNB3 PE=1 SV=1	3			1	1	1	
P56134-2	ATPK_HUMAN	10363	ATP5J2	Isoform 2 of ATP synthase subunit f, mitochondrial OS=Homo sapiens GN=ATP5J2	3			1	1	1	
Q13753-2	LAMC2_HUMAN	121604	LAMC2	Isoform Short of Laminin subunit gamma-2 OS=Homo sapiens GN=LAMC2	3			1	1	1	
Q86V88	MGDP1_HUMAN	20109	MDP1	Magnesium-dependent phosphatase 1 OS=Homo sapiens GN=MDP1 PE=1 SV=1	3			1	1	1	
Q8WVY7	UBCP1_HUMAN	36805	UBLCP1	Ubiquitin-like domain-containing CTD phosphatase 1 OS=Homo sapiens GN=UBLCP1 PE=1 SV=2	3			1	1	1	
Q9C0K3	ARP3C_HUMAN	23712	ACTR3C	Actin-related protein 3C OS=Homo sapiens GN=ACTR3C PE=2 SV=1	3			1	1	1	
Q9NQP4	PFD4_HUMAN	15314	PFDN4	Prefoldin subunit 4 OS=Homo sapiens GN=PFDN4 PE=1 SV=1	3			1	1	1	
C9JJT5	C9JJT5_HUMAN	5915	ATP5J2-PTCD1	Protein ATP5J2-PTCD1 OS=Homo sapiens GN=ATP5J2-PTCD1 PE=2 SV=2	3			1	1	1	
O14929-2	HAT1_HUMAN	39786	HAT1	Isoform B of Histone acetyltransferase type B catalytic subunit OS=Homo sapiens GN=HAT1	3			1	1		1
O75608-2	LYPA1_HUMAN	22875	LYPLA1	Isoform 2 of Acyl-protein thioesterase 1 OS=Homo sapiens GN=LYPLA1	3			1	1		1
P04275	VWF_HUMAN	309265	VWF	von Willebrand factor OS=Homo sapiens GN=VWF PE=1 SV=4	3			1	1		1
Q7Z3S9-2	NT2NL_HUMAN	24947	NOTCH2NL	Isoform 2 of Notch homolog 2 N-terminal-like protein OS=Homo sapiens GN=NOTCH2NL	3			1	1		1
P23468-3	PTPRD_HUMAN	156088	PTPRD	Isoform 3 of Receptor-type tyrosine-protein phosphatase delta OS=Homo sapiens	3			1		1	1

				GN=PTPRD							
Q07157-2	ZO1_HUMAN	186966	TJP1	Isoform Short of Tight junction protein ZO-1 OS=Homo sapiens GN=TJP1	3			1		1	1
Q96IX5	USMG5_HUMAN	6458	USMG5	Up-regulated during skeletal muscle growth protein 5 OS=Homo sapiens GN=USMG5 PE=1 SV=1	3			1		1	1
Q9HC56-2	PCDH9_HUMAN	132251	PCDH9	Isoform 2 of Protocadherin-9 OS=Homo sapiens GN=PCDH9	3				1	1	1
Q16595-2	FRDA_HUMAN	19095	FXN	Isoform 2 of Frataxin, mitochondrial OS=Homo sapiens GN=FXN	2	1	2				
Q53FT3	HIKES_HUMAN	21628	C11orf73	Protein Hikeshi OS=Homo sapiens GN=C11orf73 PE=1 SV=2	2	2	1				
P53992	SC24C_HUMAN	118325	SEC24C	Protein transport protein Sec24C OS=Homo sapiens GN=SEC24C PE=1 SV=3	2	2		1			
Q96L46	CPNS2_HUMAN	27660	CAPNS2	Calpain small subunit 2 OS=Homo sapiens GN=CAPNS2 PE=2 SV=2	2	1		2			
P02790	HEMO_HUMAN	51676	HPX	Hemopexin OS=Homo sapiens GN=HPX PE=1 SV=2	2	1			2		
P04424-2	ARLY_HUMAN	49509	ASL	Isoform 2 of Argininosuccinate lyase OS=Homo sapiens GN=ASL	2	2			1		
Q9HD42	CHM1A_HUMAN	21703	CHMP1A	Charged multivesicular body protein 1a OS=Homo sapiens GN=CHMP1A PE=1 SV=1	2	2			1		
Q15637-2	SF01_HUMAN	68633	SF1	Isoform 2 of Splicing factor 1 OS=Homo sapiens GN=SF1	2	2				1	
O60687	SRPX2_HUMAN	52972	SRPX2	Sushi repeat-containing protein SRPX2 OS=Homo sapiens GN=SRPX2 PE=1 SV=1	2		2	1			
O75962-2	TRIO_HUMAN	329389	TRIO	Isoform 2 of Triple functional domain protein OS=Homo sapiens GN=TRIO	2		2	1			
Q5VY80	RET1L_HUMAN	27509	RAET1L	Retinoic acid early transcript 1L protein OS=Homo sapiens GN=RAET1L PE=2 SV=1	2		2			1	
Q6H3X3-2	RET1G_HUMAN	23884	RAET1G	Isoform 2 of Retinoic acid early transcript 1G protein OS=Homo sapiens GN=RAET1G	2		2			1	
Q86Y82	STX12_HUMAN	31642	STX12	Syntaxin-12 OS=Homo sapiens GN=STX12 PE=1 SV=1	2		1			2	
O14618	CCS_HUMAN	29041	CCS	Copper chaperone for superoxide dismutase OS=Homo sapiens GN=CCS PE=1 SV=1	2			2	1		
O15212	PFD6_HUMAN	14583	PFDN6	Prefoldin subunit 6 OS=Homo sapiens GN=PFDN6 PE=1 SV=1	2			2	1		
O60218	AK1BA_HUMAN	36020	AKR1B10	Aldo-keto reductase family 1 member B10 OS=Homo sapiens GN=AKR1B10 PE=1 SV=2	2			2	1		
O60907-2	TBL1X_HUMAN	57049	TBL1X	Isoform 2 of F-box-like/WD repeat-containing protein TBL1X OS=Homo sapiens GN=TBL1X	2			1	2		
O95747	OXSR1_HUMAN	58022	OXSR1	Serine/threonine-protein kinase OSR1 OS=Homo sapiens GN=OXSR1 PE=1 SV=1	2			2	1		
O95825	QORL1_HUMAN	38697	CRYZL1	Quinone oxidoreductase-like protein 1 OS=Homo sapiens GN=CRYZL1 PE=1 SV=2	2			1	2		
P01583	IL1A_HUMAN	30607	IL1A	Interleukin-1 alpha OS=Homo sapiens GN=IL1A PE=1 SV=1	2			1	2		
P01860	IGHG3_HUMAN	41287	IGHG3	Ig gamma-3 chain C region OS=Homo sapiens GN=IGHG3 PE=1 SV=2	2			2	1		
P01861	IGHG4_HUMAN	35941	IGHG4	Ig gamma-4 chain C region OS=Homo sapiens GN=IGHG4 PE=1 SV=1	2			1	2		
P04818	TYSY_HUMAN	35716	TYMS	Thymidylate synthase OS=Homo sapiens GN=TYMS PE=1 SV=3	2			1	2		
P05386	RLA1_HUMAN	11514	RPLP1	60S acidic ribosomal protein P1 OS=Homo sapiens GN=RPLP1 PE=1 SV=1	2			1	2		
P07738	PMGE_HUMAN	30005	BPGM	Bisphosphoglycerate mutase OS=Homo sapiens GN=BPGM PE=1 SV=2	2			2	1		
P11233	RALA_HUMAN	23567	RALA	Ras-related protein Ral-A OS=Homo sapiens GN=RALA PE=1 SV=1	2			1	2		
P11234	RALB_HUMAN	23409	RALB	Ras-related protein Ral-B OS=Homo sapiens GN=RALB PE=1 SV=1	2			1	2		
P13164	IFM1_HUMAN	13964	IFITM1	Interferon-induced transmembrane protein 1 OS=Homo sapiens GN=IFITM1 PE=1 SV=3	2			1	2		
P13716-2	HEM2_HUMAN	39034	ALAD	Isoform 2 of Delta-aminolevulinic acid dehydratase OS=Homo sapiens GN=ALAD	2			1	2		
P18124	RL7_HUMAN	29226	RPL7	60S ribosomal protein L7 OS=Homo sapiens GN=RPL7 PE=1 SV=1	2			1	2		
P30519	HMOX2_HUMAN	36033	HMOX2	Heme oxygenase 2 OS=Homo sapiens GN=HMOX2 PE=1 SV=2	2			1	2		
P45880-1	VDAC2_HUMAN	33372	VDAC2	Isoform 1 of Voltage-dependent anion-selective channel protein 2 OS=Homo sapiens GN=VDAC2	2			2	1		
P49354	FNTA_HUMAN	44409	FNTA	Protein farnesyltransferase/geranylgeranyltransferase type-1 subunit alpha OS=Homo sapiens GN=FNTA PE=1 SV=1	2			1	2		
Q00266	METK1_HUMAN	43648	MAT1A	S-adenosylmethionine synthase isoform type-1 OS=Homo sapiens GN=MAT1A PE=1 SV=2	2			2	1		
Q01628	IFM3_HUMAN	14632	IFITM3	Interferon-induced transmembrane protein 3 OS=Homo sapiens GN=IFITM3 PE=1 SV=2	2			1	2		
Q01629	IFM2_HUMAN	14632	IFITM2	Interferon-induced transmembrane protein 2 OS=Homo sapiens GN=IFITM2 PE=1 SV=2	2			1	2		
Q02543	RL18A_HUMAN	20762	RPL18A	60S ribosomal protein L18a OS=Homo sapiens GN=RPL18A PE=1 SV=2	2			1	2		
Q12860-2	CNTN1_HUMAN	111867	CNTN1	Isoform 2 of Contactin-1 OS=Homo sapiens GN=CNTN1	2			2	1		

Q13442	HAP28_HUMAN	20630	PDAP1	28 kDa heat- and acid-stable phosphoprotein OS=Homo sapiens GN=PDAP1 PE=1 SV=1	2			1	2		
Q15019-2	SEPT2_HUMAN	45461	SEPT2	Isoform 2 of Septin-2 OS=Homo sapiens GN=SEPT2	2			2	1		
Q15067-2	ACOX1_HUMAN	74668	ACOX1	Isoform 2 of Peroxisomal acyl-coenzyme A oxidase 1 OS=Homo sapiens GN=ACOX1	2			1	2		
Q15075	EEA1_HUMAN	162466	EEA1	Early endosome antigen 1 OS=Homo sapiens GN=EEA1 PE=1 SV=2	2			1	2		
Q2TV78-2	MST1L_HUMAN	76784	MST1L	Isoform 2 of Putative macrophage stimulating 1-like protein OS=Homo sapiens GN=MST1L	2			1	2		
Q5TFE4	NT5D1_HUMAN	51845	NT5DC1	5'-nucleotidase domain-containing protein 1 OS=Homo sapiens GN=NT5DC1 PE=1 SV=1	2			2	1		
Q6NVY1	HIBCH_HUMAN	43482	HIBCH	3-hydroxyisobutyryl-CoA hydrolase, mitochondrial OS=Homo sapiens GN=HIBCH PE=1 SV=2	2			1	2		
Q86X76-2	NIT1_HUMAN	31859	NIT1	Isoform 1 of Nitrilase homolog 1 OS=Homo sapiens GN=NIT1	2			1	2		
Q8N8U9	BMPER_HUMAN	75997	BMPER	BMP-binding endothelial regulator protein OS=Homo sapiens GN=BMPER PE=1 SV=3	2			1	2		
Q8WUW1-2	BRK1_HUMAN	12046	BRK1	Isoform 2 of Protein BRICK1 OS=Homo sapiens GN=BRK1	2			1	2		
Q92783-2	STAM1_HUMAN	44972	STAM	Isoform 2 of Signal transducing adapter molecule 1 OS=Homo sapiens GN=STAM	2			2	1		
Q9GZS3	WDR61_HUMAN	33581	WDR61	WD repeat-containing protein 61 OS=Homo sapiens GN=WDR61 PE=1 SV=1	2			2	1		
Q9NTU7	CBLN4_HUMAN	21808	CBLN4	Cerebellin-4 OS=Homo sapiens GN=CBLN4 PE=1 SV=1	2			1	2		
Q9Y224	CN166_HUMAN	28068	C14orf166	UPF0568 protein C14orf166 OS=Homo sapiens GN=C14orf166 PE=1 SV=1	2			2	1		
P49593	PPM1F_HUMAN	49831	PPM1F	Protein phosphatase 1F OS=Homo sapiens GN=PPM1F PE=1 SV=3	2			1		2	
P61106	RAB14_HUMAN	23897	RAB14	Ras-related protein Rab-14 OS=Homo sapiens GN=RAB14 PE=1 SV=4	2			1		2	
P61626	LYSC_HUMAN	16537	LYZ	Lysozyme C OS=Homo sapiens GN=LYZ PE=1 SV=1	2			2		1	
P62266	RS23_HUMAN	15808	RPS23	40S ribosomal protein S23 OS=Homo sapiens GN=RPS23 PE=1 SV=3	2			1		2	
Q6ZRF8	RN207_HUMAN	70861	RNF207	RING finger protein 207 OS=Homo sapiens GN=RNF207 PE=2 SV=2	2			1		2	
Q9UGM3-2	DMBT1_HUMAN	193971	DMBT1	Isoform 2 of Deleted in malignant brain tumors 1 protein OS=Homo sapiens GN=DMBT1	2			1		2	
Q68CR8	Q68CR8_HUMAN	11739	DKFZp781M17165	Putative uncharacterized protein DKFZp781M17165 OS=Homo sapiens GN=DKFZp781M17165 PE=2 SV=1	2			2		1	
O00231-2	PSD11_HUMAN	47535	PSMD11	Isoform 2 of 26S proteasome non-ATPase regulatory subunit 11 OS=Homo sapiens GN=PSMD11	2			1			2
Q07955-2	SRSF1_HUMAN	31999	SRSF1	Isoform ASF-2 of Serine/arginine-rich splicing factor 1 OS=Homo sapiens GN=SRSF1	2			2			1
Q6VMQ6-2	MCAF1_HUMAN	118654	ATF7IP	Isoform 2 of Activating transcription factor 7-interacting protein 1 OS=Homo sapiens GN=ATF7IP	2			1			2
Q99832-2	TCPH_HUMAN	37499	CCT7	Isoform 2 of T-complex protein 1 subunit eta OS=Homo sapiens GN=CCT7	2			2			1
O14980	XPO1_HUMAN	123386	XPO1	Exportin-1 OS=Homo sapiens GN=XPO1 PE=1 SV=1	2				1	2	
O60701-2	UGDH_HUMAN	47603	UGDH	Isoform 2 of UDP-glucose 6-dehydrogenase OS=Homo sapiens GN=UGDH	2				1	2	
O75643-2	U520_HUMAN	71472	SNRNP200	Isoform 2 of U5 small nuclear ribonucleoprotein 200 kDa helicase OS=Homo sapiens GN=SNRNP200	2				2	1	
Q9Y2T2	AP3M1_HUMAN	46939	AP3M1	AP-3 complex subunit mu-1 OS=Homo sapiens GN=AP3M1 PE=1 SV=1	2				2	1	
Q5HYE9	Q5HYE9_HUMAN	13874	DKFZp313K0921	Putative uncharacterized protein DKFZp313K0921 OS=Homo sapiens GN=DKFZp313K0921 PE=2 SV=1	2				2	1	
P60673	PROF3_HUMAN	14596	PFN3	Profilin-3 OS=Homo sapiens GN=PFN3 PE=2 SV=1	2				2		1
O60513	B4GT4_HUMAN	40041	B4GALT4	Beta-1,4-galactosyltransferase 4 OS=Homo sapiens GN=B4GALT4 PE=1 SV=1	2					1	2
Q00341-2	VIGLN_HUMAN	137987	HDLBP	Isoform 2 of Vigilin OS=Homo sapiens GN=HDLBP	2					2	1
Q2TVT4	KGFL1_HUMAN	11079	KGFLP1	Putative keratinocyte growth factor-like protein 1 OS=Homo sapiens GN=KGFLP1 PE=5 SV=1	2					1	2
Q495W5-2	FUT11_HUMAN	53510	FUT11	Isoform 2 of Alpha-(1,3)-fucosyltransferase 11 OS=Homo sapiens GN=FUT11	2					2	1
Q9HCL0-2	PCD18_HUMAN	126021	PCDH18	Isoform 2 of Protocadherin-18 OS=Homo sapiens GN=PCDH18	2					2	1
Q9NRR1	CYTL1_HUMAN	15577	CYTL1	Cytokine-like protein 1 OS=Homo sapiens GN=CYTL1 PE=1 SV=1	2					1	2
Q9NYJ1	COA4_HUMAN	10134	COA4	Cytochrome c oxidase assembly factor 4 homolog, mitochondrial OS=Homo sapiens GN=COA4 PE=1 SV=2	2					1	2

Q9Y230	RUVB2_HUMAN	51157	RUVBL2	RuvB-like 2 OS=Homo sapiens GN=RUVBL2 PE=1 SV=3	2					2	1
P30153	2AAA_HUMAN	65309	PPP2R1A	Serine/threonine-protein phosphatase 2A 65 kDa regulatory subunit A alpha isoform OS=Homo sapiens GN=PPP2R1A PE=1 SV=4	1	3					
Q562E7-4	WDR81_HUMAN	197997	WDR81	Isoform 4 of WD repeat-containing protein 81 OS=Homo sapiens GN=WDR81	1	3					
Q96T21-2	SEBP2_HUMAN	87393	SECISBP2	Isoform 2 of Selenocysteine insertion sequence-binding protein 2 OS=Homo sapiens GN=SECISBP2	1	3					
O94875-11	SRBS2_HUMAN	134594	SORBS2	Isoform 11 of Sorbin and SH3 domain-containing protein 2 OS=Homo sapiens GN=SORBS2	1		3				
P04003	C4BPA_HUMAN	67033	C4BPA	C4b-binding protein alpha chain OS=Homo sapiens GN=C4BPA PE=1 SV=2	1			3			
P19652	A1AG2_HUMAN	23603	ORM2	Alpha-1-acid glycoprotein 2 OS=Homo sapiens GN=ORM2 PE=1 SV=2	1			3			
P21796	VDAC1_HUMAN	30773	VDAC1	Voltage-dependent anion-selective channel protein 1 OS=Homo sapiens GN=VDAC1 PE=1 SV=2	1			3			
Q14204	DYHC1_HUMAN	532408	DYNC1H1	Cytoplasmic dynein 1 heavy chain 1 OS=Homo sapiens GN=DYNC1H1 PE=1 SV=5	1			3			
P0CG05	LAC2_HUMAN	11294	IGLC2	Ig lambda-2 chain C regions OS=Homo sapiens GN=IGLC2 PE=1 SV=1	1				3		
P0CG06	LAC3_HUMAN	11238	IGLC3	Ig lambda-3 chain C regions OS=Homo sapiens GN=IGLC3 PE=1 SV=1	1				3		
P25205	MCM3_HUMAN	90981	MCM3	DNA replication licensing factor MCM3 OS=Homo sapiens GN=MCM3 PE=1 SV=3	1				3		
P25940	CO5A3_HUMAN	172121	COL5A3	Collagen alpha-3(V) chain OS=Homo sapiens GN=COL5A3 PE=1 SV=3	1				3		
Q5T749	KPRP_HUMAN	64136	KPRP	Keratinocyte proline-rich protein OS=Homo sapiens GN=KPRP PE=1 SV=1	1				3		
A6ND91	ASPD_HUMAN	29946	ASPDH	Putative L-aspartate dehydrogenase OS=Homo sapiens GN=ASPDH PE=2 SV=2	1					3	
P04062-2	GLCM_HUMAN	57455	GBA	Isoform Short of Glucosylceramidase OS=Homo sapiens GN=GBA	1					3	
P10720	PF4V_HUMAN	11553	PF4V1	Platelet factor 4 variant OS=Homo sapiens GN=PF4V1 PE=1 SV=1	1					3	
P62888	RL30_HUMAN	12784	RPL30	60S ribosomal protein L30 OS=Homo sapiens GN=RPL30 PE=1 SV=2	1					3	
Q8N414	PGBD5_HUMAN	51642	PGBD5	PiggyBac transposable element-derived protein 5 OS=Homo sapiens GN=PGBD5 PE=1 SV=3	1						3
P23193-2	TCEA1_HUMAN	31664	TCEA1	Isoform 2 of Transcription elongation factor A protein 1 OS=Homo sapiens GN=TCEA1	2	1	1				
Q6UX15-2	LAYN_HUMAN	42281	LAYN	Isoform 2 of Layilin OS=Homo sapiens GN=LAYN	2	1	1				
Q86SF2	GALT7_HUMAN	75389	GALNT7	N-acetylgalactosaminyltransferase 7 OS=Homo sapiens GN=GALNT7 PE=1 SV=1	2	1	1				
Q8IXL6	DMP4_HUMAN	66234	FAM20C	Extracellular serine/threonine protein kinase FAM20C OS=Homo sapiens GN=FAM20C PE=1 SV=2	2	1	1				
Q96CV9-2	OPTN_HUMAN	65202	OPTN	Isoform 2 of Optineurin OS=Homo sapiens GN=OPTN	2	1	1				
Q96S97	MYADM_HUMAN	35274	MYADM	Myeloid-associated differentiation marker OS=Homo sapiens GN=MYADM PE=1 SV=2	2	1	1				
Q9H444	CHM4B_HUMAN	24950	CHMP4B	Charged multivesicular body protein 4b OS=Homo sapiens GN=CHMP4B PE=1 SV=1	2	1	1				
Q9H772	GREM2_HUMAN	19320	GREM2	Gremlin-2 OS=Homo sapiens GN=GREM2 PE=1 SV=1	2	1	1				
Q9Y561-2	LRP12_HUMAN	93123	LRP12	Isoform 2 of Low-density lipoprotein receptor-related protein 12 OS=Homo sapiens GN=LRP12	2	1	1				
Q9Y6L7	TLL2_HUMAN	113557	TLL2	Tolloid-like protein 2 OS=Homo sapiens GN=TLL2 PE=1 SV=1	2	1	1				
O15427	MOT4_HUMAN	49469	SLC16A3	Monocarboxylate transporter 4 OS=Homo sapiens GN=SLC16A3 PE=1 SV=1	2	1		1			
P38646	GRP75_HUMAN	73680	HSPA9	Stress-70 protein, mitochondrial OS=Homo sapiens GN=HSPA9 PE=1 SV=2	2	1		1			
Q13033-2	STRN3_HUMAN	77745	STRN3	Isoform Alpha of Striatin-3 OS=Homo sapiens GN=STRN3	2	1		1			
Q8NC54	KCT2_HUMAN	29235	KCT2	Keratinocyte-associated transmembrane protein 2 OS=Homo sapiens GN=KCT2 PE=2 SV=2	2	1		1			
Q92945	FUBP2_HUMAN	73115	KHSRP	Far upstream element-binding protein 2 OS=Homo sapiens GN=KHSRP PE=1 SV=4	2	1		1			
Q96F45-2	ZN503_HUMAN	59292	ZNF503	Isoform 2 of Zinc finger protein 503 OS=Homo sapiens GN=ZNF503	2	1		1			
P63096-2	GNAI1_HUMAN	34775	GNAI1	Isoform 2 of Guanine nucleotide-binding protein G(i) subunit alpha-1 OS=Homo sapiens GN=GNAI1	2	1			1		
P82979	SARNP_HUMAN	23671	SARNP	SAP domain-containing ribonucleoprotein OS=Homo sapiens GN=SARNP PE=1 SV=3	2	1			1		
Q9NQ30	ESM1_HUMAN	20095	ESM1	Endothelial cell-specific molecule 1 OS=Homo sapiens GN=ESM1 PE=1 SV=2	2	1			1		
P49221	TGM4_HUMAN	77145	TGM4	Protein-glutamine gamma-glutamyltransferase 4 OS=Homo sapiens GN=TGM4 PE=1 SV=2	2	1					1

Q14789-2	GOGB1_HUMAN	377137	GOLGB1	Isoform 2 of Golgin subfamily B member 1 OS=Homo sapiens GN=GOLGB1	2		1	1			
O60832	DKC1_HUMAN	57674	DKC1	H/ACA ribonucleoprotein complex subunit 4 OS=Homo sapiens GN=DKC1 PE=1 SV=3	2		1			1	
P33176	KINH_HUMAN	109685	KIF5B	Kinesin-1 heavy chain OS=Homo sapiens GN=KIF5B PE=1 SV=1	2		1			1	
P58397-3	ATS12_HUMAN	168460	ADAMTS12	Isoform 3 of A disintegrin and metalloproteinase with thrombospondin motifs 12 OS=Homo sapiens GN=ADAMTS12	2		1			1	
Q14232	EI2BA_HUMAN	33712	EIF2B1	Translation initiation factor eIF-2B subunit alpha OS=Homo sapiens GN=EIF2B1 PE=1 SV=1	2		1			1	
Q9UEU0	VTI1B_HUMAN	26688	VTI1B	Vesicle transport through interaction with t-SNAREs homolog 1B OS=Homo sapiens GN=VTI1B PE=1 SV=3	2		1			1	
Q13542	4EBP2_HUMAN	12939	EIF4EBP2	Eukaryotic translation initiation factor 4E-binding protein 2 OS=Homo sapiens GN=EIF4EBP2 PE=1 SV=1	2		1				1
Q16401-2	PSMD5_HUMAN	51312	PSMD5	Isoform 2 of 26S proteasome non-ATPase regulatory subunit 5 OS=Homo sapiens GN=PSMD5	2		1				1
O43488	ARK72_HUMAN	39589	AKR7A2	Aflatoxin B1 aldehyde reductase member 2 OS=Homo sapiens GN=AKR7A2 PE=1 SV=3	2			1	1		
O43809	CPSF5_HUMAN	26227	NUDT21	Cleavage and polyadenylation specificity factor subunit 5 OS=Homo sapiens GN=NUDT21 PE=1 SV=1	2			1	1		
O75144-2	ICOSL_HUMAN	34225	ICOSLG	Isoform 2 of ICOS ligand OS=Homo sapiens GN=ICOSLG	2			1	1		
O75475-2	PSIP1_HUMAN	37725	PSIP1	Isoform 2 of PC4 and SFRS1-interacting protein OS=Homo sapiens GN=PSIP1	2			1	1		
O75508	CLD11_HUMAN	21993	CLDN11	Claudin-11 OS=Homo sapiens GN=CLDN11 PE=1 SV=2	2			1	1		
P02689	MYP2_HUMAN	14909	PMP2	Myelin P2 protein OS=Homo sapiens GN=PMP2 PE=1 SV=3	2			1	1		
P05198	IF2A_HUMAN	36112	EIF2S1	Eukaryotic translation initiation factor 2 subunit 1 OS=Homo sapiens GN=EIF2S1 PE=1 SV=3	2			1	1		
P11216	PYGB_HUMAN	96696	PYGB	Glycogen phosphorylase, brain form OS=Homo sapiens GN=PYGB PE=1 SV=5	2			1	1		
P13671	CO6_HUMAN	104786	C6	Complement component C6 OS=Homo sapiens GN=C6 PE=1 SV=3	2			1	1		
P15090	FABP4_HUMAN	14719	FABP4	Fatty acid-binding protein, adipocyte OS=Homo sapiens GN=FABP4 PE=1 SV=3	2			1	1		
P15313	VATB1_HUMAN	56833	ATP6V1B1	V-type proton ATPase subunit B, kidney isoform OS=Homo sapiens GN=ATP6V1B1 PE=1 SV=3	2			1	1		
P16112-2	PGCA_HUMAN	246306	ACAN	Isoform 2 of Aggrecan core protein OS=Homo sapiens GN=ACAN	2			1	1		
P22352	GPX3_HUMAN	25402	GPX3	Glutathione peroxidase 3 OS=Homo sapiens GN=GPX3 PE=1 SV=2	2			1	1		
P34896-2	GLYC_HUMAN	49028	SHMT1	Isoform 2 of Serine hydroxymethyltransferase, cytosolic OS=Homo sapiens GN=SHMT1	2			1	1		
P48507	GSH0_HUMAN	30727	GCLM	Glutamate--cysteine ligase regulatory subunit OS=Homo sapiens GN=GCLM PE=1 SV=1	2			1	1		
P49753	ACOT2_HUMAN	53218	ACOT2	Acyl-coenzyme A thioesterase 2, mitochondrial OS=Homo sapiens GN=ACOT2 PE=1 SV=6	2			1	1		
P50991-2	TCPD_HUMAN	54720	CCT4	Isoform 2 of T-complex protein 1 subunit delta OS=Homo sapiens GN=CCT4	2			1	1		
P53041	PPP5_HUMAN	56879	PPP5C	Serine/threonine-protein phosphatase 5 OS=Homo sapiens GN=PPP5C PE=1 SV=1	2			1	1		
P53597	SUCA_HUMAN	36250	SUCLG1	Succinyl-CoA ligase [ADP/GDP-forming] subunit alpha, mitochondrial OS=Homo sapiens GN=SUCLG1 PE=1 SV=4	2			1	1		
P63165	SUMO1_HUMAN	11557	SUMO1	Small ubiquitin-related modifier 1 OS=Homo sapiens GN=SUMO1 PE=1 SV=1	2			1	1		
Q03135-2	CAV1_HUMAN	17023	CAV1	Isoform Beta of Caveolin-1 OS=Homo sapiens GN=CAV1	2			1	1		
Q13576-2	IQGA2_HUMAN	122805	IQGAP2	Isoform 2 of Ras GTPase-activating-like protein IQGAP2 OS=Homo sapiens GN=IQGAP2	2			1	1		
Q15654	TRIP6_HUMAN	50288	TRIP6	Thyroid receptor-interacting protein 6 OS=Homo sapiens GN=TRIP6 PE=1 SV=3	2			1	1		
Q29RF7	PDS5A_HUMAN	150830	PDS5A	Sister chromatid cohesion protein PDS5 homolog A OS=Homo sapiens GN=PDS5A PE=1 SV=1	2			1	1		
Q6P1J9	CDC73_HUMAN	60577	CDC73	Parafibromin OS=Homo sapiens GN=CDC73 PE=1 SV=1	2			1	1		
Q6P2I3	FAH2B_HUMAN	34613	FAHD2B	Fumarylacetoacetate hydrolase domain-containing protein 2B OS=Homo sapiens GN=FAHD2B PE=2 SV=1	2			1	1		
Q6ZMU1	C3P1_HUMAN	40197	C3P1	Putative protein C3P1 OS=Homo sapiens GN=C3P1 PE=5 SV=3	2			1	1		
Q7Z4H3-2	HDDC2_HUMAN	19550	HDDC2	Isoform 2 of HD domain-containing protein 2 OS=Homo sapiens GN=HDDC2	2			1	1		

Q86TX2	ACOT1_HUMAN	46277	ACOT1	Acyl-coenzyme A thioesterase 1 OS=Homo sapiens GN=ACOT1 PE=1 SV=1	2			1	1		
Q86VI3	IQGA3_HUMAN	184699	IQGAP3	Ras GTPase-activating-like protein IQGAP3 OS=Homo sapiens GN=IQGAP3 PE=1 SV=2	2			1	1		
Q86WA6-2	BPHL_HUMAN	31107	BPHL	Isoform 2 of Valacyclovir hydrolase OS=Homo sapiens GN=BPHL	2			1	1		
Q8NC96-2	NECP1_HUMAN	11444	NECAP1	Isoform 2 of Adaptin ear-binding coat-associated protein 1 OS=Homo sapiens GN=NECAP1	2			1	1		
Q8NHP1	ARK74_HUMAN	36814	AKR7L	Aflatoxin B1 aldehyde reductase member 4 OS=Homo sapiens GN=AKR7L PE=2 SV=6	2			1	1		
Q8WUA2	PPIL4_HUMAN	57225	PPIL4	Peptidyl-prolyl cis-trans isomerase-like 4 OS=Homo sapiens GN=PPIL4 PE=1 SV=1	2			1	1		
Q93091	RNAS6_HUMAN	17196	RNASE6	Ribonuclease K6 OS=Homo sapiens GN=RNASE6 PE=1 SV=2	2			1	1		
Q96FX2-2	DPH3_HUMAN	6503	DPH3	Isoform 2 of DPH3 homolog OS=Homo sapiens GN=DPH3	2			1	1		
Q96KG7-2	MEG10_HUMAN	60797	MEGF10	Isoform 2 of Multiple epidermal growth factor-like domains protein 10 OS=Homo sapiens GN=MEGF10	2			1	1		
Q96LR5	UB2E2_HUMAN	22255	UBE2E2	Ubiquitin-conjugating enzyme E2 E2 OS=Homo sapiens GN=UBE2E2 PE=1 SV=1	2			1	1		
Q9BQ87	TBL1Y_HUMAN	56688	TBL1Y	F-box-like/WD repeat-containing protein TBL1Y OS=Homo sapiens GN=TBL1Y PE=2 SV=1	2			1	1		
Q9BVG4	PBDC1_HUMAN	26057	PBDC1	Protein PBDC1 OS=Homo sapiens GN=PBDC1 PE=1 SV=1	2			1	1		
Q9BX67-2	JAM3_HUMAN	29223	JAM3	Isoform 2 of Junctional adhesion molecule C OS=Homo sapiens GN=JAM3	2			1	1		
Q9H115	SNAB_HUMAN	33557	NAPB	Beta-soluble NSF attachment protein OS=Homo sapiens GN=NAPB PE=1 SV=2	2			1	1		
Q9NTZ6	RBM12_HUMAN	97395	RBM12	RNA-binding protein 12 OS=Homo sapiens GN=RBM12 PE=1 SV=1	2			1	1		
Q9NYU1	UGGG2_HUMAN	174735	UGGT2	UDP-glucose:glycoprotein glucosyltransferase 2 OS=Homo sapiens GN=UGGT2 PE=1 SV=4	2			1	1		
Q9UNK4	PA2GD_HUMAN	16546	PLA2G2D	Group IID secretory phospholipase A2 OS=Homo sapiens GN=PLA2G2D PE=1 SV=2	2			1	1		
Q9UPU3	SORC3_HUMAN	135755	SORCS3	VPS10 domain-containing receptor SorCS3 OS=Homo sapiens GN=SORCS3 PE=2 SV=2	2			1	1		
Q9NRI2	Q9NRI2_HUMAN	4575	TRAX	Disrupted in schizophrenia 1 isoform 52 (Fragment) OS=Homo sapiens GN=TRAX PE=2 SV=1	2			1	1		
O75506	HSBP1_HUMAN	8544	HSBP1	Heat shock factor-binding protein 1 OS=Homo sapiens GN=HSBP1 PE=1 SV=1	2			1		1	
P10109	ADX_HUMAN	19393	FDX1	Adrenodoxin, mitochondrial OS=Homo sapiens GN=FDX1 PE=1 SV=1	2			1		1	
P23919	KTHY_HUMAN	23819	DTYMK	Thymidylate kinase OS=Homo sapiens GN=DTYMK PE=1 SV=4	2			1		1	
P83731	RL24_HUMAN	17779	RPL24	60S ribosomal protein L24 OS=Homo sapiens GN=RPL24 PE=1 SV=1	2			1		1	
Q02985-2	FHR3_HUMAN	30720	CFHR3	Isoform 2 of Complement factor H-related protein 3 OS=Homo sapiens GN=CFHR3	2			1		1	
Q6V0I7-3	FAT4_HUMAN	542616	FAT4	Isoform 3 of Protocadherin Fat 4 OS=Homo sapiens GN=FAT4	2			1		1	
Q7Z4H8	KDEL2_HUMAN	58572	KDELC2	KDEL motif-containing protein 2 OS=Homo sapiens GN=KDELC2 PE=1 SV=2	2			1		1	
Q8N1G1	REXO1_HUMAN	131509	REXO1	RNA exonuclease 1 homolog OS=Homo sapiens GN=REXO1 PE=1 SV=3	2			1		1	
Q9NWM8	FKB14_HUMAN	24172	FKBP14	Peptidyl-prolyl cis-trans isomerase FKBP14 OS=Homo sapiens GN=FKBP14 PE=1 SV=1	2			1		1	
Q9P035	HACD3_HUMAN	43160	PTPLAD1	Very-long-chain (3R)-3-hydroxyacyl-[acyl-carrier protein] dehydratase 3 OS=Homo sapiens GN=PTPLAD1 PE=1 SV=2	2			1		1	
Q9P283-2	SEM5B_HUMAN	119280	SEMA5B	Isoform 2 of Semaphorin-5B OS=Homo sapiens GN=SEMA5B	2			1		1	
Q9UNM6	PSD13_HUMAN	42945	PSMD13	26S proteasome non-ATPase regulatory subunit 13 OS=Homo sapiens GN=PSMD13 PE=1 SV=2	2			1		1	
P62314	SMD1_HUMAN	13282	SNRPD1	Small nuclear ribonucleoprotein Sm D1 OS=Homo sapiens GN=SNRPD1 PE=1 SV=1	2			1			1
Q14520-2	HABP2_HUMAN	59864	HABP2	Isoform 2 of Hyaluronan-binding protein 2 OS=Homo sapiens GN=HABP2	2			1			1
Q14657	LAGE3_HUMAN	14804	LAGE3	EKC/KEOPS complex subunit LAGE3 OS=Homo sapiens GN=LAGE3 PE=1 SV=2	2			1			1
O43504	LTOR5_HUMAN	9614	LAMTOR5	Ragulator complex protein LAMTOR5 OS=Homo sapiens GN=LAMTOR5 PE=1 SV=1	2				1	1	
O95777	LSM8_HUMAN	10403	LSM8	U6 snRNA-associated Sm-like protein LSm8 OS=Homo sapiens GN=LSM8 PE=1 SV=3	2				1	1	
P27361-2	MK03_HUMAN	38275	MAPK3	Isoform 2 of Mitogen-activated protein kinase 3 OS=Homo sapiens GN=MAPK3	2				1	1	
P46976-2	GLYG_HUMAN	37479	GYG1	Isoform GN-1 of Glycogenin-1 OS=Homo sapiens GN=GYG1	2				1	1	
P49441	INPP_HUMAN	43998	INPP1	Inositol polyphosphate 1-phosphatase OS=Homo sapiens GN=INPP1 PE=1 SV=1	2				1	1	
Q16853	AOC3_HUMAN	84622	AOC3	Membrane primary amine oxidase OS=Homo sapiens GN=AOC3 PE=1 SV=3	2				1	1	
Q4G0F5	VP26B_HUMAN	39155	VPS26B	Vacuolar protein sorting-associated protein 26B OS=Homo sapiens GN=VPS26B PE=1 SV=2	2				1	1	
Q53S33	BOLA3_HUMAN	12114	BOLA3	BolA-like protein 3 OS=Homo sapiens GN=BOLA3 PE=1 SV=1	2				1	1	

Q6IAA8	LTOR1_HUMAN	17745	LAMTOR1	Ragulator complex protein LAMTOR1 OS=Homo sapiens GN=LAMTOR1 PE=1 SV=2	2				1	1	
Q96C23	GALM_HUMAN	37766	GALM	Aldose 1-epimerase OS=Homo sapiens GN=GALM PE=1 SV=1	2				1	1	
Q9NZN4	EHD2_HUMAN	61161	EHD2	EH domain-containing protein 2 OS=Homo sapiens GN=EHD2 PE=1 SV=2	2				1	1	
Q9UEW8	STK39_HUMAN	59474	STK39	STE20/SPS1-related proline-alanine-rich protein kinase OS=Homo sapiens GN=STK39 PE=1 SV=3	2				1	1	
C9JIZ0	C9JIZ0_HUMAN	8109	NAA38	LSM8 homolog, U6 small nuclear RNA associated (S. cerevisiae), isoform CRA_a OS=Homo sapiens GN=NAA38 PE=4 SV=1	2				1	1	
Q12959-2	DLG1_HUMAN	103321	DLG1	Isoform 2 of Disks large homolog 1 OS=Homo sapiens GN=DLG1	2				1		1
Q4VC31	CCD58_HUMAN	16620	CCDC58	Coiled-coil domain-containing protein 58 OS=Homo sapiens GN=CCDC58 PE=1 SV=1	2				1		1
Q9Y2T4-2	2ABG_HUMAN	51376	PPP2R2C	Isoform 2 of Serine/threonine-protein phosphatase 2A 55 kDa regulatory subunit B gamma isoform OS=Homo sapiens GN=PPP2R2C	2				1		1
A6NCE7	MP3B2_HUMAN	14628	MAP1LC3B2	Microtubule-associated proteins 1A/1B light chain 3 beta 2 OS=Homo sapiens GN=MAP1LC3B2 PE=2 SV=1	2					1	1
O15116	LSM1_HUMAN	15179	LSM1	U6 snRNA-associated Sm-like protein LSm1 OS=Homo sapiens GN=LSM1 PE=1 SV=1	2					1	1
P10644	KAP0_HUMAN	42982	PRKAR1A	cAMP-dependent protein kinase type I-alpha regulatory subunit OS=Homo sapiens GN=PRKAR1A PE=1 SV=1	2					1	1
P17405-4	ASM_HUMAN	69624	SMPD1	Isoform 4 of Sphingomyelin phosphodiesterase OS=Homo sapiens GN=SMPD1	2					1	1
P27694	RFA1_HUMAN	68138	RPA1	Replication protein A 70 kDa DNA-binding subunit OS=Homo sapiens GN=RPA1 PE=1 SV=2	2					1	1
P61081	UBC12_HUMAN	20900	UBE2M	NEDD8-conjugating enzyme Ubc12 OS=Homo sapiens GN=UBE2M PE=1 SV=1	2					1	1
P62256-2	UBE2H_HUMAN	17174	UBE2H	Isoform 2 of Ubiquitin-conjugating enzyme E2 H OS=Homo sapiens GN=UBE2H	2					1	1
Q13526	PIN1_HUMAN	18243	PIN1	Peptidyl-prolyl cis-trans isomerase NIMA-interacting 1 OS=Homo sapiens GN=PIN1 PE=1 SV=1	2					1	1
Q15382	RHEB_HUMAN	20497	RHEB	GTP-binding protein Rheb OS=Homo sapiens GN=RHEB PE=1 SV=1	2					1	1
Q5JNZ5	RS26L_HUMAN	13002	RPS26P11	Putative 40S ribosomal protein S26-like 1 OS=Homo sapiens GN=RPS26P11 PE=5 SV=1	2					1	1
Q6YN16-2	HSDL2_HUMAN	37320	HSDL2	Isoform 2 of Hydroxysteroid dehydrogenase-like protein 2 OS=Homo sapiens GN=HSDL2	2					1	1
Q8NES3-2	LFNG_HUMAN	28151	LFNG	Isoform 2 of Beta-1,3-N-acetylglucosaminyltransferase lunatic fringe OS=Homo sapiens GN=LFNG	2					1	1
Q8NGI1	O56B2_HUMAN	35955	OR56B2P	Putative olfactory receptor 56B2 OS=Homo sapiens GN=OR56B2P PE=5 SV=1	2					1	1
Q8WVJ2	NUDC2_HUMAN	17676	NUDCD2	NudC domain-containing protein 2 OS=Homo sapiens GN=NUDCD2 PE=1 SV=1	2					1	1
Q8WWX9	SELM_HUMAN	16082	SELM	Selenoprotein M OS=Homo sapiens GN=SELM PE=1 SV=3	2					1	1
Q9GZQ8	MLP3B_HUMAN	14688	MAP1LC3B	Microtubule-associated proteins 1A/1B light chain 3B OS=Homo sapiens GN=MAP1LC3B PE=1 SV=3	2					1	1
Q9Y2E5-2	MA2B2_HUMAN	107704	MAN2B2	Isoform 2 of Epididymis-specific alpha-mannosidase OS=Homo sapiens GN=MAN2B2	2					1	1
Q7Z5L0	VMO1_HUMAN	21534	VMO1	Vitelline membrane outer layer protein 1 homolog OS=Homo sapiens GN=VMO1 PE=1 SV=1	1	2					
Q8N6Y2-2	LRC17_HUMAN	36273	LRRC17	Isoform 2 of Leucine-rich repeat-containing protein 17 OS=Homo sapiens GN=LRRC17	1	2					
Q9UM47	NOTC3_HUMAN	243631	NOTCH3	Neurogenic locus notch homolog protein 3 OS=Homo sapiens GN=NOTCH3 PE=1 SV=2	1	2					
Q02318	CP27A_HUMAN	60235	CYP27A1	Sterol 26-hydroxylase, mitochondrial OS=Homo sapiens GN=CYP27A1 PE=1 SV=1	1		2				
Q03167-2	TGBR3_HUMAN	93428	TGFBR3	Isoform 2 of Transforming growth factor beta receptor type 3 OS=Homo sapiens GN=TGFBR3	1		2				
Q8TE85-2	GRHL3_HUMAN	68353	GRHL3	Isoform 2 of Grainyhead-like protein 3 homolog OS=Homo sapiens GN=GRHL3	1		2				
Q96JN8-2	NEUL4_HUMAN	166722	NEURL4	Isoform 2 of Neuralized-like protein 4 OS=Homo sapiens GN=NEURL4	1		2				
Q9Y5V3	MAGD1_HUMAN	86161	MAGED1	Melanoma-associated antigen D1 OS=Homo sapiens GN=MAGED1 PE=1 SV=3	1		2				
O14529	CUX2_HUMAN	161677	CUX2	Homeobox protein cut-like 2 OS=Homo sapiens GN=CUX2 PE=1 SV=4	1			2			
O75695	XRP2_HUMAN	39641	RP2	Protein XRP2 OS=Homo sapiens GN=RP2 PE=1 SV=4	1			2			
O95445	APOM_HUMAN	21253	APOM	Apolipoprotein M OS=Homo sapiens GN=APOM PE=1 SV=2	1			2			
P01111	RASN_HUMAN	21229	NRAS	GTPase NRas OS=Homo sapiens GN=NRAS PE=1 SV=1	1			2			

P01112-2	RASH_HUMAN	18870	HRAS	Isoform 2 of GTPase HRas OS=Homo sapiens GN=HRAS	1			2			
P01116-2	RASK_HUMAN	21425	KRAS	Isoform 2B of GTPase KRas OS=Homo sapiens GN=KRAS	1			2			
P04181-2	OAT_HUMAN	32853	OAT	Isoform 2 of Ornithine aminotransferase, mitochondrial OS=Homo sapiens GN=OAT	1			2			
P09525	ANXA4_HUMAN	35883	ANXA4	Annexin A4 OS=Homo sapiens GN=ANXA4 PE=1 SV=4	1			2			
P12270	TPR_HUMAN	267293	TPR	Nucleoprotein TPR OS=Homo sapiens GN=TPR PE=1 SV=3	1			2			
P29597	TYK2_HUMAN	133650	TYK2	Non-receptor tyrosine-protein kinase TYK2 OS=Homo sapiens GN=TYK2 PE=1 SV=3	1			2			
P47895	AL1A3_HUMAN	56108	ALDH1A3	Aldehyde dehydrogenase family 1 member A3 OS=Homo sapiens GN=ALDH1A3 PE=1 SV=2	1			2			
P52272-2	HNRPM_HUMAN	73621	HNRNPM	Isoform 2 of Heterogeneous nuclear ribonucleoprotein M OS=Homo sapiens GN=HNRNPM	1			2			
Q01085-2	TIAR_HUMAN	43449	TIAL1	Isoform 2 of Nucleolysin TIAR OS=Homo sapiens GN=TIAL1	1			2			
Q15437	SC23B_HUMAN	86479	SEC23B	Protein transport protein Sec23B OS=Homo sapiens GN=SEC23B PE=1 SV=2	1			2			
Q6P179-3	ERAP2_HUMAN	105526	ERAP2	Isoform 3 of Endoplasmic reticulum aminopeptidase 2 OS=Homo sapiens GN=ERAP2	1			2			
Q99623-2	PHB2_HUMAN	29044	PHB2	Isoform 2 of Prohibitin-2 OS=Homo sapiens GN=PHB2	1			2			
Q9NVJ2	ARL8B_HUMAN	21539	ARL8B	ADP-ribosylation factor-like protein 8B OS=Homo sapiens GN=ARL8B PE=1 SV=1	1			2			
B9A064	IGLL5_HUMAN	23063	IGLL5	Immunoglobulin lambda-like polypeptide 5 OS=Homo sapiens GN=IGLL5 PE=2 SV=2	1				2		
P02675	FIBB_HUMAN	55928	FGB	Fibrinogen beta chain OS=Homo sapiens GN=FGB PE=1 SV=2	1				2		
P05546	HEP2_HUMAN	57071	SERPIND1	Heparin cofactor 2 OS=Homo sapiens GN=SERPIND1 PE=1 SV=3	1				2		
P16989-2	YBOX3_HUMAN	31947	YBX3	Isoform 2 of Y-box-binding protein 3 OS=Homo sapiens GN=YBX3	1				2		
P17980	PRS6A_HUMAN	49204	PSMC3	26S protease regulatory subunit 6A OS=Homo sapiens GN=PSMC3 PE=1 SV=3	1				2		
P27635	RL10_HUMAN	24604	RPL10	60S ribosomal protein L10 OS=Homo sapiens GN=RPL10 PE=1 SV=4	1				2		
P55209-2	NP1L1_HUMAN	42762	NAP1L1	Isoform 2 of Nucleosome assembly protein 1-like 1 OS=Homo sapiens GN=NAP1L1	1				2		
P62424	RL7A_HUMAN	29996	RPL7A	60S ribosomal protein L7a OS=Homo sapiens GN=RPL7A PE=1 SV=2	1				2		
Q14031-2	CO4A6_HUMAN	163630	COL4A6	Isoform B of Collagen alpha-6(IV) chain OS=Homo sapiens GN=COL4A6	1				2		
Q2UY09	COSA1_HUMAN	116657	COL28A1	Collagen alpha-1(XXVIII) chain OS=Homo sapiens GN=COL28A1 PE=2 SV=2	1				2		
Q6UX04-2	CWC27_HUMAN	43989	CWC27	Isoform 2 of Peptidyl-prolyl cis-trans isomerase CWC27 homolog OS=Homo sapiens GN=CWC27	1				2		
Q7LG56	RIR2B_HUMAN	40737	RRM2B	Ribonucleoside-diphosphate reductase subunit M2 B OS=Homo sapiens GN=RRM2B PE=1 SV=1	1				2		
Q86UE4	LYRIC_HUMAN	63837	MTDH	Protein LYRIC OS=Homo sapiens GN=MTDH PE=1 SV=2	1				2		
Q8TB61-2	S35B2_HUMAN	42967	SLC35B2	Isoform 2 of Adenosine 3'-phospho 5'-phosphosulfate transporter 1 OS=Homo sapiens GN=SLC35B2	1				2		
Q8WW59	SPRY4_HUMAN	23129	SPRYD4	SPRY domain-containing protein 4 OS=Homo sapiens GN=SPRYD4 PE=1 SV=2	1				2		
Q8WZ42-12	TITIN_HUMAN	3994625	TTN	Isoform 12 of Titin OS=Homo sapiens GN=TTN	1				2		
Q96IZ0	PAWR_HUMAN	36568	PAWR	PRKC apoptosis WT1 regulator protein OS=Homo sapiens GN=PAWR PE=1 SV=1	1				2		
Q9NR30	DDX21_HUMAN	87344	DDX21	Nucleolar RNA helicase 2 OS=Homo sapiens GN=DDX21 PE=1 SV=5	1				2		
Q9Y265-2	RUVB1_HUMAN	42127	RUVBL1	Isoform 2 of RuvB-like 1 OS=Homo sapiens GN=RUVBL1	1				2		
Q9Y2T7	YBOX2_HUMAN	38518	YBX2	Y-box-binding protein 2 OS=Homo sapiens GN=YBX2 PE=1 SV=2	1				2		
O15498	YKT6_HUMAN	22418	YKT6	Synaptobrevin homolog YKT6 OS=Homo sapiens GN=YKT6 PE=1 SV=1	1					2	
O75884-2	RBBP9_HUMAN	19394	RBBP9	Isoform 2 of Putative hydrolase RBBP9 OS=Homo sapiens GN=RBBP9	1					2	
P08571	CD14_HUMAN	40076	CD14	Monocyte differentiation antigen CD14 OS=Homo sapiens GN=CD14 PE=1 SV=2	1					2	
P27487	DPP4_HUMAN	88279	DPP4	Dipeptidyl peptidase 4 OS=Homo sapiens GN=DPP4 PE=1 SV=2	1					2	
P32455	GBP1_HUMAN	67931	GBP1	Interferon-induced guanylate-binding protein 1 OS=Homo sapiens GN=GBP1 PE=1 SV=2	1					2	
P36405	ARL3_HUMAN	20456	ARL3	ADP-ribosylation factor-like protein 3 OS=Homo sapiens GN=ARL3 PE=1 SV=2	1					2	
P50914	RL14_HUMAN	23432	RPL14	60S ribosomal protein L14 OS=Homo sapiens GN=RPL14 PE=1 SV=4	1					2	
P51991-2	ROA3_HUMAN	37029	HNRNPA3	Isoform 2 of Heterogeneous nuclear ribonucleoprotein A3 OS=Homo sapiens GN=HNRNPA3	1					2	
P78536-2	ADA17_HUMAN	78543	ADAM17	Isoform B of Disintegrin and metalloproteinase domain-containing protein 17 OS=Homo sapiens GN=ADAM17	1					2	

Q13469-2	NFAC2_HUMAN	99784	NFATC2	Isoform 2 of Nuclear factor of activated T-cells, cytoplasmic 2 OS=Homo sapiens GN=NFATC2	1					2	
Q5EBL8-2	PDZ11_HUMAN	19492	PDZD11	Isoform 2 of PDZ domain-containing protein 11 OS=Homo sapiens GN=PDZD11	1					2	
Q5T1M5-2	FKB15_HUMAN	132467	FKBP15	Isoform 2 of FK506-binding protein 15 OS=Homo sapiens GN=FKBP15	1					2	
Q96QI5	HS3S6_HUMAN	37186	HS3ST6	Heparan sulfate glucosamine 3-O-sulfotransferase 6 OS=Homo sapiens GN=HS3ST6 PE=1 SV=2	1					2	
Q99985	SEM3C_HUMAN	85207	SEMA3C	Semaphorin-3C OS=Homo sapiens GN=SEMA3C PE=1 SV=2	1					2	
Q9UBX1	CATF_HUMAN	53366	CTSF	Cathepsin F OS=Homo sapiens GN=CTSF PE=1 SV=1	1					2	
Q9ULC4-2	MCTS1_HUMAN	19229	MCTS1	Isoform 2 of Malignant T-cell-amplified sequence 1 OS=Homo sapiens GN=MCTS1	1					2	
Q9UNA0	ATS5_HUMAN	101718	ADAMTS5	A disintegrin and metalloproteinase with thrombospondin motifs 5 OS=Homo sapiens GN=ADAMTS5 PE=1 SV=2	1					2	
Q9Y2Y0-2	AR2BP_HUMAN	17711	ARL2BP	Isoform 2 of ADP-ribosylation factor-like protein 2-binding protein OS=Homo sapiens GN=ARL2BP	1					2	
O00461	GOLI4_HUMAN	81880	GOLIM4	Golgi integral membrane protein 4 OS=Homo sapiens GN=GOLIM4 PE=1 SV=1	1						2
P35221-2	CTNA1_HUMAN	102635	CTNNA1	Isoform 2 of Catenin alpha-1 OS=Homo sapiens GN=CTNNA1	1						2
P38571-2	LICH_HUMAN	39112	LIPA	Isoform 2 of Lysosomal acid lipase/cholesteryl ester hydrolase OS=Homo sapiens GN=LIPA	1						2
P48723	HSP13_HUMAN	51927	HSPA13	Heat shock 70 kDa protein 13 OS=Homo sapiens GN=HSPA13 PE=1 SV=1	1						2
P80098	CCL7_HUMAN	11200	CCL7	C-C motif chemokine 7 OS=Homo sapiens GN=CCL7 PE=1 SV=3	1						2
Q14008-2	CKAP5_HUMAN	218525	CKAP5	Isoform 2 of Cytoskeleton-associated protein 5 OS=Homo sapiens GN=CKAP5	1						2
Q15751	HERC1_HUMAN	532228	HERC1	Probable E3 ubiquitin-protein ligase HERC1 OS=Homo sapiens GN=HERC1 PE=1 SV=2	1						2
Q8NI99	ANGL6_HUMAN	51694	ANGPTL6	Angiopoietin-related protein 6 OS=Homo sapiens GN=ANGPTL6 PE=1 SV=1	1						2
Q92499	DDX1_HUMAN	82432	DDX1	ATP-dependent RNA helicase DDX1 OS=Homo sapiens GN=DDX1 PE=1 SV=2	1						2
Q9H0X4	ITFG3_HUMAN	59660	ITFG3	Protein ITFG3 OS=Homo sapiens GN=ITFG3 PE=1 SV=1	1						2
Q9P2K8-2	E2AK4_HUMAN	183717	EIF2AK4	Isoform 2 of Eukaryotic translation initiation factor 2-alpha kinase 4 OS=Homo sapiens GN=EIF2AK4	1						2
Q9UMX0-2	UBQL1_HUMAN	59220	UBQLN1	Isoform 2 of Ubiquilin-1 OS=Homo sapiens GN=UBQLN1	1						2
K7EJF4	K7EJF4_HUMAN	1553	ZNF579	Zinc finger protein 579 (Fragment) OS=Homo sapiens GN=ZNF579 PE=4 SV=1	1						2
Q7Z7Q8	Q7Z7Q8_HUMAN	12357	MCP-3	C-C motif chemokine 7 OS=Homo sapiens GN=MCP-3 PE=2 SV=1	1						2
A8MTJ3	GNAT3_HUMAN	40357	GNAT3	Guanine nucleotide-binding protein G(t) subunit alpha-3 OS=Homo sapiens GN=GNAT3 PE=2 SV=2	1	1					
O15173-2	PGRC2_HUMAN	26170	PGRMC2	Isoform 2 of Membrane-associated progesterone receptor component 2 OS=Homo sapiens GN=PGRMC2	1	1					
O60636-2	TSN2_HUMAN	21269	TSPAN2	Isoform 2 of Tetraspanin-2 OS=Homo sapiens GN=TSPAN2	1	1					
P11488	GNAT1_HUMAN	40041	GNAT1	Guanine nucleotide-binding protein G(t) subunit alpha-1 OS=Homo sapiens GN=GNAT1 PE=1 SV=5	1	1					
P13521	SCG2_HUMAN	70941	SCG2	Secretogranin-2 OS=Homo sapiens GN=SCG2 PE=1 SV=2	1	1					
P13747	HLAE_HUMAN	40157	HLA-E	HLA class I histocompatibility antigen, alpha chain E OS=Homo sapiens GN=HLA-E PE=1 SV=3	1	1					
P17693	HLAG_HUMAN	38224	HLA-G	HLA class I histocompatibility antigen, alpha chain G OS=Homo sapiens GN=HLA-G PE=1 SV=1	1	1					
P19087	GNAT2_HUMAN	40176	GNAT2	Guanine nucleotide-binding protein G(t) subunit alpha-2 OS=Homo sapiens GN=GNAT2 PE=1 SV=4	1	1					
P38405-3	GNAL_HUMAN	20522	GNAL	Isoform 3 of Guanine nucleotide-binding protein G(olf) subunit alpha OS=Homo sapiens GN=GNAL	1	1					
P63098	CANB1_HUMAN	19300	PPP3R1	Calcineurin subunit B type 1 OS=Homo sapiens GN=PPP3R1 PE=1 SV=2	1	1					
Q18PE1-2	DOK7_HUMAN	37161	DOK7	Isoform 2 of Protein Dok-7 OS=Homo sapiens GN=DOK7	1	1					
Q7Z408	CSMD2_HUMAN	380039	CSMD2	CUB and sushi domain-containing protein 2 OS=Homo sapiens GN=CSMD2 PE=1 SV=2	1	1					
Q86YB8	ERO1B_HUMAN	53543	ERO1LB	ERO1-like protein beta OS=Homo sapiens GN=ERO1LB PE=1 SV=2	1	1					
Q8IUX1-2	T126B_HUMAN	25028	TMEM126B	Isoform 2 of Complex I assembly factor TMEM126B, mitochondrial OS=Homo	1	1					

				sapiens GN=TMEM126B							
Q8TDC3-2	BRSK1_HUMAN	86753	BRSK1	Isoform 2 of Serine/threonine-protein kinase BRSK1 OS=Homo sapiens GN=BRSK1	1	1					
Q96QR8	PURB_HUMAN	33241	PURB	Transcriptional activator protein Pur-beta OS=Homo sapiens GN=PURB PE=1 SV=3	1	1					
Q99627-2	CSN8_HUMAN	17909	COPS8	Isoform 2 of COP9 signalosome complex subunit 8 OS=Homo sapiens GN=COPS8	1	1					
Q9BRG2	SH23A_HUMAN	63093	SH2D3A	SH2 domain-containing protein 3A OS=Homo sapiens GN=SH2D3A PE=1 SV=1	1	1					
Q9GZM7-3	TINAL_HUMAN	48807	TINAGL1	Isoform 3 of Tubulointerstitial nephritis antigen-like OS=Homo sapiens GN=TINAGL1	1	1					
Q9HCH0-2	NCK5L_HUMAN	134913	NCKAP5L	Isoform 2 of Nck-associated protein 5-like OS=Homo sapiens GN=NCKAP5L	1	1					
Q9NQW7-2	XPP1_HUMAN	67227	XPNPEP1	Isoform 2 of Xaa-Pro aminopeptidase 1 OS=Homo sapiens GN=XPNPEP1	1	1					
Q9NR45	SIAS_HUMAN	40308	NANS	Sialic acid synthase OS=Homo sapiens GN=NANS PE=1 SV=2	1	1					
Q9UHN6-2	TMEM2_HUMAN	147439	TMEM2	Isoform 2 of Transmembrane protein 2 OS=Homo sapiens GN=TMEM2	1	1					
Q31611	Q31611_HUMAN	27680	HLA-G2.2	B2 microglobulin OS=Homo sapiens GN=HLA-G2.2 PE=2 SV=1	1	1					
O00193	SMAP_HUMAN	20333	SMAP	Small acidic protein OS=Homo sapiens GN=SMAP PE=1 SV=1	1		1				
O75095-2	MEGF6_HUMAN	128614	MEGF6	Isoform 2 of Multiple epidermal growth factor-like domains protein 6 OS=Homo sapiens GN=MEGF6	1		1				
P05976	MYL1_HUMAN	21145	MYL1	Myosin light chain 1/3, skeletal muscle isoform OS=Homo sapiens GN=MYL1 PE=1 SV=3	1		1				
P10301	RRAS_HUMAN	23480	RRAS	Ras-related protein R-Ras OS=Homo sapiens GN=RRAS PE=1 SV=1	1		1				
P47897	SYQ_HUMAN	87799	QARS	Glutamine--tRNA ligase OS=Homo sapiens GN=QARS PE=1 SV=1	1		1				
P51688	SPHM_HUMAN	56695	SGSH	N-sulphoglucosamine sulphohydrolase OS=Homo sapiens GN=SGSH PE=1 SV=1	1		1				
P54764	EPHA4_HUMAN	109860	EPHA4	Ephrin type-A receptor 4 OS=Homo sapiens GN=EPHA4 PE=1 SV=1	1		1				
Q07065	CKAP4_HUMAN	66022	CKAP4	Cytoskeleton-associated protein 4 OS=Homo sapiens GN=CKAP4 PE=1 SV=2	1		1				
Q13444-10	ADA15_HUMAN	83664	ADAM15	Isoform 10 of Disintegrin and metalloproteinase domain-containing protein 15 OS=Homo sapiens GN=ADAM15	1		1				
Q14549	GBX1_HUMAN	37629	GBX1	Homeobox protein GBX-1 OS=Homo sapiens GN=GBX1 PE=1 SV=2	1		1				
Q15828	CYTM_HUMAN	16511	CST6	Cystatin-M OS=Homo sapiens GN=CST6 PE=1 SV=1	1		1				
Q5VV67	PPRC1_HUMAN	177544	PPRC1	Peroxisome proliferator-activated receptor gamma coactivator-related protein 1 OS=Homo sapiens GN=PPRC1 PE=1 SV=1	1		1				
Q68BL7-2	OLM2A_HUMAN	68890	OLFML2A	Isoform 2 of Olfactomedin-like protein 2A OS=Homo sapiens GN=OLFML2A	1		1				
Q6PCE3	PGM2L_HUMAN	70442	PGM2L1	Glucose 1,6-bisphosphate synthase OS=Homo sapiens GN=PGM2L1 PE=1 SV=3	1		1				
Q8IZC6-2	CORA1_HUMAN	82801	COL27A1	Isoform 2 of Collagen alpha-1(XXVII) chain OS=Homo sapiens GN=COL27A1	1		1				
Q96RF0-2	SNX18_HUMAN	69006	SNX18	Isoform 2 of Sorting nexin-18 OS=Homo sapiens GN=SNX18	1		1				
Q9BXS1	IDI2_HUMAN	26753	IDI2	Isopentenyl-diphosphate delta-isomerase 2 OS=Homo sapiens GN=IDI2 PE=1 SV=1	1		1				
Q9NU22	MDN1_HUMAN	632820	MDN1	Midasin OS=Homo sapiens GN=MDN1 PE=1 SV=2	1		1				
Q9UJQ4-2	SALL4_HUMAN	65708	SALL4	Isoform SALL4B of Sal-like protein 4 OS=Homo sapiens GN=SALL4	1		1				
E3W975	E3W975_HUMAN	15456	C11orf58	Small acidic protein OS=Homo sapiens GN=C11orf58 PE=2 SV=1	1		1				
A2VDJ0-2	T131L_HUMAN	138688	KIAA0922	Isoform 2 of Transmembrane protein 131-like OS=Homo sapiens GN=KIAA0922	1			1			
O00487	PSDE_HUMAN	34577	PSMD14	26S proteasome non-ATPase regulatory subunit 14 OS=Homo sapiens GN=PSMD14 PE=1 SV=1	1			1			
O00625	PIR_HUMAN	32113	PIR	Pirin OS=Homo sapiens GN=PIR PE=1 SV=1	1			1			
O00743-3	PPP6_HUMAN	38947	PPP6C	Isoform 3 of Serine/threonine-protein phosphatase 6 catalytic subunit OS=Homo sapiens GN=PPP6C	1			1			
O14933-2	UB2L6_HUMAN	10086	UBE2L6	Isoform 2 of Ubiquitin/ISG15-conjugating enzyme E2 L6 OS=Homo sapiens GN=UBE2L6	1			1			
O15054-1	KDM6B_HUMAN	180702	KDM6B	Isoform 1 of Lysine-specific demethylase 6B OS=Homo sapiens GN=KDM6B	1			1			
O15260-2	SURF4_HUMAN	17970	SURF4	Isoform 2 of Surfeit locus protein 4 OS=Homo sapiens GN=SURF4	1			1			
O43379-4	WDR62_HUMAN	166511	WDR62	Isoform 4 of WD repeat-containing protein 62 OS=Homo sapiens GN=WDR62	1			1			
O75037-2	KI21B_HUMAN	181270	KIF21B	Isoform 2 of Kinesin-like protein KIF21B OS=Homo sapiens GN=KIF21B	1			1			
O75954	TSN9_HUMAN	26779	TSPAN9	Tetraspanin-9 OS=Homo sapiens GN=TSPAN9 PE=1 SV=1	1			1			
O95716	RAB3D_HUMAN	24267	RAB3D	Ras-related protein Rab-3D OS=Homo sapiens GN=RAB3D PE=1 SV=1	1			1			

P00739-2	HPTR_HUMAN	43078	HPR	Isoform 2 of Haptoglobin-related protein OS=Homo sapiens GN=HPR	1			1			
P00918	CAH2_HUMAN	29246	CA2	Carbonic anhydrase 2 OS=Homo sapiens GN=CA2 PE=1 SV=2	1			1			
P03951-2	FA11_HUMAN	63840	F11	Isoform 2 of Coagulation factor XI OS=Homo sapiens GN=F11	1			1			
P06737-2	PYGL_HUMAN	93134	PYGL	Isoform 2 of Glycogen phosphorylase, liver form OS=Homo sapiens GN=PYGL	1			1			
P10155-3	RO60_HUMAN	58483	TROVE2	Isoform 3 of 60 kDa SS-A/Ro ribonucleoprotein OS=Homo sapiens GN=TROVE2	1			1			
P11217-2	PYGM_HUMAN	87317	PYGM	Isoform 2 of Glycogen phosphorylase, muscle form OS=Homo sapiens GN=PYGM	1			1			
P15169	CBPN_HUMAN	52286	CPN1	Carboxypeptidase N catalytic chain OS=Homo sapiens GN=CPN1 PE=1 SV=1	1			1			
P16401	H15_HUMAN	22580	HIST1H1B	Histone H1.5 OS=Homo sapiens GN=HIST1H1B PE=1 SV=3	1			1			
P17677-2	NEUM_HUMAN	28766	GAP43	Isoform 2 of Neuromodulin OS=Homo sapiens GN=GAP43	1			1			
P20336	RAB3A_HUMAN	24984	RAB3A	Ras-related protein Rab-3A OS=Homo sapiens GN=RAB3A PE=1 SV=1	1			1			
P20337	RAB3B_HUMAN	24758	RAB3B	Ras-related protein Rab-3B OS=Homo sapiens GN=RAB3B PE=1 SV=2	1			1			
P20338	RAB4A_HUMAN	24390	RAB4A	Ras-related protein Rab-4A OS=Homo sapiens GN=RAB4A PE=1 SV=3	1			1			
P23634-6	AT2B4_HUMAN	133931	ATP2B4	Isoform XB of Plasma membrane calcium-transporting ATPase 4 OS=Homo sapiens GN=ATP2B4	1			1			
P30533	AMRP_HUMAN	41466	LRPAP1	Alpha-2-macroglobulin receptor-associated protein OS=Homo sapiens GN=LRPAP1 PE=1 SV=1	1			1			
P30566-2	PUR8_HUMAN	48328	ADSL	Isoform 2 of Adenylosuccinate lyase OS=Homo sapiens GN=ADSL	1			1			
P30711	GSTT1_HUMAN	27335	GSTT1	Glutathione S-transferase theta-1 OS=Homo sapiens GN=GSTT1 PE=1 SV=4	1			1			
P31483-2	TIA1_HUMAN	41801	TIA1	Isoform Short of Nucleolysin TIA-1 isoform p40 OS=Homo sapiens GN=TIA1	1			1			
P61964	WDR5_HUMAN	36588	WDR5	WD repeat-containing protein 5 OS=Homo sapiens GN=WDR5 PE=1 SV=1	1			1			
P62277	RS13_HUMAN	17222	RPS13	40S ribosomal protein S13 OS=Homo sapiens GN=RPS13 PE=1 SV=2	1			1			
P67870	CSK2B_HUMAN	24942	CSNK2B	Casein kinase II subunit beta OS=Homo sapiens GN=CSNK2B PE=1 SV=1	1			1			
P80217-2	IN35_HUMAN	31777	IFI35	Isoform 2 of Interferon-induced 35 kDa protein OS=Homo sapiens GN=IFI35	1			1			
Q00536-3	CDK16_HUMAN	56375	CDK16	Isoform 3 of Cyclin-dependent kinase 16 OS=Homo sapiens GN=CDK16	1			1			
Q01433	AMPD2_HUMAN	100688	AMPD2	AMP deaminase 2 OS=Homo sapiens GN=AMPD2 PE=1 SV=2	1			1			
Q03692	COAA1_HUMAN	66158	COL10A1	Collagen alpha-1(X) chain OS=Homo sapiens GN=COL10A1 PE=1 SV=2	1			1			
Q07507	DERM_HUMAN	24005	DPT	Dermatopontin OS=Homo sapiens GN=DPT PE=2 SV=2	1			1			
Q07666-2	KHDR1_HUMAN	45861	KHDRBS1	Isoform 2 of KH domain-containing, RNA-binding, signal transduction-associated protein 1 OS=Homo sapiens GN=KHDRBS1	1			1			
Q0VDG4-2	SCRN3_HUMAN	47670	SCRN3	Isoform 2 of Secernin-3 OS=Homo sapiens GN=SCRN3	1			1			
Q12791-2	KCMA1_HUMAN	135495	KCNMA1	Isoform 2 of Calcium-activated potassium channel subunit alpha-1 OS=Homo sapiens GN=KCNMA1	1			1			
Q13148	TADBP_HUMAN	44740	TARDBP	TAR DNA-binding protein 43 OS=Homo sapiens GN=TARDBP PE=1 SV=1	1			1			
Q13868	EXOS2_HUMAN	32789	EXOSC2	Exosome complex component RRP4 OS=Homo sapiens GN=EXOSC2 PE=1 SV=2	1			1			
Q14524-2	SCN5A_HUMAN	226812	SCN5A	Isoform 2 of Sodium channel protein type 5 subunit alpha OS=Homo sapiens GN=SCN5A	1			1			
Q15459	SF3A1_HUMAN	88886	SF3A1	Splicing factor 3A subunit 1 OS=Homo sapiens GN=SF3A1 PE=1 SV=1	1			1			
Q15771	RAB30_HUMAN	23058	RAB30	Ras-related protein Rab-30 OS=Homo sapiens GN=RAB30 PE=1 SV=2	1			1			
Q5T036	F120S_HUMAN	27929	FAM120AOS	Putative FAM120A opposite strand protein OS=Homo sapiens GN=FAM120AOS PE=5 SV=1	1			1			
Q5TF21	SOGA3_HUMAN	103199	SOGA3	Protein SOGA3 OS=Homo sapiens GN=SOGA3 PE=2 SV=1	1			1			
Q68CZ2-2	TENS3_HUMAN	129066	TNS3	Isoform 2 of Tensin-3 OS=Homo sapiens GN=TNS3	1			1			
Q6IQ22	RAB12_HUMAN	27248	RAB12	Ras-related protein Rab-12 OS=Homo sapiens GN=RAB12 PE=1 SV=3	1			1			
Q6QEF8-2	CORO6_HUMAN	31789	CORO6	Isoform 2 of Coronin-6 OS=Homo sapiens GN=CORO6	1			1			
Q6YP21-2	KAT3_HUMAN	18352	CCBL2	Isoform 2 of Kynurenine--oxoglutarate transaminase 3 OS=Homo sapiens GN=CCBL2	1			1			
Q76LX8-2	ATS13_HUMAN	147804	ADAMTS13	Isoform 2 of A disintegrin and metalloproteinase with thrombospondin motifs 13 OS=Homo sapiens GN=ADAMTS13	1			1			
Q7L4E1-2	FA73B_HUMAN	34083	FAM73B	Isoform 2 of Protein FAM73B OS=Homo sapiens GN=FAM73B	1			1			
Q7Z6B7-2	SRGP1_HUMAN	121594	SRGAP1	Isoform 2 of SLIT-ROBO Rho GTPase-activating protein 1 OS=Homo sapiens GN=SRGAP1	1			1			
Q7Z739	YTHD3_HUMAN	63861	YTHDF3	YTH domain-containing family protein 3 OS=Homo sapiens GN=YTHDF3 PE=1 SV=1	1			1			

Q86YS6	RAB43_HUMAN	23339	RAB43	Ras-related protein Rab-43 OS=Homo sapiens GN=RAB43 PE=1 SV=1	1			1			
Q8IWT0-2	ARCH_HUMAN	15709	ZBTB8OS	Isoform 2 of Protein archease OS=Homo sapiens GN=ZBTB8OS	1			1			
Q8N2F6-2	ARM10_HUMAN	33850	ARMC10	Isoform 2 of Armadillo repeat-containing protein 10 OS=Homo sapiens GN=ARMC10	1			1			
Q8NB37-2	PDDC1_HUMAN	26784	PDDC1	Isoform 2 of Parkinson disease 7 domain-containing protein 1 OS=Homo sapiens GN=PDDC1	1			1			
Q8NBI6-2	XXLT1_HUMAN	22304	XXYLT1	Isoform 2 of Xyloside xylosyltransferase 1 OS=Homo sapiens GN=XXYLT1	1			1			
Q8NFV4-4	ABHDB_HUMAN	33863	ABHD11	Isoform 4 of Alpha/beta hydrolase domain-containing protein 11 OS=Homo sapiens GN=ABHD11	1			1			
Q8NI27-2	THOC2_HUMAN	46812	THOC2	Isoform 2 of THO complex subunit 2 OS=Homo sapiens GN=THOC2	1			1			
Q8WXX0	DYH7_HUMAN	461159	DNAH7	Dynein heavy chain 7, axonemal OS=Homo sapiens GN=DNAH7 PE=1 SV=2	1			1			
Q92673	SORL_HUMAN	248426	SORL1	Sortilin-related receptor OS=Homo sapiens GN=SORL1 PE=1 SV=2	1			1			
Q93097	WNT2B_HUMAN	43770	WNT2B	Protein Wnt-2b OS=Homo sapiens GN=WNT2B PE=1 SV=2	1			1			
Q969G5	PRDBP_HUMAN	27701	PRKCDBP	Protein kinase C delta-binding protein OS=Homo sapiens GN=PRKCDBP PE=1 SV=3	1			1			
Q96AB6	NTAN1_HUMAN	34677	NTAN1	Protein N-terminal asparagine amidohydrolase OS=Homo sapiens GN=NTAN1 PE=1 SV=3	1			1			
Q96AX2-2	RAB37_HUMAN	24169	RAB37	Isoform 2 of Ras-related protein Rab-37 OS=Homo sapiens GN=RAB37	1			1			
Q96BM9	ARL8A_HUMAN	21416	ARL8A	ADP-ribosylation factor-like protein 8A OS=Homo sapiens GN=ARL8A PE=1 SV=1	1			1			
Q96DA2	RB39B_HUMAN	24622	RAB39B	Ras-related protein Rab-39B OS=Homo sapiens GN=RAB39B PE=1 SV=1	1			1			
Q96DZ7-2	T4S19_HUMAN	19617	TM4SF19	Isoform 2 of Transmembrane 4 L6 family member 19 OS=Homo sapiens GN=TM4SF19	1			1			
Q96E17	RAB3C_HUMAN	25952	RAB3C	Ras-related protein Rab-3C OS=Homo sapiens GN=RAB3C PE=2 SV=1	1			1			
Q96I99	SUCB2_HUMAN	46511	SUCLG2	Succinyl-CoA ligase [GDP-forming] subunit beta, mitochondrial OS=Homo sapiens GN=SUCLG2 PE=1 SV=2	1			1			
Q96MT7-2	WDR52_HUMAN	213865	WDR52	Isoform 2 of WD repeat-containing protein 52 OS=Homo sapiens GN=WDR52	1			1			
Q96SI9-2	STRBP_HUMAN	71967	STRBP	Isoform 2 of Spermatid perinuclear RNA-binding protein OS=Homo sapiens GN=STRBP	1			1			
Q9BQA1	MEP50_HUMAN	36724	WDR77	Methylosome protein 50 OS=Homo sapiens GN=WDR77 PE=1 SV=1	1			1			
Q9BQL6-2	FERM1_HUMAN	63235	FERMT1	Isoform 2 of Fermitin family homolog 1 OS=Homo sapiens GN=FERMT1	1			1			
Q9BTT0-2	AN32E_HUMAN	26321	ANP32E	Isoform 2 of Acidic leucine-rich nuclear phosphoprotein 32 family member E OS=Homo sapiens GN=ANP32E	1			1			
Q9BUH6	CI142_HUMAN	21640	C9orf142	Uncharacterized protein C9orf142 OS=Homo sapiens GN=C9orf142 PE=1 SV=2	1			1			
Q9BYJ9	YTHD1_HUMAN	60874	YTHDF1	YTH domain-containing family protein 1 OS=Homo sapiens GN=YTHDF1 PE=1 SV=1	1			1			
Q9GZL7	WDR12_HUMAN	47708	WDR12	Ribosome biogenesis protein WDR12 OS=Homo sapiens GN=WDR12 PE=1 SV=2	1			1			
Q9H2P0	ADNP_HUMAN	123563	ADNP	Activity-dependent neuroprotector homeobox protein OS=Homo sapiens GN=ADNP PE=1 SV=1	1			1			
Q9NRG1-2	PRDC1_HUMAN	21558	PRTFDC1	Isoform 2 of Phosphoribosyltransferase domain-containing protein 1 OS=Homo sapiens GN=PRTFDC1	1			1			
Q9NWV8-3	BABA1_HUMAN	18621	BABAM1	Isoform 3 of BRISC and BRCA1-A complex member 1 OS=Homo sapiens GN=BABAM1	1			1			
Q9NXG2	THUM1_HUMAN	39315	THUMPD1	THUMP domain-containing protein 1 OS=Homo sapiens GN=THUMPD1 PE=1 SV=2	1			1			
Q9P2F6-2	RHG20_HUMAN	130235	ARHGAP20	Isoform 2 of Rho GTPase-activating protein 20 OS=Homo sapiens GN=ARHGAP20	1			1			
Q9UNS2-2	CSN3_HUMAN	45727	COPS3	Isoform 2 of COP9 signalosome complex subunit 3 OS=Homo sapiens GN=COPS3	1			1			
Q9Y2S7	PDIP2_HUMAN	42033	POLDIP2	Polymerase delta-interacting protein 2 OS=Homo sapiens GN=POLDIP2 PE=1 SV=1	1			1			
Q9Y3A3-2	PHOCN_HUMAN	22318	MOB4	Isoform 2 of MOB-like protein phocein OS=Homo sapiens GN=MOB4	1			1			
Q9Y6V0-2	PCLO_HUMAN	530858	PCLO	Isoform 2 of Protein piccolo OS=Homo sapiens GN=PCLO	1			1			
E9PAP0	E9PAP0_HUMAN	31628	LCK	Tyrosine-protein kinase Lck (Fragment) OS=Homo sapiens GN=LCK PE=2 SV=1	1			1			
Q5SRQ3	Q5SRQ3_HUMAN	26673	CSNK2B-LY6G5B-1181	Chimera CSNK2B-LY6G5B splicing isoform 1181 OS=Homo sapiens GN=CSNK2B-LY6G5B-1181 PE=2 SV=1	1			1			
Q6MZH2	Q6MZH2_HUMAN	18994	DKFZp686J07132	Putative uncharacterized protein DKFZp686J07132 OS=Homo sapiens GN=DKFZp686J07132 PE=2 SV=1	1			1			
A0M8Q6	LAC7_HUMAN	11303	IGLC7	Ig lambda-7 chain C region OS=Homo sapiens GN=IGLC7 PE=1 SV=2	1				1		

O00214-2	LEG8_HUMAN	40397	LGALS8	Isoform 2 of Galectin-8 OS=Homo sapiens GN=LGALS8	1				1		
O43592	XPOT_HUMAN	109964	XPOT	Exportin-T OS=Homo sapiens GN=XPOT PE=1 SV=2	1				1		
O60547-2	GMDS_HUMAN	39054	GMDS	Isoform 2 of GDP-mannose 4,6 dehydratase OS=Homo sapiens GN=GMDS	1				1		
O60551	NMT2_HUMAN	56980	NMT2	Glycylpeptide N-tetradecanoyltransferase 2 OS=Homo sapiens GN=NMT2 PE=1 SV=1	1				1		
O60641-3	AP180_HUMAN	63128	SNAP91	Isoform 3 of Clathrin coat assembly protein AP180 OS=Homo sapiens GN=SNAP91	1				1		
O75106-2	AOC2_HUMAN	80516	AOC2	Isoform 2 of Retina-specific copper amine oxidase OS=Homo sapiens GN=AOC2	1				1		
O75915	PRAF3_HUMAN	21615	ARL6IP5	PRA1 family protein 3 OS=Homo sapiens GN=ARL6IP5 PE=1 SV=1	1				1		
P00403	COX2_HUMAN	25565	MT-CO2	Cytochrome c oxidase subunit 2 OS=Homo sapiens GN=MT-CO2 PE=1 SV=1	1				1		
P00746	CFAD_HUMAN	27033	CFD	Complement factor D OS=Homo sapiens GN=CFD PE=1 SV=5	1				1		
P02671-2	FIBA_HUMAN	69757	FGA	Isoform 2 of Fibrinogen alpha chain OS=Homo sapiens GN=FGA	1				1		
P06493-2	CDK1_HUMAN	27503	CDK1	Isoform 2 of Cyclin-dependent kinase 1 OS=Homo sapiens GN=CDK1	1				1		
P07451	CAH3_HUMAN	29557	CA3	Carbonic anhydrase 3 OS=Homo sapiens GN=CA3 PE=1 SV=3	1				1		
P08240-2	SRPR_HUMAN	66559	SRPR	Isoform 2 of Signal recognition particle receptor subunit alpha OS=Homo sapiens GN=SRPR	1				1		
P08263	GSTA1_HUMAN	25631	GSTA1	Glutathione S-transferase A1 OS=Homo sapiens GN=GSTA1 PE=1 SV=3	1				1		
P09210	GSTA2_HUMAN	25664	GSTA2	Glutathione S-transferase A2 OS=Homo sapiens GN=GSTA2 PE=1 SV=4	1				1		
P09471-2	GNAO_HUMAN	40087	GNAO1	Isoform Alpha-2 of Guanine nucleotide-binding protein G(o) subunit alpha OS=Homo sapiens GN=GNAO1	1				1		
P0CF74	LAC6_HUMAN	11277	IGLC6	Ig lambda-6 chain C region OS=Homo sapiens GN=IGLC6 PE=4 SV=1	1				1		
P0CG04	LAC1_HUMAN	11348	IGLC1	Ig lambda-1 chain C regions OS=Homo sapiens GN=IGLC1 PE=1 SV=1	1				1		
P11277-2	SPTB1_HUMAN	267826	SPTB	Isoform 2 of Spectrin beta chain, erythrocytic OS=Homo sapiens GN=SPTB	1				1		
P11908-2	PRPS2_HUMAN	35054	PRPS2	Isoform 2 of Ribose-phosphate pyrophosphokinase 2 OS=Homo sapiens GN=PRPS2	1				1		
P13073	COX41_HUMAN	19577	COX4I1	Cytochrome c oxidase subunit 4 isoform 1, mitochondrial OS=Homo sapiens GN=COX4I1 PE=1 SV=1	1				1		
P14735	IDE_HUMAN	117968	IDE	Insulin-degrading enzyme OS=Homo sapiens GN=IDE PE=1 SV=4	1				1		
P21108	PRPS3_HUMAN	34839	PRPS1L1	Ribose-phosphate pyrophosphokinase 3 OS=Homo sapiens GN=PRPS1L1 PE=1 SV=2	1				1		
P22531	SPR2E_HUMAN	7855	SPRR2E	Small proline-rich protein 2E OS=Homo sapiens GN=SPRR2E PE=2 SV=2	1				1		
P22532	SPR2D_HUMAN	7905	SPRR2D	Small proline-rich protein 2D OS=Homo sapiens GN=SPRR2D PE=2 SV=2	1				1		
P22695	QCR2_HUMAN	48443	UQCRC2	Cytochrome b-c1 complex subunit 2, mitochondrial OS=Homo sapiens GN=UQCRC2 PE=1 SV=3	1				1		
P23297	S10A1_HUMAN	10546	S100A1	Protein S100-A1 OS=Homo sapiens GN=S100A1 PE=1 SV=2	1				1		
P24941-2	CDK2_HUMAN	30035	CDK2	Isoform 2 of Cyclin-dependent kinase 2 OS=Homo sapiens GN=CDK2	1				1		
P26358-2	DNMT1_HUMAN	184819	DNMT1	Isoform 2 of DNA (cytosine-5)-methyltransferase 1 OS=Homo sapiens GN=DNMT1	1				1		
P30419-2	NMT1_HUMAN	48141	NMT1	Isoform Short of Glycylpeptide N-tetradecanoyltransferase 1 OS=Homo sapiens GN=NMT1	1				1		
P31350-2	RIR2_HUMAN	51093	RRM2	Isoform 2 of Ribonucleoside-diphosphate reductase subunit M2 OS=Homo sapiens GN=RRM2	1				1		
P35222	CTNB1_HUMAN	85497	CTNNB1	Catenin beta-1 OS=Homo sapiens GN=CTNNB1 PE=1 SV=1	1				1		
P35232	PHB_HUMAN	29804	PHB	Prohibitin OS=Homo sapiens GN=PHB PE=1 SV=1	1				1		
P35325	SPR2B_HUMAN	7975	SPRR2B	Small proline-rich protein 2B OS=Homo sapiens GN=SPRR2B PE=2 SV=1	1				1		
P35326	SPR2A_HUMAN	7965	SPRR2A	Small proline-rich protein 2A OS=Homo sapiens GN=SPRR2A PE=1 SV=1	1				1		
P36954	RPB9_HUMAN	14523	POLR2I	DNA-directed RNA polymerase II subunit RPB9 OS=Homo sapiens GN=POLR2I PE=1 SV=1	1				1		
P40306	PSB10_HUMAN	28936	PSMB10	Proteasome subunit beta type-10 OS=Homo sapiens GN=PSMB10 PE=1 SV=1	1				1		
P43243-2	MATR3_HUMAN	62576	MATR3	Isoform 2 of Matrin-3 OS=Homo sapiens GN=MATR3	1				1		
P43307-2	SSRA_HUMAN	29373	SSR1	Isoform 2 of Translocon-associated protein subunit alpha OS=Homo sapiens GN=SSR1	1				1		
P46531	NOTC1_HUMAN	272505	NOTCH1	Neurogenic locus notch homolog protein 1 OS=Homo sapiens GN=NOTCH1 PE=1 SV=4	1				1		
P48729-2	KC1A_HUMAN	41937	CSNK1A1	Isoform 2 of Casein kinase I isoform alpha OS=Homo sapiens GN=CSNK1A1	1				1		
P49368-2	TCPG_HUMAN	56431	CCT3	Isoform 2 of T-complex protein 1 subunit gamma OS=Homo sapiens GN=CCT3	1				1		

P60891	PRPS1_HUMAN	34834	PRPS1	Ribose-phosphate pyrophosphokinase 1 OS=Homo sapiens GN=PRPS1 PE=1 SV=2	1				1		
P61163	ACTZ_HUMAN	42614	ACTR1A	Alpha-centractin OS=Homo sapiens GN=ACTR1A PE=1 SV=1	1				1		
P61353	RL27_HUMAN	15798	RPL27	60S ribosomal protein L27 OS=Homo sapiens GN=RPL27 PE=1 SV=2	1				1		
P62917	RL8_HUMAN	28025	RPL8	60S ribosomal protein L8 OS=Homo sapiens GN=RPL8 PE=1 SV=2	1				1		
P83876	TXN4A_HUMAN	16786	TXNL4A	Thioredoxin-like protein 4A OS=Homo sapiens GN=TXNL4A PE=1 SV=1	1				1		
P84103	SRSF3_HUMAN	19330	SRSF3	Serine/arginine-rich splicing factor 3 OS=Homo sapiens GN=SRSF3 PE=1 SV=1	1				1		
Q00526	CDK3_HUMAN	35046	CDK3	Cyclin-dependent kinase 3 OS=Homo sapiens GN=CDK3 PE=1 SV=1	1				1		
Q03164-2	KMT2A_HUMAN	427733	KMT2A	Isoform 2 of Histone-lysine N-methyltransferase 2A OS=Homo sapiens GN=KMT2A	1				1		
Q04941	PLP2_HUMAN	16691	PLP2	Proteolipid protein 2 OS=Homo sapiens GN=PLP2 PE=1 SV=1	1				1		
Q13492-2	PICAL_HUMAN	68764	PICALM	Isoform 2 of Phosphatidylinositol-binding clathrin assembly protein OS=Homo sapiens GN=PICALM	1				1		
Q13835-2	PKP1_HUMAN	80497	PKP1	Isoform 1 of Plakophilin-1 OS=Homo sapiens GN=PKP1	1				1		
Q14004	CDK13_HUMAN	164923	CDK13	Cyclin-dependent kinase 13 OS=Homo sapiens GN=CDK13 PE=1 SV=2	1				1		
Q14165	MLEC_HUMAN	32234	MLEC	Malectin OS=Homo sapiens GN=MLEC PE=1 SV=1	1				1		
Q14320	FA50A_HUMAN	40242	FAM50A	Protein FAM50A OS=Homo sapiens GN=FAM50A PE=1 SV=2	1				1		
Q14764	MVP_HUMAN	99327	MVP	Major vault protein OS=Homo sapiens GN=MVP PE=1 SV=4	1				1		
Q15008-2	PSMD6_HUMAN	41252	PSMD6	Isoform 2 of 26S proteasome non-ATPase regulatory subunit 6 OS=Homo sapiens GN=PSMD6	1				1		
Q15427	SF3B4_HUMAN	44386	SF3B4	Splicing factor 3B subunit 4 OS=Homo sapiens GN=SF3B4 PE=1 SV=1	1				1		
Q15517	CDSN_HUMAN	51522	CDSN	Corneodesmosin OS=Homo sapiens GN=CDSN PE=1 SV=3	1				1		
Q16854-2	DGUOK_HUMAN	21439	DGUOK	Isoform 2 of Deoxyguanosine kinase, mitochondrial OS=Homo sapiens GN=DGUOK	1				1		
Q3V6T2-2	GRDN_HUMAN	212620	CCDC88A	Isoform 2 of Girdin OS=Homo sapiens GN=CCDC88A	1				1		
Q5JUX0	SPIN3_HUMAN	29207	SPIN3	Spindlin-3 OS=Homo sapiens GN=SPIN3 PE=1 SV=1	1				1		
Q63ZY3-2	KANK2_HUMAN	91921	KANK2	Isoform 2 of KN motif and ankyrin repeat domain-containing protein 2 OS=Homo sapiens GN=KANK2	1				1		
Q6DN03	H2B2C_HUMAN	21472	HIST2H2BC	Putative histone H2B type 2-C OS=Homo sapiens GN=HIST2H2BC PE=5 SV=3	1				1		
Q6DRA6	H2B2D_HUMAN	18018	HIST2H2BD	Putative histone H2B type 2-D OS=Homo sapiens GN=HIST2H2BD PE=5 SV=3	1				1		
Q7Z5L9-2	I2BP2_HUMAN	59481	IRF2BP2	Isoform 2 of Interferon regulatory factor 2-binding protein 2 OS=Homo sapiens GN=IRF2BP2	1				1		
Q8IZD9	DOCK3_HUMAN	233103	DOCK3	Dedicator of cytokinesis protein 3 OS=Homo sapiens GN=DOCK3 PE=1 SV=1	1				1		
Q8N2S1-2	LTBP4_HUMAN	165742	LTBP4	Isoform 2 of Latent-transforming growth factor beta-binding protein 4 OS=Homo sapiens GN=LTBP4	1				1		
Q8N5S1	S2541_HUMAN	40795	SLC25A41	Solute carrier family 25 member 41 OS=Homo sapiens GN=SLC25A41 PE=2 SV=2	1				1		
Q8N9W4-2	GG6L2_HUMAN	49583	GOLGA6L2	Isoform 2 of Golgin subfamily A member 6-like protein 2 OS=Homo sapiens GN=GOLGA6L2	1				1		
Q8NB90-2	SPAT5_HUMAN	86077	SPATA5	Isoform 2 of Spermatogenesis-associated protein 5 OS=Homo sapiens GN=SPATA5	1				1		
Q8TCD5	NT5C_HUMAN	23383	NT5C	5'(3')-deoxyribonucleotidase, cytosolic type OS=Homo sapiens GN=NT5C PE=1 SV=2	1				1		
Q8WW01-2	SEN15_HUMAN	13888	TSEN15	Isoform 2 of tRNA-splicing endonuclease subunit Sen15 OS=Homo sapiens GN=TSEN15	1				1		
Q92575	UBXN4_HUMAN	56778	UBXN4	UBX domain-containing protein 4 OS=Homo sapiens GN=UBXN4 PE=1 SV=2	1				1		
Q92620	PRP16_HUMAN	140503	DHX38	Pre-mRNA-splicing factor ATP-dependent RNA helicase PRP16 OS=Homo sapiens GN=DHX38 PE=1 SV=2	1				1		
Q96DB5	RMD1_HUMAN	35808	RMDN1	Regulator of microtubule dynamics protein 1 OS=Homo sapiens GN=RMDN1 PE=1 SV=1	1				1		
Q96GX9-3	MTNB_HUMAN	22858	APIP	Isoform 2 of Methylthioribulose-1-phosphate dehydratase OS=Homo sapiens GN=APIP	1				1		
Q96L21	RL10L_HUMAN	24519	RPL10L	60S ribosomal protein L10-like OS=Homo sapiens GN=RPL10L PE=1 SV=3	1				1		
Q96RE9-2	ZN300_HUMAN	70300	ZNF300	Isoform 2 of Zinc finger protein 300 OS=Homo sapiens GN=ZNF300	1				1		
Q96TA2-2	YMEL1_HUMAN	79832	YME1L1	Isoform 2 of ATP-dependent zinc metalloprotease YME1L1 OS=Homo sapiens GN=YME1L1	1				1		
Q9BWX1-	PHF7_HUMAN	39259	PHF7	Isoform 2 of PHD finger protein 7 OS=Homo sapiens GN=PHF7	1				1		

2											
Q9BYE4	SPR2G_HUMAN	8158	SPRR2G	Small proline-rich protein 2G OS=Homo sapiens GN=SPRR2G PE=2 SV=1	1				1		
Q9BZK3	NACP1_HUMAN	23306	NACAP1	Putative nascent polypeptide-associated complex subunit alpha-like protein OS=Homo sapiens GN=NACAP1 PE=5 SV=1	1				1		
Q9H098-2	F107B_HUMAN	34759	FAM107B	Isoform 2 of Protein FAM107B OS=Homo sapiens GN=FAM107B	1				1		
Q9H3H3-2	CK068_HUMAN	31430	C11orf68	Isoform 2 of UPF0696 protein C11orf68 OS=Homo sapiens GN=C11orf68	1				1		
Q9H8L6	MMRN2_HUMAN	104409	MMRN2	Multimerin-2 OS=Homo sapiens GN=MMRN2 PE=1 SV=2	1				1		
Q9H910-2	HN1L_HUMAN	18416	HN1L	Isoform 2 of Hematological and neurological expressed 1-like protein OS=Homo sapiens GN=HN1L	1				1		
Q9HBI1-2	PARVB_HUMAN	45183	PARVB	Isoform 2 of Beta-parvin OS=Homo sapiens GN=PARVB	1				1		
Q9HCB6	SPON1_HUMAN	90973	SPON1	Spondin-1 OS=Homo sapiens GN=SPON1 PE=1 SV=2	1				1		
Q9NP81	SYSM_HUMAN	58283	SARS2	Serine--tRNA ligase, mitochondrial OS=Homo sapiens GN=SARS2 PE=1 SV=1	1				1		
Q9NVA2-2	SEP11_HUMAN	50823	SEPT11	Isoform 2 of Septin-11 OS=Homo sapiens GN=SEPT11	1				1		
Q9NZR2	LRP1B_HUMAN	515498	LRP1B	Low-density lipoprotein receptor-related protein 1B OS=Homo sapiens GN=LRP1B PE=1 SV=2	1				1		
Q9NZT1	CALL5_HUMAN	15893	CALML5	Calmodulin-like protein 5 OS=Homo sapiens GN=CALML5 PE=1 SV=2	1				1		
Q9P2T1-2	GMPR2_HUMAN	39805	GMPR2	Isoform 2 of GMP reductase 2 OS=Homo sapiens GN=GMPR2	1				1		
Q9UBW7	ZMYM2_HUMAN	154911	ZMYM2	Zinc finger MYM-type protein 2 OS=Homo sapiens GN=ZMYM2 PE=1 SV=1	1				1		
Q9UDY4	DNJB4_HUMAN	37807	DNAJB4	DnaJ homolog subfamily B member 4 OS=Homo sapiens GN=DNAJB4 PE=1 SV=1	1				1		
Q9Y247	FA50B_HUMAN	38709	FAM50B	Protein FAM50B OS=Homo sapiens GN=FAM50B PE=1 SV=1	1				1		
Q9Y263	PLAP_HUMAN	87157	PLAA	Phospholipase A-2-activating protein OS=Homo sapiens GN=PLAA PE=1 SV=2	1				1		
Q9Y2S6	TMA7_HUMAN	7066	TMA7	Translation machinery-associated protein 7 OS=Homo sapiens GN=TMA7 PE=1 SV=1	1				1		
Q9Y316-2	MEMO1_HUMAN	31307	MEMO1	Isoform 2 of Protein MEMO1 OS=Homo sapiens GN=MEMO1	1				1		
Q9Y3D6	FIS1_HUMAN	16938	FIS1	Mitochondrial fission 1 protein OS=Homo sapiens GN=FIS1 PE=1 SV=2	1				1		
Q9Y4E8-2	UBP15_HUMAN	109297	USP15	Isoform 2 of Ubiquitin carboxyl-terminal hydrolase 15 OS=Homo sapiens GN=USP15	1				1		
Q9Y657	SPIN1_HUMAN	29601	SPIN1	Spindlin-1 OS=Homo sapiens GN=SPIN1 PE=1 SV=3	1				1		
A8MSS1	A8MSS1_HUMAN	10156	FKBP1C	Peptidyl-prolyl cis-trans isomerase OS=Homo sapiens GN=FKBP1C PE=2 SV=2	1				1		
B4E241	B4E241_HUMAN	14203	SFRS3	Serine/arginine-rich-splicing factor 3 OS=Homo sapiens GN=SFRS3 PE=2 SV=1	1				1		
J3KPZ3	J3KPZ3_HUMAN	513636	MYCBP2	Probable E3 ubiquitin-protein ligase MYCBP2 OS=Homo sapiens GN=MYCBP2 PE=2 SV=1	1				1		
Q567R9	Q567R9_HUMAN	16830	CIP29	CIP29 protein OS=Homo sapiens GN=CIP29 PE=2 SV=1	1				1		
O43598	DNPH1_HUMAN	19108	DNPH1	2'-deoxynucleoside 5'-phosphate N-hydrolase 1 OS=Homo sapiens GN=DNPH1 PE=1 SV=1	1					1	
O60271-2	JIP4_HUMAN	145135	SPAG9	Isoform 2 of C-Jun-amino-terminal kinase-interacting protein 4 OS=Homo sapiens GN=SPAG9	1					1	
O60841	IF2P_HUMAN	138827	EIF5B	Eukaryotic translation initiation factor 5B OS=Homo sapiens GN=EIF5B PE=1 SV=4	1					1	
O75077-2	ADA23_HUMAN	92046	ADAM23	Isoform Beta of Disintegrin and metalloproteinase domain-containing protein 23 OS=Homo sapiens GN=ADAM23	1					1	
O75348	VATG1_HUMAN	13758	ATP6V1G1	V-type proton ATPase subunit G 1 OS=Homo sapiens GN=ATP6V1G1 PE=1 SV=3	1					1	
O75717	WDHD1_HUMAN	125967	WDHD1	WD repeat and HMG-box DNA-binding protein 1 OS=Homo sapiens GN=WDHD1 PE=1 SV=1	1					1	
O76071	CIAO1_HUMAN	37840	CIAO1	Probable cytosolic iron-sulfur protein assembly protein CIAO1 OS=Homo sapiens GN=CIAO1 PE=1 SV=1	1					1	
O76081-6	RGS20_HUMAN	27060	RGS20	Isoform 5 of Regulator of G-protein signaling 20 OS=Homo sapiens GN=RGS20	1					1	
O95372	LYPA2_HUMAN	24737	LYPLA2	Acyl-protein thioesterase 2 OS=Homo sapiens GN=LYPLA2 PE=1 SV=1	1					1	
O95670-2	VATG2_HUMAN	8954	ATP6V1G2	Isoform 2 of V-type proton ATPase subunit G 2 OS=Homo sapiens GN=ATP6V1G2	1					1	
O95758-1	PTBP3_HUMAN	56502	PTBP3	Isoform 1 of Polypyrimidine tract-binding protein 3 OS=Homo sapiens GN=PTBP3	1					1	
P01591	IGJ_HUMAN	18099	IGJ	Immunoglobulin J chain OS=Homo sapiens GN=IGJ PE=1 SV=4	1					1	
P01833	PIGR_HUMAN	83284	PIGR	Polymeric immunoglobulin receptor OS=Homo sapiens GN=PIGR PE=1 SV=4	1					1	
P02776	PLF4_HUMAN	10845	PF4	Platelet factor 4 OS=Homo sapiens GN=PF4 PE=1 SV=2	1					1	
P08174-2	DAF_HUMAN	48717	CD55	Isoform 1 of Complement decay-accelerating factor OS=Homo sapiens GN=CD55	1					1	

P15927-2	RFA2_HUMAN	30156	RPA2	Isoform 2 of Replication protein A 32 kDa subunit OS=Homo sapiens GN=RPA2	1					1	
P17987	TCPA_HUMAN	60344	TCP1	T-complex protein 1 subunit alpha OS=Homo sapiens GN=TCP1 PE=1 SV=1	1					1	
P34741	SDC2_HUMAN	22160	SDC2	Syndecan-2 OS=Homo sapiens GN=SDC2 PE=1 SV=2	1					1	
P40227-2	TCPZ_HUMAN	53289	CCT6A	Isoform 2 of T-complex protein 1 subunit zeta OS=Homo sapiens GN=CCT6A	1					1	
P41208	CETN2_HUMAN	19738	CETN2	Centrin-2 OS=Homo sapiens GN=CETN2 PE=1 SV=1	1					1	
P48509	CD151_HUMAN	28295	CD151	CD151 antigen OS=Homo sapiens GN=CD151 PE=1 SV=3	1					1	
P49591	SYSC_HUMAN	58777	SARS	Serine--tRNA ligase, cytoplasmic OS=Homo sapiens GN=SARS PE=1 SV=3	1					1	
P51809-2	VAMP7_HUMAN	30217	VAMP7	Isoform 2 of Vesicle-associated membrane protein 7 OS=Homo sapiens GN=VAMP7	1					1	
P52848-2	NDST1_HUMAN	62065	NDST1	Isoform 2 of Bifunctional heparan sulfate N-deacetylase/N-sulfotransferase 1 OS=Homo sapiens GN=NDST1	1					1	
P53618	COPB_HUMAN	107142	COPB1	Coatomer subunit beta OS=Homo sapiens GN=COPB1 PE=1 SV=3	1					1	
P54136-2	SYRC_HUMAN	67140	RARS	Isoform Monomeric of Arginine--tRNA ligase, cytoplasmic OS=Homo sapiens GN=RARS	1					1	
P55769	NH2L1_HUMAN	14174	NHP2L1	NHP2-like protein 1 OS=Homo sapiens GN=NHP2L1 PE=1 SV=3	1					1	
P62333	PRS10_HUMAN	44173	PSMC6	26S protease regulatory subunit 10B OS=Homo sapiens GN=PSMC6 PE=1 SV=1	1					1	
P62899-2	RL31_HUMAN	14632	RPL31	Isoform 2 of 60S ribosomal protein L31 OS=Homo sapiens GN=RPL31	1					1	
Q08211	DHX9_HUMAN	140958	DHX9	ATP-dependent RNA helicase A OS=Homo sapiens GN=DHX9 PE=1 SV=4	1					1	
Q12888-2	TP53B_HUMAN	214117	TP53BP1	Isoform 2 of Tumor suppressor p53-binding protein 1 OS=Homo sapiens GN=TP53BP1	1					1	
Q12913-2	PTPRJ_HUMAN	57190	PTPRJ	Isoform 2 of Receptor-type tyrosine-protein phosphatase eta OS=Homo sapiens GN=PTPRJ	1					1	
Q13724-2	MOGS_HUMAN	80703	MOGS	Isoform 2 of Mannosyl-oligosaccharide glucosidase OS=Homo sapiens GN=MOGS	1					1	
Q14114-3	LRP8_HUMAN	99092	LRP8	Isoform 3 of Low-density lipoprotein receptor-related protein 8 OS=Homo sapiens GN=LRP8	1					1	
Q2LD37-4	K1109_HUMAN	555354	KIAA1109	Isoform 4 of Uncharacterized protein KIAA1109 OS=Homo sapiens GN=KIAA1109	1					1	
Q5JS54-2	PSMG4_HUMAN	17656	PSMG4	Isoform 2 of Proteasome assembly chaperone 4 OS=Homo sapiens GN=PSMG4	1					1	
Q66K74	MAP1S_HUMAN	112211	MAP1S	Microtubule-associated protein 1S OS=Homo sapiens GN=MAP1S PE=1 SV=2	1					1	
Q7Z7H5-3	TMED4_HUMAN	20781	TMED4	Isoform 3 of Transmembrane emp24 domain-containing protein 4 OS=Homo sapiens GN=TMED4	1					1	
Q8IZ52-2	CHSS2_HUMAN	34955	CHPF	Isoform 2 of Chondroitin sulfate synthase 2 OS=Homo sapiens GN=CHPF	1					1	
Q8N4A0-2	GALT4_HUMAN	45702	GALNT4	Isoform 2 of Polypeptide N-acetylgalactosaminyltransferase 4 OS=Homo sapiens GN=GALNT4	1					1	
Q8NF91-4	SYNE1_HUMAN	1005239	SYNE1	Isoform 4 of Nesprin-1 OS=Homo sapiens GN=SYNE1	1					1	
Q8NFC6	BD1L1_HUMAN	330466	BOD1L1	Biorientation of chromosomes in cell division protein 1-like 1 OS=Homo sapiens GN=BOD1L1 PE=1 SV=2	1					1	
Q8TDP1-2	RNH2C_HUMAN	17712	RNASEH2C	Isoform 2 of Ribonuclease H2 subunit C OS=Homo sapiens GN=RNASEH2C	1					1	
Q96EM0	T3HPD_HUMAN	38138	L3HYPDH	Trans-L-3-hydroxyproline dehydratase OS=Homo sapiens GN=L3HYPDH PE=1 SV=2	1					1	
Q99447-3	PCY2_HUMAN	45622	PCYT2	Isoform 3 of Ethanolamine-phosphate cytidylyltransferase OS=Homo sapiens GN=PCYT2	1					1	
Q9BSG0	PADC1_HUMAN	21042	PRADC1	Protease-associated domain-containing protein 1 OS=Homo sapiens GN=PRADC1 PE=1 SV=1	1					1	
Q9BUN1-2	MENT_HUMAN	18583	MENT	Isoform 2 of Protein MENT OS=Homo sapiens GN=MENT	1					1	
Q9BVM4	GGACT_HUMAN	17329	GGACT	Gamma-glutamylaminecyclotransferase OS=Homo sapiens GN=GGACT PE=1 SV=2	1					1	
Q9BXI9-2	C1QT6_HUMAN	30861	C1QTNF6	Isoform 2 of Complement C1q tumor necrosis factor-related protein 6 OS=Homo sapiens GN=C1QTNF6	1					1	
Q9C0C4	SEM4C_HUMAN	92623	SEMA4C	Semaphorin-4C OS=Homo sapiens GN=SEMA4C PE=1 SV=2	1					1	
Q9H0R5-4	GBP3_HUMAN	61928	GBP3	Isoform 2 of Guanylate-binding protein 3 OS=Homo sapiens GN=GBP3	1					1	
Q9H6B4	CLMP_HUMAN	41281	CLMP	CXADR-like membrane protein OS=Homo sapiens GN=CLMP PE=1 SV=1	1					1	
Q9NRN7	ADPPT_HUMAN	35776	AASDHPPT	L-aminoadipate-semialdehyde dehydrogenase-phosphopantetheinyl transferase OS=Homo sapiens GN=AASDHPPT PE=1 SV=2	1					1	
Q9NRP0-2	OSTC_HUMAN	19171	OSTC	Isoform 2 of Oligosaccharyltransferase complex subunit OSTC OS=Homo sapiens	1					1	

				GN=OSTC							
Q9NVS9	PNPO_HUMAN	29988	PNPO	Pyridoxine-5'-phosphate oxidase OS=Homo sapiens GN=PNPO PE=1 SV=1	1					1	
Q9P2K2	TXD16_HUMAN	93572	TXNDC16	Thioredoxin domain-containing protein 16 OS=Homo sapiens GN=TXNDC16 PE=2 SV=4	1					1	
Q9UBF6-2	RBX2_HUMAN	11557	RNF7	Isoform 2 of RING-box protein 2 OS=Homo sapiens GN=RNF7	1					1	
Q9UI30	TR112_HUMAN	14199	TRMT112	tRNA methyltransferase 112 homolog OS=Homo sapiens GN=TRMT112 PE=1 SV=1	1					1	
Q9UPA5	BSN_HUMAN	416469	BSN	Protein bassoon OS=Homo sapiens GN=BSN PE=2 SV=4	1					1	
Q9Y4U1	MMAC_HUMAN	31728	MMACHC	Methylmalonic aciduria and homocystinuria type C protein OS=Homo sapiens GN=MMACHC PE=1 SV=3	1					1	
Q9Y6N8	CAD10_HUMAN	88451	CDH10	Cadherin-10 OS=Homo sapiens GN=CDH10 PE=1 SV=2	1					1	
F2Z307	F2Z307_HUMAN	11355	ATP6V1G2-DDX39B	Protein ATP6V1G2-DDX39B OS=Homo sapiens GN=ATP6V1G2-DDX39B PE=4 SV=1	1					1	
F8VUJ3	F8VUJ3_HUMAN	66238	POC1B-GALNT4	Protein POC1B-GALNT4 OS=Homo sapiens GN=POC1B-GALNT4 PE=2 SV=1	1					1	
O43175	SERA_HUMAN	56650	PHGDH	D-3-phosphoglycerate dehydrogenase OS=Homo sapiens GN=PHGDH PE=1 SV=4	1						1
O43294-2	TGFI1_HUMAN	47941	TGFB1I1	Isoform 2 of Transforming growth factor beta-1-induced transcript 1 protein OS=Homo sapiens GN=TGFB1I1	1						1
O43692	PI15_HUMAN	29065	PI15	Peptidase inhibitor 15 OS=Homo sapiens GN=PI15 PE=1 SV=1	1						1
O43847-2	NRDC_HUMAN	139284	NRD1	Isoform 2 of Nardilysin OS=Homo sapiens GN=NRD1	1						1
O95150-2	TNF15_HUMAN	21857	TNFSF15	Isoform 2 of Tumor necrosis factor ligand superfamily member 15 OS=Homo sapiens GN=TNFSF15	1						1
P04066	FUCO_HUMAN	53689	FUCA1	Tissue alpha-L-fucosidase OS=Homo sapiens GN=FUCA1 PE=1 SV=4	1						1
P05090	APOD_HUMAN	21276	APOD	Apolipoprotein D OS=Homo sapiens GN=APOD PE=1 SV=1	1						1
P17516	AK1C4_HUMAN	37067	AKR1C4	Aldo-keto reductase family 1 member C4 OS=Homo sapiens GN=AKR1C4 PE=1 SV=3	1						1
P22090	RS4Y1_HUMAN	29456	RPS4Y1	40S ribosomal protein S4, Y isoform 1 OS=Homo sapiens GN=RPS4Y1 PE=1 SV=2	1						1
P22492	H1T_HUMAN	22019	HIST1H1T	Histone H1t OS=Homo sapiens GN=HIST1H1T PE=2 SV=4	1						1
P50502	F10A1_HUMAN	41332	ST13	Hsc70-interacting protein OS=Homo sapiens GN=ST13 PE=1 SV=2	1						1
P55212-2	CASP6_HUMAN	22574	CASP6	Isoform Beta of Caspase-6 OS=Homo sapiens GN=CASP6	1						1
P61024	CKS1_HUMAN	9660	CKS1B	Cyclin-dependent kinases regulatory subunit 1 OS=Homo sapiens GN=CKS1B PE=1 SV=1	1						1
Q02325	PLGB_HUMAN	10971	PLGLB1	Plasminogen-like protein B OS=Homo sapiens GN=PLGLB1 PE=1 SV=1	1						1
Q02539	H11_HUMAN	21842	HIST1H1A	Histone H1.1 OS=Homo sapiens GN=HIST1H1A PE=1 SV=3	1						1
Q13315	ATM_HUMAN	350687	ATM	Serine-protein kinase ATM OS=Homo sapiens GN=ATM PE=1 SV=4	1						1
Q14982-2	OPCM_HUMAN	37267	OPCML	Isoform 2 of Opioid-binding protein/cell adhesion molecule OS=Homo sapiens GN=OPCML	1						1
Q15195	PLGA_HUMAN	10915	PLGLA	Plasminogen-like protein A OS=Homo sapiens GN=PLGLA PE=2 SV=1	1						1
Q15389-2	ANGP1_HUMAN	57456	ANGPT1	Isoform 2 of Angiopoietin-1 OS=Homo sapiens GN=ANGPT1	1						1
Q15424-2	SAFB1_HUMAN	95181	SAFB	Isoform 2 of Scaffold attachment factor B1 OS=Homo sapiens GN=SAFB	1						1
Q16222-2	UAP1_HUMAN	57028	UAP1	Isoform AGX1 of UDP-N-acetylhexosamine pyrophosphorylase OS=Homo sapiens GN=UAP1	1						1
Q16864-2	VATF_HUMAN	16402	ATP6V1F	Isoform 2 of V-type proton ATPase subunit F OS=Homo sapiens GN=ATP6V1F	1						1
Q6YHU6-2	THADA_HUMAN	183444	THADA	Isoform 2 of Thyroid adenoma-associated protein OS=Homo sapiens GN=THADA	1						1
Q6ZVC0-2	NYAP1_HUMAN	88030	NYAP1	Isoform 2 of Neuronal tyrosine-phosphorylated phosphoinositide-3-kinase adapter 1 OS=Homo sapiens GN=NYAP1	1						1
Q8IXL7-2	MSRB3_HUMAN	20010	MSRB3	Isoform 2 of Methionine-R-sulfoxide reductase B3 OS=Homo sapiens GN=MSRB3	1						1
Q8IZP2	ST134_HUMAN	27407	ST13P4	Putative protein FAM10A4 OS=Homo sapiens GN=ST13P4 PE=5 SV=1	1						1
Q8NE31-3	FA13C_HUMAN	55007	FAM13C	Isoform 3 of Protein FAM13C OS=Homo sapiens GN=FAM13C	1						1
Q8TD47	RS4Y2_HUMAN	29295	RPS4Y2	40S ribosomal protein S4, Y isoform 2 OS=Homo sapiens GN=RPS4Y2 PE=2 SV=3	1						1
Q99674-4	CGRE1_HUMAN	33456	CGREF1	Isoform 4 of Cell growth regulator with EF hand domain protein 1 OS=Homo sapiens GN=CGREF1	1						1
Q99733-2	NP1L4_HUMAN	44079	NAP1L4	Isoform 2 of Nucleosome assembly protein 1-like 4 OS=Homo sapiens GN=NAP1L4	1						1

Q9BR76	COR1B_HUMAN	54235	CORO1B	Coronin-1B OS=Homo sapiens GN=CORO1B PE=1 SV=1	1						1
Q9BRR6-2	ADPGK_HUMAN	53961	ADPGK	Isoform 2 of ADP-dependent glucokinase OS=Homo sapiens GN=ADPGK	1						1
Q9NP77-2	SSU72_HUMAN	16931	SSU72	Isoform 2 of RNA polymerase II subunit A C-terminal domain phosphatase SSU72 OS=Homo sapiens GN=SSU72	1						1
Q9NS86	LANC2_HUMAN	50854	LANCL2	LanC-like protein 2 OS=Homo sapiens GN=LANCL2 PE=1 SV=1	1						1
Q9NZR1	TMOD2_HUMAN	39595	TMOD2	Tropomodulin-2 OS=Homo sapiens GN=TMOD2 PE=1 SV=1	1						1
Q9NZU5	LMCD1_HUMAN	40833	LMCD1	LIM and cysteine-rich domains protein 1 OS=Homo sapiens GN=LMCD1 PE=1 SV=1	1						1
Q9P203-5	BTBD7_HUMAN	85546	BTBD7	Isoform 5 of BTB/POZ domain-containing protein 7 OS=Homo sapiens GN=BTBD7	1						1
Q9UBS3	DNJB9_HUMAN	25518	DNAJB9	DnaJ homolog subfamily B member 9 OS=Homo sapiens GN=DNAJB9 PE=1 SV=1	1						1
Q9UNE7-2	CHIP_HUMAN	27067	STUB1	Isoform 2 of E3 ubiquitin-protein ligase CHIP OS=Homo sapiens GN=STUB1	1						1
Q9Y2W1	TR150_HUMAN	108666	THRAP3	Thyroid hormone receptor-associated protein 3 OS=Homo sapiens GN=THRAP3 PE=1 SV=2	1						1
Q9Y5G5-2	PCDG8_HUMAN	89865	PCDHGA8	Isoform 2 of Protocadherin gamma-A8 OS=Homo sapiens GN=PCDHGA8	1						1
Q9Y5H4-2	PCDG1_HUMAN	90160	PCDHGA1	Isoform 2 of Protocadherin gamma-A1 OS=Homo sapiens GN=PCDHGA1	1						1
H3BQK2	H3BQK2_HUMAN	63945	PARP6	Poly [ADP-ribose] polymerase 6 (Fragment) OS=Homo sapiens GN=PARP6 PE=4 SV=1	1						1
Q8IY32	Q8IY32_HUMAN	100685	tmp_locus_38	Putative uncharacterized protein tmp_locus_38 OS=Homo sapiens GN=tmp_locus_38 PE=2 SV=1	1						1
Q96P63	SPB12_HUMAN	46276	SERPINB12	Serpin B12 OS=Homo sapiens GN=SERPINB12 PE=1 SV=1	1				0		
