# Supplementary material for: Proteomic Identification and Time-Course Monitoring of Secreted Proteins During Expansion of Human Mesenchymal Stem/Stromal in Stirred-Tank Bioreactor
Source: Front Bioeng Biotechnol. 2019 Jun 26;7:154. doi: 10.3389/fbioe.2019.00154 (PMC6607109; doi:10.3389/fbioe.2019.00154)
Supplement: Supplementary Table 2 — Multiple Reaction Monitoring (MRM) parameters for quantification of target proteins in cell supernatants. [file Data_Sheet_3.PDF]

| Protein Gene | Protein Name | Peptide Modified Sequence | Precursor Mz | Precursor Charge | Collision Energy | Product Mz | Product Charge | Fragment Ion |
|---|---|---|---|---|---|---|---|---|
| TGFBI BIGH3 | Q15582 | LTLLAPLNSVFK | 658,811971 | 2 | 23,3 | 1101,666657 | 1 | y10 |
| TGFBI BIGH3 | Q15582 | LTLLAPLNSVFK | 658,811971 | 2 | 23,3 | 988,582593 | 1 | y9 |
| TGFBI BIGH3 | Q15582 | LTLLAPLNSVFK | 658,811971 | 2 | 23,3 | 875,498529 | 1 | y8 |
| TGFBI BIGH3 | Q15582 | LTLLAPLNSVFK | 658,811971 | 2 | 23,3 | 804,461415 | 1 | y7 |
| TGFB1 TGFB | P01137 | EAVPEPVLLSR | 605,707266 | 2 | 21,3 | 910,535643 | 1 | y8 |
| TGFB1 TGFB | P01137 | EAVPEPVLLSR | 605,707266 | 2 | 21,3 | 813,482879 | 1 | y7 |
| TGFB1 TGFB | P01137 | EAVPEPVLLSR | 605,707266 | 2 | 21,3 | 684,440286 | 1 | y6 |
| TGFB1 TGFB | P01137 | EAVPEPVLLSR | 605,707266 | 2 | 21,3 | 488,319108 | 1 | y4 |
| COL1A2 | P08123 | GFPGTPGLPGFK | 588,185361 | 2 | 20,7 | 873,482879 | 1 | y9 |
| COL1A2 | P08123 | GFPGTPGLPGFK | 588,185361 | 2 | 20,7 | 715,413737 | 1 | y7 |
| COL1A2 | P08123 | GFPGTPGLPGFK | 588,185361 | 2 | 20,7 | 448,255445 | 1 | y4 |
| COL1A2 | P08123 | GFPGTPGLPGFK | 588,185361 | 2 | 20,7 | 358,210506 | 2 | y7 |
| COL3A1 | P02461 | DGSPGEPGANGLPGAAGER | 855,382981 | 2 | 30,6 | 560,2787 | 1 | y6 |
| COL3A1 | P02461 | DGSPGEPGANGLPGAAGER | 855,382981 | 2 | 30,6 | 329,16937 | 2 | y7 |
| COL3A1 | P02461 | DGSPGEPGANGLPGAAGER | 855,382981 | 2 | 30,6 | 543,204532 | 1 | b6 |
| COL1A1 | P02452 | GPAGPQGPR | 418,963691 | 2 | 14,4 | 682,363098 | 1 | y7 |
| COL1A1 | P02452 | GPAGPQGPR | 418,963691 | 2 | 14,4 | 611,325985 | 1 | y6 |
| COL1A1 | P02452 | GPAGPQGPR | 418,963691 | 2 | 14,4 | 554,304521 | 1 | y5 |
| IGFBP2 BP2 IBP2 | P18065 | TPC[+57.1]QQELDQVLER | 539,598443 | 3 | 18,1 | 759,399543 | 1 | y6 |
| IGFBP2 BP2 IBP2 | P18065 | TPC[+57.1]QQELDQVLER | 539,598443 | 3 | 18,1 | 516,314023 | 1 | y4 |
| IGFBP2 BP2 IBP2 | P18065 | TPC[+57.1]QQELDQVLER | 539,598443 | 3 | 18,1 | 417,245609 | 1 | y3 |
| IGFBP2 BP2 IBP2 | P18065 | TPC[+57.1]QQELDQVLER | 539,598443 | 3 | 18,1 | 744,298115 | 1 | b6 |
| IGFBP3 IBP3 | P17936 | AYLLPAPPAPGNASESEEDR | 695,745016 | 3 | 23,7 | 868,90793 | 2 | y17 |
| IGFBP3 IBP3 | P17936 | AYLLPAPPAPGNASESEEDR | 695,745016 | 3 | 23,7 | 812,365898 | 2 | y16 |
| IGFBP3 IBP3 | P17936 | AYLLPAPPAPGNASESEEDR | 695,745016 | 3 | 23,7 | 728,320959 | 2 | y14 |
| IGFBP3 IBP3 | P17936 | AYLLPAPPAPGNASESEEDR | 695,745016 | 3 | 23,7 | 595,749638 | 2 | y11 |
| IGFBP4 IBP4 | P22692 | LPGGLEPK | 405,984961 | 2 | 14 | 697,387916 | 1 | y7 |
| IGFBP4 IBP4 | P22692 | LPGGLEPK | 405,984961 | 2 | 14 | 244,165568 | 1 | y2 |
| IGFBP4 IBP4 | P22692 | LPGGLEPK | 405,984961 | 2 | 14 | 349,197596 | 2 | y7 |
| IGFBP4 IBP4 | P22692 | LPGGLEPK | 405,984961 | 2 | 14 | 325,187031 | 1 | b4 |
| IGFBP5 IBP5 | P24593 | FVGGAENTAHPR | 419,456403 | 3 | 13,8 | 480,267741 | 1 | y4 |
| IGFBP5 IBP5 | P24593 | FVGGAENTAHPR | 419,456403 | 3 | 13,8 | 272,171716 | 1 | y2 |
| IGFBP5 IBP5 | P24593 | FVGGAENTAHPR | 419,456403 | 3 | 13,8 | 505,244129 | 2 | y10 |
| IGFBP5 IBP5 | P24593 | FVGGAENTAHPR | 419,456403 | 3 | 13,8 | 247,144104 | 1 | b2 |
| IGFBP6 IBP6 | P24592 | HLDSVLQQLQTEVYR | 610,684196 | 3 | 20,7 | 908,483607 | 1 | y7 |
| IGFBP6 IBP6 | P24592 | HLDSVLQQLQTEVYR | 610,684196 | 3 | 20,7 | 795,399543 | 1 | y6 |
| IGFBP6 IBP6 | P24592 | HLDSVLQQLQTEVYR | 610,684196 | 3 | 20,7 | 667,340966 | 1 | y5 |
| IGFBP6 IBP6 | P24592 | HLDSVLQQLQTEVYR | 610,684196 | 3 | 20,7 | 921,478856 | 1 | b8 |
| IGFBP7 | Q16270 | AGAAAGGPGVSGVC[+57.1]VC[+57.1]K | 759,876781 | 2 | 27 | 1176,54999 | 1 | y12 |
| IGFBP7 | Q16270 | AGAAAGGPGVSGVC[+57.1]VC[+57.1]K | 759,876781 | 2 | 27 | 809,364421 | 1 | y7 |
| IGFBP7 | Q16270 | AGAAAGGPGVSGVC[+57.1]VC[+57.1]K | 759,876781 | 2 | 27 | 200,102967 | 1 | b3 |
| IGFBP7 | Q16270 | AGAAAGGPGVSGVC[+57.1]VC[+57.1]K | 759,876781 | 2 | 27 | 271,140081 | 1 | b4 |
| IL6 IFNB2 | P05231 | NLDAITTPDPTTNASLLTK | 663,071879 | 3 | 22,5 | 629,337892 | 2 | y12 |
| IL6 IFNB2 | P05231 | NLDAITTPDPTTNASLLTK | 663,071879 | 3 | 22,5 | 523,298038 | 2 | y10 |
| IL6 IFNB2 | P05231 | NLDAITTPDPTTNASLLTK | 663,071879 | 3 | 22,5 | 228,134267 | 1 | b2 |
| IL6 IFNB2 | P05231 | NLDAITTPDPTTNASLLTK | 663,071879 | 3 | 22,5 | 414,198324 | 1 | b4 |
| CXCL1 GRO GRO1 GROA MGSA SCYB1 | P09341 | NIQSVNVK | 451,519686 | 2 | 15,6 | 360,224145 | 1 | y3 |
| CXCL1 GRO GRO1 GROA MGSA SCYB1 | P09341 | NIQSVNVK | 451,519686 | 2 | 15,6 | 246,181218 | 1 | y2 |
| CXCL1 GRO GRO1 GROA MGSA SCYB1 | P09341 | NIQSVNVK | 451,519686 | 2 | 15,6 | 228,134267 | 1 | b2 |
| CXCL1 GRO GRO1 GROA MGSA SCYB1 | P09341 | NIQSVNVK | 451,519686 | 2 | 15,6 | 656,336215 | 1 | b6 |
| CCL2 MCP1 SCYA2 | P13500 | EIC[+57.1]ADPK | 416,976766 | 2 | 14,4 | 430,229624 | 1 | y4 |
| CCL2 MCP1 SCYA2 | P13500 | EIC[+57.1]ADPK | 416,976766 | 2 | 14,4 | 359,192511 | 1 | y3 |
| CCL2 MCP1 SCYA2 | P13500 | EIC[+57.1]ADPK | 416,976766 | 2 | 14,4 | 295,633774 | 2 | y5 |
| CCL2 MCP1 SCYA2 | P13500 | EIC[+57.1]ADPK | 416,976766 | 2 | 14,4 | 215,61845 | 2 | y4 |
| CXCL3 | P19876 | SPGPHC[+57.1]AQTEVIATLK | 855,479321 | 2 | 30,6 | 874,52441 | 1 | y8 |
| CXCL3 | P19876 | SPGPHC[+57.1]AQTEVIATLK | 855,479321 | 2 | 30,6 | 644,434138 | 1 | y6 |
| CXCL3 | P19876 | SPGPHC[+57.1]AQTEVIATLK | 855,479321 | 2 | 30,6 | 545,365724 | 1 | y5 |
| CXCL3 | P19876 | SPGPHC[+57.1]AQTEVIATLK | 855,479321 | 2 | 30,6 | 432,28166 | 1 | y4 |
| SPARC ON | P09486 | LEAGDHPVELLAR | 474,204336 | 3 | 15,7 | 700,435201 | 1 | y6 |
| SPARC ON | P09486 | LEAGDHPVELLAR | 474,204336 | 3 | 15,7 | 601,366787 | 1 | y5 |
| SPARC ON | P09486 | LEAGDHPVELLAR | 474,204336 | 3 | 15,7 | 653,841133 | 2 | y12 |
| SPARC ON | P09486 | LEAGDHPVELLAR | 474,204336 | 3 | 15,7 | 243,133933 | 1 | b2 |
| TIMP1 CLGI TIMP | P01033 | GFQALGDAADIR | 617,676481 | 2 | 21,8 | 901,473771 | 1 | y9 |
| TIMP1 CLGI TIMP | P01033 | GFQALGDAADIR | 617,676481 | 2 | 21,8 | 830,436657 | 1 | y8 |
| TIMP1 CLGI TIMP | P01033 | GFQALGDAADIR | 617,676481 | 2 | 21,8 | 717,352593 | 1 | y7 |
| TIMP2 | P16035 | QEFLDIEDP | 553,586741 | 2 | 19,4 | 701,335212 | 1 | y6 |
| TIMP2 | P16035 | QEFLDIEDP | 553,586741 | 2 | 19,4 | 588,251148 | 1 | y5 |
| TIMP2 | P16035 | QEFLDIEDP | 553,586741 | 2 | 19,4 | 473,224205 | 1 | y4 |
| TIMP2 | P16035 | QEFLDIEDP | 553,586741 | 2 | 19,4 | 360,140141 | 1 | y3 |
| THBS1 TSP TSP1 | P07996 | FTGSQPFGQGVEHATANK | 626,341603 | 3 | 21,2 | 869,447556 | 1 | y8 |
| THBS1 TSP TSP1 | P07996 | FTGSQPFGQGVEHATANK | 626,341603 | 3 | 21,2 | 770,379142 | 1 | y7 |
| THBS1 TSP TSP1 | P07996 | FTGSQPFGQGVEHATANK | 626,341603 | 3 | 21,2 | 641,336549 | 1 | y6 |
| THBS1 TSP TSP1 | P07996 | FTGSQPFGQGVEHATANK | 626,341603 | 3 | 21,2 | 504,277637 | 1 | y5 |
| FN1 | P02751 | VTWAPPPSIDLTNFLVR | 964,123251 | 2 | 34,6 | 977,541457 | 1 | y8 |
| FN1 | P02751 | VTWAPPPSIDLTNFLVR | 964,123251 | 2 | 34,6 | 648,382771 | 1 | y5 |
| FN1 | P02751 | VTWAPPPSIDLTNFLVR | 964,123251 | 2 | 34,6 | 734,911558 | 2 | y13 |
| FN1 | P02751 | VTWAPPPSIDLTNFLVR | 964,123251 | 2 | 34,6 | 686,385176 | 2 | y12 |
| MMP1 | P03956 | AFQLWSNVTPLTFTK | 877,516276 | 2 | 31,4 | 1107,604451 | 1 | y10 |
| MMP1 | P03956 | AFQLWSNVTPLTFTK | 877,516276 | 2 | 31,4 | 807,461081 | 1 | y7 |
| MMP1 | P03956 | AFQLWSNVTPLTFTK | 877,516276 | 2 | 31,4 | 706,413403 | 1 | y6 |
| MMP1 | P03956 | AFQLWSNVTPLTFTK | 877,516276 | 2 | 31,4 | 347,171381 | 1 | b3 |
| MMP14 | P50281 | FYGLQVTGK | 507,090436 | 2 | 17,7 | 404,25036 | 1 | y4 |
| MMP14 | P50281 | FYGLQVTGK | 507,090436 | 2 | 17,7 | 305,181946 | 1 | y3 |
| MMP14 | P50281 | FYGLQVTGK | 507,090436 | 2 | 17,7 | 204,134267 | 1 | y2 |
| MMP14 | P50281 | FYGLQVTGK | 507,090436 | 2 | 17,7 | 311,139019 | 1 | b2 |
